# Supplementary material for: Implementation and impact of mhealth in the management of diabetes mellitus in Africa: A systematic review and meta-analysis
Source: PLOS Digit Health. 2025 Apr 8;4(4):e0000776. doi: 10.1371/journal.pdig.0000776 (PMC11978007; doi:10.1371/journal.pdig.0000776)
Supplement: S1 Table — Table of all the articles yielded by the search strategy described above it, prior to title and abstract screening. It shows all the yields from the databases searched. (DOCX) [file pdig.0000776.s001.docx]

| S/N | ARTICLE | Major exclusion reasons |
| --- | --- | --- |
|  | An Adaptive, Algorithm-based Text Message Intervention to Promote Health Behavior Adherence in Type 2 Diabetes: Treatment Development and Proof-of-Concept Trial.  Celano C.M., Massey C., Long J., Kim S., Velasquez O., Healy B.C., Wexler D.J., Madva E.N., Huffman J.C.  Journal of Diabetes Science and Technology. (no pagination), 2021. Date of Publication: 2021.  [Article]  AN: 2014498666 | Not relevant to the main subject/ wrong population or geographical location/ duplicate or repetition |
|  | Digital messaging to support control for type 2 diabetes (StAR2D): a multicentre randomised controlled trial.  Farmer A., Bobrow K., Leon N., Williams N., Phiri E., Namadingo H., Cooper S., Prince J., Crampin A., Besada D., Daviaud E., Yu L.-M., N'goma J., Springer D., Pauly B., Tarassenko L., Norris S., Nyirenda M., Levitt N.  BMC public health. 21(1) (pp 1907), 2021. Date of Publication: 21 Oct 2021.  [Article]  AN: 636584010 | Included |
|  | Awareness and readiness to use telemonitoring to support diabetes care among care providers at teaching hospitals in Ethiopia: An institution-based cross-sectional study.  Seboka B.T., Yilma T.M., Birhanu A.Y.  BMJ Open. 11(10) (no pagination), 2021. Article Number: e050812. Date of Publication: 29 Oct 2021.  [Article]  AN: 636367375 | Not relevant to the main subject/ wrong population or geographical location/ duplicate or repetition |
|  | Preliminary phytochemical investigation and in-vitro antidiabetic activity of an ayurvedic formulation.  Gayatri S., Angel Seslin Monica V.  Bulletin of Pharmaceutical Sciences. Assiut. 43(2) (pp 135-139), 2020. Date of Publication: September 2020.  [Article]  AN: 2010477499 | Not relevant to the main subject/ wrong population or geographical location/ duplicate or repetition |
|  | Cardiovascular calcification in chronic kidney disease: Risk factors and effect of alpha-keto acid tablets.  Xiang N., Liao H., Zhai Z., Gong J.  Tropical Journal of Pharmaceutical Research. 20(11) (pp 2451-2457), 2021. Date of Publication: November 2021.  [Article]  AN: 2015901016 | Not relevant to the main subject/ wrong population or geographical location/ duplicate or repetition |
|  | Prediction of steady-state plasma concentrations of olanzapine in Chinese Han in patients based on a retrospective population pharmacokinetic model.  Wang X., Han Y., Zhou H., Cao B., Zhu M., Liu C., Gao C., McLeod H.L., Fang M.  Tropical Journal of Pharmaceutical Research. 20(11) (pp 2433-2441), 2021. Date of Publication: November 2021.  [Article]  AN: 2015901014 | Not relevant to the main subject/ wrong population or geographical location/ duplicate or repetition |
|  | Smartphone-Based mHealth and Internet of Things for Diabetes Control and Self-Management.  Mehbodniya A., Suresh Kumar A., Rane K.P., Bhatia K.K., Singh B.K.  Journal of Healthcare Engineering. 2021 (no pagination), 2021. Article Number: 2116647. Date of Publication: 2021.  [Article]  AN: 2015337727 | Not relevant to the main subject/ wrong population or geographical location/ duplicate or repetition |
|  | A Prospective, Non-randomized Feasibility and Preliminary Efficacy Study of a Telemedicine-Enabled Co-management Intervention for Adults With Type 2 Diabetes and Moderate Anxiety and/or Depression.  Magee M.F., Kaltman S.I., Mete M., Nassar C.M.  The science of diabetes self-management and care. 47(2) (pp 144-152), 2021. Date of Publication: 01 Apr 2021.  [Article]  AN: 636610399 | Not relevant to the main subject/ wrong population or geographical location/ duplicate or repetition |
|  | Effectiveness of using SOKARY mobile application on the compliance of patients with type II diabetes: A quasi-experimental study.  Yahia E.A., Bayoumi M.M.  Pakistan Journal of Medical and Health Sciences. 15(7) (pp 2001-2005), 2021. Date of Publication: July 2021.  [Article]  AN: 2015750454 | Not relevant to the main subject/ wrong population or geographical location/ duplicate or repetition |
|  | On the efficacy of behavior change techniques in mHealth for self-management of diabetes: A meta-analysis.  El-Gayar O., Ofori M., Nawar N.  Journal of Biomedical Informatics. 119 (no pagination), 2021. Article Number: 103839. Date of Publication: July 2021.  [Review]  AN: 2013173401 | Not relevant to the main subject/ wrong population or geographical location/ duplicate or repetition |
|  | The effect of lifestyle intervention on diabetes prevention by ethnicity: A systematic review of intervention characteristics using the tidier framework.  Chen M., Ukke G.G., Moran L.J., Sood S., Bennett C.J., Khomami M.B., Absetz P., Teede H., Harrison C.L., Lim S.  Nutrients. 13(11) (no pagination), 2021. Article Number: 4118. Date of Publication: November 2021.  [Article]  AN: 2014525252 | Not relevant to the main subject/ wrong population or geographical location/ duplicate or repetition |
|  | New-onset restless leg syndrome in a covid-19 patient: A case report with literature review.  Mohiuddin O., Khan A.A., Shah S.M.I., Malick M.D.Z., Memon S.F., Jalees S., Yasmin F.  Pan African Medical Journal. 38 (no pagination), 2021. Article Number: 318. Date of Publication: 2021.  [Article]  AN: 2007515185 | Not relevant to the main subject/ wrong population or geographical location/ duplicate or repetition |
|  | Management of diabetic ketoacidosis (DKA) in children during COVID-19 pandemic at a tertiary care center.  Tahri A., Messoudi N., Benyakhlef S., Yaagoubi L., Rouf S., Latrech H.  Pediatric Diabetes. Conference: 47th Annual Conference of the International Society for Pediatric and Adolescent Diabetes, ISPAD 2021. Virtual. 22(SUPPL 30) (pp 48), 2021. Date of Publication: October 2021.  [Conference Abstract]  AN: 636558788 | Not relevant to the main subject/ wrong population or geographical location/ duplicate or repetition |
|  | Association between dietary magnesium intake and glycemic markers in ghanaian women of reproductive age: A pilot cross-sectional study.  Bentil H.J., Abreu A.M., Adu-Afarwuah S., Rossi J.S., Tovar A., Oaks B.M.  Nutrients. 13(11) (no pagination), 2021. Article Number: 4141. Date of Publication: November 2021.  [Article]  AN: 2014569463 | Not relevant to the main subject/ wrong population or geographical location/ duplicate or repetition |
|  | Role of Mobile Technology in Multiple Sclerosis Patients' Health Education.  Ahmed M.B., Zakaria M., Zamzam D., Emara T.H.  QJM. Conference: 41st Annual International Ain Shams Medical Congress. Online. 114(SUPPL 1) (no pagination), 2021. Date of Publication: October 2021.  [Conference Abstract]  AN: 636515080 | Not relevant to the main subject/ wrong population or geographical location/ duplicate or repetition |
|  | Seizing the moment to rethink health systems.  Nimako K., Kruk M.E.  The Lancet Global Health. 9(12) (pp e1758-e1762), 2021. Date of Publication: December 2021.  [Review]  AN: 2015605336 | Not relevant to the main subject/ wrong population or geographical location/ duplicate or repetition |
|  | The Challenges of Managing Pediatric Diabetes and Other Endocrine Disorders During the COVID-19 Pandemic: Results From an International Cross-Sectional Electronic Survey.  Elbarbary N.S., dos Santos T.J., de Beaufort C., Wiltshire E., Pulungan A., Scaramuzza A.E.  Frontiers in Endocrinology. 12 (no pagination), 2021. Article Number: 735554. Date of Publication: 05 Nov 2021.  [Article]  AN: 636469764 | Not relevant to the main subject/ wrong population or geographical location/ duplicate or repetition |
|  | Technology-based nutrition interventions using the Mediterranean diet: a systematic review.  Benajiba N., Dodge E., Khaled M.B., Chavarria E.A., Sammartino C.J., Aboul-Enein B.H.  Nutrition reviews. (no pagination), 2021. Date of Publication: 19 Oct 2021.  [Article]  AN: 636479893 | Not relevant to the main subject/ wrong population or geographical location/ duplicate or repetition |
|  | The effect of morning versus evening administration of empagliflozin on its pharmacokinetics and pharmacodynamics characteristics in healthy adults: A two-way crossover, non-randomised trial.  ElDash R.M., Raslan M.A., Shaheen S.M., Sabri N.A.  F1000Research. 10 (no pagination), 2021. Article Number: 321. Date of Publication: 2021.  [Article]  AN: 635126009 | Not relevant to the main subject/ wrong population or geographical location/ duplicate or repetition |
|  | Designing an integrated, nurse-driven and home-based digital intervention to improve insulin management in under-resourced settings.  Ngassa Piotie P., Wood P., Webb E.M., Hugo J.F.M., Rheeder P.  Therapeutic Advances in Endocrinology and Metabolism. 12 (no pagination), 2021. Date of Publication: 2021.  [Article]  AN: 2014068378 | Not relevant to the main subject/ wrong population or geographical location/ duplicate or repetition |
|  | Implantable cardiac device infections prevalence: diagnostic and therapeutic implications.  Yahia H., Alazab A., Aly R., Elmaraghi S., Andraos A.  Open Access Macedonian Journal of Medical Sciences. 9(B) (pp 909-916), 2021. Date of Publication: 2021.  [Article]  AN: 2013693565 | Not relevant to the main subject/ wrong population or geographical location/ duplicate or repetition |
|  | Effects of a low carbohydrate intervention with nutritional ketosis on liver markers: A real-world experience.  Athinarayanan S., McKenzie A.L., Adams R.N., VanTieghem M., Roberts C.G.P., Phinney S.D.  Hepatology. Conference: 72nd Annual Meeting of the American Association for the Study of Liver Diseases, AASLD 2021. Virtual. 74(SUPPL 1) (pp 124A-125A), 2021. Date of Publication: October 2021.  [Conference Abstract]  AN: 636385209 | Not relevant to the main subject/ wrong population or geographical location/ duplicate or repetition |
|  | Pattern of abnormalities amongst chest X-rays of adults undergoing computer-assisted digital chest X-ray screening for tuberculosis in Peri-Urban Blantyre, Malawi: A cross-sectional study.  Twabi H.H., Semphere R., Mukoka M., Chiume L., Nzawa R., Feasey H.R.A., Lipenga T., MacPherson P., Corbett E.L., Nliwasa M.  Tropical Medicine and International Health. 26(11) (pp 1427-1437), 2021. Date of Publication: November 2021.  [Article]  AN: 2013333806 | Not relevant to the main subject/ wrong population or geographical location/ duplicate or repetition |
|  | Factors influencing the effective management of diabetes during humanitarian crises in low- and middle-income countries: a systematic review.  Song K., Lee A.  Public Health. 199 (pp 110-117), 2021. Date of Publication: October 2021.  [Review]  AN: 2014935802 | Not relevant to the main subject/ wrong population or geographical location/ duplicate or repetition |
|  | Low mortality of people living with diabetes mellitus diagnosed with COVID-19 and managed at a field hospital in Western Cape Province, South Africa.  van der Westhuizen J.-N., Hussey N., Zietsman M., Saldulker N., Manning K., Dave J.A., Bulajic B., Ras T.  South African Medical Journal. 111(10) (pp 961-967), 2021. Date of Publication: 2021.  [Article]  AN: 2015174860 | Not relevant to the main subject/ wrong population or geographical location/ duplicate or repetition |
|  | Development of multi-compartment 3d-printed tablets loaded with self-nanoemulsified formulations of various drugs: A new strategy for personalized medicine.  Ahmed T.A., Felimban R.I., Tayeb H.H., Rizg W.Y., Alnadwi F.H., Alotaibi H.A., Alhakamy N.A., Abd-Allah F.I., Mohamed G.A., Zidan A.S., El-Say K.M.  Pharmaceutics. 13(10) (no pagination), 2021. Article Number: 1733. Date of Publication: October 2021.  [Article]  AN: 2014301441 | Not relevant to the main subject/ wrong population or geographical location/ duplicate or repetition |
|  | Diabetes Mellitus and COVID-19: Review Article.  Nassar M., Daoud A., Nso N., Medina L., Ghernautan V., Bhangoo H., Nyein A., Mohamed M., Alqassieh A., Soliman K., Alfishawy M., Sachmechi I., Misra A.  Diabetes and Metabolic Syndrome: Clinical Research and Reviews. 15(6) (no pagination), 2021. Article Number: 102268. Date of Publication: 01 Nov 2021.  [Article]  AN: 2014690218 | Not relevant to the main subject/ wrong population or geographical location/ duplicate or repetition |
|  | Antimicrobial stewardship and economic evaluation of urinary tract infection management in primary health care in tunisia.  Essafi S., Letaief A.O., Phillips E., Vardanega V.  Family Medicine and Primary Care Review. 23(3) (pp 295-300), 2021. Date of Publication: 2021.  [Article]  AN: 2014114910 | Not relevant to the main subject/ wrong population or geographical location/ duplicate or repetition |
|  | Urinary proteomics combined with home blood pressure telemonitoring for health care reform trial: rational and protocol.  Thijs L., Asayama K., Maestre G.E., Hansen T.W., Buyse L., Wei D.-M., Melgarejo J.D., Brguljan-Hitij J., Cheng H.-M., de Souza F., Gilis-Malinowska N., Kawecka-Jaszcz K., Mels C., Mokwatsi G., Muxfeldt E.S., Narkiewicz K., Odili A.N., Rajzer M., Schutte A.E., Stolarz-Skrzypek K., Tsai Y.-W., Vanassche T., Vanholder R., Zhang Z.-Y., Verhamme P., Kruger R., Mischak H., Staessen J.A.  Blood pressure. 30(5) (pp 269-281), 2021. Date of Publication: 01 Oct 2021.  [Article]  AN: 636030583 | Not relevant to the main subject/ wrong population or geographical location/ duplicate or repetition |
|  | A systematic review of the effectiveness of person-centred interventions for serious physical illness in terms of self-report outcomes and costs.  Nkhoma K., Gwyther L., Farrant L., Petrus R., Petersen I., Giusti A., Harding R.  Palliative Medicine. Conference: 17th EAPC World Congress, EAPC 2021. Online. 35(1 SUPPL) (pp 164), 2021. Date of Publication: September 2021.  [Conference Abstract]  AN: 636233459 | Not relevant to the main subject/ wrong population or geographical location/ duplicate or repetition |
|  | Impact of covid-19 pandemic on chronic diseases care follow-up and current perspectives in low resource settings: A narrative review.  Fekadu G., Bekele F., Tolossa T., Fetensa G., Turi E., Getachew M., Abdisa E., Assefa L., Afeta M., Demisew W., Dugassa D., Diriba D.C., Labata B.G.  International Journal of Physiology, Pathophysiology and Pharmacology. 13(3) (pp 86-93), 2021. Article Number: IJPPP0133845. Date of Publication: 2021.  [Review]  AN: 2013336437 | Not relevant to the main subject/ wrong population or geographical location/ duplicate or repetition |
|  | Health service utilisation among African migrants in China: A nationwide cross-sectional study.  Xiong M.Z., Zhao P., Zou X., Hall B., Cao H., Wang C.  BMJ Open. 11(9) (no pagination), 2021. Article Number: e046746. Date of Publication: 16 Sep 2021.  [Article]  AN: 636009223 | Not relevant to the main subject/ wrong population or geographical location/ duplicate or repetition |
|  | AZITHROMYCIN-INDUCED TORSADES DE POINTES.  Mehra I., Isaac S., Kundu S., Chahal K.  Chest. Conference: CHEST 2021 Annual Meeting. Virtual, Online. 160(4 Supplement) (pp A155), 2021. Date of Publication: October 2021.  [Conference Abstract]  AN: 2014929701 | Not relevant to the main subject/ wrong population or geographical location/ duplicate or repetition |
|  | Process evaluation of a brief messaging intervention to improve diabetes treatment adherence in sub-Saharan Africa.  Leon N., Namadingo H., Cooper S., Bobrow K., Mwantisi C., Nyasulu M., Sicwebu N., Crampin A., Levitt N., Farmer A.  BMC public health. 21(1) (pp 1576), 2021. Date of Publication: 21 Aug 2021.  [Article]  AN: 635970395 | Not relevant to the main subject/ wrong population or geographical location/ duplicate or repetition |
|  | Effect of Statewide Lockdown in Response to COVID-19 Pandemic on Physical Activity Levels of Hemodialysis Patients.  Han M., Preciado P., Thwin O., Tao X., Tapia-Silva L.M., Fuentes L.R., Hakim M., Patel A., Tisdale L., Zhang H., Kotanko P.  Blood Purification. 50(4-5) (pp 602-609), 2021. Date of Publication: 01 Jul 2021.  [Article]  AN: 634670030 | Not relevant to the main subject/ wrong population or geographical location/ duplicate or repetition |
|  | Transvenous lead extraction in patients with persistent left superior vena cava.  Curnis A., Aboelhassan M., Cerini M., Salghetti F., Fabbricatore D., Maiolo V., Arabia G., Giacopelli D., Fouad D.A., Bontempi L.  Journal of Cardiovascular Electrophysiology. 32(5) (pp 1407-1410), 2021. Date of Publication: May 2021.  [Article]  AN: 2011181798 | Not relevant to the main subject/ wrong population or geographical location/ duplicate or repetition |
|  | Digital interventions self-management education for type 1 and 2 diabetes: A systematic review and meta-analysis.  Nkhoma D.E., Soko C.J., Bowrin P., Manga Y.B., Greenfield D., Househ M., Li(Jack) Y.-C., Iqbal U.  Computer Methods and Programs in Biomedicine. 210 (no pagination), 2021. Article Number: 106370. Date of Publication: October 2021.  [Article]  AN: 2014414946 | Not relevant to the main subject/ wrong population or geographical location/ duplicate or repetition |
|  | Perceptions of South African Nutrition Professionals of Avocados: Findings of the South Africa Avocado Growers' Association (SAAGA) Consumer Survey.  Piderit M.  South African Journal of Clinical Nutrition. Conference: International Congress of Dietetics, ICD 2021. Virtual. 34(3) (pp 171), 2021. Date of Publication: 2021.  [Conference Abstract]  AN: 636086712 | Not relevant to the main subject/ wrong population or geographical location/ duplicate or repetition |
|  | Insights into dynamic mechanism of ligand binding to peroxisome proliferator-activated receptor gamma toward potential pharmacological applications.  Miyamae Y.  Biological and Pharmaceutical Bulletin. 44(9) (pp 1185-1195), 2021. Date of Publication: September 2021.  [Review]  AN: 2014362706 | Not relevant to the main subject/ wrong population or geographical location/ duplicate or repetition |
|  | Diabetes during the fasting month of Ramadan: Is telemedicine as efficient as conventional follow-up? results from a moroccan comparative study.  Motaib I., Elamari S., Khalis M., Oudghiri M.D., Laidi S., Chadli A.  Diabetes, Metabolic Syndrome and Obesity: Targets and Therapy. 14 (pp 3959-3964), 2021. Date of Publication: 2021.  [Article]  AN: 2013731922 | Not relevant to the main subject/ wrong population or geographical location/ duplicate or repetition |
|  | Managing metabolic issues: Evaluation of a short-term comparative implementation project introducing a dietitian to an HIV service.  Yan H., Hunter A., Burns F., Madge S., Barber T.  HIV Medicine. Conference: 5th Conference of the British HIV Association, BHIVA with the British Association for Sexual Health and HIV, BASHH. Virtual. 22(SUPPL 2) (pp 53-54), 2021. Date of Publication: August 2021.  [Conference Abstract]  AN: 635945596 | Not relevant to the main subject/ wrong population or geographical location/ duplicate or repetition |
|  | Glycemic control among children and adolescents with type 1 diabetes during COVID-19 pandemic in Egypt: a pilot study.  Elhenawy Y.I., Eltonbary K.Y.  International Journal of Diabetes in Developing Countries. 41(3) (pp 389-395), 2021. Date of Publication: July 2021.  [Article]  AN: 2013111390 | Not relevant to the main subject/ wrong population or geographical location/ duplicate or repetition |
|  | Development of a Mobile Application Platform for Self-Management of Obesity Using Artificial Intelligence Techniques.  Sefa-Yeboah S.M., Osei Annor K., Koomson V.J., Saalia F.K., Steiner-Asiedu M., Mills G.A.  International Journal of Telemedicine and Applications. 2021 (no pagination), 2021. Article Number: 6624057. Date of Publication: 2021.  [Article]  AN: 2014506254 | Not relevant to the main subject/ wrong population or geographical location/ duplicate or repetition |
|  | Comparing two telehealth interventions for patients with clinic-refractory type 2 diabetes: A randomized controlled trial.  Crowley M.J., Tarkington P.E., Bosworth H.B., Maciejewski M.L., Steinhauser K., Jeffreys A.S., Coffman C., Smith V., Danus S., Jeter D.H., Strawbridge E., Wilmot T.C., Tisdale G.A., Marcano T., Overby D.L., Durkee M.A., Bullard S., Dar M., Mundy A., Szabo S.T., Desai S., Kobe E.A., Elliott N.M., Edelman D.  Diabetes. Conference: 81st Scientific Sessions of the American Diabetes Association, ADA 2021. Virtual. 70(SUPPL 1) (no pagination), 2021. Date of Publication: June 2021.  [Conference Abstract]  AN: 635917261 | Not relevant to the main subject/ wrong population or geographical location/ duplicate or repetition |
|  | Predictors of engagement in clinical care: Coyot1 to California.  Garcia J.J.F., Reid M.W., Pyatak E., Fox D.S., Fogel J.L., Salcedo-Rodriguez E., Bisno D.I., Miller D., Mittal A., Raymond J.  Diabetes. Conference: 81st Scientific Sessions of the American Diabetes Association, ADA 2021. Virtual. 70(SUPPL 1) (no pagination), 2021. Date of Publication: June 2021.  [Conference Abstract]  AN: 635917250 | Not relevant to the main subject/ wrong population or geographical location/ duplicate or repetition |
|  | The role of continuous glucose monitoring, diabetes smartphone applications, and self-care behavior in glycemic control: Results of a multi-national online survey.  Kebede M.M., Schuett C., Pischke C.R.  Journal of Clinical Medicine. 8(1) (no pagination), 2019. Article Number: 109. Date of Publication: January 2019.  [Article]  AN: 2002417634 | Not relevant to the main subject/ wrong population or geographical location/ duplicate or repetition |
|  | Pandemics and burden of stroke and epilepsy in sub-saharan africa: Experience from a longstanding health programme.  Leone M., Ciccacci F., Orlando S., Petrolati S., Guidotti G., Majid N.A., Tolno V.T., Sagno J., Thole D., Corsi F.M., Bartolo M., Marazzi M.C.  International Journal of Environmental Research and Public Health. 18(5) (pp 1-19), 2021. Article Number: 2766. Date of Publication: 01 Mar 2021.  [Review]  AN: 2006165519 | Not relevant to the main subject/ wrong population or geographical location/ duplicate or repetition |
|  | Effect of Layperson-Delivered, Empathy-Focused Program of Telephone Calls on Loneliness, Depression, and Anxiety among Adults during the COVID-19 Pandemic: A Randomized Clinical Trial.  Kahlon M.K., Aksan N., Aubrey R., Clark N., Cowley-Morillo M., Jacobs E.A., Mundhenk R., Sebastian K.R., Tomlinson S.  JAMA Psychiatry. 78(6) (pp 616-622), 2021. Date of Publication: June 2021.  [Article]  AN: 634291443 | Not relevant to the main subject/ wrong population or geographical location/ duplicate or repetition |
|  | Preventive infection control in cardiac device implantation. Pravention von Infektionen bei der Implantation kardialer Devices <Pravention von Infektionen bei der Implantation kardialer Devices.>  Khalifa M.M.M., Kolta M.L., Tawfik M., Khaled S., Fakhry E.E.  Herzschrittmachertherapie und Elektrophysiologie. 32(1) (pp 54-61), 2021. Date of Publication: March 2021.  [Article]  AN: 2007087444 | Not relevant to the main subject/ wrong population or geographical location/ duplicate or repetition |
|  | Pilot Mobile Phone Intervention in Promoting Type 2 Diabetes Management in an Urban Area in Ghana: A Randomized Controlled Trial.  Asante E., Bam V., Diji A.K.-A., Lomotey A.Y., Owusu Boateng A., Sarfo-Kantanka O., Oparebea Ansah E., Adjei D.  The Diabetes educator. 46(5) (pp 455-464), 2020. Date of Publication: 01 Oct 2020.  [Article]  AN: 633082075 | Included |
|  | Interventions to improve daily medication use among adolescents and young adults: What can we learn for youth pre-exposure prophylaxis services?.  Velloza J., Kapogiannis B., Bekker L.-G., Celum C., Hosek S., Delany-Moretlwe S., Baggaley R., Dalal S.  AIDS. 35(3) (pp 463-475), 2021. Date of Publication: 01 Mar 2021.  [Article]  AN: 635378946 | Not relevant to the main subject/ wrong population or geographical location/ duplicate or repetition |
|  | Self-measured blood pressure monitoring (SBPM) during the COVID-19 pandemic.  Girma B., Curtis N.C.  Journal of General Internal Medicine. Conference: 2021 Annual Meeting of the Society of General Internal Medicine, SGIM 2021. Virtual. 36(SUPPL 1) (pp S138), 2021. Date of Publication: 2021.  [Conference Abstract]  AN: 635796663 | Not relevant to the main subject/ wrong population or geographical location/ duplicate or repetition |
|  | A qualitative evaluation of health coaching, text messaging, and educational interventions to improve self-care among african american adults with uncontrolled diabetes.  Gutierrez M.L., Graff J.C., Lopez L., Tate R., Williams S., Scott M.D., Pope Z., Fairrow-Davis P.P., Bailey J.E.  Journal of General Internal Medicine. Conference: 2021 Annual Meeting of the Society of General Internal Medicine, SGIM 2021. Virtual. 36(SUPPL 1) (pp S55), 2021. Date of Publication: 2021.  [Conference Abstract]  AN: 635796631 | Not relevant to the main subject/ wrong population or geographical location/ duplicate or repetition |
|  | Association between depressive symptoms and text message sentiment in patients with type 2 diabetes receiving health coach support.  Nava-Frenier M., Harris B., Rodriguez A., Stevenson C., Biggers A., Perez R., Sharp L.K., Gerber B.  Journal of General Internal Medicine. Conference: 2021 Annual Meeting of the Society of General Internal Medicine, SGIM 2021. Virtual. 36(SUPPL 1) (pp S2-S3), 2021. Date of Publication: 2021.  [Conference Abstract]  AN: 635796433 | Not relevant to the main subject/ wrong population or geographical location/ duplicate or repetition |
|  | Engaging African American patients with diabetes to form a patient advisory council for the management of diabetes in everyday life.  Gutierrez M.L., Bailey J.E., Lopez L., Tate R.  Journal of General Internal Medicine. Conference: 2021 Annual Meeting of the Society of General Internal Medicine, SGIM 2021. Virtual. 36(SUPPL 1) (pp S64), 2021. Date of Publication: 2021.  [Conference Abstract]  AN: 635796400 | Not relevant to the main subject/ wrong population or geographical location/ duplicate or repetition |
|  | Nonspecific presentation of hydralazine-induced ANCA-associated vasculitis.  Kowalski A., Sigler L.E., Ahmed H., Smith D.  Journal of General Internal Medicine. Conference: 2021 Annual Meeting of the Society of General Internal Medicine, SGIM 2021. Virtual. 36(SUPPL 1) (pp S251), 2021. Date of Publication: 2021.  [Conference Abstract]  AN: 635795772 | Not relevant to the main subject/ wrong population or geographical location/ duplicate or repetition |
|  | Development of a mobile phone based ophthalmoscope for telemedicine.  Blanckenberg M., Worst C., Scheffer C.  Conference proceedings : ... Annual International Conference of the IEEE Engineering in Medicine and Biology Society. IEEE Engineering in Medicine and Biology Society. Conference. (pp 5236-5239), 2011. Date of Publication: 2011.  [Article]  AN: 366427465 | Not relevant to the main subject/ wrong population or geographical location/ duplicate or repetition |
|  | Educational films for improving screening and self-management of gestational diabetes in India and Uganda (GUIDES): study protocol for a cluster-randomised controlled trial.  Oakley L.L., R D., Namara A., Sahu B., Nadal I.P., Ana Y., Coombe H., Oteng-Ntim E., Seeley J., Nyirenda M., Babu G., Kinra S.  Trials. 22(1) (no pagination), 2021. Article Number: 501. Date of Publication: December 2021.  [Article]  AN: 2013307605 | Not relevant to the main subject/ wrong population or geographical location/ duplicate or repetition |
|  | TH9 cells and their associated cytokines: the probable players in systemic lupus erythematosus (SLE) and type 1 diabetes mellitus (T1DM).  Mohannad N., Moaaz M., Mohamed Shehata R.  Annals of the Rheumatic Diseases. Conference: European Congress of Rheumatology, EULAR 2021. Virtual. 80(SUPPL 1) (pp 1197-1198), 2021. Date of Publication: June 2021.  [Conference Abstract]  AN: 635709676 | Not relevant to the main subject/ wrong population or geographical location/ duplicate or repetition |
|  | Canagliflozin stability study and ecofriendly chromatographic determination of its degradation product: A comparative study.  Emam A.A.  Journal of Separation Science. 41(4) (pp 822-830), 2018. Date of Publication: February 2018.  [Article]  AN: 620830204 | Not relevant to the main subject/ wrong population or geographical location/ duplicate or repetition |
|  | Web-based health information seeking among African American and hispanic men living with chronic conditions: Cross-sectional survey study.  Sherman L.D., Goidel K., Bergeron C.D., Smith M.L.  Journal of Medical Internet Research. 23(7) (no pagination), 2021. Article Number: e26180. Date of Publication: July 2021.  [Article]  AN: 2013625982 | Not relevant to the main subject/ wrong population or geographical location/ duplicate or repetition |
|  | Detecting Glaucoma in Rural Kenya: Results from a Teleglaucoma Pilot Project in Nyamira, Kenya.  Marco S.A., Amin S., Virani A., Rudnisky C.J., Ishani S., Kiage D., Damji K.F.  Journal of Glaucoma. 30(3) (pp e99-e104), 2021. Date of Publication: March 2021.  [Article]  AN: 634356494 | Not relevant to the main subject/ wrong population or geographical location/ duplicate or repetition |
|  | Feasibility of Advanced Practice Nurse - Led Telehealth Service in Patients with Myeloproliferative Neoplasm in the Community: A Singapore Single-Centre Report.  Lim C.C., Chen X., Lee Y.M., Teo W.Z., Tung M.L., Chng W.-J., Ooi M.  Blood. Conference: 62nd ASH Annual Meeting. Virtual, Online. 136(Supplement 1) (pp 18-19), 2020. Date of Publication: 05 Nov 2020.  [Conference Abstract]  AN: 2013848857 | Not relevant to the main subject/ wrong population or geographical location/ duplicate or repetition |
|  | Effectiveness of a structured nutrition education course for caregivers of children and adolescents with type 1 diabetes in improving glycemic and dietary outcomes: A cluster-randomized controlled trial protocol.  Ndahura N.B., Munga J., Kimiywe J., Mupere E.  Open Access Journal of Clinical Trials. 13 (no pagination), 2021. Date of Publication: 2021.  [Article]  AN: 2007175850 | Not relevant to the main subject/ wrong population or geographical location/ duplicate or repetition |
|  | A glassy carbon electrode for the determination of linagliptin, an antidiabet-ic drug in pure form, tablets and some biological fluids by adsorptive stripping voltammetry.  Gahlan A.A., Haredy A.M., Derayea S.M., Omar M.A., Saleh G.A.  Current Pharmaceutical Design. 27(20) (pp 2415-2424), 2021. Date of Publication: June 2021.  [Article]  AN: 2013175484 | Not relevant to the main subject/ wrong population or geographical location/ duplicate or repetition |
|  | Control of blood pressure and cardiovascular risk in Moroccan patients with newly diagnosed hypertension: A 3-month observational study in primary care.  Alami M., El Hattaoui M., Seqat M., Sadik J., Aouad A., Benghanem Gharbi M.  Therapeutic Advances in Cardiovascular Disease. 11(2) (pp 49-56), 2017. Date of Publication: February 2017.  [Article]  AN: 614319672 | Not relevant to the main subject/ wrong population or geographical location/ duplicate or repetition |
|  | Convergence of infectious and non-communicable disease epidemics in rural South Africa: a cross-sectional, population-based multimorbidity study.  Olivier S., Gunda R., Koole O., Surujdeen A., Gareta D., Munatsi D., Dreyer J., Nxumalo S., Ording-Jespersen G., Khan K., Moodley S., Shen Y.-J., Khoza T., Mhlongo N., Nyamande K., Cuadros D., Tanser F., Herbst K., Seeley J., Ndung'u T., Pillay D., Wong E.B., Modise T.H., Smit T.K., Mpofana I.B., Sikhosana Z.E.L., Bucibo S., Baisley K.J., Grant A.D., Hanekom W.A., Siedner M.J., Suleman M., Kalideen J., Jackpersad R., Moropane K., Mfolo B., Malomane K., Khumalo H., Buthelezi N., Mbonambi N., Ngubane H., Simelane T., Buthelezi K., Ntuli S., Zondi N., Nene S., Ndlovu B., Ntimbane T., Mbuyisa M., Mkhize X., Sibiya M., Ntombela N., Dlamini M., Chonco H., Dlamini H., Mlambo D., Mzimela N., Buthelezi Z., Mthembu Z., Bhengu T., Mtehmbu S., Mthethwa P., Mbatha Z., Mthembu W.P., Mkhwanazi A., Sikhali M., Mkhwanazi P., Mkhwanazi N., Myeni R., Mfeka F., Gumede H., Mfeka N., Zungu A., Mfekayi N., Zulu S., Buthelezi M., Dube M., Matthews P., Dlamini S., Kambonde H., Mthembu L., Mchunu S., Gumbi S., Madolo T., Nkosi T., Mkhwanazi S., Steto M., Mhlongo S., Vellem V., Tshivase P., Kwinda J., Magwaza B., Nsibande S., Mthombeni S., Mthembu S.C., Rapulana A., Cousins J., Zondi T., Padayachi N., Mabetlela F., Ntshangase S., Luthuli N., Ngcobo S., Brien K., Ndlela S., Ngema N., Ntshakala N., Singh A., Singh R., Pillay L., Chetty K., Govender A., Ramkalawon P., Mabaso N., Perumal K., Makhari S., Khuluse N., Zitha N., Khati H., Mofokeng M., Majozi N., Gqaleni N., Keal H., Ngcobo P., Criticos C., Zondo R., Kalyan D., Mavimbela C., Ramnanan A., Harilall S.  The Lancet Global Health. 9(7) (pp e967-e976), 2021. Date of Publication: July 2021.  [Article]  AN: 2013049735 | Not relevant to the main subject/ wrong population or geographical location/ duplicate or repetition |
|  | Physician assessment of patients' COVID-19 risk and impact on lasmiditan, ubrogepant, or rimegepant prescription decisions: Analysis from a cross-sectional, retrospective US patient chart audit.  Ju S., Cooper V., Gottschalk C.H., Schobel V.R.  Headache. Conference: 63rd American Headache Society Annual Scientific Meeting, AHS 2021. Virtual. 61(SUPPL 1) (pp 137), 2021. Date of Publication: June 2021.  [Conference Abstract]  AN: 635482256 | Not relevant to the main subject/ wrong population or geographical location/ duplicate or repetition |
|  | Evaluation of the anti-osteoporotic effects of metformin and sitagliptin in postmenopausal diabetic women.  Hegazy S.K.  Journal of Bone and Mineral Metabolism. 33(2) (pp 207-212), 2015. Date of Publication: March 2015.  [Article]  AN: 53057171 | Not relevant to the main subject/ wrong population or geographical location/ duplicate or repetition |
|  | Changing CHANGE: Adaptations of an evidence-based telehealth cardiovascular disease risk reduction intervention.  Zullig L.L., McCant F., Silberberg M., Johnson F., Granger B.B., Bosworth H.B.  Translational Behavioral Medicine. 8(2) (pp 225-232), 2018. Date of Publication: 01 Mar 2018.  [Article]  AN: 621773906 | Not relevant to the main subject/ wrong population or geographical location/ duplicate or repetition |
|  | Determining the potential of mobilephone-based health interventions in Kumasi, Ghana.  Stephani V., Opoku D., Otupiri E.  Ghana medical journal. 54(2) (pp 88-92), 2020. Date of Publication: 01 Jun 2020.  [Article]  AN: 634198257 | Not relevant to the main subject/ wrong population or geographical location/ duplicate or repetition |
|  | Refining a traditional urban-rural classification approach to better assess heterogeneity of treatment effects in patient-centered outcomes research.  Surbhi S., Tolley E.A., Cossman R.E., Dashputre A.A., Bailey J.E.  MethodsX. 8 (no pagination), 2021. Article Number: 101299. Date of Publication: January 2021.  [Article]  AN: 2011453413 | Not relevant to the main subject/ wrong population or geographical location/ duplicate or repetition |
|  | The feasibility and acceptability of a web-based physical activity for the heart (PATH) intervention designed to reduce the risk of heart disease among inactive African Americans: Protocol for a pilot randomized controlled trial.  Kariuki J.K., Gibbs B.B., Erickson K.I., Kriska A., Sereika S., Ogutu D., Milton H., Wagner L.V., Rao N., Peralta R., Bobb J., Bermudez A., Hirshfield S., Goetze T., Burke L.E.  Contemporary Clinical Trials. 104 (no pagination), 2021. Article Number: 106380. Date of Publication: May 2021.  [Article]  AN: 2011577848 | Not relevant to the main subject/ wrong population or geographical location/ duplicate or repetition |
|  | Telemedicine in the Western Cape Department of Health during the first peak of the COVID-19 pandemic: Leveraging data to save lives by activating a telemedicine response.  David N.J., Brey Z., Ismail M.  African journal of primary health care & family medicine. 13(1) (pp e1-e4), 2021. Date of Publication: 20 May 2021.  [Article]  AN: 635255112 | Not relevant to the main subject/ wrong population or geographical location/ duplicate or repetition |
|  | Health system interventions for adults with type 2 diabetes in low- And middle-income countries: A systematic review and metaanalysis.  Flood D., Hane J., Dunn M., Brown S.J., Wagenaar B.H., Rogers E.A., Heisler M., Rohloff P., Chopra V.  PLoS Medicine. 17(11) (no pagination), 2020. Article Number: e1003434. Date of Publication: 12 Nov 2020.  [Review]  AN: 2010216005 | Not relevant to the main subject/ wrong population or geographical location/ duplicate or repetition |
|  | Is the lack of smartphone data skewing wealth indices in low-income settings?.  Poirier M.J.P., Barnighausen T., Harling G., Sie A., Grepin K.A.  Population Health Metrics. 19(1) (no pagination), 2021. Article Number: 4. Date of Publication: December 2021.  [Article]  AN: 2010317956 | Not relevant to the main subject/ wrong population or geographical location/ duplicate or repetition |
|  | Mobile apps for self-management in pregnancy: a systematic review.  Iyawa G.E., Dansharif A.R., Khan A.  Health and Technology. 11(2) (pp 283-294), 2021. Date of Publication: March 2021.  [Review]  AN: 2010374312 | Not relevant to the main subject/ wrong population or geographical location/ duplicate or repetition |
|  | The global impact of the COVID-19 pandemic on the management and course of chronic urticaria.  Kocaturk E., Salman A., Cherrez-Ojeda I., Criado P.R., Peter J., Comert-Ozer E., Abuzakouk M., Agondi R.C., Al-Ahmad M., Altrichter S., Arnaout R., Arruda L.K., Asero R., Bauer A., Ben-Shoshan M., Bernstein J.A., Bizjak M., Boccon-Gibod I., Bonnekoh H., Bouillet L., Brzoza Z., Busse P., Campos R.A., Carne E., Conlon N., Criado R.F., de Souza Lima E.M., Demir S., Dissemond J., Dogan Gunaydin S., Dorofeeva I., Ensina L.F., Ertas R., Ferrucci S.M., Figueras-Nart I., Fomina D., Franken S.M., Fukunaga A., Gimenez-Arnau A.M., Godse K., Goncalo M., Gotua M., Grattan C., Guillet C., Inomata N., Jakob T., Karakaya G., Kasperska-Zajac A., Katelaris C.H., Kosnik M., Krasowska D., Kulthanan K., Kumaran M.S., Lang C., Larco-Sousa J.I., Lazaridou E., Leslie T.A., Lippert U., llosa O.C., Makris M., Marsland A., Medina I.V., Meshkova R., Palitot E.B., Parisi C.A.S., Pickert J., Ramon G.D., Rodriguez-Gonzalez M., Rosario N., Rudenko M., Rutkowski K., Sanchez J., Schliemann S., Sekerel B.E., Serpa F.S., Serra-Baldrich E., Song Z., Soria A., Staevska M., Staubach P., Tagka A., Takahagi S., Thomsen S.F., Treudler R., Vadasz Z., Valle S.O.R., Van Doorn M.B.A., Vestergaard C., Wagner N., Wang D., Wang L., Wedi B., Xepapadaki P., Yucel E., Zalewska-Janowska A., Zhao Z., Zuberbier T., Maurer M.  Allergy: European Journal of Allergy and Clinical Immunology. 76(3) (pp 816-830), 2021. Date of Publication: March 2021.  [Article]  AN: 2007692510 | Not relevant to the main subject/ wrong population or geographical location/ duplicate or repetition |
|  | Results of a Culturally Tailored Smartphone-Delivered Physical Activity Intervention Among Midlife African American Women: Feasibility Trial.  Joseph R.P., Ainsworth B.E., Hollingshead K., Todd M., Keller C.  JMIR mHealth and uHealth. 9(4) (pp e27383), 2021. Date of Publication: 22 Apr 2021.  [Article]  AN: 634866287 | Not relevant to the main subject/ wrong population or geographical location/ duplicate or repetition |
|  | Successful insulin glargine treatment in two pet guinea pigs with suspected type 1 diabetes mellitus.  Kreilmeier-Berger T., Zeugswetter F.K., Blohm K.-O., Schwendenwein I., Baszler E., Ploderer B., Burgener I.A., Kunzel F.  Animals. 11(4) (no pagination), 2021. Article Number: 1025. Date of Publication: April 2021.  [Article]  AN: 2006887282 | Not relevant to the main subject/ wrong population or geographical location/ duplicate or repetition |
|  | Baseline incision characteristics and early scar maturation indices following cardiac device implantation.  Mehta N.K., Morgaenko K., Haines D., Rojas-Pena E., Heard B., Malhotra R., Darby A., Mangrum J.M., Mason P., Campbell C., Bilchick K.  Journal of Arrhythmia. 37(2) (pp 400-406), 2021. Date of Publication: April 2021.  [Article]  AN: 2010196207 | Not relevant to the main subject/ wrong population or geographical location/ duplicate or repetition |
|  | Fast track pathway: A specific tool for improving early referral of patients with diabetic foot ulcers.  Marco M.  Journal of Wound Care. Conference: World Union of Wound Healing Societies, WUWHS 2020. Abu Dhabi . 29(SUPPL 7B) (pp 234), 2020. Date of Publication: 2020.  [Conference Abstract]  AN: 635069603 | Not relevant to the main subject/ wrong population or geographical location/ duplicate or repetition |
|  | Management of patients with diabetes and obesity in the COVID-19 era: Experiences and learnings from South and East Europe, the Middle East, and Africa.  Giorgino F., Bhana S., Czupryniak L., Dagdelen S., Galstyan G.R., Janez A., Lalic N., Nouri N., Rahelic D., Stoian A.P., Raz I.  Diabetes Research and Clinical Practice. 172 (no pagination), 2021. Article Number: 108617. Date of Publication: February 2021.  [Review]  AN: 2010667775 | Not relevant to the main subject/ wrong population or geographical location/ duplicate or repetition |
|  | The necessity of implementing telemedicine systems in the Islamic republic of Iran.  Keshvardoost S., Dehnavieh R., Bahaadinibeigy K.  Eastern Mediterranean Health Journal. 27(2) (pp 113-115), 2021. Date of Publication: 2021.  [Article]  AN: 2006871459  PMID  33665794 [<http://www.ncbi.nlm.nih.gov/pubmed/?term=33665794>]  Status | Not relevant to the main subject/ wrong population or geographical location/ duplicate or repetition |
|  | Factors influencing healthcare providers' attitude and willingness to use information technology in diabetes management.  Seboka B.T., Yilma T.M., Birhanu A.Y.  BMC medical informatics and decision making. 21(1) (pp 24), 2021. Date of Publication: 21 Jan 2021.  [Article]  AN: 634083219 | Not relevant to the main subject/ wrong population or geographical location/ duplicate or repetition |
|  | Feasibility of text message sleep assessment in african american and latino patients with type 2 diabetes.  Biggers A., Henkins J., Barton I., Hubbard C., Perez R., Sharp L.K., Gerber B.S.  Journal of Clinical Sleep Medicine. 17(1) (pp 69-78), 2021. Date of Publication: 2021.  [Article]  AN: 2011053224 | Not relevant to the main subject/ wrong population or geographical location/ duplicate or repetition |
|  | Implementation and adaptation of physician-pharmacist collaborative management hypertension service for geriatric patients during the covid-19 pandemic.  Zhang A., Madden W., Garcia F., Levine S., Thompson K., Kostas T.  Journal of the American Geriatrics Society. Conference: American Geriatrics Society Annual Meeting, AGS 2021. Virtual. 69(SUPPL 1) (pp S243), 2021. Date of Publication: April 2021.  [Conference Abstract]  AN: 634826954 | Not relevant to the main subject/ wrong population or geographical location/ duplicate or repetition |
|  | Impact of DSMES app interventions on medication adherence in type 2 diabetes mellitus: systematic review and meta-analysis.  Enricho Nkhoma D., Jenya Soko C., Joseph Banda K., Greenfield D., Li Y.-C.J., Iqbal U.  BMJ health & care informatics. 28(1) (no pagination), 2021. Date of Publication: 01 Apr 2021.  [Review]  AN: 634810489 | Not relevant to the main subject/ wrong population or geographical location/ duplicate or repetition |
|  | Association between visual acuity impairment and social determinants of health in Tunisian diabetic adults, 2016.  Cherif I., Rejaibi S., Mansour N.B., Zoghlami N., Saidi O., Skhiri A., Ben Slama F., Hsairi M., Romthane H.B., Turki Z., Aounallah-Skhiri H.  Revista Argentina de Endocrinologia y Metabolismo. Conference: 19th International Congress of Endocrinology, 4th latin American Congress of Endocrinology, CONLAEN and 13th Congress of the Argrntine Federation of Endocrinology Societies, FASEN. Virtual. 58(SUPPL 1) (pp 114), 2021. Date of Publication: 2021.  [Conference Abstract]  AN: 634781601 | Not relevant to the main subject/ wrong population or geographical location/ duplicate or repetition |
|  | Safety and tolerability of mass diethylcarbamazine and albendazole administration for the elimination of lymphatic filariasis in Kenya: An active surveillance study.  Khaemba C., Barry A., Omondi W.P., Bota K., Matendechero S., Wandera C., Siyoi F., Kirui E., Oluka M., Nambwa P., Gurumurthy P., Njenga S.M., Guantai A., Aklillu E.  Pharmaceuticals. 14(3) (no pagination), 2021. Article Number: 264. Date of Publication: March 2021.  [Article]  AN: 2006837674 | Not relevant to the main subject/ wrong population or geographical location/ duplicate or repetition |
|  | COVID-19 associated with diabetes and other noncommunicable diseases led to a global health crisis.  Barone M.T.U., Ngongo B., Harnik S.B., Oliveira L.X.D., Vegh D., de Luca P.V., Pedrosa H.C., Giraudo F., Cardona-Hernandez R., Chaudhury N., Menna-Barreto L.  Diabetes Research and Clinical Practice. 171 (no pagination), 2021. Article Number: 108587. Date of Publication: January 2021.  [Review]  AN: 2010405128 | Not relevant to the main subject/ wrong population or geographical location/ duplicate or repetition |
|  | Technology-assisted cognitive-behavioral therapy intervention for end-stage renal disease.  Jakubowski K.P., Jhamb M., Yabes J., Gujral S., Oberlin L.E., Bender F.H., Steel J.L.  Translational Behavioral Medicine. 10(3) (pp 657-663), 2020. Date of Publication: 01 Jun 2020.  [Article]  AN: 2010108609 | Not relevant to the main subject/ wrong population or geographical location/ duplicate or repetition |
|  | Multinational Association of Supportive Care in Cancer (MASCC) 2020 clinical practice recommendations for the management of immune checkpoint inhibitor endocrinopathies and the role of advanced practice providers in the management of immune-mediated toxicities.  Cooksley T., Girotra M., Ginex P., Gordon R.A., Anderson R., Blidner A., Choi J., Dougan M., Glezerman I., Gupta D., Johnson D., Shannon V.R., Suarez-Almazor M., Rapoport B.L.  Supportive Care in Cancer. 28(12) (pp 6175-6181), 2020. Date of Publication: December 2020.  [Article]  AN: 2005992717 | Not relevant to the main subject/ wrong population or geographical location/ duplicate or repetition |
|  | Rams Have Heart, a Mobile App Tracking Activity and Fruit and Vegetable Consumption to Support the Cardiovascular Health of College Students: Development and Usability Study.  Krzyzanowski M.C., Kizakevich P.N., Duren-Winfield V., Eckhoff R., Hampton J., Blackman Carr L.T., McCauley G., Roberson K.B., Onsomu E.O., Williams J., Price A.A.  JMIR mHealth and uHealth. 8(8) (pp e15156), 2020. Date of Publication: 05 Aug 2020.  [Article]  AN: 632545065 | Not relevant to the main subject/ wrong population or geographical location/ duplicate or repetition |
|  | Effect of a nurse-led lifestyle choice and coaching intervention on systolic blood pressure among type 2 diabetic patients with a high atherosclerotic cardiovascular risk: study protocol for a cluster-randomized trial.  Lumu W., Kibirige D., Wesonga R., Bahendeka S.  Trials. 22(1) (no pagination), 2021. Article Number: 133. Date of Publication: December 2021.  [Article]  AN: 2010468291 | Not relevant to the main subject/ wrong population or geographical location/ duplicate or repetition |
|  | Development and Evaluation of a Tailored Mobile Health Intervention to Improve Medication Adherence in Black Patients With Uncontrolled Hypertension and Type 2 Diabetes: Pilot Randomized Feasibility Trial.  Schoenthaler A., Leon M., Butler M., Steinhaeuser K., Wardzinski W.  JMIR mHealth and uHealth. 8(9) (pp e17135), 2020. Date of Publication: 23 Sep 2020.  [Article]  AN: 632958842 | Not relevant to the main subject/ wrong population or geographical location/ duplicate or repetition |
|  | Clinical pharmacokinetic evaluation of optimized liquisolid tablets as a potential therapy for male sexual dysfunction.  Alotaibi F.O., Alhakamy N.A., Omar A.M., El-Say K.M.  Pharmaceutics. 12(12) (pp 1-23), 2020. Article Number: 1187. Date of Publication: December 2020.  [Article]  AN: 2005574766 | Not relevant to the main subject/ wrong population or geographical location/ duplicate or repetition |
|  | Abstracts from the NIH Office of Research on Women's Health 2020 Annual BIRCWH Meeting: Building Interdisciplinary Research Careers in Women's Health.  Anonymous  Journal of Women's Health. Conference: 2020 Annual BIRCWH Meeting: Building Interdisciplinary Research Careers in Women's Health. Virtual. 29(12) (no pagination), 2020. Date of Publication: 2020.  [Conference Review]  AN: 634418791 | Not relevant to the main subject/ wrong population or geographical location/ duplicate or repetition |
|  | Management of type 2 diabetes in clinical practices in sub-Saharan Africa: Results of the AMAR-AFO study in Senegal and Ivory Cost. Prise en charge du diabete de type 2 en pratique medicale courante en Afrique sub-saharienne: resultats de l'etude AMAR-AFO au Senegal et en Cote-d'Ivoire <Prise en charge du diabete de type 2 en pratique medicale courante en Afrique sub-saharienne: resultats de l'etude AMAR-AFO au Senegal et en Cote-d'Ivoire.>  Diop S.-N., Wade A., Lokrou A., Diedhiou D., Adoueni V.-K.  Medecine des Maladies Metaboliques. 7(4) (pp 363-367), 2013. Date of Publication: September 2013.  [Article]  AN: 372236822 | Not relevant to the main subject/ wrong population or geographical location/ duplicate or repetition |
|  | An atypical presentation of amyloidosis in the diabetic patient: The importance of recognizing anchoring bias.  Lantz J., Delaney S.  Journal of General Internal Medicine. Conference: Annual Meeting of the Society of General Internal Medicine, SGIM 2020. Birmingham, AL United States. 35(SUPPL 1) (pp S377-S378), 2020. Date of Publication: July 2020.  [Conference Abstract]  AN: 633957783 | Not relevant to the main subject/ wrong population or geographical location/ duplicate or repetition |
|  | Patient perspectives regarding the social and cultural acceptability of a biblically-based healthy eating and weight loss intervention: A mixed methods research study.  Finch K.A., Starks S., Leak C.L., Graff J.C., Surbhi S., Gaffney S.M., Duncan M., Bailey J.E.  Journal of General Internal Medicine. Conference: Annual Meeting of the Society of General Internal Medicine, SGIM 2020. Birmingham, AL United States. 35(SUPPL 1) (pp S223), 2020. Date of Publication: July 2020.  [Conference Abstract]  AN: 633957165 | Not relevant to the main subject/ wrong population or geographical location/ duplicate or repetition |
|  | Evaluation of the care for diabetes patients by general practitioners in the province of khouribga, Morocco. Evaluation de la prise en charge des diabetiques par le medecin generaliste dans la province de khouribga (Maroc) <Evaluation de la prise en charge des diabetiques par le medecin generaliste dans la province de khouribga (Maroc).>  Hassoune S., Badri S., Nani S., Belhadi L., Maaroufi A.  Eastern Mediterranean Health Journal. 19(1) (pp 52-58), 2013. Date of Publication: 2013.  [Article]  AN: 373941530 | Not relevant to the main subject/ wrong population or geographical location/ duplicate or repetition |
|  | Impact of directed health assessments for patients with diabetes in the age of COVID-19.  Astle K., Hohmann N., Stringer K.  JACCP Journal of the American College of Clinical Pharmacy. Conference: 2020 ACCP Annual Meeting. Virtual. 3(8) (pp 1547-1548), 2020. Date of Publication: 2020.  [Conference Abstract]  AN: 634123147 | Not relevant to the main subject/ wrong population or geographical location/ duplicate or repetition |
|  | Prevention of stroke: a global perspective.  Pandian J.D., Gall S.L., Kate M.P., Silva G.S., Akinyemi R.O., Ovbiagele B.I., Lavados P.M., Gandhi D.B.C., Thrift A.G.  The Lancet. 392(10154) (pp 1269-1278), 2018. Date of Publication: October 2018.  [Review]  AN: 2001154602 | Not relevant to the main subject/ wrong population or geographical location/ duplicate or repetition |
|  | Artificial intelligence using deep learning to screen for referable and vision-threatening diabetic retinopathy in Africa: a clinical validation study.  Bellemo V., Lim Z.W., Lim G., Nguyen Q.D., Xie Y., Yip M.Y.T., Hamzah H., Ho J., Lee X.Q., Hsu W., Lee M.L., Musonda L., Chandran M., Chipalo-Mutati G., Muma M., Tan G.S.W., Sivaprasad S., Menon G., Wong T.Y., Ting D.S.W.  The Lancet. Digital health. 1(1) (pp e35-e44), 2019. Date of Publication: 01 May 2019.  [Article]  AN: 634088317 | Not relevant to the main subject/ wrong population or geographical location/ duplicate or repetition |
|  | Optimizing Peripandemic Care for Veteran Major Non-Traumatic Lower Extremity Amputees: A Proposal Informed by a National Retrospective Descriptive Analysis of COVID-19 Risk Factor Prevalence.  Dittman J.M., Tse W., Amendola M.F.  Military medicine. 185(11-12) (pp e2124-e2130), 2020. Date of Publication: 30 Dec 2020.  [Article]  AN: 632235852 | Not relevant to the main subject/ wrong population or geographical location/ duplicate or repetition |
|  | Effects of ginger powder supplementation on glycemic status and lipid profile in newly diagnosed obese patients with type 2 diabetes mellitus.  El Gayar M.H., Aboromia M.M.M., Ibrahim N.A., Abdel Hafiz M.H.  Obesity Medicine. 14 (no pagination), 2019. Article Number: 100094. Date of Publication: June 2019.  [Article]  AN: 2001968213 | Not relevant to the main subject/ wrong population or geographical location/ duplicate or repetition |
|  | Financial Incentives and Nurse Coaching to Enhance Diabetes Outcomes (FINANCE-DM): A trial protocol.  Egede L.E., Walker R., Williams J.S., Knapp R., Dismuke C.E., Davidson T., Campbell J.A.  BMJ Open. 10(12) (no pagination), 2020. Article Number: e043760. Date of Publication: 22 Dec 2020.  [Article]  AN: 633739401 | Not relevant to the main subject/ wrong population or geographical location/ duplicate or repetition |
|  | Knowledge of gestational diabetes mellitus at first consultation in a multi-ethnic pregnant population in the Oslo region, Norway - a cross-sectional study.  Borgen I., Garnweidner-Holme L.M., Jacobsen A.F., Fayyad S., Cvancarova Smastuen M., Lukasse M.  Ethnicity & health. (pp 1-14), 2019. Date of Publication: 16 Aug 2019.  [Article]  AN: 629093984 | Not relevant to the main subject/ wrong population or geographical location/ duplicate or repetition |
|  | Leveraging public-private partnerships to provide a person-centred high-tech high-touch approach to obesity management in Lagos, Nigeria.  Mobisson N., Bankole F., Njoku K., Adediran O., Adebo A.  Obesity Reviews. Conference: European and International Congress on Obesity, ECOICO 2020. Virtual. 21(SUPPL 1) (no pagination), 2020. Date of Publication: 2020.  [Conference Abstract]  AN: 633902162 | Not relevant to the main subject/ wrong population or geographical location/ duplicate or repetition |
|  | Telephone based weight loss intervention: Relevance for developing countries.  Ayisi Addo S., Steiner-Asiedu M.  Critical reviews in food science and nutrition. 59(13) (pp 2095-2101), 2019. Date of Publication: 2019.  [Article]  AN: 628716896 | Not relevant to the main subject/ wrong population or geographical location/ duplicate or repetition |
|  | Predictors of acute coronary syndrome in pre hospital patients with chest pain. Facteurs predictifs du syndrome coronaire aigu chez les patients souffrant de douleur thoracique en pre hospitalier <Facteurs predictifs du syndrome coronaire aigu chez les patients souffrant de douleur thoracique en pre hospitalier.>  Zelfani S., Boudiche S., Manai H., Mourali S., Daghfous M.  Tunisie Medicale. 98(1) (pp 55-59), 2020. Date of Publication: January 2020.  [Article]  AN: 2004200586 | Not relevant to the main subject/ wrong population or geographical location/ duplicate or repetition |
|  | Lipid-based gliclazide nanoparticles for treatment of diabetes: Formulation, pharmacokinetics, pharmacodynamics and subacute toxicity study.  Nazief A.M., Hassaan P.S., Khalifa H.M., Sokar M.S., El-Kamel A.H.  International Journal of Nanomedicine. 15 (pp 1129-1148), 2020. Date of Publication: 2020.  [Article]  AN: 2003818537 | Not relevant to the main subject/ wrong population or geographical location/ duplicate or repetition |
|  | Mobile-Enhanced Peer Support for African Americans with Type 2 Diabetes: a Randomized Controlled Trial.  Presley C., Agne A., Shelton T., Oster R., Cherrington A.  Journal of General Internal Medicine. 35(10) (pp 2889-2896), 2020. Date of Publication: 01 Oct 2020.  [Article]  AN: 2005654210 | Not relevant to the main subject/ wrong population or geographical location/ duplicate or repetition |
|  | Integration of paper microfluidic sensors into contact lenses for tear fluid analysis.  Moreddu R., Elsherif M., Adams H., Moschou D., Cordeiro M.F., Wolffsohn J.S., Vigolo D., Butt H., Cooper J.M., Yetisen A.K.  Lab on a Chip. 20(21) (pp 3970-3979), 2020. Date of Publication: 07 Nov 2020.  [Article]  AN: 633223881 | Not relevant to the main subject/ wrong population or geographical location/ duplicate or repetition |
|  | Diabetes and CVD Risk: Special Considerations in African Americans Related to Care.  Wittwer J.A., Golden S.H., Joseph J.J.  Current Cardiovascular Risk Reports. 14(10) (no pagination), 2020. Article Number: 15. Date of Publication: 01 Oct 2020.  [Review]  AN: 2005798825 | Not relevant to the main subject/ wrong population or geographical location/ duplicate or repetition |
|  | Ecg changes in a patient with covid-19 treated with hydroxychloroquine and azithromycin. Electrocardiogrammes d'un patient covid-19 traite par l'association hydroxychloroquine et azithromycine <Electrocardiogrammes d'un patient covid-19 traite par l'association hydroxychloroquine et azithromycine.>  Benouna M.E., Ech-Chenbouli A.  Pan African Medical Journal. 35(Supplement 2) (pp 1-3), 2020. Article Number: 112. Date of Publication: 2020.  [Article]  AN: 2005480405 | Not relevant to the main subject/ wrong population or geographical location/ duplicate or repetition |
|  | Feasibility of Long-term Proteasome Inhibition in Multiple Myeloma by in-class Transition From Bortezomib to Ixazomib.  Manda S., Yimer H.A., Noga S.J., Girnius S., Yasenchak C.A., Charu V., Lyons R., Aiello J., Bogard K., Ferrari R.H., Cherepanov D., Demers B., Lu V., Whidden P., Kambhampati S., Birhiray R.E., Jhangiani H.S., Boccia R., Rifkin R.M.  Clinical Lymphoma, Myeloma and Leukemia. 20(11) (pp e910-e925), 2020. Date of Publication: November 2020.  [Article]  AN: 2007738013 | Not relevant to the main subject/ wrong population or geographical location/ duplicate or repetition |
|  | Barriers to initiation of insulin therapy in poorly controlled type 2 diabetes based on self-determination theory. Obstacles face a la mise en place de l'insulinotherapie dans le diabete de type 2 mal controle selon la theorie de l'autodetermination <Obstacles face a la mise en place de l'insulinotherapie dans le diabete de type 2 mal controle selon la theorie de l'autodetermination.>  Khaloo P., Rabizadeh S., Alemi H., Salehi S., Majdzadeh R., Mirmiranpour H., Rajab A., Esteghamati A., Nakhjavani M.  Eastern Mediterranean Health Journal. 26(11) (pp 1331-1338), 2020. Date of Publication: November 2020.  [Article]  AN: 2005521716 | Not relevant to the main subject/ wrong population or geographical location/ duplicate or repetition |
|  | Racial differences in the effectiveness of a multifactorial telehealth intervention to slow diabetic kidney disease.  Kobe E.A., Diamantidis C.J., Bosworth H.B., Davenport C.A., Oakes M., Alexopoulos A.-S., Pendergast J., Patel U.D., Crowley M.J.  Medical Care. 58(11) (pp 968-973), 2020. Date of Publication: 01 Nov 2020.  [Article]  AN: 633245037 | Not relevant to the main subject/ wrong population or geographical location/ duplicate or repetition |
|  | Practical telehealth to improve control and engagement for patients with clinic-refractory diabetes mellitus (PRACTICE-DM): Protocol and baseline data for a randomized trial.  Kobe E.A., Edelman D., Tarkington P.E., Bosworth H.B., Maciejewski M.L., Steinhauser K., Jeffreys A.S., Coffman C.J., Smith V.A., Strawbridge E.M., Szabo S.T., Desai S., Garrett M.P., Wilmot T.C., Marcano T.J., Overby D.L., Tisdale G.A., Durkee M., Bullard S., Dar M.S., Mundy A.C., Hiner J., Fredrickson S.K., Majette Elliott N.T., Howard T., Jeter D.H., Danus S., Crowley M.J.  Contemporary Clinical Trials. 98 (no pagination), 2020. Article Number: 106157. Date of Publication: November 2020.  [Article]  AN: 2008326658 | Not relevant to the main subject/ wrong population or geographical location/ duplicate or repetition |
|  | Critical appraisal of a mHealth-assisted community-based cardiovascular disease risk screening program in rural Kenya: an operational research study.  Aw M., Ochieng B.O., Attambo D., Opot D., Aw J., Francis S., Hawkes M.T.  Pathogens and Global Health. 114(7) (pp 379-387), 2020. Date of Publication: 02 Oct 2020.  [Article]  AN: 2006095214 | Not relevant to the main subject/ wrong population or geographical location/ duplicate or repetition |
|  | Indirect implications of COVID-19 prevention strategies on non-communicable diseases.  Modesti P.A., Wang J., Damasceno A., Agyemang C., Van Bortel L., Persu A., Zhao D., Jarraya F., Marzotti I., Bamoshmoosh M., Parati G., Schutte A.E.  BMC Medicine. 18(1) (no pagination), 2020. Article Number: 256. Date of Publication: 14 Aug 2020.  [Article]  AN: 632573620 | Not relevant to the main subject/ wrong population or geographical location/ duplicate or repetition |
|  | A systematic review of the spectrum of cardiac arrhythmias in Sub-Saharan Africa.  Yuyun M.F., Bonny A., Ng G.A., Sliwa K., Kengne A.P., Chin A., Mocumbi A.O., Ngantcha M., Ajijola O.A., Bukhman G.  Global Heart. 15(1) (no pagination), 2020. Article Number: 37. Date of Publication: 08 May 2020.  [Review]  AN: 2007065429 | Not relevant to the main subject/ wrong population or geographical location/ duplicate or repetition |
|  | COVID-19 outbreak and pediatric diabetes: Perceptions of health care professionals worldwide.  Elbarbary N.S., dos Santos T.J., de Beaufort C., Agwu J.C., Calliari L.E., Scaramuzza A.E.  Pediatric Diabetes. 21(7) (pp 1083-1092), 2020. Date of Publication: 01 Nov 2020.  [Article]  AN: 2005876580 | Not relevant to the main subject/ wrong population or geographical location/ duplicate or repetition |
|  | Mobile technologies to support healthcare provider to healthcare provider communication and management of care.  Goncalves-Bradley D.C., J Maria A.R., Ricci-Cabello I., Villanueva G., Fonhus M.S., Glenton C., Lewin S., Henschke N., Buckley B.S., Mehl G.L., Tamrat T., Shepperd S.  Cochrane Database of Systematic Reviews. 2020(8) (no pagination), 2020. Article Number: CD012927. Date of Publication: 18 Aug 2020.  [Review]  AN: 632609657 | Not relevant to the main subject/ wrong population or geographical location/ duplicate or repetition |
|  | Impact of mobile phone text messaging intervention on adherence among patients with diabetes in a rural setting: A randomized controlled trial.  Owolabi E.O., Goon D.T., Ajayi A.I.  Medicine (United States). 99(12) (no pagination), 2020. Article Number: e18953. Date of Publication: 2020.  [Review]  AN: 632820769 | Not relevant to the main subject/ wrong population or geographical location/ duplicate or repetition |
|  | Non-hospitalized Adults with COVID-19 Differ Noticeably from Hospitalized Adults in Their Demographic, Clinical, and Social Characteristics.  Bergquist S.H., Partin C., Roberts D.L., O'Keefe J.B., Tong E.J., Zreloff J., Jarrett T.L., Moore M.A.  SN Comprehensive Clinical Medicine. 2(9) (pp 1349-1357), 2020. Date of Publication: 01 Sep 2020.  [Article]  AN: 2005862598 | Not relevant to the main subject/ wrong population or geographical location/ duplicate or repetition |
|  | COVID-19 in people living with diabetes: An international consensus.  Caballero A.E., Ceriello A., Misra A., Aschner P., McDonnell M.E., Hassanein M., Ji L., Mbanya J.C., Fonseca V.A.  Journal of Diabetes and its Complications. 34(9) (no pagination), 2020. Article Number: 107671. Date of Publication: September 2020.  [Article]  AN: 2006988363 | Not relevant to the main subject/ wrong population or geographical location/ duplicate or repetition |
|  | Prevalence and factors associated with depression among type 2 diabetes patients in a Reference Hospital in Cameroon.  Aroke D., Mapoure Y.N., Mbarga T.N.F., Dimala C.A., Danwe V.K., Njamnshi A.K., Choukem S.-P.  Neurology Psychiatry and Brain Research. 37 (pp 123-128), 2020. Date of Publication: September 2020.  [Article]  AN: 2007511010 | Not relevant to the main subject/ wrong population or geographical location/ duplicate or repetition |
|  | Value and Feasibility of Telephone Follow-Up in Ethiopian Surgical Patients.  Starr N., Gebeyehu N., Tesfaye A., Forrester J.A., Bekele A., Bitew S., Wayessa E., Weiser T.G., Negussie T.  Surgical Infections. 21(6) (pp 533-539), 2020. Date of Publication: August 2020.  [Article]  AN: 632607057 | Not relevant to the main subject/ wrong population or geographical location/ duplicate or repetition |
|  | Smartphone-assisted glaucoma screening in patients with type 2 diabetes: A pilot study.  Bilong Y., Domngang C.N., Nwanlih G.G., Katte J.-C., Afetane T.E., Kagmeni G., Mbanya J.C., Kumar N., Sharma A., Sobngwi E.  Medical Hypothesis, Discovery, and Innovation in Ophthalmology. 9(1) (pp 61-95), 2020. Date of Publication: Spring 2020.  [Article]  AN: 2004133857 | Not relevant to the main subject/ wrong population or geographical location/ duplicate or repetition |
|  | Debate: Exposing the most serious infirmity - racism's impact on health in the era of COVID-19.  Farquharson W.H., Thornton C.J.  Child and Adolescent Mental Health. 25(3) (pp 182-183), 2020. Date of Publication: 01 Sep 2020.  [Note]  AN: 2005617818 | Not relevant to the main subject/ wrong population or geographical location/ duplicate or repetition |
|  | The management of diabetes in everyday life study: Design and methods for a pragmatic randomized controlled trial comparing the effectiveness of text messaging versus health coaching.  Bailey J.E., Surbhi S., Gatwood J., Butterworth S., Coday M., Shuvo S.A., Dashputre A.A., Brooks I.M., Binkley B.L., Riordan C.J., Steinberg H.O., Gutierrez M.L., Haley L.E., Leak C.L., Tolley E.A.  Contemporary Clinical Trials. 96 (no pagination), 2020. Article Number: 106080. Date of Publication: September 2020.  [Article]  AN: 2007144835 | Not relevant to the main subject/ wrong population or geographical location/ duplicate or repetition |
|  | Personal health of spine surgeons can impact perceptions, decision-making and healthcare delivery during the COVID-19 pandemic-a worldwide study.  Sayari A.J., Harada G.K., Louie P.K., McCarthy M.H., Nolte M.T., Mallow G.M., Siyaji Z., Germscheid N., Cheung J.P.Y., Neva M.H., El-Sharkawi M., Valacco M., Sciubba D.M., Chutkan N.B., An H.S., Samartzis D.  Neurospine. 17(2) (pp 313-330), 2020. Date of Publication: June 2020.  [Article]  AN: 2004632145 | Not relevant to the main subject/ wrong population or geographical location/ duplicate or repetition |
|  | Universal HIV Testing and Treatment (UTT) Integrated with Chronic Disease Screening and Treatment: the SEARCH study.  Chamie G., Hickey M.D., Kwarisiima D., Ayieko J., Kamya M.R., Havlir D.V.  Current HIV/AIDS Reports. 17(4) (pp 315-323), 2020. Date of Publication: 01 Aug 2020.  [Review]  AN: 2005166122 | Not relevant to the main subject/ wrong population or geographical location/ duplicate or repetition |
|  | Exposure to waste sites and their impact on health: a panel and geospatial analysis of nationally representative data from South Africa, 2008-2015.  Tomita A., Cuadros D.F., Burns J.K., Tanser F., Slotow R.  The Lancet Planetary Health. 4(6) (pp e223-e234), 2020. Date of Publication: June 2020.  [Article]  AN: 2006740839 | Not relevant to the main subject/ wrong population or geographical location/ duplicate or repetition |
|  | Brief report: International perspectives on the pediatric COVID-19 experience.  Yilmaz O., Gochicoa-Rangel L., Blau H., Epaud R., Lands L.C., Lombardi E., Moore P.E., Stein R.T., Wong G.W.K., Zar H.J.  Pediatric Pulmonology. 55(7) (pp 1598-1600), 2020. Date of Publication: 01 Jul 2020.  [Note]  AN: 2004810736 | Not relevant to the main subject/ wrong population or geographical location/ duplicate or repetition |
|  | A qualitative study of users' experiences after 3 months: the first Rwandan diabetes self-management Smartphone application "Kir'App".  Kabeza C.B., Harst L., Schwarz P.E.H., Timpel P.  Therapeutic Advances in Endocrinology and Metabolism. 11 (no pagination), 2020. Date of Publication: 2020.  [Article]  AN: 2004912887 | Not relevant to the main subject/ wrong population or geographical location/ duplicate or repetition |
|  | The potential of mobile health clinics in chronic disease prevention and health promotion in universal healthcare systems. An on-field experiment.  Bertoncello C., Cocchio S., Fonzo M., Bennici S.E., Russo F., Putoto G.  International Journal for Equity in Health. 19(1) (no pagination), 2020. Article Number: 59. Date of Publication: 01 May 2020.  [Article]  AN: 631654452 | Not relevant to the main subject/ wrong population or geographical location/ duplicate or repetition |
|  | Upheaval in cancer care during the COVID-19 outbreak.  Salako O., Okunade K., Allsop M., Habeebu M., Toye M., Oluyede G., Fagbenro G., Salako B.  ecancermedicalscience. 14 (no pagination), 2020. Article Number: ed97. Date of Publication: 01 Apr 2020.  [Article]  AN: 2005841315 | Not relevant to the main subject/ wrong population or geographical location/ duplicate or repetition |
|  | Cost-effectiveness of diabetic retinopathy screening programs using telemedicine: A systematic review.  Avidor D., Loewenstein A., Waisbourd M., Nutman A.  Cost Effectiveness and Resource Allocation. 18(1) (no pagination), 2020. Article Number: 16. Date of Publication: 06 Apr 2020.  [Review]  AN: 631486214 | Not relevant to the main subject/ wrong population or geographical location/ duplicate or repetition |
|  | Impact of a telephonic intervention to improve diabetes control on health care utilization and cost for adults in South Bronx, New York.  Tabaei B.P., Howland R.E., Gonzalez J.S., Chamany S., Walker E.A., Schechter C.B., Wu W.Y.  Diabetes Care. 43(4) (pp 743-750), 2020. Date of Publication: 01 Apr 2020.  [Article]  AN: 2005395589 | Not relevant to the main subject/ wrong population or geographical location/ duplicate or repetition |
|  | In-vitro and in-vivo performance of locally manufactured glimepiride tablet generics compared to the innovator (Amaryl) tablets.  Abou-Taleb B.A., Megallaa M.H., Khalafallah N.M., Khalil S.H.  Drug Development and Industrial Pharmacy. 46(2) (pp 192-199), 2020. Date of Publication: 01 Feb 2020.  [Article]  AN: 2004144366 | Not relevant to the main subject/ wrong population or geographical location/ duplicate or repetition |
|  | Role of obstetric ultrasound in reducing maternal and neonatal mortality in developing countries: From facts to acts.  Stefanovic V.  Donald School Journal of Ultrasound in Obstetrics and Gynecology. 14(1) (pp 43-49), 2020. Date of Publication: January-March 2020.  [Review]  AN: 2003865180 | Not relevant to the main subject/ wrong population or geographical location/ duplicate or repetition |
|  | Diabetes detection and communication among patients admitted through the emergency department of a public hospital.  Levi O.U., Webb F., Simmons D.  International Journal of Environmental Research and Public Health. 17(3) (no pagination), 2020. Article Number: 980. Date of Publication: 01 Feb 2020.  [Article]  AN: 2003718321 | Not relevant to the main subject/ wrong population or geographical location/ duplicate or repetition |
|  | Mobile Phone Use in the Management of Diabetes in Nigeria: A New Potential Weapon.  Olamoyegun M.A., Emuoyibofarhe O.J., Ala O.A., Ugwu E.  West African journal of medicine. 37(3) (pp 201-208), 2020. Date of Publication: 01 Jul 2020.  [Article]  AN: 631955273 | Not relevant to the main subject/ wrong population or geographical location/ duplicate or repetition |
|  | Recent advances in computational tools and resources for the self-management of type 2 diabetes.  Moonian O., Jodheea-Jutton A., Khedo K.K., Baichoo S., Nagowah S.D., Nagowah L., Mungloo-Dilmohamud Z., Cheerkoot-Jalim S.  Informatics for health & social care. 45(1) (pp 77-95), 2020. Date of Publication: 01 Jan 2020.  [Review]  AN: 630450680 | Not relevant to the main subject/ wrong population or geographical location/ duplicate or repetition |
|  | Magnitude of sedentary behavior and associated factors among secondary school adolescents in Debre Berhan town, Ethiopia.  Mohammed O.Y., Tesfahun E., Mohammed A.  BMC public health. 20(1) (pp 86), 2020. Date of Publication: 20 Jan 2020.  [Article]  AN: 630671770 | Not relevant to the main subject/ wrong population or geographical location/ duplicate or repetition |
|  | Efficacy, acceptability and feasibility of daily text-messaging in promoting glycaemic control and other clinical outcomes in a low-resource setting of South Africa: A randomised controlled trial.  Owolabi E.O., Goon D.T., Ajayi A.I.  PLoS ONE. 14(11) (no pagination), 2019. Article Number: e0224791. Date of Publication: 2019.  [Article]  AN: 2003994670 | Included |
|  | Barriers to the Use of Mobile Health in Improving Health Outcomes in Developing Countries: Systematic Review.  Kruse C., Betancourt J., Ortiz S., Valdes Luna S.M., Bamrah I.K., Segovia N.  Journal of medical Internet research. 21(10) (pp e13263), 2019. Date of Publication: 09 Oct 2019.  [Review]  AN: 629551653 | Not relevant to the main subject/ wrong population or geographical location/ duplicate or repetition |
|  | Corrigendum: How do smart device apps for diabetes self-management correspond with theoretical indicators of empowerment? an analysis of app features (International Journal of Technology Assessment in Health Care (2019) 35 (150-159) DOI: 10.1017/S0266462319000163).  Brew-Sam N., Chib A.  International Journal of Technology Assessment in Health Care. 35(3) (pp 252), 2019. Date of Publication: 2019.  [Erratum]  AN: 628290397 | Not relevant to the main subject/ wrong population or geographical location/ duplicate or repetition |
|  | Optimising orbit counting of arbitrary order by equation selection.  Melckenbeeck I., Audenaert P., Van Parys T., Van De Peer Y., Colle D., Pickavet M.  BMC bioinformatics. 20(1) (pp 27), 2019. Date of Publication: 15 Jan 2019.  [Article]  AN: 625962381 | Not relevant to the main subject/ wrong population or geographical location/ duplicate or repetition |
|  | Morphine induced respiratory depression in an adult sickle cell disease patient.  Otokwala J.G.  Journal of the Intensive Care Society. Conference: Intensive Care Society State of the Art 2019. Birmingham United Kingdom. 21(2 SUPPL) (pp 220-221), 2020. Date of Publication: 2020.  [Conference Abstract]  AN: 633777623 | Not relevant to the main subject/ wrong population or geographical location/ duplicate or repetition |
|  | Cellular-enabled glucometer use in a minority medicaid population improved HbA1c reduction.  Osman N., Folawewo K., Ganta V., Hodge M., Nunlee-Bland G., Odonkor W., Zenebe A.  Endocrine Practice. Conference: 2020 American Association of Clinical Endocrinologists Annual Scientific and Clinical Congress, AACE 2020. Washington DC United States. 26(SUPPL 2) (pp 100-101), 2020. Date of Publication: May 2020.  [Conference Abstract]  AN: 633717921 | Not relevant to the main subject/ wrong population or geographical location/ duplicate or repetition |
|  | Dialysis care during the COVID-19 (C19) pandemic: A large urban academic dialysis center experience.  Navarrete J.E., Oliver K., Ishmail T., Franch H.A., Apata I.W.  Journal of the American Society of Nephrology. Conference: Kidney Week 2020. Virtual United States. 31 (pp 804), 2020. Date of Publication: 2020.  [Conference Abstract]  AN: 633696996 | Not relevant to the main subject/ wrong population or geographical location/ duplicate or repetition |
|  | Development of a gestational diabetes selfmanagement and remote monitoring mobile platform.  Collier J., Fortuin J., Adams S.  Journal of Diabetes Science and Technology. Conference: 19th Annual Diabetes Technology Meeting, DTM 2019. Bethesda, MD United States. 14(2) (pp A24), 2020. Date of Publication: 2020.  [Conference Abstract]  AN: 633682574 | Not relevant to the main subject/ wrong population or geographical location/ duplicate or repetition |
|  | PNS116 Maintaining Routine Healthcare for Chronic Medical Conditions during the COVID-19 Pandemic: What WE CAN Learn from Australia.  Thet Lwin Z.M., Higgins T.C., Winn K., Naing K.H.  Value in Health. Conference: Virtual ISPOR Europe 2020. Virtual, Online. 23(Supplement 2) (pp S660), 2020. Date of Publication: December 2020.  [Conference Abstract]  AN: 2010301078 | Not relevant to the main subject/ wrong population or geographical location/ duplicate or repetition |
|  | Scoping Review of Outpatient Diabetic Retinopathy Screening.  Carter A.S.  Journal of the National Medical Association. Conference: 2020 NMA Annual Meeting. Virtual, Online. 112(5 Supplement) (pp S17), 2020. Date of Publication: October 2020.  [Conference Abstract]  AN: 2010302092 | Not relevant to the main subject/ wrong population or geographical location/ duplicate or repetition |
|  | PDB1 Telemedicine Use for Gestational Diabetes: A Systematic Review and Meta-Analysis of Randomised Controlled Trials.  Leblalta B., Kebaili H., Lee S.  Value in Health Regional Issues. Conference: ISPOR Asia Pacific 2020. Virtual, Online. 22(Supplement) (pp S32), 2020. Date of Publication: September 2020.  [Conference Abstract]  AN: 2007669616 | Not relevant to the main subject/ wrong population or geographical location/ duplicate or repetition |
|  | Assessing diabetic retinopathy severity and specialist follow-up rate in a diabetic telemedicine study.  Cohen D.C., Sun J., Benjamin J., Henderer J., Barbera A., Chen L., Grachevskaya J., Zhang Y.  Investigative Ophthalmology and Visual Science. Conference: 2020 Annual Meeting Association for Research in Vision and Ophthalmology, ARVO 2020. Baltimore, MD United States. 61(7) (no pagination), 2020. Date of Publication: 2020.  [Conference Abstract]  AN: 632695166 | Not relevant to the main subject/ wrong population or geographical location/ duplicate or repetition |
|  | Impact of Artificial Intelligence for Diabetic Retinopathy Screening in a Malawi, Africa Clinic.  Soliz P., Zamora G., Aslan J., Nemeth S.C., Benson J., Burgess P.  Investigative Ophthalmology and Visual Science. Conference: 2020 Annual Meeting Association for Research in Vision and Ophthalmology, ARVO 2020. Baltimore, MD United States. 61(7) (no pagination), 2020. Date of Publication: 2020.  [Conference Abstract]  AN: 632694921 | Not relevant to the main subject/ wrong population or geographical location/ duplicate or repetition |
|  | Cutaneous crospovidone due to "skin popping".  Marka A., Hoyt B.S., Dagrosa A.T., Barton D.T., Kim A., Linos K., Yank S.  American Journal of Dermatopathology. Conference: 40th Symposium of the International Society of Dermatopathology, ISDP 2019. Lisbon Portugal. 42(6) (pp e79), 2020. Date of Publication: 2020.  [Conference Abstract]  AN: 632594381 | Not relevant to the main subject/ wrong population or geographical location/ duplicate or repetition |
|  | HEALTHY DIET/LIFESTYLE TEXT- MESSAGE BASED INTERVENTION IN PATIENTS WITH CHRONIC KIDNEY DISEASE.  Kagalwalla M., Iddrisu M.-D., Missikpode C., Frydrych A., Wilk M., Gerber B., Sharp L., Lash J., Cedillo-Couvert E., Porter A.  American Journal of Kidney Diseases. Conference: NKF 2020 Spring Clinical Meetings. New Orleans United States. 75(4) (pp 592), 2020. Date of Publication: April 2020.  [Conference Abstract]  AN: 2005717133 | Not relevant to the main subject/ wrong population or geographical location/ duplicate or repetition |
|  | Dissecting myocardium.  Laymouna R., Samoka A., Haggag A., Almaghraby A., Hassan Y., El-Sharkawy E., Zaki A.  European Heart Journal Cardiovascular Imaging. Conference: Annual Meeting of the European Association of Echocardiography, EUROECHO 2019. Vienna Australia. 21(Supplement 1) (pp i546), 2020. Date of Publication: January 2020.  [Conference Abstract]  AN: 631348806 | Not relevant to the main subject/ wrong population or geographical location/ duplicate or repetition |
|  | Adherence to Dietary Regimen and Recommended Physical Activity: A missing Link in the Management of Diabetes in Low-Resource Settings of Eastern Cape South Africa.  Owolabi E.O.  Metabolism: Clinical and Experimental. Conference: 17th Annual World Congress On Insulin Resistance, Diabetes, And Cardiovascular Disease 2019. The Metabolic Institute of America (TMIOA), Los Angeles United States. 104(Supplement) (no pagination), 2020. Article Number: 154058. Date of Publication: March 2020.  [Conference Abstract]  AN: 2004712684 | Not relevant to the main subject/ wrong population or geographical location/ duplicate or repetition |
|  | High Level of Acceptability and Feasibility with Low Level of Efficacy of Daily Text-Messaging on Glycaemic Status and Self-Management: Result of a Randomised Trial Among Low-Income Earning Black South Africans.  Owolabi E.O., Ter Goon D., Ajayi A.I.  Metabolism: Clinical and Experimental. Conference: 17th Annual World Congress On Insulin Resistance, Diabetes, And Cardiovascular Disease 2019. The Metabolic Institute of America (TMIOA), Los Angeles United States. 104(Supplement) (no pagination), 2020. Article Number: 154057. Date of Publication: March 2020.  [Conference Abstract]  AN: 2004712727 | Not relevant to the main subject/ wrong population or geographical location/ duplicate or repetition |
|  | Delays in management of ST-segment elevation myocardial infarction.  Bezdah L., Ben Ahmed I., Mrabet A., Saidi O., Ben Romdhane H.  Archives of Cardiovascular Diseases Supplements. Conference: 30es JOURNEES EUROPEENNES de la SOCIETE FRANCAISE de CARDIOLOGIE. Paris France. 12(1) (pp 25), 2020. Date of Publication: January 2020.  [Conference Abstract]  AN: 2003913262 | Not relevant to the main subject/ wrong population or geographical location/ duplicate or repetition |
|  | 366 Factors Impacting Post-operative Opioid Use among Patients Undergoing Implantation of Inflatable Penile Prosthesis.  Mohan C., Ehlers M., McGowan M., Akerman J., McCormick B., Coward R.M., Figler B.  Journal of Sexual Medicine. Conference: 20th Annual Fall Scientific Meeting of SMSNA. Omni Nashville Hotel, Nashville United States. 17(1 Supplement 1) (pp S100), 2020. Date of Publication: January 2020.  [Conference Abstract]  AN: 2004351846 | Not relevant to the main subject/ wrong population or geographical location/ duplicate or repetition |
|  | Corrigendum to "Assessment of Rwandan diabetic patients' needs and expectations to develop their first diabetes self-management smartphone application (Kir'App)" (Therapeutic Advances in Endocrinology and Metabolism, (2019), 10, (1-21), 10.1177/2042018819845318).  Anonymous  Therapeutic Advances in Endocrinology and Metabolism. 11 (no pagination), 2020. Date of Publication: 2020.  [Erratum]  AN: 2007503403 | Not relevant to the main subject/ wrong population or geographical location/ duplicate or repetition |
|  | Design and Development of Diabetes Management System Using Machine Learning.  Sowah R.A., Bampoe-Addo A.A., Armoo S.K., Saalia F.K., Gatsi F., Sarkodie-Mensah B.  International Journal of Telemedicine and Applications. 2020 (no pagination), 2020. Article Number: 8870141. Date of Publication: 2020.  [Article]  AN: 2007350301 | Not relevant to the main subject/ wrong population or geographical location/ duplicate or repetition |
|  | Assessment of Rwandan diabetic patients' needs and expectations to develop their first diabetes self-management smartphone application (Kir'App).  Kabeza C.B., Harst L., Schwarz P.E.H., Timpel P.  Therapeutic Advances in Endocrinology and Metabolism. 10 (no pagination), 2019. Date of Publication: 2019.  [Article]  AN: 2003619897 | Not relevant to the main subject/ wrong population or geographical location/ duplicate or repetition |
|  | Boosting diabetes and pre-diabetes detection in rural Ghana.  Nyarko B.E., Amoah R.S., Crimi A.  F1000Research. 8 (no pagination), 2019. Article Number: 289. Date of Publication: 2019.  [Article]  AN: 629293364 | Not relevant to the main subject/ wrong population or geographical location/ duplicate or repetition |
|  | Self-management of non-communicable diseases in low- And middle-income countries: A scoping review.  Hearn J., Ssinabulya I., Schwartz J.I., Akiteng A.R., Ross H.J., Cafazzo J.A.  PLoS ONE. 14(7) (no pagination), 2019. Article Number: e0219141. Date of Publication: 2019.  [Article]  AN: 2002368798 | Not relevant to the main subject/ wrong population or geographical location/ duplicate or repetition |
|  | NIBG: An efficient near-infrared spectroscopy-based device for tele-monitoring blood glucose level of diabetes outpatients in an e-health system.  Olakanmi O., Kamil I.A., Atilola O.P.  International Journal of Biomedical Engineering and Technology. 30(2) (pp 95-112), 2019. Date of Publication: 2019.  [Article]  AN: 628469669 | Not relevant to the main subject/ wrong population or geographical location/ duplicate or repetition |
|  | Risk factors for open-Angle glaucoma in persons of latin american descent.  Wurster P., Harris A., Gonzalez A.C., Adjei S., Verticchio Vercellin A., Mathew S., Lang M., Eikenberry J., Siesky B.  Journal of Glaucoma. 29(3) (pp 217-225), 2020. Date of Publication: 01 Mar 2020.  [Article]  AN: 630578324 | Not relevant to the main subject/ wrong population or geographical location/ duplicate or repetition |
|  | Mobile phone access and comfort: implications for HIV and tuberculosis care in India and South Africa.  Cox S.N., Elf J.L., Lokhande R., Ogale Y.P., DiAndreth L., Dupuis E., Milovanovic M., Mpungose N., Mave V., Suryavanshi N., Gupta A., Martinson N., Golub J.E., Mathad J.S.  The international journal of tuberculosis and lung disease : the official journal of the International Union against Tuberculosis and Lung Disease. 23(7) (pp 865-872), 2019. Date of Publication: 01 Jul 2019.  [Article]  AN: 629157672 | Not relevant to the main subject/ wrong population or geographical location/ duplicate or repetition |
|  | Ethnic and gender differences in the management of type 2 diabetes: a cross-sectional study from Norwegian general practice.  Tran A.T., Berg T.J., Gjelsvik B., Mdala I., Thue G., Cooper J.G., Nokleby K., Claudi T., Bakke A., Sandberg S., Jenum A.K.  BMC health services research. 19(1) (pp 904), 2019. Date of Publication: 28 Nov 2019.  [Article]  AN: 630014457 | Not relevant to the main subject/ wrong population or geographical location/ duplicate or repetition |
|  | Effectiveness of digital health using the transtheoretical model to prevent or delay type 2 diabetes in impaired glucose tolerance patients: protocol for a randomized control trial.  Alzeidan R., Shata Z., Hassounah M.M., Baghdadi L.R., Hersi A., Fayed A., Kashour T., Elmorshedy H.  BMC public health. 19(1) (pp 1550), 2019. Date of Publication: 21 Nov 2019.  [Article]  AN: 629946198 | Not relevant to the main subject/ wrong population or geographical location/ duplicate or repetition |
|  | Planetary Health Annual Meeting 2019.  Anonymous  The Lancet Planetary Health. Conference: Planetary Health Annual Meeting 2019. Stanford University, United States. 3(Supplement 1) (pp S1-S22), 2019. Date of Publication: September 2019.  [Conference Review]  AN: 2002769613 | Not relevant to the main subject/ wrong population or geographical location/ duplicate or repetition |
|  | SMFM 39th Annual Meeting-The Pregnancy Meeting.  Anonymous  American Journal of Obstetrics and Gynecology. Conference: SMFM 39th Annual Meeting-The Pregnancy Meeting. Las Vegas United States. 220(1 Supplement) (no pagination), 2019. Date of Publication: January 2019.  [Conference Review]  AN: 2001540485 | Not relevant to the main subject/ wrong population or geographical location/ duplicate or repetition |
|  | Artificial intelligence using deep learning to screen for referable and vision-threatening diabetic retinopathy in Africa: a clinical validation study.  Bellemo V., Lim Z.W., Lim G., Nguyen Q.D., Xie Y., Yip M.Y.T., Hamzah H., Ho J., Lee X.Q., Hsu W., Lee M.L., Musonda L., Chandran M., Chipalo-Mutati G., Muma M., Tan G.S.W., Sivaprasad S., Menon G., Wong T.Y., Ting D.S.W.  The Lancet Digital Health. 1(1) (pp e35-e44), 2019. Date of Publication: May 2019.  [Article]  AN: 2001857247 | Not relevant to the main subject/ wrong population or geographical location/ duplicate or repetition |
|  | Predictors of poor adherence to hypertension treatment. Les facteurs predictifs de la mauvaise observance au traitement de l'hypertension arterielle <Les facteurs predictifs de la mauvaise observance au traitement de l'hypertension arterielle.>  Omezzine R.G., Akkara A., Koubaa A.A., Sriha A.B., Rdissi A., Amamou K.  Tunisie Medicale. 97(4) (pp 564-571), 2019. Date of Publication: 2019.  [Article]  AN: 2002768345 | Not relevant to the main subject/ wrong population or geographical location/ duplicate or repetition |
|  | Bone mineral density in virologically suppressed people aged 60 years or older with HIV-1 switching from a regimen containing tenofovir disoproxil fumarate to an elvitegravir, cobicistat, emtricitabine, and tenofovir alafenamide single-tablet regimen: a multicentre, open-label, phase 3b, randomised trial.  Maggiolo F., Rizzardini G., Raffi F., Pulido F., Mateo-Garcia M.G., Molina J.-M., Ong E., Shao Y., Piontkowsky D., Das M., McNicholl I., Haubrich R.  The Lancet HIV. 6(10) (pp e655-e666), 2019. Date of Publication: October 2019.  [Article]  AN: 2002986933 | Not relevant to the main subject/ wrong population or geographical location/ duplicate or repetition |
|  | Fabrication and characterization of glimepiride nanosuspension by ultrasonication-assisted precipitation for improvement of oral bioavailability and in vitro alpha-glucosidase inhibition.  Rahim H., Sadiq A., Khan S., Amin F., Ullah R., Shahat A.A., Mahmood H.M.  International Journal of Nanomedicine. 14 (pp 6287-6296), 2019. Date of Publication: 2019.  [Article]  AN: 2002363970 | Not relevant to the main subject/ wrong population or geographical location/ duplicate or repetition |
|  | Community-Driven Priorities in Smartphone Application Development: Leveraging Social Networks to Self-Manage Type 2 Diabetes in a Low-Income African American Neighborhood.  Surkan P.J., Mezzanotte K.S., Sena L.M., Chang L.W., Gittelsohn J., Lagerros Y.T., Quinn C.C., Zachary W.W.  International Journal of Environmental Research and Public Health. 16(15) (no pagination), 2019. Article Number: 2715. Date of Publication: 01 Aug 2019.  [Article]  AN: 2002288061 | Not relevant to the main subject/ wrong population or geographical location/ duplicate or repetition |
|  | Community-and mHealth-based integrated management of diabetes in primary healthcare in Rwanda (D2;Rwanda): The protocol of a mixed-methods study including a cluster randomised controlled trial.  Lygidakis C., Uwizihiwe J.P., Kallestrup P., Bia M., Condo J., Vogele C.  BMJ Open. 9(7) (no pagination), 2019. Article Number: e028427. Date of Publication: 01 Jul 2019.  [Article]  AN: 628694872 | Not relevant to the main subject/ wrong population or geographical location/ duplicate or repetition |
|  | Impact of mobile health applications on self-management in patients with type 2 diabetes mellitus: Protocol of a systematic review.  Bene B.A., O'Connor S., Mastellos N., Majeed A., Fadahunsi K.P., O'Donoghue J.  BMJ Open. 9(6) (no pagination), 2019. Article Number: e025714. Date of Publication: 01 Jun 2019.  [Review]  AN: 628279467 | Not relevant to the main subject/ wrong population or geographical location/ duplicate or repetition |
|  | Popular diabetes apps and the impact of diabetes app use on self-care behaviour: A survey among the digital community of persons with diabetes on social media.  Kebede M.M., Pischke C.R.  Frontiers in Endocrinology. 10(MAR) (no pagination), 2019. Article Number: 135. Date of Publication: 2019.  [Article]  AN: 627929314 | Not relevant to the main subject/ wrong population or geographical location/ duplicate or repetition |
|  | One-way SMS and healthcare outcomes in Africa: Systematic review of randomised trials with meta-analysis.  Linde D.S., Korsholm M., Katanga J., Rasch V., Lundh A., Andersen M.S.  PLoS ONE. 14(6) (no pagination), 2019. Article Number: e0217485. Date of Publication: June 2019.  [Article]  AN: 2002079914 | Not relevant to the main subject/ wrong population or geographical location/ duplicate or repetition |
|  | Hydrogel optical fibers for continuous glucose monitoring.  Elsherif M., Hassan M.U., Yetisen A.K., Butt H.  Biosensors and Bioelectronics. 137 (pp 25-32), 2019. Date of Publication: 15 July 2019.  [Article]  AN: 2001931426 | Not relevant to the main subject/ wrong population or geographical location/ duplicate or repetition |
|  | Combined vildagliptin and memantine treatment downregulates expression of amyloid precursor protein, and total and phosphorylated tau in a rat model of combined Alzheimer's disease and type 2 diabetes.  Khalaf S.S., Hafez M.M., Mehanna E.T., Mesbah N.M., Abo-Elmatty D.M.  Naunyn-Schmiedeberg's Archives of Pharmacology. 392(6) (pp 685-695), 2019. Date of Publication: 04 Jun 2019.  [Article]  AN: 626373943 | Not relevant to the main subject/ wrong population or geographical location/ duplicate or repetition |
|  | Racial and ethnic healthcare disparities in patients undergoing laser lead extraction.  Rodriguez Y., Irizarry F., Carrillo R.G.  International Journal of Cardiology. 286 (pp 181-185), 2019. Date of Publication: 1 July 2019.  [Article]  AN: 2000934186 | Not relevant to the main subject/ wrong population or geographical location/ duplicate or repetition |
|  | Does mobile phone survey method matter? Reliability of computer-assisted telephone interviews and interactive voice response non-communicable diseases risk factor surveys in low and middle income countries.  Pariyo G.W., Greenleaf A.R., Gibson D.G., Ali J., Selig H., Labrique A.B., Kibria G.M.A., Khan I.A., Masanja H., Flora M.S., Ahmed S., Hyder A.A.  PLoS ONE. 14(4) (no pagination), 2019. Article Number: e0214450. Date of Publication: April 2019.  [Article]  AN: 2001810581 | Not relevant to the main subject/ wrong population or geographical location/ duplicate or repetition |
|  | Protocatechuic acid ameliorates neurobehavioral deficits via suppression of oxidative damage, inflammation, caspase-3 and acetylcholinesterase activities in diabetic rats.  Adedara I.A., Fasina O.B., Ayeni M.F., Ajayi O.M., Farombi E.O.  Food and Chemical Toxicology. 125 (pp 170-181), 2019. Date of Publication: March 2019.  [Article]  AN: 2001440664 | Not relevant to the main subject/ wrong population or geographical location/ duplicate or repetition |
|  | High throughput profiling of whole plasma N-glycans in type II diabetes mellitus patients and healthy individuals: A perspective from a Ghanaian population.  Adua E., Memarian E., Russell A., Trbojevic-Akmacic I., Gudelj I., Juric J., Roberts P., Lauc G., Wang W.  Archives of Biochemistry and Biophysics. 661 (pp 10-21), 2019. Date of Publication: January 2019.  [Article]  AN: 2001240693 | Not relevant to the main subject/ wrong population or geographical location/ duplicate or repetition |
|  | Adapting the Diabetes Prevention Program for low and middle-income countries: Protocol for a cluster randomised trial to evaluate 'Lifestyle Africa'.  Catley D., Puoane T., Tsolekile L., Resnicow K., Fleming K., Hurley E.A., Smyth J.M., Vitolins M.Z., Lambert E.V., Levitt N., Goggin K.  BMJ Open. 9(11) (no pagination), 2019. Article Number: e031400. Date of Publication: 01 Nov 2019.  [Article]  AN: 629808128 | Not relevant to the main subject/ wrong population or geographical location/ duplicate or repetition |
|  | Does the use of electronic devices provoke the carpal tunnel syndrome (CTS) symptoms and functional impairment? A cross-sectional study.  Al Shahrani A.S., Albogami S.S., Alabdali A.F., Alohali S.K., Almedbal H.S., Aldossary G.F.  Egyptian Rheumatologist. 41(4) (pp 313-317), 2019. Date of Publication: October 2019.  [Article]  AN: 2001700975 | Not relevant to the main subject/ wrong population or geographical location/ duplicate or repetition |
|  | Results from the first teleglaucoma pilot project in Addis Ababa, Ethiopia.  Giorgis A.T., Alemu A.M., Arora S., Gessesse G.W., Melka F., Woldeyes A., Amin S., Kassam F., Kurji A.K., Damji K.F.  Journal of Glaucoma. 28(8) (pp 701-707), 2019. Date of Publication: 01 Aug 2019.  [Article]  AN: 627661840 | Not relevant to the main subject/ wrong population or geographical location/ duplicate or repetition |
|  | Effect of Nigella Sativa oil versus metformin on glycemic control and biochemical parameters of newly diagnosed type 2 diabetes mellitus patients.  Moustafa H.A.M., El Wakeel L.M., Halawa M.R., Sabri N.A., El-Bahy A.Z., Singab A.N.  Endocrine. 65(2) (pp 286-294), 2019. Date of Publication: 15 Aug 2019.  [Article]  AN: 627955866 | Not relevant to the main subject/ wrong population or geographical location/ duplicate or repetition |
|  | The use of text messaging for improving adherence to anti-diabetic regimen and glycaemic control in low-resource settings of South Africa: A study protocol for a randomised controlled trial.  Owolabi E.O., Goon D.T.  Contemporary Clinical Trials Communications. 15 (no pagination), 2019. Article Number: 100418. Date of Publication: September 2019.  [Article]  AN: 2002371073 | Not relevant to the main subject/ wrong population or geographical location/ duplicate or repetition |
|  | Validation of smartphone-based retinal photography for diabetic retinopathy screening.  Bilong Y., Katte J.-C., Koki G., Kagmeni G., Obama O.P.N., Fofe H.R.N., Mvilongo C., Nkengfack O., Bimbai A.M., Sobngwi E., Mbacham W., Mbanya J.C., Bella L.A., Sharma A.  Ophthalmic Surgery Lasers and Imaging Retina. 50(5) (pp S18-S22), 2019. Date of Publication: 2019.  [Conference Paper]  AN: 2002355720 | Not relevant to the main subject/ wrong population or geographical location/ duplicate or repetition |
|  | BE-SMART (Basal Early Strategies to Maximize HbA1c Reduction with Oral Therapy): Expert Opinion.  Bajaj S., Das A.K., Kalra S., Sahay R., Saboo B., Das S., Shunmugavelu M., Jacob J., Priya G., Khandelwal D., Dutta D., Chawla M., Surana V., Tiwaskar M., Joshi A., Shrestha P.K., Bhattarai J., Bhowmik B., Latt T.S., Aye T.T., Vijayakumar G., Baruah M., Jawad F., Unnikrishnan A.G., Chowdhury S., Pathan M.F., Somasundaram N., Sumanathilaka M., Raza A., Bahendeka S.K., Coetzee A., Ruder S., Ramaiya K., Lamptey R., Bavuma C., Shaikh K., Uloko A., Chaudhary S., Abdela A.A., Akanov Z., Rodriguez-Saldana J., Faradji R., Tiago A., Reja A., Czupryniak L.  Diabetes Therapy. 10(4) (pp 1189-1204), 2019. Date of Publication: 01 Aug 2019.  [Review]  AN: 627827236 | Not relevant to the main subject/ wrong population or geographical location/ duplicate or repetition |
|  | Mobile Health and Technology Usage by Patients in the Diabetes, Nutrition, and Weight Management Clinic at an Urban Academic Medical Center.  Stockman M.-C., Modzelewski K., Steenkamp D.  Diabetes Technology and Therapeutics. 21(7) (pp 400-405), 2019. Date of Publication: July 2019.  [Article]  AN: 628409233 | Not relevant to the main subject/ wrong population or geographical location/ duplicate or repetition |
|  | Diabetes in developing countries.  Misra A., Gopalan H., Jayawardena R., Hills A.P., Soares M., Reza-Albarran A.A., Ramaiya K.L.  Journal of Diabetes. 11(7) (pp 522-539), 2019. Date of Publication: July 2019.  [Review]  AN: 627275210 | Not relevant to the main subject/ wrong population or geographical location/ duplicate or repetition |
|  | Glycemic control and diabetes complications among diabetes mellitus patients attending at University of Gondar Hospital, Northwest Ethiopia.  Fasil A., Biadgo B., Abebe M.  Diabetes, Metabolic Syndrome and Obesity: Targets and Therapy. 12 (pp 75-83), 2019. Date of Publication: 2019.  [Article]  AN: 2001352250 | Not relevant to the main subject/ wrong population or geographical location/ duplicate or repetition |
|  | Comparison of three meta-analytic methods using data from digital interventions on type 2 diabetes.  Kebede M.M., Peters M., Heise T.L., Pischke C.R.  Diabetes, Metabolic Syndrome and Obesity: Targets and Therapy. 12 (pp 59-73), 2019. Date of Publication: 2019.  [Article]  AN: 2001352249 | Not relevant to the main subject/ wrong population or geographical location/ duplicate or repetition |
|  | A community-based diabetes group pilot incorporating a community health worker and photovoice methodology in an urban primary care practice.  Jia J., Quintiliani L., Truong V., Jean C., Branch J., Lasser K.E.  Cogent Medicine. 6(1) (pp 1-14), 2019. Date of Publication: 01 Jan 2019.  [Article]  AN: 626021834 | Not relevant to the main subject/ wrong population or geographical location/ duplicate or repetition |
|  | Implementation of cardiovascular screening in hispanic patient population with ra, sle and psa.  Gandrabur L., Young Kim W., Sen A., Nes D., Ash J., Wasserman A., Sperber K.  Arthritis and Rheumatology. Conference: American College of Rheumatology/Association of Rheumatology Health Professionals Annual Scientific Meeting, ACR/ARHP 2019. Atlanta, GA United States. 71(Supplement 10) (pp 505-507), 2019. Date of Publication: October 2019.  [Conference Abstract]  AN: 633060379 | Not relevant to the main subject/ wrong population or geographical location/ duplicate or repetition |
|  | Business model initiatives to improve access to essential medicines in limited resources countries. a pharmaceutical company's approach.  Nusser H., Salimullah T., Canon V., Shecker N., Hinder R., Stevens R.  American Journal of Tropical Medicine and Hygiene. Conference: 68th Annual Meeting of the American Society of Tropical Medicine and Hygiene, ASTMH 2019. National Harbor, MD United States. 101(5 Supplement) (pp 221), 2019. Date of Publication: November 2019.  [Conference Abstract]  AN: 630644054 | Not relevant to the main subject/ wrong population or geographical location/ duplicate or repetition |
|  | Barriers to follow-up care in an underserved community-based tele-ophthalmology screening program.  Zolot A., Abenoza N., Kim J.E., Medic V., Davis K., Romant J., Castro A., Pachero M.S.  Investigative Ophthalmology and Visual Science. Conference: 2019 Annual Meeting Association for Research in Vision and Ophthalmology, ARVO 2019. Vancouver, BC Canada. 60(9) (no pagination), 2019. Date of Publication: July 2019.  [Conference Abstract]  AN: 629936768 | Not relevant to the main subject/ wrong population or geographical location/ duplicate or repetition |
|  | Efficacy, feasibility and acceptability of mHealth technology (SMS) for promoting glycaemic status and self-management among lowincome earning adults in Eastern Cape, South Africa.  Owolabi E.O., Goon D.T.  Trials. Conference: 5th International Clinical Trials Methodology Conference, ICTMC 2019. Brighton United Kingdom. 20(Supplement 1) (no pagination), 2019. Date of Publication: October 2019.  [Conference Abstract]  AN: 629761089 | Not relevant to the main subject/ wrong population or geographical location/ duplicate or repetition |
|  | The impact of digital health coaching on the diabetes distress scale (DDS).  Allison M., Srivastava U., Burton S.B., Rasulnia M., Patel D.  Diabetes. Conference: 79th Scientific Sessions of the American Diabetes Association, ADA 2019. San Francisco, CA United States. 68(Supplement 1) (no pagination), 2019. Date of Publication: June 2019.  [Conference Abstract]  AN: 629614731 | Not relevant to the main subject/ wrong population or geographical location/ duplicate or repetition |
|  | Assessment of the Rwandan diabetics' needs and expectations to develop their first diabetes self-management Smartphone Application (Kir'App).  Kabeza C.B., Harst L., Schwarz P.E.H., Timpel P.  Diabetologie und Stoffwechsel. Conference: 54. Jahrestagung der Deutsche Diabetes-Gesellschaft, DDG 2019. Berlin Germany. 14(Supplement 1) (pp S9), 2019. Date of Publication: May 2019.  [Conference Abstract]  AN: 629492913 | Not relevant to the main subject/ wrong population or geographical location/ duplicate or repetition |
|  | A mobile personal health record for behavioral health homes: Preliminary results from a randomized trial.  Druss B.  Schizophrenia Bulletin. Conference: 2019 Congress of the Schizophrenia International Research Society, SIRS 2019. Orlando, FL United States. 45(Supplement 2) (pp S134-S135), 2019. Date of Publication: April 2019.  [Conference Abstract]  AN: 629480027 | Not relevant to the main subject/ wrong population or geographical location/ duplicate or repetition |
|  | Comparison of the measurement of the blood pressure in consultation versus home monitoring for the evaluation of the blood pressure targets in the diabetics of type 2.  Benmekki A., Tabti E., Drici Z., Chiali S., Lounici A.  Archives of Cardiovascular Diseases Supplements. Conference: 38es JHTA: 12th International Meeting of the French Society of Hypertension. Paris France. 11(3 Supplement) (pp e362-e363), 2019. Date of Publication: August 2019.  [Conference Abstract]  AN: 2002370515 | Not relevant to the main subject/ wrong population or geographical location/ duplicate or repetition |
|  | Masked hypertension incidence and risk factors in the area of Blida (Algeria).  Bachir Cherif A., Bennouar S., Bouamra A., Temmar M., Bouafia M.  Archives of Cardiovascular Diseases Supplements. Conference: 38es JHTA: 12th International Meeting of the French Society of Hypertension. Paris France. 11(3 Supplement) (pp e361-e362), 2019. Date of Publication: August 2019.  [Conference Abstract]  AN: 2002370495 | Not relevant to the main subject/ wrong population or geographical location/ duplicate or repetition |
|  | Study of effect of therapy of chronic obstructive pulmonary disease on coexistent chronic heart failure.  Rafa S., Aboelhoda A., Khamis A., Swied R.  European Journal of Heart Failure. Conference: Heart Failure 2019 and the World Congress on Acute Heart Failure. Athens Greece. 21(Supplement 1) (pp 421), 2019. Date of Publication: May 2019.  [Conference Abstract]  AN: 628557960 | Not relevant to the main subject/ wrong population or geographical location/ duplicate or repetition |
|  | Measuring medicines adherence in children: A systematic review.  Aldosari M., Conroy S., Oliveira A.  Archives of Disease in Childhood. Conference: 2018 Neonatal and Paediatric Pharmacists Conference. Bristol United Kingdom. 104(7) (no pagination), 2019. Date of Publication: July 2019.  [Conference Abstract]  AN: 628472690 | Not relevant to the main subject/ wrong population or geographical location/ duplicate or repetition |
|  | Update on metabolic syndrome, diabetes management, and HTN guidelines for the addiction psychiatrist.  Gupta R.A., Freitas S.C., Manocha P.  American Journal on Addictions. Conference: 30th Annual Meeting and Scientific Symposium of the American Academy of Addiction Psychiatry, AAAP 2019. San Diego, CA United States. 28(3) (pp 166), 2019. Date of Publication: May 2019.  [Conference Abstract]  AN: 628190917 | Not relevant to the main subject/ wrong population or geographical location/ duplicate or repetition |
|  | Vasoplegia in verapamil overdose.  Watson A., Loberger J., Godsey V., Kong M.  Journal of Investigative Medicine. Conference: Southern Regional Meeting 2019. New Orleans, LA United States. 67(2) (pp 506), 2019. Date of Publication: February 2019.  [Conference Abstract]  AN: 626929514 | Not relevant to the main subject/ wrong population or geographical location/ duplicate or repetition |
|  | Effectiveness of Technologically Enhanced Peer Support in Improving Glycemic Management Among Predominantly African American, Low-Income Adults With Diabetes.  Heisler M., Choi H., Mase R., Long J.A., Reeves P.J.  The Diabetes educator. 45(3) (pp 260-271), 2019. Date of Publication: 01 Jun 2019.  [Article]  AN: 627577712 | Not relevant to the main subject/ wrong population or geographical location/ duplicate or repetition |
|  | The impact of exposure of diabetic rats to 900 MHz electromagnetic radiation emitted from mobile phone antenna on hepatic oxidative stress.  Ismaiil L.A., Joumaa W.H., Moustafa M.E.  Electromagnetic biology and medicine. 38(4) (pp 287-296), 2019. Date of Publication: 2019.  [Article]  AN: 628735791 | Not relevant to the main subject/ wrong population or geographical location/ duplicate or repetition |
|  | Design, synthesis, structure-activity relationships and X-ray structural studies of novel 1-oxopyrimido[4,5-c]quinoline-2-acetic acid derivatives as selective and potent inhibitors of human aldose reductase.  Crespo I., Gimenez-Dejoz J., Porte S., Cousido-Siah A., Mitschler A., Podjarny A., Pratsinis H., Kletsas D., Pares X., Ruiz F.X., Metwally K., Farres J.  European Journal of Medicinal Chemistry. 152 (pp 160-174), 2018. Date of Publication: 25 May 2018.  [Article]  AN: 2000707781 | Not relevant to the main subject/ wrong population or geographical location/ duplicate or repetition |
|  | Adapting the Diabetes Prevention Program for low- and middle-income countries: Preliminary implementation findings from lifestyle Africa.  Catley D., Puoane T., Goggin K., Tsolekile L.P., Resnicow K., Fleming K., Smyth J.M., Hurley E.A., Schlachter S., Vitolins M.Z., Lambert E.V., Hassen M., Muhali K., Schoor R.  Translational Behavioral Medicine. 10(1) (pp 46-54), 2018. Date of Publication: 10 Dec 2018.  [Article]  AN: 631244193 | Not relevant to the main subject/ wrong population or geographical location/ duplicate or repetition |
|  | The Management of Diabetes in Everyday Life (MODEL) program: Development of a tailored text message intervention to improve diabetes self-care activities among underserved African-American adults.  Gatwood J., Shuvo S., Ross A., Riordan C., Smith P., Gutierrez M.L., Coday M., Bailey J.  Translational Behavioral Medicine. 10(1) (pp 204-212), 2018. Date of Publication: 10 Dec 2018.  [Article]  AN: 631244173 | Not relevant to the main subject/ wrong population or geographical location/ duplicate or repetition |
|  | World Congress of Cardiology & Cardiovascular Health 2018.  Anonymous  Global Heart. Conference: World Congress of Cardiology & Cardiovascular Health 2018. Dubai United Arab Emirates. 13(4) (no pagination), 2018. Date of Publication: December 2018.  [Conference Review]  AN: 2001540459 | Not relevant to the main subject/ wrong population or geographical location/ duplicate or repetition |
|  | Comparative analysis for optimizing the modified release tacrolimus (Advagraf) after kidney transplantation: A prospective randomized trial.  Bakr M.A., Nagib A.M., Donia A.F., Denewar A.A., Abu-Elmagd M.M., Abbas M.H., Abdel-Rahman A.M., Mashaly M.E., Elsaftawy M.M., Ghoneim M.A.  Saudi journal of kidney diseases and transplantation : an official publication of the Saudi Center for Organ Transplantation, Saudi Arabia. 29(6) (pp 1267-1273), 2018. Date of Publication: 01 Nov 2018.  [Article]  AN: 625705044 | Not relevant to the main subject/ wrong population or geographical location/ duplicate or repetition |
|  | Philadelphia Telemedicine Glaucoma Detection and Follow-up Study: Ocular Findings at Two Health Centers.  Hark L., Acito M., Adeghate J., Henderer J., Okudolo J., Malik K., Molineaux J., Eburuoh R., Zhan T., Katz L.J.  Journal of health care for the poor and underserved. 29(4) (pp 1400-1415), 2018. Date of Publication: 2018.  [Article]  AN: 625024389 | Not relevant to the main subject/ wrong population or geographical location/ duplicate or repetition |
|  | Eliciting the Impact of Digital Consulting for Young People Living With Long-Term Conditions (LYNC Study): Cognitive Interviews to Assess the Face and Content Validity of Two Patient-Reported Outcome Measures.  Sturt J., Dliwayo T.R., Forjaz V., Hamilton K., Bryce C., Fraser J., Griffiths F.  Journal of medical Internet research. 20(10) (pp e268), 2018. Date of Publication: 11 Oct 2018.  [Article]  AN: 624341878 | Not relevant to the main subject/ wrong population or geographical location/ duplicate or repetition |
|  | A Church-Based Weight Loss Intervention in African American Adults using Text Messages (LEAN Study): Cluster Randomized Controlled Trial.  Newton R.L., Carter L.A., Johnson W., Zhang D., Larrivee S., Kennedy B.M., Harris M., Hsia D.S.  Journal of medical Internet research. 20(8) (pp e256), 2018. Date of Publication: 24 Aug 2018.  [Article]  AN: 627790013 | Not relevant to the main subject/ wrong population or geographical location/ duplicate or repetition |
|  | Clinical Inertia in a Randomized Trial of Telemedicine-Based Chronic Disease Management: Lessons Learned.  Barton A.B., Okorodudu D.E., Bosworth H.B., Crowley M.J.  Telemedicine journal and e-health : the official journal of the American Telemedicine Association. 24(10) (pp 742-748), 2018. Date of Publication: 01 Oct 2018.  [Article]  AN: 624451058 | Not relevant to the main subject/ wrong population or geographical location/ duplicate or repetition |
|  | Community-based screening for cardiovascular risk using a novel mHealth tool in rural Kenya.  Mannik J., Figol A., Churchill V., Aw J., Francis S., Karino E., Chesire J.K., Opot D., Ochieng B., Hawkes M.T.  Journal of innovation in health informatics. 25(3) (pp 176-182), 2018. Date of Publication: 31 Oct 2018.  [Article]  AN: 624833286 | Not relevant to the main subject/ wrong population or geographical location/ duplicate or repetition |
|  | Telemedicine cardiovascular risk reduction in veterans: The CITIES trial.  Bosworth H.B., Olsen M.K., McCant F., Stechuchak K.M., Danus S., Crowley M.J., Goldstein K.M., Zullig L.L., Oddone E.Z.  American Heart Journal. 199 (pp 122-129), 2018. Date of Publication: May 2018.  [Article]  AN: 621165044 | Not relevant to the main subject/ wrong population or geographical location/ duplicate or repetition |
|  | Cost-Effectiveness of the Interventions to Avoid Complications and Management in Diabetes Mellitus: A Narrative Review from South-East Asian Perspective.  Jamshed S.Q., Bhagavathula A.S., Al-Shami A.K.  Romanian Journal of Diabetes, Nutrition and Metabolic Diseases. 25(2) (pp 197-208), 2018. Date of Publication: 01 Jun 2018.  [Review]  AN: 623320991 | Not relevant to the main subject/ wrong population or geographical location/ duplicate or repetition |
|  | From HIV prevention to non-communicable disease health promotion efforts in sub-Saharan Africa: A Narrative Review.  Juma K., Reid M., Roy M., Vorkoper S., Temu T.M., Levitt N.S., Oladepo O., Zakus D., Yonga G.  AIDS. 32(Supplement 1) (pp S63-S73), 2018. Date of Publication: 01 Jul 2018.  [Review]  AN: 623108249 | Not relevant to the main subject/ wrong population or geographical location/ duplicate or repetition |
|  | Protocol of economic evaluation and equity impact analysis of mHealth and community groups for prevention and control of diabetes in rural Bangladesh in a three-arm cluster randomised controlled trial.  Haghparast-Bidgoli H., Shaha S.K., Kuddus A., Chowdhury M.A.R., Jennings H., Ahmed N., Morrison J., Akter K., Nahar B., Nahar T., King C., Skordis-Worrall J., Batura N., Khan J.A., Mansaray A., Hunter R., Khan A.K.A., Costello A., Azad K., Fottrell E.  BMJ Open. 8(8) (no pagination), 2018. Article Number: e022035. Date of Publication: 01 Aug 2018.  [Article]  AN: 623793659 | Not relevant to the main subject/ wrong population or geographical location/ duplicate or repetition |
|  | Rubber stamp templates for improving clinical documentation: A paper-based, m-Health approach for quality improvement in low-resource settings.  Kleczka B., Musiega A., Rabut G., Wekesa P., Mwaniki P., Marx M., Kumar P.  International Journal of Medical Informatics. 114 (pp 121-129), 2018. Date of Publication: June 2018.  [Article]  AN: 619004021 | Not relevant to the main subject/ wrong population or geographical location/ duplicate or repetition |
|  | Pregnant women's experiences with an integrated diagnostic and decision support device for antenatal care in Ghana.  Abejirinde I.-O.O., Douwes R., Bardaji A., Abugnaba-Abanga R., Zweekhorst M., van Roosmalen J., De Brouwere V.  BMC Pregnancy and Childbirth. 18(1) (no pagination), 2018. Article Number: 209. Date of Publication: 05 Jun 2018.  [Article]  AN: 622427823 | Not relevant to the main subject/ wrong population or geographical location/ duplicate or repetition |
|  | Effectiveness of Digital Interventions for Improving Glycemic Control in Persons with Poorly Controlled Type 2 Diabetes: A Systematic Review, Meta-analysis, and Meta-regression Analysis.  Kebede M.M., Zeeb H., Peters M., Heise T.L., Pischke C.R.  Diabetes Technology and Therapeutics. 20(11) (pp 767-782), 2018. Date of Publication: November 2018.  [Article]  AN: 624870315 | Not relevant to the main subject/ wrong population or geographical location/ duplicate or repetition |
|  | Physical disability and diabetes mellitus; qualitative exploration of patients' perception and behavior.  Gillani S.W., Sulaiman S.A.S., Abdul M.I.M., Saad S.Y.  Current Diabetes Reviews. 14(5) (pp 472-480), 2018. Date of Publication: 2018.  [Article]  AN: 623538582 | Not relevant to the main subject/ wrong population or geographical location/ duplicate or repetition |
|  | Clinical relevance of smartphone apps for diabetes management: A global overview.  Huang Z., Soljak M., Boehm B.O., Car J.  Diabetes/Metabolism Research and Reviews. 34(4) (no pagination), 2018. Article Number: e2990. Date of Publication: May 2018.  [Article]  AN: 621996105 | Not relevant to the main subject/ wrong population or geographical location/ duplicate or repetition |
|  | Prevalence of diabetic retinopathy and visual impairment in patients with diabetes mellitus in Zambia through the implementation of a mobile diabetic retinopathy screening project in the Copperbelt province: A cross-sectional study.  Lewis A.D., Hogg R.E., Chandran M., Musonda L., North L., Chakravarthy U., Sivaprasad S., Menon G.  Eye (Basingstoke). 32(7) (pp 1201-1208), 2018. Date of Publication: 01 Jul 2018.  [Article]  AN: 621033634 | Not relevant to the main subject/ wrong population or geographical location/ duplicate or repetition |
|  | Effect of smart electronic devices on micra leadless pacemakers.  Peddareddy L., Merchant F.M., El-Chami M.F., Smith P.  Heart Rhythm. Conference: 39th Annual Scientific Sessions of the Heart Rhythm Society, Heart Rhythm 2018. Boston, MA United States. 15(5 Supplement 1) (pp S296), 2018. Date of Publication: May 2018.  [Conference Abstract]  AN: 622469432 | Not relevant to the main subject/ wrong population or geographical location/ duplicate or repetition |
|  | Gastroretentive Microsponge as a Promising Tool for Prolonging the Release of Mitiglinide Calcium in Type-2 Diabetes Mellitus: Optimization and Pharmacokinetics Study.  Mahmoud D.B.E.D., Shukr M.H., ElMeshad A.N.  AAPS PharmSciTech. 19(6) (pp 2519-2532), 2018. Date of Publication: 01 Aug 2018.  [Article]  AN: 622557355 | Not relevant to the main subject/ wrong population or geographical location/ duplicate or repetition |
|  | A mobile computer aided system for optic nerve head detection.  Elloumi Y., Akil M., Kehtarnavaz N.  Computer Methods and Programs in Biomedicine. 162 (pp 139-148), 2018. Date of Publication: August 2018.  [Article]  AN: 2000780021 | Not relevant to the main subject/ wrong population or geographical location/ duplicate or repetition |
|  | Affordability and availability of off-patent drugs in the United States - The case for importing from abroad: Observational study.  Gupta R., Bollyky T.J., Cohen M., Ross J.S., Kesselheim A.S.  BMJ (Online). 360 (no pagination), 2018. Article Number: k831. Date of Publication: 2018.  [Article]  AN: 621295446 | Not relevant to the main subject/ wrong population or geographical location/ duplicate or repetition |
|  | SMS-based intervention in type 2 diabetes: Clinical trial in Senegal.  Wargny M., Kleinebreil L., Diop S.N., Ndour-Mbaye M., Ba M., Balkau B., Simon D.  BMJ Innovations. 4(3) (pp 142-146), 2018. Date of Publication: 01 Jul 2018.  [Article]  AN: 622561979 | Included |
|  | Sensitive spectrofluorimetric methods for determination of sitagliptin phosphate, dipeptidyl peptidase-4 inhibitor, in pharmaceutical tablets and spiked human urine.  Ali M.F.B., Atia N.N.  Current Pharmaceutical Analysis. 14(5) (pp 483-490), 2018. Date of Publication: 2018.  [Article]  AN: 623002994 | Not relevant to the main subject/ wrong population or geographical location/ duplicate or repetition |
|  | Development of a health dialogue model for patients with diabetes: A complex intervention in a low-/middle income country.  Reid M., Walsh C., Raubenheimer J., Bradshaw T., Pienaar M., Hassan C., Nyoni C., Le Roux M.  International Journal of Africa Nursing Sciences. 8 (pp 122-131), 2018. Date of Publication: 01 Jan 2018.  [Article]  AN: 2000764479 | Not relevant to the main subject/ wrong population or geographical location/ duplicate or repetition |
|  | Assessment of self-management in patients with diabetes using the novel LMC Skills, Confidence and Preparedness Index (SCPI).  Aronson R., Brown R.E., Jiandani D., Walker A., Orzech N., Mbuagbaw L.  Diabetes Research and Clinical Practice. 137 (pp 128-136), 2018. Date of Publication: March 2018.  [Article]  AN: 620514668 | Not relevant to the main subject/ wrong population or geographical location/ duplicate or repetition |
|  | Clinical pharmacokinetic study for the effect of glimepiride matrix tablets developed by quality by design concept.  Ahmed T.A., Suhail M.A.A., Hosny K.M., Abd-Allah F.I.  Drug Development and Industrial Pharmacy. 44(1) (pp 66-81), 2018. Date of Publication: 02 Jan 2018.  [Article]  AN: 618300595 | Not relevant to the main subject/ wrong population or geographical location/ duplicate or repetition |
|  | Effect of homocysteine-lowering therapy on diabetic nephropathy in children and adolescents with type 1 diabetes.  Elbarbary N., Ismail E.A.R., Zaki M.A., Ibrahim M.Z., El-Hamamsy M.  Hormone Research in Paediatrics. Conference: 57th Annual Meeting of the European Society for Paediatric Endocrinology, ESPE 2018. Athens Greece. 90(Supplement 1) (pp 66), 2018. Date of Publication: September 2018.  [Conference Abstract]  AN: 630602636 | Not relevant to the main subject/ wrong population or geographical location/ duplicate or repetition |
|  | SIG (special interest group)-ENDOPED/RUTE (Brazil): Seven years integrating pediatric endocrinology centers throughout the country.  Arrais R.F., Alves C.A.D., Junior G.G., Castro L.C.G., Filho G.M.G., Kopacek C., Damiani D., Del Roio R., Junior L., Punales M.K.  Hormone Research in Paediatrics. Conference: 57th Annual Meeting of the European Society for Paediatric Endocrinology, ESPE 2018. Athens Greece. 90(Supplement 1) (pp 537), 2018. Date of Publication: September 2018.  [Conference Abstract]  AN: 630604728 | Not relevant to the main subject/ wrong population or geographical location/ duplicate or repetition |
|  | Philadelphia telemedicine glaucoma detection and follow-up study: Adherence to community eye exam appointments.  Hark L.A., Katz L.J., Myers J.S., Haller J.A.  Investigative Ophthalmology and Visual Science. Conference: 2018 Annual Meeting of the Association for Research in Vision and Ophthalmology, ARVO 2018. Honolulu, HI United States. 59(9) (no pagination), 2018. Date of Publication: July 2018.  [Conference Abstract]  AN: 628582559 | Not relevant to the main subject/ wrong population or geographical location/ duplicate or repetition |
|  | Philadelphia telemedicine glaucoma detection and follow-up study: Interphysician agreement for screening images.  Kolomeyer N.N., Hark L.A., Katz L.J., Myers J.S., Lee D., Razeghinejad M.R., Leite S., Rahmatnejad K., Zhan T., Leiby B., Hegarty S., Lee P.P.  Investigative Ophthalmology and Visual Science. Conference: 2018 Annual Meeting of the Association for Research in Vision and Ophthalmology, ARVO 2018. Honolulu, HI United States. 59(9) (no pagination), 2018. Date of Publication: July 2018.  [Conference Abstract]  AN: 628422389 | Not relevant to the main subject/ wrong population or geographical location/ duplicate or repetition |
|  | Change in knowledge of tobacco use and secondhand smoke exposure among health workers in Uganda.  Mamudu H., Namusisi K., Bazeyo W., Olando Y., Surabhi J., Makumbi F., Pack R., Rutebemberwa E.  Tobacco Induced Diseases. Conference: 17th World Conference on Tobacco or Health. Cape Town South Africa. 16(Supplement 1) (pp 244), 2018. Date of Publication: 2018.  [Conference Abstract]  AN: 627399898 | Not relevant to the main subject/ wrong population or geographical location/ duplicate or repetition |
|  | Telemedicine cardiovascular screening program in low-income settings by smartphone based technology.  Maurizi N., Peschiera S., Ochola E., Fumagalli C., Rughetti R., Avvantaggiato F., Tamba M., Corradi F., Marchionni N., Esposito C., Parigi G.B., Perlini S., Cecchi F., Kansiime J., Olivotto I.  European Heart Journal. Conference: European Society of Cardiology Congress, ESC 2018. Munich Germany. 39(Supplement 1) (pp 860-861), 2018. Date of Publication: August 2018.  [Conference Abstract]  AN: 627252082 | Not relevant to the main subject/ wrong population or geographical location/ duplicate or repetition |
|  | A randomized trial using mobile short-text messaging to improve cardiovascular risk profile in poorly controlled diabetes in kenya.  Soin G., Kunyiha N., Shah J., Patel K., Arisi C., Njenga E., Jeilan M., Sorathia S., Ngunga L.M., Barasa A.L.  Circulation. Conference: 2018 American Heart Association Scientific Sessions. Chicago, IL United States. 138(Supplement 1) (no pagination), 2018. Date of Publication: November 2018.  [Conference Abstract]  AN: 626955750 | No full article |
|  | Short-term efficacy of the combination of perindopril-amlodipine versus perindopril-indapamide on blood pressure control in a population of type 2 diabetics in sub-saharan africa.  Florence K.S.M.  Journal of Hypertension. Conference: 27th Scientific Meeting of the International Society of Hypertension, ISH 2018. Beijing China. 36(Supplement 3) (pp e243), 2018. Date of Publication: October 2018.  [Conference Abstract]  AN: 625307797 | Not relevant to the main subject/ wrong population or geographical location/ duplicate or repetition |
|  | "I'M BEGINNING TO REMEMBER": THE SHARP STUDY'S NEIGHBORHOOD APPROACH TO AFRICAN AMERICAN BRAIN HEALTH.  Croff R., Francois E., Towns J., Pruitt A., Kaye J.A.  Alzheimer's and Dementia. Conference: Alzheimer's Association International Conference 2018. Chicago United States. 14(7 Supplement) (pp P590), 2018. Date of Publication: July 2018.  [Conference Abstract]  AN: 2001205591 | Not relevant to the main subject/ wrong population or geographical location/ duplicate or repetition |
|  | The influences of ethnicity on the quality of type 2 diabetes care in Norwegian general practice.  Tran A.T., Gjelsvik B., Berg T.J., Nokleby K., Cooper J.G., Bakke A., Claudi T., Sandberg S., Thue G., Jenum A.K.  Diabetologia. Conference: 54th Annual Meeting of the European Association for the Study Diabetes, EASD 2018. Berlin Germany. 61(Supplement 1) (pp S421-S422), 2018. Date of Publication: October 2018.  [Conference Abstract]  AN: 624031216 | Not relevant to the main subject/ wrong population or geographical location/ duplicate or repetition |
|  | Baseline health utility scores in subjects with suspected ocular disease: An interim analysis of the philadelphia telemedicine glaucoma detection and follow-up study.  Mehta R., Prioli K.M., Hark L., Katz L.J., Myers J.S., Pizzi L.T.  Value in Health. Conference: 23rd Annual Meeting of the International Society for Pharmacoeconomics and Outcomes Research, ISPOR 2018. Baltimore, MD United States. 21(Supplement 1) (pp S126), 2018. Date of Publication: May 2018.  [Conference Abstract]  AN: 623583973 | Not relevant to the main subject/ wrong population or geographical location/ duplicate or repetition |
|  | Virtual education for older adults with type 2 diabetes during transitions.  Whitehouse C.R., Long J.A., Mcleer Maloney L., Horowitz D.A., Bowles K.  Diabetes. Conference: 78th Scientific Sessions of the American Diabetes Association, ADA 2018. Orlando, FL United States. 67(Supplement 1) (pp A185), 2018. Date of Publication: July 2018.  [Conference Abstract]  AN: 623566928 | Not relevant to the main subject/ wrong population or geographical location/ duplicate or repetition |
|  | Effect of remote glucose monitoring utilizing computerized insulin dose adjustment algorithms on A1c levels-a preliminary report.  Davidson M.B., Davidson J.  Diabetes. Conference: 78th Scientific Sessions of the American Diabetes Association, ADA 2018. Orlando, FL United States. 67(Supplement 1) (pp A234), 2018. Date of Publication: July 2018.  [Conference Abstract]  AN: 623566559 | Not relevant to the main subject/ wrong population or geographical location/ duplicate or repetition |
|  | Hemoglobin A1c point of care clinical disparity.  Moll G.W.  Endocrine Reviews. Conference: 100th Annual Meeting of the Endocrine Society, ENDO 2018. Chicago, IL United States. 39(2 Supplement 1) (no pagination), 2018. Date of Publication: April 2018.  [Conference Abstract]  AN: 623114511 | Not relevant to the main subject/ wrong population or geographical location/ duplicate or repetition |
|  | Dilated cardiomyopathy with severe left ventricular dysfunction and right atrial thrombi secondary to thyrotoxicosis.  Antit S., Slama I., Chenik S., Belakhel S., Mestiri A., Boussabeh I., Thameur M., Zakhama L., Benyoussef S.  European Journal of Heart Failure. Conference: Heart Failure 2018 and the 5th World Congress on Acute Heart Failure. Vienna Austria. 20(Supplement 1) (pp 169), 2018. Date of Publication: May 2018.  [Conference Abstract]  AN: 622651044 | Not relevant to the main subject/ wrong population or geographical location/ duplicate or repetition |
|  | Corrie health: Re-engineering discharge and recovery from acute myocardial infarctions.  Wang J., Marvel F.A., Spaulding E.M., Lee M., Fashanu O., Xun H., Shah L., Yang W.E., Merali F., Demo R., Martin S.S.  Journal of General Internal Medicine. Conference: 41st Annual Meeting of the Society of General Internal Medicine, SGIM 2018. Denver, CO United States. 33(2 Supplement 1) (pp 777-778), 2018. Date of Publication: 2018.  [Conference Abstract]  AN: 622330408 | Not relevant to the main subject/ wrong population or geographical location/ duplicate or repetition |
|  | Sofosbuvir/Ledipasvir: Efficacy and tolerance in HCV positive patients naive or pre-treated in Cameroun.  Tchamgoue S., Leundji H., Tagny-Sartre M., Tzeuton C., Esmat G.  Journal of Hepatology. Conference: 53rd Annual Meeting of the European Association for the Study of the Liver, International Liver Congress 2018. Paris France. 68(Supplement 1) (pp S294), 2018. Date of Publication: April 2018.  [Conference Abstract]  AN: 621860282 | Not relevant to the main subject/ wrong population or geographical location/ duplicate or repetition |
|  | Feasibility of cardiovascular screening in low-income settings using smartphone-based technologies.  Fumagalli C., Maurizi N., O'konu S., Rughetti R., Avvantaggiato F., Tamba M., Targetti M., Passantino S., Arretini A., Tomberli A., Baldini K., Barlocco F., Marchionni N., Cecchi F., Olivotto I.  Europace. Conference: 1st Annual Conference of the European Heart Rhythm Association, EHRA 2018. Barcelona Spain. 20(Supplement 1) (pp i24-i25), 2018. Date of Publication: March 2018.  [Conference Abstract]  AN: 621570195 | Not relevant to the main subject/ wrong population or geographical location/ duplicate or repetition |
|  | Proposing a risk-factor based clinical app to predict outcomes following initial hospitalization in patients with multiple sclerosis.  Sharma K., Fattal D., Kamholz J., Bittner F.  Multiple Sclerosis Journal. Conference: 3rd Annual Americas Committee for Treatment and Research in Multiple Sclerosis Forum, ACTRIMS 2018. San Diego, CA United States. 24(1 Supplement 1) (pp 123), 2018. Date of Publication: February 2018.  [Conference Abstract]  AN: 621547961 | Not relevant to the main subject/ wrong population or geographical location/ duplicate or repetition |
|  | Different resolution techniques for management of overlapped spectra: Application for the determination of novel co-formulated hypoglycemic drugs in their combined pharmaceutical dosage form.  Moussa B.A., Mahrouse M.A., Fawzy M.G.  Spectrochimica acta. Part A, Molecular and biomolecular spectroscopy. 205 (pp 235-242), 2018. Date of Publication: 05 Dec 2018.  [Article]  AN: 625605347 | Not relevant to the main subject/ wrong population or geographical location/ duplicate or repetition |
|  | Study protocol: The Technology-Enhanced Coaching (TEC) program to improve diabetes outcomes - A randomized controlled trial.  Heisler M., Mase R., Brown B., Wilson S., Reeves P.J.  Contemporary Clinical Trials. 55 (pp 24-33), 2017. Date of Publication: 01 Apr 2017.  [Article]  AN: 614279504 | Not relevant to the main subject/ wrong population or geographical location/ duplicate or repetition |
|  | Effectiveness of behavioral change techniques employed in eHealth interventions designed to improve glycemic control in persons with poorly controlled type 2 diabetes: a systematic review and meta-analysis protocol.  Kebede M., Christianson L., Khan Z., Heise T.L., Pischke C.R.  Systematic reviews. 6(1) (pp 211), 2017. Article Number: 211. Date of Publication: 24 Oct 2017.  [Article]  AN: 623256283 | Not relevant to the main subject/ wrong population or geographical location/ duplicate or repetition |
|  | A PLGA-reinforced PEG in situ gel formulation for improved sustainability of hypoglycaemic activity of glimepiride in streptozotocin-induced diabetic rats.  Ahmed O.A.A., El-Say K.M., Alahdal A.M.  Scientific reports. 7(1) (pp 16384), 2017. Date of Publication: 27 Nov 2017.  [Article]  AN: 628259460 | Not relevant to the main subject/ wrong population or geographical location/ duplicate or repetition |
|  | Retinal imaging with smartphone.  Ademola-Popoola D.S., Olatunji V.A.  Nigerian journal of clinical practice. 20(3) (pp 341-345), 2017. Date of Publication: 01 Mar 2017.  [Article]  AN: 628457175 | Not relevant to the main subject/ wrong population or geographical location/ duplicate or repetition |
|  | The impact of amorphisation and spheronization techniques on the improved in vitro & in vivo performance of glimepiride tablets.  Makar R.R., Latif R., Hosni E.A., El Gazayerly O.N.  Advanced Pharmaceutical Bulletin. 7(4) (pp 557-567), 2017. Date of Publication: 2017.  [Article]  AN: 621107272 | Not relevant to the main subject/ wrong population or geographical location/ duplicate or repetition |
|  | Efficacy and safety of a combination of red yeast rice and olive extract in hypercholesterolemic patients with and without statin-associated myalgia.  Tshongo Muhindo C., Ahn S.A., Rousseau M.F., Dierckxsens Y., Hermans M.P.  Complementary Therapies in Medicine. 35 (pp 140-144), 2017. Date of Publication: December 2017.  [Article]  AN: 619123844 | Not relevant to the main subject/ wrong population or geographical location/ duplicate or repetition |
|  | Protective effects of asiatic acid in a spontaneous type 2 diabetic mouse model.  Sun W., Xu G., Luo G., Wu L., Hou Y., Guo X., Zhou J., Xu T., Qin L., Fan Y., Han L., Matsabisa M., Ma X., Liu T.  Molecular Medicine Reports. 16(2) (pp 1333-1339), 2017. Date of Publication: August 2017.  [Article]  AN: 617296573 | Not relevant to the main subject/ wrong population or geographical location/ duplicate or repetition |
|  | Telephone-Delivered Behavioral Skills Intervention for African American Adults with Type 2 Diabetes: A Randomized Controlled Trial.  Egede L.E., Williams J.S., Voronca D.C., Gebregziabher M., Lynch C.P.  Journal of General Internal Medicine. 32(7) (pp 775-782), 2017. Date of Publication: 01 Jul 2017.  [Article]  AN: 614978495 | Not relevant to the main subject/ wrong population or geographical location/ duplicate or repetition |
|  | Preparation and in vitro/in vivo evaluation of metformin hydrochloride rectal dosage forms for treatment of patients with type II diabetes.  Zaghloul A.-A., Lila A., Abd-Allah F., Nada A.  Journal of Drug Targeting. 25(5) (pp 463-470), 2017. Date of Publication: 28 May 2017.  [Article]  AN: 614212732 | Not relevant to the main subject/ wrong population or geographical location/ duplicate or repetition |
|  | A cluster-randomized trial to estimate the effect of mobile screening and treatment feedback on HbA1c and diabetes-related complications in Tshwane primary health care clinics, South Africa.  Webb E.M., Rheeder P.  Primary Care Diabetes. 11(6) (pp 546-554), 2017. Date of Publication: December 2017.  [Article]  AN: 617182635 | Not relevant to the main subject/ wrong population or geographical location/ duplicate or repetition |
|  | Feasibility of Using Mobile ECG Recording Technology to Detect Atrial Fibrillation in Low-Resource Settings.  Evans G.F., Shirk A., Muturi P., Soliman E.Z.  Global Heart. 12(4) (pp 285-289), 2017. Date of Publication: December 2017.  [Article]  AN: 616482298 | Not relevant to the main subject/ wrong population or geographical location/ duplicate or repetition |
|  | Single Anastomosis Sleeve Ileal Bypass: New Step in the Evolution of Bariatric Surgeries.  Salama T.M.S., Sabry K., Ghamrini Y.E.  Journal of Investigative Surgery. 30(5) (pp 291-296), 2017. Date of Publication: 03 Sep 2017.  [Article]  AN: 612884945 | Not relevant to the main subject/ wrong population or geographical location/ duplicate or repetition |
|  | Matrix tablet containing quaternary inclusion complex of domperidone for treatment of diabetic gastroparesis.  Ghorpade V.S., Mali K.K., Dias R.J., Havaldar V.D., Raut G.S.  Indian Journal of Pharmaceutical Education and Research. 51(4 Supplement) (pp S588-S600), 2017. Date of Publication: October-December 2017.  [Article]  AN: 620640420 | Not relevant to the main subject/ wrong population or geographical location/ duplicate or repetition |
|  | A mini-review of spectrophotometric and chromatographic analysis and bioanalysis of selected recently approved anti-diabetic combinations.  Abdel-Ghany M.F., Ayad M.F., Tadros M.M.  Research Journal of Pharmacy and Technology. 10(9) (pp 3161-3172), 2017. Date of Publication: September 2017.  [Short Survey]  AN: 619085736 | Not relevant to the main subject/ wrong population or geographical location/ duplicate or repetition |
|  | Case mix of patients managed in the resuscitation area of a district-level public hospital in Cape Town. Eventail des patients traites dans le service de reanimation d'un hopital public de district au Cap, en Afrique du Sud <Eventail des patients traites dans le service de reanimation d'un hopital public de district au Cap, en Afrique du Sud.>  Hunter L.D., Lahri S., van Hoving D.J.  African Journal of Emergency Medicine. 7(1) (pp 19-23), 2017. Date of Publication: 01 Mar 2017.  [Article]  AN: 614255704 | Not relevant to the main subject/ wrong population or geographical location/ duplicate or repetition |
|  | Evaluation of diabetic retinal screening and factors for ophthalmology referral in a telemedicine network.  Jani P.D., Forbes L., Choudhury A., Preisser J.S., Viera A.J., Garg S.  JAMA Ophthalmology. 135(7) (pp 706-714), 2017. Date of Publication: July 2017.  [Article]  AN: 617382404  IMPORTANCE: | Not relevant to the main subject/ wrong population or geographical location/ duplicate or repetition |
|  | Phospholipid complex enriched micelles: A novel drug delivery approach for promoting the antidiabetic effect of repaglinide.  Kassem A.A., Abd El-Alim S.H., Basha M., Salama A.  European Journal of Pharmaceutical Sciences. 99 (pp 75-84), 2017. Date of Publication: 01 Mar 2017.  [Article]  AN: 613707514 | Not relevant to the main subject/ wrong population or geographical location/ duplicate or repetition |
|  | Evaluating the feasibility and uptake of a community-led HIV testing and multi-disease health campaign in rural Uganda.  Kabami J., Chamie G., Kwarisiima D., Biira E., Ssebutinde P., Petersen M., Charlebois E.D., Kamya M.R., Havlir D.V., Clark T.D.  Journal of the International AIDS Society. 20(1) (no pagination), 2017. Article Number: 21514. Date of Publication: 2017.  [Article]  AN: 619282073 | Not relevant to the main subject/ wrong population or geographical location/ duplicate or repetition |
|  | High rates of viral suppression in adults and children with high CD4+ counts using a streamlined ART delivery model in the SEARCH trial in rural Uganda and Kenya.  Kwarisiima D., Kamya M.R., Owaraganise A., Mwangwa F., Byonanebye D.M., Ayieko J., Plenty A., Black D., Clark T.D., Nzarubara B., Snyman K., Brown L., Bukusi E., Cohen C.R., Geng E.H., Charlebois E.D., Ruel T.D., Petersen M.L., Havlir D., Jain V.  Journal of the International AIDS Society. 20(Supplement 4) (pp 58-67), 2017. Date of Publication: 21 Jul 2017.  [Article]  AN: 617457928 | Not relevant to the main subject/ wrong population or geographical location/ duplicate or repetition |
|  | Using Mobile Health (mHealth) Technology in the Management of Diabetes Mellitus, Physical Inactivity, and Smoking.  Rehman H., Kamal A.K., Sayani S., Morris P.B., Merchant A.T., Virani S.S.  Current Atherosclerosis Reports. 19(4) (no pagination), 2017. Article Number: 16. Date of Publication: 01 Apr 2017.  [Review]  AN: 614583228 | Not relevant to the main subject/ wrong population or geographical location/ duplicate or repetition |
|  | A remarkable case of late-onset biatrial bacterial endocarditis of the Gore HELEX septal occluder.  Thiagaraj A.K., Bloomingdale R., Telila T., Afonso L.C.  Journal of Cardiology Cases. 16(6) (pp 202-204), 2017. Date of Publication: December 2017.  [Article]  AN: 618325012 | Not relevant to the main subject/ wrong population or geographical location/ duplicate or repetition |
|  | Philadelphia telemedicine glaucoma detection and follow-up study: methods and visit 1 results.  Hark L.A., Katz L.J., Waisbourd M., Myers J.S., Johnson D., Fudemberg S.S., Mantravadi A., Henderer J.D., Bui T.D., Lee J., Haller J.A.  Investigative Ophthalmology and Visual Science. Conference: 2017 Annual Meeting of the Association for Research in Vision and Ophthalmology, ARVO 2017. Baltimore, MD United States. 58(8) (no pagination), 2017. Date of Publication: June 2017.  [Conference Abstract]  AN: 621491061 | Not relevant to the main subject/ wrong population or geographical location/ duplicate or repetition |
|  | Philadelphia telemedicine glaucoma detection and follow-up study: visit 1 and visit 2 satisfaction survey results.  Brodowski C.E., Johnson D., Sapru S., Hark I.A., Myers J.S., Fudemberg S., Mantravadi A., Henderer J., Doyle V., Molineaux J., Divers M., Burns C., Haller J.A., Katz L.J.  Investigative Ophthalmology and Visual Science. Conference: 2017 Annual Meeting of the Association for Research in Vision and Ophthalmology, ARVO 2017. Baltimore, MD United States. 58(8) (no pagination), 2017. Date of Publication: June 2017.  [Conference Abstract]  AN: 621490849 | Not relevant to the main subject/ wrong population or geographical location/ duplicate or repetition |
|  | Philadelphia telemedicine glaucoma detection and follow-up study: Comparison of ocular outcomes at two health centers.  Okudolo J., Hark L.A., Katz L.J., Acito M., DeVirgilio T., Molineaux J., Mazen M., Henderer J., Doyle V., Johnson D., Divers M., Burns C., Haller J.A.  Investigative Ophthalmology and Visual Science. Conference: 2017 Annual Meeting of the Association for Research in Vision and Ophthalmology, ARVO 2017. Baltimore, MD United States. 58(8) (no pagination), 2017. Date of Publication: June 2017.  [Conference Abstract]  AN: 621488261 | Not relevant to the main subject/ wrong population or geographical location/ duplicate or repetition |
|  | Philadelphia telemedicine glaucoma detection and follow-up study: Diagnostic positive predictive value between visit 1 and visit 2.  Ines A., Rahmatnejad K., Hark L.A., Katz L.J., Waisbourd M., Myers J.S., Leiby B.T., Fudemberg S., Mantravadi A., Doyle V., Johnson D., Molineaux J., Divers M., Burns C., Haller J.A.  Investigative Ophthalmology and Visual Science. Conference: 2017 Annual Meeting of the Association for Research in Vision and Ophthalmology, ARVO 2017. Baltimore, MD United States. 58(8) (no pagination), 2017. Date of Publication: June 2017.  [Conference Abstract]  AN: 621487665 | Not relevant to the main subject/ wrong population or geographical location/ duplicate or repetition |
|  | 2017 North American Forum on Family Planning Scientific Abstracts.  Anonymous  Contraception. Conference: 2017 North American Forum on Family Planning. Atlanta, GA United States. 96(4) (no pagination), 2017. Date of Publication: October 2017.  [Conference Review]  AN: 621355723 | Not relevant to the main subject/ wrong population or geographical location/ duplicate or repetition |
|  | How the ajinomoto foundation approaches the challenges of nutrition security.  Kuriwaki K.  Annals of Nutrition and Metabolism. Conference: 21st International Congress of Nutrition, ICN 2017. Buenos Aires Argentina. 71(Supplement 2) (pp 1362-1363), 2017. Date of Publication: 2017.  [Conference Abstract]  AN: 619277261 | Not relevant to the main subject/ wrong population or geographical location/ duplicate or repetition |
|  | Nutrition screening for over nutrition in adults aged 18-49 years in Urban City of Kampala, Uganda.  Ssenyondo M., Ainomugisha P., Nyakake L.  Annals of Nutrition and Metabolism. Conference: 21st International Congress of Nutrition, ICN 2017. Buenos Aires Argentina. 71(Supplement 2) (pp 929-930), 2017. Date of Publication: 2017.  [Conference Abstract]  AN: 619276355 | Not relevant to the main subject/ wrong population or geographical location/ duplicate or repetition |
|  | MHealth for improving quality of antenatal care in northern Ghana: The Bliss4Midwives project.  Adepoju I.O.O., Douwes R., Abugnaba-Abanga R., Van Der Heiden M., Apentibadek N., Zweekhorst M., Bardaji A., Van Roosmalen J., De Brouwere V.  Tropical Medicine and International Health. Conference: 10th European Congress on Tropical Medicine and International Health. Antwerp Belgium. 22(Supplement 1) (pp 81), 2017. Date of Publication: October 2017.  [Conference Abstract]  AN: 618978024 | Not relevant to the main subject/ wrong population or geographical location/ duplicate or repetition |
|  | The Charla de Lupus (lupus chat) program: Assessing the needs of teens and young adults with lupus and their caregivers to develop a family model nutrition and fitness intervention.  Flores M.T., Rose J., Toral P., Mendez L., Pichardo D.M., Horton R., Imundo L.F.  Arthritis and Rheumatology. Conference: American College of Rheumatology/Association of Rheumatology Health Professionals Annual Scientific Meeting, ACR/ARHP 2017. San Diego, CA United States. 69(Supplement 10) (no pagination), 2017. Date of Publication: October 2017.  [Conference Abstract | Not relevant to the main subject/ wrong population or geographical location/ duplicate or repetition |
|  | Baseline characteristics and treatment patterns of Canadian CAnagliflozin REgistry (CanCARE): Assessment of canagliflozin treatment in usual clinical practice in Canada.  Woo V., Bajaj H., Bell A., Clement M., Georgijev N., Camacho F., Culham M.  Diabetologia. Conference: 53rd Annual Meeting of the European Association for the Study of Diabetes, EASD 2017. Lisbon Portugal. 60(1 Supplement 1) (pp S403-S404), 2017. Date of Publication: September 2017.  [Conference Abstract]  AN: 618052451 | Not relevant to the main subject/ wrong population or geographical location/ duplicate or repetition |
|  | Postmortem diagnosis of adult meningitis caused by Streptococcus Agalactiae.  Chindemi C.  Rechtsmedizin. Conference: 10th International Symposium Advances in Legal Medicine combined with the 96th Annual Conference German Society of Legal Medicine. Dusseldorf Germany. 27(4) (pp 344-345), 2017. Date of Publication: August 2017.  [Conference Abstract]  AN: 617954782 | Not relevant to the main subject/ wrong population or geographical location/ duplicate or repetition |
|  | Stent retrieval with a trilobed snare from the right ventricle.  Ramirez D.E., Flores L., Tortolani A.  Journal of Vascular Surgery. Conference: 2017 Vascular Annual Meeting of the Society for Vascular Surgery. San Diego, CA United States. 65(6 Supplement 1) (pp 122S), 2017. Date of Publication: June 2017.  [Conference Abstract]  AN: 617747245 | Not relevant to the main subject/ wrong population or geographical location/ duplicate or repetition |
|  | Racial and ethnic disparities associated with the management of patients with cardiac device-related infections.  Rodriguez Y., Irizarry F., Carrillo R.G.  Heart Rhythm. Conference: 38th Annual Scientific Sessions of the Heart Rhythm Society, Heart Rhythm 2017. Chicago, IL United States. 14(5 Supplement 1) (pp S11), 2017. Date of Publication: May 2017.  [Conference Abstract]  AN: 617041626 | Not relevant to the main subject/ wrong population or geographical location/ duplicate or repetition |
|  | Effectiveness of using a web-based portal on diabetes control in an African-American population.  Repaka N., Nunlee-Bland G., Dai Y., Odonkor W., Ganta V.  Diabetes. Conference: 77th Scientific Sessions of the American Diabetes Association, ADA 2017. San Diego, CA United States. 66(Supplement 1) (pp A227), 2017. Date of Publication: June 2017.  [Conference Abstract]  AN: 616968001 | Not relevant to the main subject/ wrong population or geographical location/ duplicate or repetition |
|  | Characteristics of underserved adults enrolled in the mobile diabetes detective (MoDD) randomized controlled trial.  Heitkemper E., Mamykina L., Cassells A., Tobin J., Smaldone A.  Diabetes. Conference: 77th Scientific Sessions of the American Diabetes Association, ADA 2017. San Diego, CA United States. 66(Supplement 1) (pp A188-A189), 2017. Date of Publication: June 2017.  [Conference Abstract]  AN: 616962468 | Not relevant to the main subject/ wrong population or geographical location/ duplicate or repetition |
|  | Community health workers, mobile health, or both for management of medicaid patients with diabetes.  Katz R.J., Nunlee-Bland G., Magee M.F., Young H., Witkin L., Nassar C., Cohen J.L.  Diabetes. Conference: 77th Scientific Sessions of the American Diabetes Association, ADA 2017. San Diego, CA United States. 66(Supplement 1) (pp A96-A97), 2017. Date of Publication: June 2017.  [Conference Abstract]  AN: 616962044 | Not relevant to the main subject/ wrong population or geographical location/ duplicate or repetition |
|  | Strategies for dissemination and communication in patients with non-insulin-treated type 2 diabetes.  Vu M., Young L., Buse J.B., Mitchell M., Blakeney T., Rees J., Grimm K., Niblock F., Weaver M., Donahue K.  Diabetes. Conference: 77th Scientific Sessions of the American Diabetes Association, ADA 2017. San Diego, CA United States. 66(Supplement 1) (pp A603), 2017. Date of Publication: June 2017.  [Conference Abstract]  AN: 616960901 | Not relevant to the main subject/ wrong population or geographical location/ duplicate or repetition |
|  | Lifecourse alcohol patterns and the risk for diabetes, hypertension and heart problem onset.  Kerr W.C., Ye Y.  Alcoholism: Clinical and Experimental Research. Conference: 40th Annual Scientific Meeting of the Research Society on Alcoholism. Denver, CO United States. 41(Supplement 1) (pp 65A), 2017. Date of Publication: June 2017.  [Conference Abstract]  AN: 616802260 | Not relevant to the main subject/ wrong population or geographical location/ duplicate or repetition |
|  | A review of patients who fail to disclose their HIV status to the GP: Is care compromised?.  Friday D., Meade R., Botchey S., Brook G.  HIV Medicine. Conference: 23rd Annual Conference of the British HIV Association, BHIVA 2017. Liverpool United Kingdom. 18(Supplement 1) (pp 56), 2017. Date of Publication: April 2017.  [Conference Abstract]  AN: 616068181 | Not relevant to the main subject/ wrong population or geographical location/ duplicate or repetition |
|  | Textmessagingto improve outcomes in patients with painful diabetic peripheral neuropathy (PDPN).  Bauer V., Wang C.-H., Goodman N., Craig T.L., Glosner S., Juhn M., Cappelleri J.C., Sadosky A., Cooley C., Lapin B., Masi C.  Journal of General Internal Medicine. Conference: 40th Annual Meeting of the Society of General Internal Medicine, SGIM 2017. Washington, DC United States. 32(2 Supplement 1) (pp S328), 2017. Date of Publication: April 2017.  [Conference Abstract]  AN: 615582204 | Not relevant to the main subject/ wrong population or geographical location/ duplicate or repetition |
|  | Racialvariations in medical care spending patterns among high-risk primary care patients: Results from the STOP-DKD study.  Machen L., Davenport C., Oakes M., Patel U., Diamantidis C.J.  Journal of General Internal Medicine. Conference: 40th Annual Meeting of the Society of General Internal Medicine, SGIM 2017. Washington, DC United States. 32(2 Supplement 1) (pp S299), 2017. Date of Publication: April 2017.  [Conference Abstract]  AN: 615581810 | Not relevant to the main subject/ wrong population or geographical location/ duplicate or repetition |
|  | More than meets the eye: Amyloidoma at insulin injection site.  Austin E.E., Agha A.  Diabetic Medicine. Conference: Diabetes UK Professional Conference 2017. Manchester United Kingdom. 34(Supplement 1) (pp 97), 2017. Date of Publication: March 2017.  [Conference Abstract]  AN: 614844991 | Not relevant to the main subject/ wrong population or geographical location/ duplicate or repetition |
|  | Applying RE-AIM to evaluate two community-based programs designed to improve access to eye care for those at high-risk for glaucoma.  Sapru S., Berktold J., Crews J.E., Katz L.J., Hark L., Girkin C.A., Owsley C., Francis B., Saaddine J.B.  Evaluation and program planning. 65 (pp 40-46), 2017. Date of Publication: 01 Dec 2017.  [Article]  AN: 622751589 | Not relevant to the main subject/ wrong population or geographical location/ duplicate or repetition |
|  | SMS Education for the Promotion of Diabetes Self-Management in Low & Middle Income Countries: A Randomized Controlled Trial in Egypt.  Abaza H., Marschollek M., Schulze M.  Studies in health technology and informatics. 245 (pp 1209), 2017. Date of Publication: 2017.  [Article]  AN: 622454709 | Not relevant to the main subject/ wrong population or geographical location/ duplicate or repetition |
|  | mHealth Application Areas and Technology Combinations*. A Comparison of Literature from High and Low/Middle Income Countries.  Abaza H., Marschollek M.  Methods of information in medicine. 56(7) (pp e105-e122), 2017. Date of Publication: 08 Aug 2017.  [Article]  AN: 622252201 | Not relevant to the main subject/ wrong population or geographical location/ duplicate or repetition |
|  | Mediators and Moderators of Improvements in Medication Adherence.  Hofer R., Choi H., Mase R., Fagerlin A., Spencer M., Heisler M.  Health education & behavior : the official publication of the Society for Public Health Education. 44(2) (pp 285-296), 2017. Date of Publication: 01 Apr 2017.  [Article]  AN: 621961032 | Not relevant to the main subject/ wrong population or geographical location/ duplicate or repetition |
|  | SMS education for the promotion of diabetes self-management in low & middle income countries: a pilot randomized controlled trial in Egypt.  Abaza H., Marschollek M.  BMC public health. 17(1) (pp 962), 2017. Date of Publication: 19 Dec 2017.  [Article]  AN: 621700734 | Included |
|  | Participation of African Americans in e-Health and m-Health Studies: A Systematic Review.  James D.C., Harville C., Sears C., Efunbumi O., Bondoc I.  Telemedicine journal and e-health : the official journal of the American Telemedicine Association. 23(5) (pp 351-364), 2017. Date of Publication: 01 May 2017.  [Review]  AN: 621323658 | Not relevant to the main subject/ wrong population or geographical location/ duplicate or repetition |
|  | Bibliometric analysis of worldwide scientific literature in mobile - health: 2006-2016.  Sweileh W.M., Al-Jabi S.W., AbuTaha A.S., Zyoud S.H., Anayah F.M.A., Sawalha A.F.  BMC medical informatics and decision making. 17(1) (pp 72), 2017. Date of Publication: 30 May 2017.  [Article]  AN: 620722965 | Not relevant to the main subject/ wrong population or geographical location/ duplicate or repetition |
|  | Telemedicine to Improve Access to Specialist Care in Fetal Heart Rate Monitoring: Analysis of 17 Years of TOCOMAT Network Clinical Activity.  Tagliaferri S., Esposito F.G., Ippolito A., Mereghini F., Magenes G., Martinelli P., Campanile M., Signorini M.G.  Telemedicine journal and e-health : the official journal of the American Telemedicine Association. 23(3) (pp 226-232), 2017. Date of Publication: 01 Mar 2017.  [Article]  AN: 619640722 | Not relevant to the main subject/ wrong population or geographical location/ duplicate or repetition |
|  | Oxidative stress and expression of insulin signaling proteins in the brain of diabetic rats: Role of Nigella sativa oil and antidiabetic drugs.  Balbaa M., Abdulmalek S.A., Khalil S.  PLoS ONE. 12(5) (no pagination), 2017. Article Number: e0172429. Date of Publication: May 2017.  [Article]  AN: 616160657 | Not relevant to the main subject/ wrong population or geographical location/ duplicate or repetition |
|  | Pharmacopoeia and herbal monograph, the aim and use of WHO's herbal monograph, WHO's guide lines for herbal monograph, pharmacognostical research and monographs of organized, unorganized drugs and drugs from animal sources.  Alamgir A.N.M.  Progress in Drug Research. 73 (pp 295-353), 2017. Date of Publication: 2017.  [Chapter]  AN: 618223379 | Not relevant to the main subject/ wrong population or geographical location/ duplicate or repetition |
|  | Effect of a community health worker intervention among Latinos with poorly controlled type 2 diabetes: The miami healthy heart initiative randomized clinical trial.  Carrasquillo O., Lebron C., Alonzo Y., Li H., Chang A., Kenya S.  JAMA Internal Medicine. 177(7) (pp 948-954), 2017. Date of Publication: July 2017.  [Article]  AN: 617402703 | Not relevant to the main subject/ wrong population or geographical location/ duplicate or repetition |
|  | Mitochondrial respiration and ROS emission during beta-oxidation in the heart: An experimental-computational study.  Cortassa S., Sollott S.J., Aon M.A.  PLoS Computational Biology. 13(6) (no pagination), 2017. Article Number: e1005588. Date of Publication: June 2017.  [Article]  AN: 617155739 | Not relevant to the main subject/ wrong population or geographical location/ duplicate or repetition |
|  | A study on the interaction between metformin and constituents of a commercial herbal product.  Arhewoh M.I., Eraga S.O., Irabor J., Iwuagwu M.A.  Tropical Journal of Pharmaceutical Research. 16(7) (pp 1703-1709), 2017. Date of Publication: July 2017.  [Article]  AN: 617689425 | Not relevant to the main subject/ wrong population or geographical location/ duplicate or repetition |
|  | A qualitative study to explore the perception and behavior of patients towards diabetes management with physical disability.  Gillani S.W., Sulaiman S.A.S., Abdul M.I.M., Saad S.Y.  Diabetology and Metabolic Syndrome. 9(1) (no pagination), 2017. Article Number: 58. Date of Publication: 24 Jul 2017.  [Article]  AN: 617459410 | Not relevant to the main subject/ wrong population or geographical location/ duplicate or repetition |
|  | Cholecalciferol improves glycemic control in type 2 diabetic patients: A 6-month prospective interventional study.  Nada A.M., Shaheen D.A.  Therapeutics and Clinical Risk Management. 13 (pp 813-820), 2017. Date of Publication: 07 Jul 2017.  [Article]  AN: 617280019 | Not relevant to the main subject/ wrong population or geographical location/ duplicate or repetition |
|  | Effectiveness of a targeted education module for healthcare professionals attending a diabetic retinopathy training session in Zimbabwe.  Woodward R., Matimba A.  African Journal of Diabetes Medicine. 25(1) (pp 21-23), 2017. Date of Publication: 01 May 2017.  [Article]  AN: 616943325 | Not relevant to the main subject/ wrong population or geographical location/ duplicate or repetition |
|  | Diabetes in Sub-Saharan Africans: Relevance for the Belgian clinician. Diabetes bij Sub-Sahara-Afrikanen: Relevantie voor de kliniek in Belgie <Diabetes bij Sub-Sahara-Afrikanen: Relevantie voor de kliniek in Belgie.>  Van Offel S., Van Olmen J., De Block C., Rottiers R.  Tijdschrift voor Geneeskunde. 73(7) (pp 423-428), 2017. Date of Publication: 01 Apr 2017.  [Article]  AN: 615288664 | Not relevant to the main subject/ wrong population or geographical location/ duplicate or repetition |
|  | Fasting, Diabetes, and Optimizing Health Outcomes for Ramadan Observers: A Literature Review.  Almansour H.A., Chaar B., Saini B.  Diabetes Therapy. 8(2) (pp 227-249), 2017. Date of Publication: 01 Apr 2017.  [Review]  AN: 615166380 | Not relevant to the main subject/ wrong population or geographical location/ duplicate or repetition |
|  | mHealth Interventions to Counter Noncommunicable Diseases in Developing Countries: Still an Uncertain Promise.  Beratarrechea A., Moyano D., Irazola V., Rubinstein A.  Cardiology Clinics. 35(1) (pp 13-30), 2017. Date of Publication: 01 Feb 2017.  [Review]  AN: 613411203 | Not relevant to the main subject/ wrong population or geographical location/ duplicate or repetition |
|  | The effect of text message support on diabetes self-management in developing countries - A randomised trial.  Van Olmen J., Kegels G., Korachais C., de Man J., Van Acker K., Kalobu J.C., van Pelt M., Ku G.M., Hen H., Kanda D., Malombo B., Darras C., Schellevis F.  Journal of Clinical and Translational Endocrinology. 7 (pp 33-41), 2017. Date of Publication: 01 Mar 2017.  [Article]  AN: 614202205 | Not relevant to the main subject/ wrong population or geographical location/ duplicate or repetition |
|  | Evaluation of Text Messaging Effects on Health Goal Adherence in the Management of Participants With Chronic Diseases.  Stagg S.J., Speroni K.G., Daniel M.G., Eigenbrode M., Geisler L.  Professional case management. 22(3) (pp 126-135), 2017. Date of Publication: 01 May 2017.  [Article]  AN: 617856903 | Not relevant to the main subject/ wrong population or geographical location/ duplicate or repetition |
|  | Process evaluation of a mobile health intervention for people with diabetes in low income countries - the implementation of the TEXT4DSM study.  Van Olmen J., Van Pelt M., Malombo B., Ku G.M., Kanda D., Heang H., Darras C., Kegels G., Schellevis F.  Journal of telemedicine and telecare. 23(1) (pp 96-105), 2017. Date of Publication: 01 Jan 2017.  [Article]  AN: 617450777 | Not relevant to the main subject/ wrong population or geographical location/ duplicate or repetition |
|  | Rheumatoid arthritis: Notable biomarkers linking to chronic systemic conditions and cancer.  Olumuyiwa-Akeredolu O.-O.O., Pretorius E.  Current Pharmaceutical Design. 22(7) (pp 918-924), 2016. Date of Publication: 01 Feb 2016.  [Article]  AN: 608517326 | Not relevant to the main subject/ wrong population or geographical location/ duplicate or repetition |
|  | A guide for using experimental design in chromatographic method development: Applied to the analysis of selected anti-diabetic pharmaceutical combinations.  Ayoub B.M., Abdel-Aziz O.  Pharmazie. 71(12) (pp 683-690), 2016. Date of Publication: 01 Aug 2016.  [Article]  AN: 613451140 | Not relevant to the main subject/ wrong population or geographical location/ duplicate or repetition |
|  | Usability of Commercially Available Mobile Applications for Diverse Patients.  Sarkar U., Gourley G.I., Lyles C.R., Tieu L., Clarity C., Newmark L., Singh K., Bates D.W.  Journal of General Internal Medicine. 31(12) (pp 1417-1426), 2016. Date of Publication: 01 Dec 2016.  [Article]  AN: 611262168 | Not relevant to the main subject/ wrong population or geographical location/ duplicate or repetition |
|  | Open-label randomized trial of titrated disease management for patients with hypertension: Study design and baseline sample characteristics.  Jackson G.L., Weinberger M., Kirshner M.A., Stechuchak K.M., Melnyk S.D., Bosworth H.B., Coffman C.J., Neelon B., Van Houtven C., Gentry P.W., Morris I.J., Rose C.M., Taylor J.P., May C.L., Han B., Wainwright C., Alkon A., Powell L., Edelman D.  Contemporary Clinical Trials. 50 (pp 5-15), 2016. Date of Publication: 01 Sep 2016.  [Article]  AN: 611231527 | Not relevant to the main subject/ wrong population or geographical location/ duplicate or repetition |
|  | Challenges confronting African Americans and Hispanics living with chronic illness in their families.  Saulsberry L., Blendon R.J., Benson J.M.  Chronic Illness. 12(4) (pp 281-291), 2016. Date of Publication: 01 Dec 2016.  [Article]  AN: 613342178 | Not relevant to the main subject/ wrong population or geographical location/ duplicate or repetition |
|  | Implementation and Operational Research: Cost and Efficiency of a Hybrid Mobile Multidisease Testing Approach with High HIV Testing Coverage in East Africa.  Chang W., Chamie G., Mwai D., Clark T.D., Thirumurthy H., Charlebois E.D., Petersen M., Kabami J., Ssemmondo E., Kadede K., Kwarisiima D., Sang N., Bukusi E.A., Cohen C.R., Kamya M., Havlir D.V., Kahn J.G.  Journal of Acquired Immune Deficiency Syndromes. 73(3) (pp e39-e45), 2016. Date of Publication: 01 Nov 2016.  [Article]  AN: 611645738 | Not relevant to the main subject/ wrong population or geographical location/ duplicate or repetition |
|  | Smartphone for Managing Diabetes.  Joob B., Wiwanitkit V.  Canadian Journal of Diabetes. 40(2) (pp 108), 2016. Date of Publication: 01 Apr 2016.  [Letter]  AN: 607755655  Embase Accession Number  20160042175  PMID  26778683 [<http://www.ncbi.nlm.nih.gov/pubmed/?term=26778683>]  Status | Not relevant to the main subject/ wrong population or geographical location/ duplicate or repetition |
|  | Knowledge, attitudes, and practices associated with chronic kidney disease in northern Tanzania: A community-based study.  Stanifer J.W., Turner E.L., Egger J.R., Thielman N., Karia F., Maro V., Kilonzo K., Patel U.D., Yeates K.  PLoS ONE. 11(6) (no pagination), 2016. Article Number: e0156336. Date of Publication: 01 Jun 2016.  [Article]  AN: 610893062 | Not relevant to the main subject/ wrong population or geographical location/ duplicate or repetition |
|  | Quality improvement for cardiovascular disease care in low- and middle-income countries: A systematic review.  Lee E.S., Vedanthan R., Jeemon P., Kamano J.H., Kudesia P., Rajan V., Engelgau M., Moran A.E.  PLoS ONE. 11(6) (no pagination), 2016. Article Number: e0157036. Date of Publication: June 2016.  [Review]  AN: 610895384 | Not relevant to the main subject/ wrong population or geographical location/ duplicate or repetition |
|  | A complex behavioural change intervention to reduce the risk of diabetes and prediabetes in the pre-conception period in Malaysia: Study protocol for a randomised controlled trial.  Skau J.K.H., Nordin A.B.A., Cheah J.C.H., Ali R., Zainal R., Aris T., Ali Z.M., Matzen P., Biesma R., Aagaard-Hansen J., Hanson M.A., Norris S.A.  Trials. 17(1) (no pagination), 2016. Article Number: 215. Date of Publication: 27 Apr 2016.  [Article]  AN: 610067860 | Not relevant to the main subject/ wrong population or geographical location/ duplicate or repetition |
|  | A hybrid mobile approach for population-wide HIV testing in rural east Africa: An observational study.  Chamie G., Clark T.D., Kabami J., Kadede K., Ssemmondo E., Steinfeld R., Lavoy G., Kwarisiima D., Sang N., Jain V., Thirumurthy H., Liegler T., Balzer L.B., Petersen M.L., Cohen C.R., Bukusi E.A., Kamya M.R., Havlir D.V., Charlebois E.D.  The Lancet HIV. 3(3) (pp e111-e119), 2016. Date of Publication: 01 Mar 2016.  [Article]  AN: 608303074 | Not relevant to the main subject/ wrong population or geographical location/ duplicate or repetition |
|  | Assessing the needs of older HIV+ adults; Initial data from the core healthy aging initiative (CHAI).  Adeyemi O., Catrambone J., Rebolledo W., Burke K., Bahk M., Carmack A.  Open Forum Infectious Diseases. Conference: ID Week 2016. New Orleans, LA United States. 3(Supplement 1) (no pagination), 2016. Date of Publication: September 2016.  [Conference Abstract]  AN: 627972141 | Not relevant to the main subject/ wrong population or geographical location/ duplicate or repetition |
|  | Development and feasibility of the reach tailored text messaging intervention for low-income adults with type 2 diabetes.  Nelson L.A., Mayberry L.S., Wallston K.A., Kripalani S., Gentry C.K., Brown A., Gregory B.P., Acuff S.W., Harper K.J., Bergner E.M., Lestourgeon L.M., Mcphillips S.E., Gebretsadik T., Elasy T.A., Johnson K.B., Osborn C.Y.  Diabetes. Conference: 76th Scientific Sessions of the American Diabetes Association, ADA 2016. New Orleans, LA United States. 65(Supplement 1) (pp A207), 2016. Date of Publication: 2016.  [Conference Abstract]  AN: 620237843 | Not relevant to the main subject/ wrong population or geographical location/ duplicate or repetition |
|  | Technology for longevity: Predictors for use of technology.  Kort H.S.M.  Medicina (Brazil). Conference: 1. Congresso Brasileiro de Gerontecnologia. Ribeirao Preto, SP Brazil. 49(Supplement 2) (pp 24-26), 2016. Date of Publication: April 2016.  [Conference Abstract]  AN: 619950835 | Not relevant to the main subject/ wrong population or geographical location/ duplicate or repetition |
|  | Community-based screening for cardiovascular risk using a novel mobile health (mhealth) technology in rural Kenya.  Ochieng B.O., Aw J., Mannik J., Chesire J., Hawkes M.  American Journal of Tropical Medicine and Hygiene. Conference: 65th Annual Meeting of the American Society of Tropical Medicine and Hygiene, ASTMH 2016. Atlanta, GA United States. 95(5 Supplement 1) (pp 63-64), 2016. Date of Publication: November 2016.  [Conference Abstract]  AN: 619250388 | Not relevant to the main subject/ wrong population or geographical location/ duplicate or repetition |
|  | Wills eye community intervention to improve glaucoma detection and followup care.  Hark L.A., Katz L.J., Johnson D.M., Molineaux J., Myers J.S., Resende A., Reber S., Waisbourd M., Leiby B., Haller J.A.  Investigative Ophthalmology and Visual Science. Conference: 2016 Annual Meeting of the Association for Research in Vision and Ophthalmology, ARVO 2016. Seattle, WA United States. 57(12) (pp 2588), 2016. Date of Publication: September 2016.  [Conference Abstract]  AN: 616081575 | Not relevant to the main subject/ wrong population or geographical location/ duplicate or repetition |
|  | Optimizing amyloid beta detection in retinas of a double transgenic Alzheimer s disease mouse model and of human subjects with dementia.  Knels L., Hempel S., Valtink M., Funk R., Ader M., Schroeder C., Loffler J.  Investigative Ophthalmology and Visual Science. Conference: 2016 Annual Meeting of the Association for Research in Vision and Ophthalmology, ARVO 2016. Seattle, WA United States. 57(12) (pp 114), 2016. Date of Publication: September 2016.  [Conference Abstract]  AN: 616081139 | Not relevant to the main subject/ wrong population or geographical location/ duplicate or repetition |
|  | Implementing a telemedicine screening program for ophthalmic disease at bugando medical center in Mwanza, Tanzania.  Coombs P., Nicol C., Shimba J., Rweyemamu S., Mutayabalwa D., Peck R., Smart L., Chan R.V.P., Jonas K., Sun G.  Investigative Ophthalmology and Visual Science. Conference: 2016 Annual Meeting of the Association for Research in Vision and Ophthalmology, ARVO 2016. Seattle, WA United States. 57(12) (pp 1581), 2016. Date of Publication: September 2016.  [Conference Abstract]  AN: 616036805 | Not relevant to the main subject/ wrong population or geographical location/ duplicate or repetition |
|  | Awareness and management of elevated blood pressure among HIV-infected adults receiving antiretroviral therapy in urban Zambia.  Bauer S., Zyambo Z., Mwanza M., Chilengi R., Davies M.-A., Egger M., Furrer H., Vinikoor M., Wandeler G.  Journal of the International AIDS Society. Conference: International Congress of Drug Therapy in HIV Infection 2016. Glasgow United Kingdom. 19(Supplement 7) (pp 135-136), 2016. Date of Publication: October 2016.  [Conference Abstract]  AN: 615420740 | Not relevant to the main subject/ wrong population or geographical location/ duplicate or repetition |
|  | Pancreatic cancer - Our experience.  Abdallah R.  Annals of Oncology. Conference: 18th World Congress on Gastrointestinal Cancer, ESMO 2016. Barcelona Spain. 27(Supplement 2) (pp ii98), 2016. Date of Publication: June 2016.  [Conference Abstract]  AN: 613674945 | Not relevant to the main subject/ wrong population or geographical location/ duplicate or repetition |
|  | The efficacy of a diabetic registry to improve survival rates in under-resourced environments.  Anderson T., Maynor A., Beech D., Alema-Mensah E.  Cancer Epidemiology Biomarkers and Prevention. Conference: 8th AACR Conference on the Science of Health Disparities in Racial/Ethnic Minorities and the Medically Underserved. Atlanta, GA United States. 25(3 Supplement) (no pagination), 2016. Date of Publication: March 2016.  [Conference Abstract]  AN: 613276243 | Not relevant to the main subject/ wrong population or geographical location/ duplicate or repetition |
|  | 8th AACR Conference on the Science of Health Disparities in Racial/Ethnic Minorities and the Medically Underserved.  Anonymous  Cancer Epidemiology Biomarkers and Prevention. Conference: 8th AACR Conference on the Science of Health Disparities in Racial/Ethnic Minorities and the Medically Underserved. Atlanta, GA United States. 25(3 Supplement) (no pagination), 2016. Date of Publication: March 2016.  [Conference Review]  AN: 613272988 | Not relevant to the main subject/ wrong population or geographical location/ duplicate or repetition |
|  | Costs of hybrid mobile multi-disease testing with high HIV test coverage, East Africa.  Chang W., Chamie G., Thirumurthy H., Clark T., Charlebois E., Petersen M., Kamya M.R., Havlir D.V., Kahn J.G.  Topics in Antiviral Medicine. Conference: 23rd Conference on Retroviruses and Opportunistic Infections, CROI 2016. Boston, MA United States. 24(E-1) (pp 461), 2016. Date of Publication: June 2016.  [Conference Abstract]  AN: 613267794 | Not relevant to the main subject/ wrong population or geographical location/ duplicate or repetition |
|  | Diabetic macular edema, budget impact analysis from the perspective of the Algerian public health system.  Chachoua A.  Value in Health. Conference: ISPOR 19th Annual European Congress. Vienna Austria. 19(7) (pp A565), 2016. Date of Publication: November 2016.  [Conference Abstract]  AN: 613236818 | Not relevant to the main subject/ wrong population or geographical location/ duplicate or repetition |
|  | Towards a personalised care of T1DM in a nonprofit organisation, T1 Diams, in Mauritius.  Guness P.K., Dustagheer A., Jean Pierre D.  Pediatric Diabetes. Conference: 42nd Annual Meeting of the International Society for Pediatric and Adolescent Diabetes, ISPAD 2016. Valencia Spain. 17(Supplement 24) (pp 130), 2016. Date of Publication: October 2016.  [Conference Abstract]  AN: 613188621 | Not relevant to the main subject/ wrong population or geographical location/ duplicate or repetition |
|  | Protective effects of asiatic acid in spontaneous type 2 diabetic mice model.  Sun W., Xu G., Luo G., Wu L., Hou Y., Guo X., Zhou J., Xu T., Qin L., Fan Y., Han L., Matsabisa M., Ma X., Liu T.  Journal of Alternative and Complementary Medicine. Conference: International Congress on Integrative Medicine and Health, ICIMH 2016. Las Vegas, NV United States. 22(6) (pp A36), 2016. Date of Publication: 2016.  [Conference Abstract]  AN: 611808340 | Not relevant to the main subject/ wrong population or geographical location/ duplicate or repetition |
|  | Profile of patients admitted for hypertensive acute heart failure.  Bami A., Houari C., Kemayou N., Kapche D., Azzouzi L., Habbal R.  Archives of Cardiovascular Diseases Supplements. Conference: Printemps de la Cardiologie 2016. Dijon France. Conference Publication: (var.pagings). 8(3) (pp 236-237), 2016. Date of Publication: April 2016.  [Conference Abstract]  AN: 72300411 | Not relevant to the main subject/ wrong population or geographical location/ duplicate or repetition |
|  | Using text messages to support improved self-care decisions in medically underserved African-Americans with uncontrolled diabetes: Best practices from the patient's perspective.  Udoko A.N., Bailey J.E., Ransone S., Binkley B.L., Gatwood J., Coday M., Graff J.C.  Journal of General Internal Medicine. Conference: 39th Annual Meeting of the Society of General Internal Medicine, SGIM 2016. Hollywood, FL United States. Conference Publication: (var.pagings). 31(2 SUPPL. 1) (pp S460-S461), 2016. Date of Publication: May 2016.  [Conference Abstract]  AN: 72288817 | Not relevant to the main subject/ wrong population or geographical location/ duplicate or repetition |
|  | Primary care engagement in emergency department patients with multiple chronic conditions in a medically underserved area.  Jackson B., Carlton E.L., Relyea G., Ahn S.N., Bailey J.E.  Journal of General Internal Medicine. Conference: 39th Annual Meeting of the Society of General Internal Medicine, SGIM 2016. Hollywood, FL United States. Conference Publication: (var.pagings). 31(2 SUPPL. 1) (pp S360-S361), 2016. Date of Publication: May 2016.  [Conference Abstract]  AN: 72288630 | Not relevant to the main subject/ wrong population or geographical location/ duplicate or repetition |
|  | Mobile apps forvulnerable populations study.  Sarkar U., Gourley G.I., Lyles C., Tieu L., Clarity C., Newmark L., Singh K., Bates D.W.  Journal of General Internal Medicine. Conference: 39th Annual Meeting of the Society of General Internal Medicine, SGIM 2016. Hollywood, FL United States. Conference Publication: (var.pagings). 31(2 SUPPL. 1) (pp S303), 2016. Date of Publication: May 2016.  [Conference Abstract]  AN: 72288528 | Not relevant to the main subject/ wrong population or geographical location/ duplicate or repetition |
|  | Delivery of a dietary intervention using text messaging among english or Spanish-speaking adults.  Collins T.C., Valverde M.G., Lu K., Geana M.  Journal of General Internal Medicine. Conference: 39th Annual Meeting of the Society of General Internal Medicine, SGIM 2016. Hollywood, FL United States. Conference Publication: (var.pagings). 31(2 SUPPL. 1) (pp S174-S175), 2016. Date of Publication: May 2016.  [Conference Abstract]  AN: 72288292 | Not relevant to the main subject/ wrong population or geographical location/ duplicate or repetition |
|  | A tailored breastfeeding support intervention for women with gestational diabetes.  Stuebe A.M., Bonuck K., Adatorwovor R., Schwartz T.A., Berry D.  American Journal of Obstetrics and Gynecology. Conference: 36th Annual Meeting of the Society for Maternal-Fetal Medicine: The Pregnancy Meeting. Atlanta, GA United States. Conference Publication: (var.pagings). 214(1 SUPPL. 1) (pp S68), 2016. Date of Publication: January 2016.  [Conference Abstract]  AN: 72164294 | Not relevant to the main subject/ wrong population or geographical location/ duplicate or repetition |
|  | Group Conference Call Diabetes Educational Support Sessions: A Pilot Study.  Appel S.J., Buxbaum S.G.  Journal of National Black Nurses' Association : JNBNA. 27(2) (pp 32-38), 2016. Date of Publication: 01 Dec 2016.  [Article]  AN: 623362509 | Not relevant to the main subject/ wrong population or geographical location/ duplicate or repetition |
|  | The effectiveness of e-& mHealth interventions to promote physical activity and healthy diets in developing countries: A systematic review.  Muller A.M., Alley S., Schoeppe S., Vandelanotte C.  International Journal of Behavioral Nutrition and Physical Activity. 13(1) (no pagination), 2016. Article Number: 109. Date of Publication: 10 Oct 2016.  [Review]  AN: 612599620 | Not relevant to the main subject/ wrong population or geographical location/ duplicate or repetition |
|  | The Design, Usability, and Feasibility of a Family-Focused Diabetes Self-Care Support mHealth Intervention for Diverse, Low-Income Adults with Type 2 Diabetes.  Mayberry L.S., Berg C.A., Harper K.J., Osborn C.Y.  Journal of Diabetes Research. 2016 (no pagination), 2016. Article Number: 7586385. Date of Publication: 2016.  [Article]  AN: 613373260 | Not relevant to the main subject/ wrong population or geographical location/ duplicate or repetition |
|  | Tablet-Aided BehavioraL intervention EffecT on Self-management skills (TABLETS) for Diabetes.  Lynch C.P., Williams J.S., Ruggiero K.J., Knapp R.G., Egede L.E.  Trials. 17(1) (no pagination), 2016. Article Number: 157. Date of Publication: 2016.  [Article]  AN: 613896944 | Not relevant to the main subject/ wrong population or geographical location/ duplicate or repetition |
|  | Application of spiking technique coupled with derivative spectrophotometry for the analysis of a novel anti-diabetic combination of two coformulated drugs with highly different concentrations.  Ayoub B.M.  Der Pharma Chemica. 8(10) (pp 12-14), 2016. Date of Publication: 2016.  [Short Survey]  AN: 612192793 | Not relevant to the main subject/ wrong population or geographical location/ duplicate or repetition |
|  | Understanding the Knowledge Gap Experienced by U.S. Safety Net Patients in Teleretinal Screening.  George S.M., Hayes E.M., Fish A., Daskivich L.P., Ogunyemi O.I.  AMIA ... Annual Symposium proceedings. AMIA Symposium. 2016 (pp 590-599), 2016. Date of Publication: 2016.  [Article]  AN: 617665901 | Not relevant to the main subject/ wrong population or geographical location/ duplicate or repetition |
|  | Pre-pregnancy community-based intervention for couples in Malaysia: application of intervention mapping.  Norris S.A., Ho J.C., Rashed A.A., Vinding V., Skau J.K., Biesma R., Aagaard-Hansen J., Hanson M., Matzen P.  BMC public health. 16(1) (pp 1167), 2016. Date of Publication: 17 Nov 2016.  [Article]  AN: 617451377 | Not relevant to the main subject/ wrong population or geographical location/ duplicate or repetition |
|  | Tele-ophthalmology: Opportunities for improving diabetes eye care in resource- and specialist-limited Sub-Saharan African countries.  Matimba A., Woodward R., Tambo E., Ramsay M., Gwanzura L., Guramatunhu S.  Journal of telemedicine and telecare. 22(5) (pp 311-316), 2016. Date of Publication: 01 Jul 2016.  [Article]  AN: 617025501 | Not relevant to the main subject/ wrong population or geographical location/ duplicate or repetition |
|  | A needs assessment of people living with diabetes and diabetic retinopathy.  Hall C.E., Hall A.B., Kok G., Mallya J., Courtright P.  BMC research notes. 9 (pp 56), 2016. Date of Publication: 01 Feb 2016.  [Article]  AN: 616093651 | Not relevant to the main subject/ wrong population or geographical location/ duplicate or repetition |
|  | Diabetes Applications for Arabic Speakers: A Critical Review of Available Apps for Android and iOS Operated Smartphones.  Alhuwail D.  Studies in health technology and informatics. 225 (pp 587-591), 2016. Date of Publication: 2016.  [Article]  AN: 615534027 | Not relevant to the main subject/ wrong population or geographical location/ duplicate or repetition |
|  | Continuing medical education (CME) in diabetes of GP's in Algeria, 2004-2014. Formation medicale continue (FMC) au diabete des medecins generalistes en Algerie, 2004-2014 <Formation medicale continue (FMC) au diabete des medecins generalistes en Algerie, 2004-2014.>  Malek R., Roula D., Belhadj M., Babinet F., Khalfa S., Lezzar E.-K., Benfenatki N., Bouderda Z., Rezig M.-F.  Medecine des Maladies Metaboliques. 9(2) (pp 203-206), 2015. Date of Publication: March 2015.  [Article]  AN: 2000577269 | Not relevant to the main subject/ wrong population or geographical location/ duplicate or repetition |
|  | 8th Annual Symposium on Self-Monitoring of Blood Glucose (SMBG): April 16-18, 2015, Republic of Malta.  Parkin C.G., Homberg A., Hinzmann R.  Diabetes Technology and Therapeutics. 17(11) (pp 832-850), 2015. Date of Publication: November 2015.  [Conference Paper]  AN: 606817895 | Not relevant to the main subject/ wrong population or geographical location/ duplicate or repetition |
|  | Impaired mitochondrial energy supply coupled to increased H2O2 emission under energy/redox stress leads to myocardial dysfunction during Type I diabetes.  Tocchetti C.G., Stanley B.A., Sivakumaran V., Bedja D., O'Rourke B., Paolocci N., Cortassa S., Aon M.A.  Clinical Science. 129(7) (pp 561-574), 2015. Date of Publication: 2015.  [Article]  AN: 607429183 | Not relevant to the main subject/ wrong population or geographical location/ duplicate or repetition |
|  | The metabolic syndrome in hypertensive black population of South Algeria. Le syndrome metabolique chez les hypertendus de la population noire du sud algerien <Le syndrome metabolique chez les hypertendus de la population noire du sud algerien.>  Bachir Cherif A., Temmar M., Chibane A., Labat C., Atif M.L., Taleb A., Benetos A., Bouafia M.T.  Annales de Cardiologie et d'Angeiologie. 64(3) (pp 158-163), 2015. Date of Publication: 01 Jun 2015.  [Article]  AN: 604647675 | Not relevant to the main subject/ wrong population or geographical location/ duplicate or repetition |
|  | Phone-based intervention under nurse guidance after stroke: Concept for lowering blood pressure after stroke in Sub-Saharan Africa.  Ovbiagele B.  Journal of Stroke and Cerebrovascular Diseases. 24(1) (pp 1-9), 2015. Date of Publication: 01 Jan 2015.  [Review]  AN: 601179081 | Not relevant to the main subject/ wrong population or geographical location/ duplicate or repetition |
|  | MDiabetes: A diabetes prevention initiative via mobile phones, in Senegal. MDiabete : le mobile au service de la lutte contre le diabete au Senegal <MDiabete : le mobile au service de la lutte contre le diabete au Senegal.>  Mbaye M.N., Diop S.-N., Sarr A., Cisse M.K., Niang M.N., Gueye B.-O., Eskandar H., Kleinebreil L.  Medecine des Maladies Metaboliques. 9(2) (pp 143-146), 2015. Date of Publication: 2015.  [Article]  AN: 607792907 | Not relevant to the main subject/ wrong population or geographical location/ duplicate or repetition |
|  | Smartphone-based visual acuitymeasurement for screening and clinical assessment.  Brady C.J., Eghrari A.O., Labrique A.B.  JAMA - Journal of the American Medical Association. 314(24) (pp 2682-2683), 2015. Date of Publication: 22 Dec 2015.  [Review]  AN: 607435127 | Not relevant to the main subject/ wrong population or geographical location/ duplicate or repetition |
|  | Development and validation of a smartphone-based visual acuity test (peek acuity) for clinical practice and Community-Based Fieldwork.  Bastawrous A., Rono H.K., Livingstone I.A.T., Weiss H.A., Jordan S., Kuper H., Burton M.J.  JAMA Ophthalmology. 133(8) (pp 930-937), 2015. Date of Publication: 01 Aug 2015.  [Article]  AN: 605678331 | Not relevant to the main subject/ wrong population or geographical location/ duplicate or repetition |
|  | Tackling the growing diabetes burden in Sub-Saharan Africa: A framework for enhancing outcomes in stroke patients.  Ovbiagele B.  Journal of the Neurological Sciences. 348(1-2) (pp 136-141), 2015. Date of Publication: 15 Jan 2015.  [Article]  AN: 601389658 | Not relevant to the main subject/ wrong population or geographical location/ duplicate or repetition |
|  | Effect of YoYo bitters on the dissolution of lisinopril tablets.  Ayandokun Olubunmi A., Oyetunde Olubukola O., Akinleye Moshood A.  Dissolution Technologies. 22(1) (pp 6-10), 2015. Date of Publication: 2015.  [Article]  AN: 602890983 | Not relevant to the main subject/ wrong population or geographical location/ duplicate or repetition |
|  | The impact of changing antiseptic skin preparation agent used for cardiac implantable electronic device (CIED) procedures on the risk of infection.  Qintar M., Zardkoohi O., Hammadah M., Hsu A., Wazni O., Wilkoff B.L., Tarakji K.G.  PACE - Pacing and Clinical Electrophysiology. 38(2) (pp 240-246), 2015. Date of Publication: 01 Feb 2015.  [Article]  AN: 602219035 | Not relevant to the main subject/ wrong population or geographical location/ duplicate or repetition |
|  | A sacrospinous ligament fixation under local anesthesia in Djibouti.  Senturk M.B., Yildiz Y.Y., Yildiz S., Guraslan H.  Journal of Gynecologic Surgery. 31(3) (pp 184-186), 2015. Date of Publication: 01 Jun 2015.  [Article]  AN: 604931428 | Not relevant to the main subject/ wrong population or geographical location/ duplicate or repetition |
|  | Text-messaging program improves outcomes in outpatient cardiovascular rehabilitation.  Lounsbury P., Elokda A.S., Gylten D., Arena R., Clarke W., Gordon E.E.I.  IJC Heart and Vasculature. 7 (pp 170-175), 2015. Date of Publication: June 01, 2015.  [Article]  AN: 604592064 | Not relevant to the main subject/ wrong population or geographical location/ duplicate or repetition |
|  | Positive impact of self-monitoring of blood glucose on diabetes management in male patients with type 2 diabetes from aseer diabetic center, Abha, Kingdom of Saudi Arabia.  Omer S.H., Qahtani M.A.A.A., Altieb A.M., Awwad A.A., Al-Gathradhi M., Vijayaraghavalu S.  Pharmacie Globale. 6(3) (pp 1-5), 2015. Date of Publication: 2015.  [Article]  AN: 605640531 | Not relevant to the main subject/ wrong population or geographical location/ duplicate or repetition |
|  | The EQUALITY Program: Evaluating change in patient attitudes and knowledge about glaucoma.  Rhodes L.A., Huisingh C.E., McGwin G., Mennemeyer S., Crews J., Girkin C.A., Owsley C.  Investigative Ophthalmology and Visual Science. Conference: 2015 Annual Meeting of the Association for Research in Vision and Ophthalmology, ARVO 2015. Denver, CO United States. 56(7) (pp 3705), 2015. Date of Publication: June 2015.  [Conference Abstract]  AN: 615919064 | Not relevant to the main subject/ wrong population or geographical location/ duplicate or repetition |
|  | Prinzmetal angina in major hemophilia a patient: A case report.  Massi M.R., Houssou B., Camara M., Nisserine K., Quessar A.A.Q., Oukkache B.  Blood. Conference: 57th Annual Meeting of the American Society of Hematology, ASH 2015. San Diego, CA United States. Conference Publication: (var.pagings). 126(23) (pp 4711), 2015. Date of Publication: 03 Dec 2015.  [Conference Abstract]  AN: 72175401 | Not relevant to the main subject/ wrong population or geographical location/ duplicate or repetition |
|  | The characteristics, functional impact, physical activity levels and health related quality of life in women with chronic musculoskeletal disease and chronic diseases attending community health centers in south africa.  Hendricks C., Jelsma J., Parker R.  Annals of the Rheumatic Diseases. Conference: Annual European Congress of Rheumatology of the European League Against Rheumatism, EULAR 2015. Rome Italy. Conference Publication: (var.pagings). 74(SUPPL. 2) (pp 1313-1314), 2015. Date of Publication: June 2015.  [Conference Abstract]  AN: 72154627 | Not relevant to the main subject/ wrong population or geographical location/ duplicate or repetition |
|  | The role of KCNJ11 gene in neonatal diabetes.  El Dayem S.A., Shawky S., El Kader M.A., Kamel S., Khalifa R.H., Lebedy D.E., Ahmed D.  Hormone Research in Paediatrics. Conference: 54th Annual Meeting of the European Society for Paediatric Endocrinology, ESPE 2015. Barcelona Spain. Conference Publication: (var.pagings). 84(SUPPL. 1) (pp 386), 2015. Date of Publication: September 2015.  [Conference Abstract]  AN: 72086144 | Not relevant to the main subject/ wrong population or geographical location/ duplicate or repetition |
|  | Evaluation of physician awareness of the presentations of type 1 diabetes in children and adolescents in Egypt, a pilot study.  Aly H.H., Abd Elmaksoud A., Awad A.  Pediatric Diabetes. Conference: Joint Annual Conference of the International Society for Pediatric and Adolescent Diabetes and Australasian Paediatric Endocrine Group, ISPAD+APEG 2015. Brisbane, QLD Australia. Conference Publication: (var.pagings). 16(SUPPL. 21) (pp 108), 2015. Date of Publication: October 2015.  [Conference Abstract]  AN: 72073360 | Not relevant to the main subject/ wrong population or geographical location/ duplicate or repetition |
|  | Making strides in the management of diabetes: Breaking cultural barriers through technology.  Riley T.N.  Pediatric Diabetes. Conference: Joint Annual Conference of the International Society for Pediatric and Adolescent Diabetes and Australasian Paediatric Endocrine Group, ISPAD+APEG 2015. Brisbane, QLD Australia. Conference Publication: (var.pagings). 16(SUPPL. 21) (pp 101), 2015. Date of Publication: October 2015.  [Conference Abstract]  AN: 72073344 | Not relevant to the main subject/ wrong population or geographical location/ duplicate or repetition |
|  | Smartphone apps and dietary counseling: How interesting in the management of patients in primary care medicine?.  Amstutz D., Da Costa D., Rolet S., Golard I., Jackson Y.  Praxis. Conference: SGIM Jahresversammlung 2015. Basel Switzerland. Conference Publication: (var.pagings). 104(SUPPL. 1) (pp 96), 2015. Date of Publication: May 2015.  [Conference Abstract]  AN: 71977044 | Not relevant to the main subject/ wrong population or geographical location/ duplicate or repetition |
|  | Mobile technology and self-management support for people with diabetes and periodontitis: Results of the oral health buddy study (OH Buddy).  Piatt G., Ly E., Abelson J., Krenz C., Kraay A., Jones D., Ojo A., Chatterjee S., Neighbors B., Neighbors H.  Diabetes. Conference: 75th Scientific Sessions of the American Diabetes Association. Boston, MA United States. Conference Publication: (var.pagings). 64(SUPPL. 1) (pp A228-A229), 2015. Date of Publication: June 2015.  [Conference Abstract]  AN: 71940707 | Not relevant to the main subject/ wrong population or geographical location/ duplicate or repetition |
|  | Implementation of tele-ophthalmology for diabetic patients in a resource-limited setting in sub-saharan africa.  Matimba A., Woodward R.L.M., Gwanzura L., Mangwiro J.C., Guramatunhu S.  Diabetes. Conference: 75th Scientific Sessions of the American Diabetes Association. Boston, MA United States. Conference Publication: (var.pagings). 64(SUPPL. 1) (pp A170), 2015. Date of Publication: June 2015.  [Conference Abstract]  AN: 71940478 | Not relevant to the main subject/ wrong population or geographical location/ duplicate or repetition |
|  | Effectiveness of a scalable telemedicine intervention for veterans with persistent poor diabetes control.  Crowley M.J., Edelman D., Mcandrew A.T., Kistler S., Danus S., Webb J.A., Zanga J., Sanders L.L., Coffman C.J., Jackson G.L., Bosworth H.B.  Diabetes. Conference: 75th Scientific Sessions of the American Diabetes Association. Boston, MA United States. Conference Publication: (var.pagings). 64(SUPPL. 1) (pp A80), 2015. Date of Publication: June 2015.  [Conference Abstract]  AN: 71940126 | Not relevant to the main subject/ wrong population or geographical location/ duplicate or repetition |
|  | Mobile technology access, usage, and attitudes among low-income, minority individuals in East Harlem, NY Victoria.  Mayer L., Fei K., Buquez B., Negron R., Simon E.P., Horowitz C.R.  Journal of General Internal Medicine. Conference: 38th Annual Meeting of the Society of General Internal Medicine. Toronto, ON Canada. Conference Publication: (var.pagings). 30(SUPPL. 2) (pp S210), 2015. Date of Publication: April 2015.  [Conference Abstract]  AN: 71877804 | Not relevant to the main subject/ wrong population or geographical location/ duplicate or repetition |
|  | Association between diabetes self-care and perceived support in a sample of low-income African Americans.  Payne B.D., Oster R., Shelley J.P., A. Agne A., Cherrington A.  Journal of General Internal Medicine. Conference: 38th Annual Meeting of the Society of General Internal Medicine. Toronto, ON Canada. Conference Publication: (var.pagings). 30(SUPPL. 2) (pp S103), 2015. Date of Publication: April 2015.  [Conference Abstract]  AN: 71877571 | Not relevant to the main subject/ wrong population or geographical location/ duplicate or repetition |
|  | Improving medication adherence in hypertension using home measured blood pressure and telemedicine reporting to modify patient and physician behavior.  Peters A., Rakita V., Homko C., Kothapalli P., Bove A.  Journal of the American College of Cardiology. Conference: 64th Annual Scientific Session of the American College of Cardiology and i2 Summit: Innovation in Intervention, ACC.15. San Diego, CA United States. Conference Publication: (var.pagings). 65(10 SUPPL. 1) (pp A1395), 2015. Date of Publication: 17 Mar 2015.  [Conference Abstract]  AN: 71834452 | Not relevant to the main subject/ wrong population or geographical location/ duplicate or repetition |
|  | Diabetes Connect: Developing a Mobile Health Intervention to Link Diabetes Community Health Workers With Primary Care.  Cherrington A.L., Agne A.A., Lampkin Y., Birl A., Shelton T.C., Guzman A., Willig J.H.  The Journal of ambulatory care management. 38(4) (pp 333-345), 2015. Date of Publication: 01 Oct 2015.  [Article]  AN: 616663612 | Not relevant to the main subject/ wrong population or geographical location/ duplicate or repetition |
|  | Implementation of the HealthKick intervention in primary schools in low-income settings in the Western Cape Province, South Africa: a process evaluation.  de Villiers A., Steyn N.P., Draper C.E., Hill J., Dalais L., Fourie J., Lombard C., Barkhuizen G., Lambert E.V.  BMC public health. 15 (pp 818), 2015. Date of Publication: 22 Aug 2015.  [Article]  AN: 615762691 | Not relevant to the main subject/ wrong population or geographical location/ duplicate or repetition |
|  | Telehealth program for type 2 diabetes: usability, satisfaction, and clinical usefulness in an urban community health center.  Welch G., Balder A., Zagarins S.  Telemedicine journal and e-health : the official journal of the American Telemedicine Association. 21(5) (pp 395-403), 2015. Date of Publication: 01 May 2015.  [Article]  AN: 614957468 | Not relevant to the main subject/ wrong population or geographical location/ duplicate or repetition |
|  | Community Interventions to Improve Glycemic Control in African Americans with Type 2 Diabetes: A Systemic Review.  Smalls B.L., Walker R.J., Bonilha H.S., Campbell J.A., Egede L.E.  Global journal of health science. 7(5) (pp 171-182), 2015. Date of Publication: 2015.  [Review]  AN: 606783643 | Not relevant to the main subject/ wrong population or geographical location/ duplicate or repetition |
|  | Management of NCD in low- And middle-income countries.  Checkley W., Ghannem H., Irazola V., Kimaiyo S., Levitt N.S., Miranda J.J., Niessen L., Prabhakaran D., Rabadan-Diehl C., Ramirez-Zea M., Rubinstein A., Sigamani A., Smith R., Tandon N., Wu Y., Xavier D., Yan L.L., GRAND South Network, National Heart, Lung, and Blood Institute Centers of Excellence  Global Heart. 9(4) (pp 431-443), 2014. Date of Publication: 01 Dec 2014.  [Review]  AN: 601313206 | Not relevant to the main subject/ wrong population or geographical location/ duplicate or repetition |
|  | Rationale and design of the Miami Healthy Heart Initiative: A randomized controlled study of a community health worker intervention among Latino patients with poorly controlled diabetes.  Carrasquillo O., Patberg E., Alonzo Y., Li H., Kenya S.  International Journal of General Medicine. 7 (pp 115-125), 2014. Date of Publication: 27 Feb 2014.  [Article]  AN: 372512347 | Not relevant to the main subject/ wrong population or geographical location/ duplicate or repetition |
|  | Technology-Intensified Diabetes Education Study (TIDES) in African Americans with type 2 diabetes: Study protocol for a randomized controlled trial.  Williams J.S., Lynch C.P., Knapp R.G., Egede L.E.  Trials. 15(1) (no pagination), 2014. Article Number: 460. Date of Publication: November 25, 2014.  [Article]  AN: 605028710 | Not relevant to the main subject/ wrong population or geographical location/ duplicate or repetition |
|  | Patient Characteristics and Participation in a Genetic Study.  Amiri L., Cassidy-Bushrow A.E., Dakki H., Li J., Wells K., Oliveria S.A., Yood M.U., Thomas A., Lanfear D.E.  Journal of Investigative Medicine. 62(1) (pp 26-32), 2014. Date of Publication: 2014.  [Article]  AN: 613758808 | Not relevant to the main subject/ wrong population or geographical location/ duplicate or repetition |
|  | Patient characteristics and participation in a genetic study: A type 2 diabetes cohort.  Amiri L., Cassidy-Bushrow A.E., Dakki H., Li J., Wells K., Oliveria S.A., Yood M.U., Thomas A., Lanfear D.E.  Journal of Investigative Medicine. 62(1) (pp 26-32), 2014. Date of Publication: January 2014.  [Article]  AN: 372577831 | Not relevant to the main subject/ wrong population or geographical location/ duplicate or repetition |
|  | How do mobile phone diabetes programs drive behavior change? Evidence from a mixed methods observational cohort study.  Nundy S., Mishra A., Hogan P., Lee S.M., Solomon M.C., Peek M.E.  The Diabetes educator. 40(6) (pp 806-819), 2014. Date of Publication: 01 Nov 2014.  [Article]  AN: 609437118 | Not relevant to the main subject/ wrong population or geographical location/ duplicate or repetition |
|  | Bioequivalence of Glucophage (metformin) tablets from Europe and the united states tested in healthy volunteers.  Friedrich C., Brand T., Ring A., Meinicke T.  Journal of Bioequivalence and Bioavailability. 6(2) (pp 61-66), 2014. Date of Publication: 2014.  [Article]  AN: 372957727 | Not relevant to the main subject/ wrong population or geographical location/ duplicate or repetition |
|  | Role of health in predicting moves to poor neighborhoods among Hurricane Katrina survivors.  Arcaya M.C., Subramanian S.V., Rhodes J.E., Waters M.C.  Proceedings of the National Academy of Sciences of the United States of America. 111(46) (pp 16246-16253), 2014. Date of Publication: 18 Nov 2014.  [Article]  AN: 600459786 | Not relevant to the main subject/ wrong population or geographical location/ duplicate or repetition |
|  | Potential efficiency benefits of nonmydriatic ultrawide field retinal imaging in an ocular telehealth diabetic retinopathy program.  Silva P.S., Cavallerano J.D., Tolls D., Omar A., Thakore K., Patel B., Sehizadeh M., Tolson A.M., Sun J.K., Aiello L.M., Aiello L.P.  Diabetes Care. 37(1) (pp 50-55), 2014. Date of Publication: January 2014.  [Article]  AN: 372111949 | Not relevant to the main subject/ wrong population or geographical location/ duplicate or repetition |
|  | Means of communication for an early detection of diabetic nephropathy among the diabetics followed in the academic hospital of Cotonou. Moyens de communication en vue du depistage precoce de la nephropathie diabetique chez les diabetiques suivis a l'hopital universitaire de Cotonou <Moyens de communication en vue du depistage precoce de la nephropathie diabetique chez les diabetiques suivis a l'hopital universitaire de Cotonou.>  Vigan J., Adja E., Zannou J., Agboton B.L., Kerekou C.A., Amoussou-Guenou D., Zannou M.D., Djrolo F.  Nephrologie et Therapeutique. 10(3) (pp 165-169), 2014. Date of Publication: June 2014.  [Article]  AN: 53130139 | Not relevant to the main subject/ wrong population or geographical location/ duplicate or repetition |
|  | Effect of food and tablet-dissolution characteristics on the bioavailability of a linagliptin fixed-dose combination with metformin: Evidence from two randomized trials.  Metzmann K., Schnell D., Jungnik A., Ring A., Theodor R., Hohl K., Meinicke T., Friedrich C.  International Journal of Clinical Pharmacology and Therapeutics. 52(7) (pp 549-563), 2014. Date of Publication: July 2014.  [Article]  AN: 373481867 | Not relevant to the main subject/ wrong population or geographical location/ duplicate or repetition |
|  | Linagliptin fixed-dose combination with metformin is bioequivalent to co- Administration of linagliptin and metformin as individual tablets.  Buschke S., Ring A., Friedrich C., Metzmann K., Meinicke T.  International Journal of Clinical Pharmacology and Therapeutics. 52(7) (pp 537-548), 2014. Date of Publication: July 2014.  [Article]  AN: 373481866 | Not relevant to the main subject/ wrong population or geographical location/ duplicate or repetition |
|  | Design and evaluation of bilayer tablets of glimepiride and metformin hydrochloride with combination of hydrophilic and hydrophobic polymers by hot melt extrusion.  Wagh K.S., Kale S.S., Mali K.D., Patil S.K., Baviskar D.T.  Asian Journal of Pharmaceutical and Clinical Research. 7(5) (pp 300-304), 2014. Date of Publication: 01 Nov 2014.  [Article]  AN: 601157808 | Not relevant to the main subject/ wrong population or geographical location/ duplicate or repetition |
|  | What works for obesity prevention and treatment in black Americans? Research directions.  Kumanyika S.K., Whitt-Glover M.C., Haire-Joshu D.  Obesity Reviews. 15(Supplement4) (pp 204-212), 2014. Date of Publication: 2014.  [Review]  AN: 600319751 | Not relevant to the main subject/ wrong population or geographical location/ duplicate or repetition |
|  | Preventing diabetic blindness: A priority for South Africa.  Hofman K.J., Cook C., Levitt N.  South African Medical Journal. 104(10) (pp 661-662), 2014. Date of Publication: 01 Oct 2014.  [Article]  AN: 604804913 | Not relevant to the main subject/ wrong population or geographical location/ duplicate or repetition |
|  | An ICT-based diabetes management system tested for health care delivery in the African context.  Takenga C., Berndt R.-D., Musongya O., Kitero J., Katoke R., Molo K., Kazingufu B., Meni M., Vikandy M., Takenga H.  International Journal of Telemedicine and Applications. 2014 (no pagination), 2014. Article Number: 437307. Date of Publication: 2014.  [Article]  AN: 604867282 | Included |
|  | Lixisenatide, a drug developed to treat type 2 diabetes, shows neuroprotective effects in a mouse model of Alzheimer's disease.  McClean P.L., Holscher C.  Neuropharmacology. 86 (pp 241-258), 2014. Date of Publication: 01 Nov 2014.  [Article]  AN: 600110279 | Not relevant to the main subject/ wrong population or geographical location/ duplicate or repetition |
|  | Smartphone use by patients with diabetes.  Ross A.L., Boyd-Woschinko G.S., Kaiser D.L., Alifarag A.M., King D., Diefenbach M., Tamler R.  Endocrine Reviews. Conference: 96th Annual Meeting and Expo of the Endocrine Society, ENDO 2014. Chicago, IL United States. Conference Publication: (var.pagings). 35(SUPPL. 3) (no pagination), 2014. Date of Publication: 2014.  [Conference Abstract]  AN: 72336986 | Not relevant to the main subject/ wrong population or geographical location/ duplicate or repetition |
|  | Late-Breaking Abstracts American Society of Hypertension 29th Annual Scientific Meeting.  Anonymous  Journal of the American Society of Hypertension. Conference: 29th Annual Scientific Meeting and Exposition of the American Society of Hypertension Inc., ASH 2014. New York, NY United States. Conference Publication: (var.pagings). 8(SUPPL. 1) (no pagination), 2014. Date of Publication: August 2014.  [Conference Review]  AN: 72308407 | Not relevant to the main subject/ wrong population or geographical location/ duplicate or repetition |
|  | Improved access of diabetes care in low resource area by mobile care delivery.  Hind E.A.E., Eltom M., Ostenson C., Johansson P., Wahlstrom R.  Diabetes Research and Clinical Practice. Conference: 10th International Diabetes Federation-Western Pacific Region Congress and the 6th AASD Scientific Meeting. Singapore Singapore. Conference Publication: (var.pagings). 106(SUPPL. 1) (pp S96), 2014. Date of Publication: November 2014.  [Conference Abstract]  AN: 71824876 | Not relevant to the main subject/ wrong population or geographical location/ duplicate or repetition |
|  | Diabetes care via telemedicine in South Africa.  Segal D., Rowe G., Johnson R., Shannon U., Plaatjie S., Schwulst A.  Journal of Endocrinology, Metabolism and Diabetes of South Africa. Conference: 49th Congress of the Society for Endocrinology, Metabolism and Diabetes of South Africa, SEMDSA. Durban South Africa. Conference Publication: (var.pagings). 19(1 2014) (pp 31), 2014. Date of Publication: 2014.  [Conference Abstract]  AN: 71790234 | Not relevant to the main subject/ wrong population or geographical location/ duplicate or repetition |
|  | Brain amyloidosis in streptozocin-induced diabetes in the 5XFAD mouse model of Alzheimer's disease.  Sonn K., Zharkovsky A.  European Neuropsychopharmacology. Conference: 27th European College of Neuropsychopharmacology, ECNP Congress. Berlin Germany. Conference Publication: (var.pagings). 24(SUPPL. 2) (pp S632), 2014. Date of Publication: October 2014.  [Conference Abstract]  AN: 71641637 | Not relevant to the main subject/ wrong population or geographical location/ duplicate or repetition |
|  | Effect of allopurinol versus angiotensin converting enzyme inhibitors in decreasing microalbuminuria in type I diabetic patients.  El-Samahy M.H., Elbarbary N.S., Afify M.A.-A., Sallam D.E.  Pediatric Diabetes. Conference: 40th Annual Conference of the International Society for Pediatric and Adolescent Diabetes, ISPAD 2014. Toronto, ON Canada. Conference Publication: (var.pagings). 15(SUPPL. 19) (pp 84), 2014. Date of Publication: September 2014.  [Conference Abstract]  AN: 71619679 | Not relevant to the main subject/ wrong population or geographical location/ duplicate or repetition |
|  | Group B streptococcal lead-associated endocarditis in a poorly controlled diabetic.  Karagodin I., Kuppalli K., Berger M.  Journal of General Internal Medicine. Conference: 37th Annual Meeting of the Society of General Internal Medicine, SGIM 2014. San Diego, CA United States. Conference Publication: (var.pagings). 29(SUPPL. 1) (pp S360), 2014. Date of Publication: April 2014.  [Conference Abstract]  AN: 71495558 | Not relevant to the main subject/ wrong population or geographical location/ duplicate or repetition |
|  | Race/ethnic disparities in weight and glycemia in older adults receiving lifestyle interventions via peer-leaders with or without mobile enhancement for diabetes prevention and management.  Dang S., Oropesa L., Byrne M.M., Gutt M., Andrade F., Guanipa C., Sorial A., Pelaez M., Schwarzberg R., Roos B., Valencia W., Florez H.  Journal of the American Geriatrics Society. Conference: 2014 Annual Scientific Meeting of the American Geriatrics Society. Orlando, FL United States. Conference Publication: (var.pagings). 62(SUPPL. 1) (pp S216), 2014. Date of Publication: March 2014.  [Conference Abstract]  AN: 71470745 | Not relevant to the main subject/ wrong population or geographical location/ duplicate or repetition |
|  | Confessions of an outer london HIV clinic: Characteristics of those who chose not to disclose their HIV status to general practice.  Byrne R., West R., Daniels D., Forbes K.  HIV Medicine. Conference: 3rd Joint Conference of the British HIV Association, BHIVA with the British Association for Sexual Health and HIV, BASHH. Liverpool United Kingdom. Conference Publication: (var.pagings). 15(SUPPL. 3) (pp 20), 2014. Date of Publication: April 2014.  [Conference Abstract]  AN: 71431872 | Not relevant to the main subject/ wrong population or geographical location/ duplicate or repetition |
|  | Interest in and use of smart phone technology for diabetes self-management in diverse populations.  Humble J., Debon M., Krukowski R., Womack C., Bailey J.E.  Journal of Investigative Medicine. Conference: American Federation for Medical Research Southern Regional Meeting, AFMR 2014. New Orleans, LA United States. Conference Publication: (var.pagings). 62(2) (pp 584), 2014. Date of Publication: February 2014.  [Conference Abstract]  AN: 71392324 | Not relevant to the main subject/ wrong population or geographical location/ duplicate or repetition |
|  | Management of type 2 diabetes in clinical practices in sub-Saharan Africa: Results of the AMAR-AFO study in Senegal and Ivory Cost.  Diop S.N., Lokrou A., Diedhiou D., Adoueni V.-K.  Diabetes Research and Clinical Practice. Conference: 2nd African Diabetes Congress 2014. Yaounde Cameroon. Conference Publication: (var.pagings). 103(SUPPL. 1) (pp S61), 2014. Date of Publication: February 2014.  [Conference Abstract]  AN: 71385865 | Not relevant to the main subject/ wrong population or geographical location/ duplicate or repetition |
|  | Social media and diabetes care in Africa: The case of Cameroon.  Tamghe F., Katte J.C., Okondoua A., Fokapu P., Youmbi B., Kouam Kouam C., Fetse G., Dehayem M., Sobngwi E.  Diabetes Research and Clinical Practice. Conference: 2nd African Diabetes Congress 2014. Yaounde Cameroon. Conference Publication: (var.pagings). 103(SUPPL. 1) (pp S59), 2014. Date of Publication: February 2014.  [Conference Abstract]  AN: 71385861 | Not relevant to the main subject/ wrong population or geographical location/ duplicate or repetition |
|  | Social media and diabetes care in Africa.  Adejumo O.  Diabetes Research and Clinical Practice. Conference: 2nd African Diabetes Congress 2014. Yaounde Cameroon. Conference Publication: (var.pagings). 103(SUPPL. 1) (pp S58-S59), 2014. Date of Publication: February 2014.  [Conference Abstract]  AN: 71385860 | Not relevant to the main subject/ wrong population or geographical location/ duplicate or repetition |
|  | Primary care physicians and patients factors influencing eye care provision and utilisation in a group of diabetic patients.  Jingi A., Ebana-Mvogo C., Ellong A.  Diabetes Research and Clinical Practice. Conference: 2nd African Diabetes Congress 2014. Yaounde Cameroon. Conference Publication: (var.pagings). 103(SUPPL. 1) (pp S50-S51), 2014. Date of Publication: February 2014.  [Conference Abstract]  AN: 71385846 | Not relevant to the main subject/ wrong population or geographical location/ duplicate or repetition |
|  | Starting a hospital-based diabetes management program in a semi-urban setting: From community action to implementation.  Katte J.C., Fetse G., Kouam Kouam C., Dehayem M., Sobngwi E.  Diabetes Research and Clinical Practice. Conference: 2nd African Diabetes Congress 2014. Yaounde Cameroon. Conference Publication: (var.pagings). 103(SUPPL. 1) (pp S41), 2014. Date of Publication: February 2014.  [Conference Abstract]  AN: 71385827 | Not relevant to the main subject/ wrong population or geographical location/ duplicate or repetition |
|  | Radio-clinical, biological and evolutional characteristics of pulmonary tuberculosis in patients with diabetes mellitus.  Tchankam C., Pefura Yone E.W., Nouedoui C., Kuaban C.  Diabetes Research and Clinical Practice. Conference: 2nd African Diabetes Congress 2014. Yaounde Cameroon. Conference Publication: (var.pagings). 103(SUPPL. 1) (pp S18), 2014. Date of Publication: February 2014.  [Conference Abstract]  AN: 71385786 | Not relevant to the main subject/ wrong population or geographical location/ duplicate or repetition |
|  | Nanocomplexes of an insulinotropic drug: Optimization, microparticle formation, and antidiabetic activity in rats.  Elmowafy E., Osman R., El-Shamy A.H., Awad G.A.S.  International Journal of Nanomedicine. 9(1) (pp 4449-4465), 2014. Date of Publication: 2014.  [Article]  AN: 2004242579 | Not relevant to the main subject/ wrong population or geographical location/ duplicate or repetition |
|  | Lixisenatide, a drug developed to treat type 2 diabetes, shows neuroprotective effects in a mouse model of Alzheimer's disease.  McClean P.L., Holscher C.  Neuropharmacology. 86 (pp 241-258), 2014. Date of Publication: 01 Nov 2014.  [Article]  AN: 609264702 | Not relevant to the main subject/ wrong population or geographical location/ duplicate or repetition |
|  | Lixisenatide, a drug developed to treat type 2 diabetes, shows neuroprotective effects in a mouse model of Alzheimer's disease.  McClean P.L., Holscher C.  Neuropharmacology. 86 (pp 241-258), 2014. Date of Publication: November 2014.  [Article]  AN: 373801271 | Not relevant to the main subject/ wrong population or geographical location/ duplicate or repetition |
|  | Role of mobile phone technology in health education in Asian and African countries: a systematic review.  Sahu M., Grover A., Joshi A.  International journal of electronic healthcare. 7(4) (pp 269-286), 2014. Date of Publication: 2014.  [Review]  AN: 603674520 | Not relevant to the main subject/ wrong population or geographical location/ duplicate or repetition |
|  | Diabetes in patients with severe mental illness: A review of caseload in an inner city community diabetes service using case management and partnership working with mental health services.  Ridout J., Wignall A., Asumah M., Jones M., Gable D.  Diabetic Medicine. Conference: Diabetes UK Professional Conference 2013. Manchester United Kingdom. Conference Publication: (var.pagings). 30(SUPPL. 1) (pp 180), 2013. Date of Publication: March 2013.  [Conference Abstract]  AN: 71019278 | Not relevant to the main subject/ wrong population or geographical location/ duplicate or repetition |
|  | Managing hypertension in urban underserved subjects using telemedicine-A clinical trial.  Bove A.A., Homko C.J., Santamore W.P., Kashem M., Kerper M., Elliott D.J.  American Heart Journal. 165(4) (pp 615-621), 2013. Date of Publication: April 2013.  [Article]  AN: 52478501 | Not relevant to the main subject/ wrong population or geographical location/ duplicate or repetition |
|  | Developing a behavioral model for mobile phone-based diabetes interventions.  Nundy S., Dick J.J., Solomon M.C., Peek M.E.  Patient Education and Counseling. 90(1) (pp 125-132), 2013. Date of Publication: January 2013.  [Article]  AN: 52251091 | Not relevant to the main subject/ wrong population or geographical location/ duplicate or repetition |
|  | The effectiveness of text messaging programs on adherence to treatment regimens among adults aged 18 to 45 years diagnosed with asthma: A systematic review protocol.  DiBello K.K., Boyar K.L., Abrenica S.C., Worral P.S.  JBI Database of Systematic Reviews and Implementation Reports. 11(8) (pp 170-185), 2013. Date of Publication: 2013.  [Article]  AN: 369813562 | Not relevant to the main subject/ wrong population or geographical location/ duplicate or repetition |
|  | Review of infectious diseases applications for iPhone/iPad and android: From pocket to patient.  Moodley A., Mangino J.E., Goff D.A.  Clinical Infectious Diseases. 57(8) (pp 1145-1154), 2013. Date of Publication: 15 Oct 2013.  [Article]  AN: 369941248 | Not relevant to the main subject/ wrong population or geographical location/ duplicate or repetition |
|  | Mobilizing your medications: An automated medication reminder application for mobile phones and hypertension medication adherence in a high-risk urban population.  Patel S., Jacobus-Kantor L., Marshall L., Ritchie C., Kaplinski M., Khurana P.S., Katz R.J.  Journal of Diabetes Science and Technology. 7(3) (pp 630-639), 2013. Date of Publication: May 2013.  [Conference Paper]  AN: 373114619 | Not relevant to the main subject/ wrong population or geographical location/ duplicate or repetition |
|  | Ocular disease, knowledge and technology applications in patients with diabetes.  Threatt J., Williamson J.F., Huynh K., Davis R.M.  American Journal of the Medical Sciences. 345(4) (pp 266-270), 2013. Date of Publication: April 2013.  [Conference Paper]  AN: 368723516 | Not relevant to the main subject/ wrong population or geographical location/ duplicate or repetition |
|  | Rationale, design, and methodology for the optimizing outcomes in women with gestational diabetes mellitus and their infants study.  Berry D.C., Neal M., Hall E.G., Schwartz T.A., Verbiest S., Bonuck K., Goodnight W., Brody S., Dorman K.F., Menard M.K., Stuebe A.M.  BMC Pregnancy and Childbirth. 13 (no pagination), 2013. Article Number: 184. Date of Publication: 10 Oct 2013.  [Article]  AN: 52811206 | Not relevant to the main subject/ wrong population or geographical location/ duplicate or repetition |
|  | Sucralose.  AlDeeb O.A.A., Mahgoub H., Foda N.H.  Profiles of Drug Substances, Excipients and Related Methodology. 38 (pp 423-462), 2013. Date of Publication: 2013.  [Article]  AN: 368991220 | Not relevant to the main subject/ wrong population or geographical location/ duplicate or repetition |
|  | Improving diabetic retinopathy screening in Africa: Patient satisfaction with teleophthalmology versus ophthalmologist-based screening.  Kurji K., Kiage D., Rudnisky C.J., Damji K.F.  Middle East African Journal of Ophthalmology. 20(1) (pp 56-60), 2013. Date of Publication: January-March 2013.  [Article]  AN: 369077740 | Not relevant to the main subject/ wrong population or geographical location/ duplicate or repetition |
|  | The cholesterol, hypertension, and glucose education (CHANGE) study: Results from a randomized controlled trial in African Americans with diabetes.  Crowley M.J., Powers B.J., Olsen M.K., Grubber J.M., Koropchak C., Rose C.M., Gentry P., Bowlby L., Trujillo G., Maciejewski M.L., Bosworth H.B.  American Heart Journal. 166(1) (pp 179-186.e2), 2013. Date of Publication: July 2013.  [Article]  AN: 52586061 | Not relevant to the main subject/ wrong population or geographical location/ duplicate or repetition |
|  | Efficiency of an intervention package for arterial hypertension comprising telemanagement in a Cameroonian rural setting: The TELEMED-CAM study.  Kingue S., Angandji P., Menanga A.P., Ashutantang G., Sobngwi E., Dossou-Yovo R.A., Kaze F.F., Kengne A.P., Dzudie A., Ndobo P., Muna W.  Pan African Medical Journal. 15 (no pagination), 2013. Article Number: 153. Date of Publication: 2013.  [Article]  AN: 370235689 | Not relevant to the main subject/ wrong population or geographical location/ duplicate or repetition |
|  | The muranga teleophthalmology study: Comparison of virtual (teleglaucoma) with in-person clinical assessment to diagnose glaucoma.  Kiage D., Kherani I.N., Gichuhi S., Damji K.F., Nyenze M.  Middle East African Journal of Ophthalmology. 20(2) (pp 150-157), 2013. Date of Publication: April-June 2013.  [Article]  AN: 368829363 | Not relevant to the main subject/ wrong population or geographical location/ duplicate or repetition |
|  | Preventing diabetes blindness: Cost effectiveness of a screening programme using digital non-mydriatic fundus photography for diabetic retinopathy in a primary health care setting in South Africa.  Khan T., Bertram M.Y., Jina R., Mash B., Levitt N., Hofman K.  Diabetes Research and Clinical Practice. 101(2) (pp 170-176), 2013. Date of Publication: August 2013.  [Article]  AN: 52644580 | Not relevant to the main subject/ wrong population or geographical location/ duplicate or repetition |
|  | A systematic review of IT for diabetes self-management: Are we there yet?.  El-Gayar O., Timsina P., Nawar N., Eid W.  International Journal of Medical Informatics. 82(8) (pp 637-652), 2013. Date of Publication: August 2013.  [Review]  AN: 52644232 | Not relevant to the main subject/ wrong population or geographical location/ duplicate or repetition |
|  | Local knowledge, use pattern and geographical distribution of Moringa oleifera Lam. (Moringaceae) in Nigeria.  Popoola J.O., Obembe O.O.  Journal of Ethnopharmacology. 150(2) (pp 682-691), 2013. Date of Publication: 25 Nov 2013.  [Article]  AN: 52827305 | Not relevant to the main subject/ wrong population or geographical location/ duplicate or repetition |
|  | 2nd National Congress on Medicinal Plants.  Anonymous  Iranian Journal of Pharmaceutical Research. Conference: 2nd National Congress on Medicinal Plants. (2nd). Tehran Iran, Islamic Republic of. Conference Publication: (1497 pages). 12(Supplement 2) (pp 43), 2013. Date of Publication: Oct 2013.  [Conference Review]  AN: 75003105 | Not relevant to the main subject/ wrong population or geographical location/ duplicate or repetition |
|  | Sudan: Childhood diabetes teams.  Abdullah M.A.  Pediatric Diabetes. Conference: 39th Annual Conference of the International Society for Pediatric and Adolescent Diabetes, ISPAD 2013. Gothenburg Sweden. Conference Publication: (var.pagings). 14(SUPPL. 18) (pp 16), 2013. Date of Publication: October 2013.  [Conference Abstract]  AN: 71557159 | Not relevant to the main subject/ wrong population or geographical location/ duplicate or repetition |
|  | Right atrial thrombus associated with indwelling dialysis catheters.  Ahuja J.K.  Journal of General Internal Medicine. Conference: 36th Annual Meeting of the Society of General Internal Medicine, SGIM 2013. Denver, CO United States. Conference Publication: (var.pagings). 28(SUPPL. 1) (pp S383), 2013. Date of Publication: June 2013.  [Conference Abstract]  AN: 71293522 | Not relevant to the main subject/ wrong population or geographical location/ duplicate or repetition |
|  | Comparison of the antiplatelet effect of crushed clopidogrel versus whole tablet in diabetic patients presenting with an acute coronary syndrome.  Khochtali I., Addad F., Weslati C., Mahjoub S., Kachboura S.  Diabetes. Conference: 73rd Scientific Sessions of the American Diabetes Association. Chicago, IL United States. Conference Publication: (var.pagings). 62(SUPPL. 1) (pp A597), 2013. Date of Publication: July 2013.  [Conference Abstract]  AN: 71288735 | Not relevant to the main subject/ wrong population or geographical location/ duplicate or repetition |
|  | How can mobile phone diabetes programs change behavior?.  Nundy S., Hogan P., Dick J.J., Goddu A.P., Solomon M.C., Chin M.H., Peek M.E.  Diabetes. Conference: 73rd Scientific Sessions of the American Diabetes Association. Chicago, IL United States. Conference Publication: (var.pagings). 62(SUPPL. 1) (pp A211), 2013. Date of Publication: July 2013.  [Conference Abstract]  AN: 71287274 | Not relevant to the main subject/ wrong population or geographical location/ duplicate or repetition |
|  | Recruiting for a text message intervention for rural african-american women with diabetes.  May N.B., Winstead-Derlega C., Zimmerman P., Plews-Ogan M., Nees M., Dillingham R., Nadkarni M., Green R.  Diabetes. Conference: 73rd Scientific Sessions of the American Diabetes Association. Chicago, IL United States. Conference Publication: (var.pagings). 62(SUPPL. 1) (pp A178), 2013. Date of Publication: July 2013.  [Conference Abstract]  AN: 71287145 | Not relevant to the main subject/ wrong population or geographical location/ duplicate or repetition |
|  | Improving clinical outcomes and cost-effectiveness in SCI through clinical video telehealth (CVT): A case report.  Quinones E.C., Jimenez Z., Coleman B.R.  PM and R. Conference: 2013 Annual Assembly of the American Academy of Physical Medicine and Rehabilitation. National Harbor, MD United States. Conference Publication: (var.pagings). 5(9 SUPPL. 1) (pp S242), 2013. Date of Publication: September 2013.  [Conference Abstract]  AN: 71205989 | Not relevant to the main subject/ wrong population or geographical location/ duplicate or repetition |
|  | Evaluation of cardiac implantable electronic device infection outcomes from a large end stage renal disease database.  Guha A., Maddox W.R., Colombo R., Nahman Jr. N.S., Kintziger K., Kheda M., Sorrentino R.A.  Heart Rhythm. Conference: 34th Annual Scientific Sessions of the Heart Rhythm Society, Heart Rhythm 2013. Denver, CO United States. Conference Publication: (var.pagings). 10(9) (pp 1422), 2013. Date of Publication: September 2013.  [Conference Abstract]  AN: 71193263 | Not relevant to the main subject/ wrong population or geographical location/ duplicate or repetition |
|  | Overweight and obesity: A comparative study between predominantly stationary market women and their mobile counterparts in tamale metropolis of Ghana.  Aryee P., Helegbe G., Ahmed A., Kasim A.  Annals of Nutrition and Metabolism. Conference: 20th International Congress of Nutrition. Granada Spain. Conference Publication: (var.pagings). 63(SUPPL. 1) (pp 1313), 2013. Date of Publication: 2013.  [Conference Abstract]  AN: 71180240 | Not relevant to the main subject/ wrong population or geographical location/ duplicate or repetition |
|  | 10th International Congress on Adolescent Health.  Anonymous  Turkish Archives of Pediatrics. Conference: 10th International Congress on Adolescent Health. (10th). Istanbul Turkey. Conference Publication: Nil Arisoy (139 pages). 48(Suppl 2) (pp 1), 2013. Date of Publication: June 2013.  [Conference Review]  AN: 75000411 | Not relevant to the main subject/ wrong population or geographical location/ duplicate or repetition |
|  | Superficialized brachiobasilic fistula formed as a 1-step or as a second procedure. what is the best?.  Boubaker K., Kaaroud H., Kheder A.  Nephrology Dialysis Transplantation. Conference: 50th ERA-EDTA Congress. Istanbul Turkey. Conference Publication: (var.pagings). 28(SUPPL. 1) (pp i236), 2013. Date of Publication: May 2013.  [Conference Abstract]  AN: 71075707 | Not relevant to the main subject/ wrong population or geographical location/ duplicate or repetition |
|  | Adherence to therapy among diabetes patients in a Niger delta referral center.  Suleiman I.A., Siasia W.O., Egbesu I.E.  Value in Health. Conference: ISPOR 18th Annual International Meeting. New Orleans, LA United States. Conference Publication: (var.pagings). 16(3) (pp A167-A168), 2013. Date of Publication: May 2013.  [Conference Abstract]  AN: 71060721 | Not relevant to the main subject/ wrong population or geographical location/ duplicate or repetition |
|  | Managing hypertension in urban underserved subjects using telemedicine: A clinical trial.  Bove A.A., Homko C., Santamore W., Kashem M., Kerper M., Elliott D.  Journal of the American College of Cardiology. Conference: 62nd Annual Scientific Session of the American College of Cardiology and i2 Summit: Innovation in Intervention, ACC.13. San Francisco, CA United States. Conference Publication: (var.pagings). 61(10 SUPPL. 1) (pp E1401), 2013. Date of Publication: 12 Mar 2013.  [Conference Abstract]  AN: 71020764 | Not relevant to the main subject/ wrong population or geographical location/ duplicate or repetition |
|  | Does an abbreviated dose of clopidogrel negatively impact the light transmission aggregometry response to platelets?.  Sawasany M.  JACC: Cardiovascular Interventions. Conference: Cardiovascular Research Technologies, CRT 2013. Washington, DC United States. Conference Publication: (var.pagings). 6(2 SUPPL. 1) (pp S14), 2013. Date of Publication: February 2013.  [Conference Abstract]  AN: 71012695 | Not relevant to the main subject/ wrong population or geographical location/ duplicate or repetition |
|  | Diabetes connect: Feasibility of a peer support diabetes intervention for low-income African Americans.  Maduforo U., Falola M., Agne A., Cherrington A.  Journal of Investigative Medicine. Conference: American Federation for Medical Research Southern Regional Meeting, AFMR 2013. New Orleans, LA United States. Conference Publication: (var.pagings). 61(2) (pp 492), 2013. Date of Publication: February 2013.  [Conference Abstract]  AN: 70993348 | Not relevant to the main subject/ wrong population or geographical location/ duplicate or repetition |
|  | Acute sensineural hearing loss in streptococcus agalactiae (group b) endocarditis infection after one dose of gentamycin.  Jinenez A., Khan Y., Sachdeva B.  Journal of Investigative Medicine. Conference: American Federation for Medical Research Southern Regional Meeting, AFMR 2013. New Orleans, LA United States. Conference Publication: (var.pagings). 61(2) (pp 412), 2013. Date of Publication: February 2013.  [Conference Abstract]  AN: 70993074 | Not relevant to the main subject/ wrong population or geographical location/ duplicate or repetition |
|  | Real-time tele-monitoring of glucose as adjunct to the management of type 2 diabetes in primary care.  Heudebert A., Eichold B., Arrieta M.I., Roach D., Brown S., Hansberry S., Brye W., Steigler S., Mitchell F., Oliver B., Foreman R., Moody K., Crook E.  Journal of Investigative Medicine. Conference: American Federation for Medical Research Southern Regional Meeting, AFMR 2013. New Orleans, LA United States. Conference Publication: (var.pagings). 61(2) (pp 398), 2013. Date of Publication: February 2013.  [Conference Abstract]  AN: 70993023 | Not relevant to the main subject/ wrong population or geographical location/ duplicate or repetition |
|  | Population pharmacokinetic modeling of a novel delayed-release formulation of metformin (MetDR).  Taylor A., Chigutsa E., Monteleone J., Fineman M.  Journal of Pharmacokinetics and Pharmacodynamics. Conference: American Conference on Pharmacometrics 2013, ACoP 2013. Fort Lauderdale, FL United States. Conference Publication: (var.pagings). 40(1 SUPPL. 1) (pp S101-S102), 2013. Date of Publication: May 2013.  [Conference Abstract]  AN: 71272605 | Not relevant to the main subject/ wrong population or geographical location/ duplicate or repetition |
|  | Diabetes GPSD - An innovative method to engage people living with diabetes to behavioural change.  Lui N.O.L.  Canadian Journal of Diabetes. Conference: 16th Annual Canadian Diabetes Association/Canadian Society of Endocrinology and Metabolism Professional Conference and Annual Meetings. Montreal, QC Canada. Conference Publication: (var.pagings). 37(SUPPL. 4) (pp S18), 2013. Date of Publication: October 2013.  [Conference Abstract]  AN: 71210483 | Not relevant to the main subject/ wrong population or geographical location/ duplicate or repetition |
|  | Comparison of the antiplatelet effect of crushed clopidogrel vs. Whole tablet in diabetic patients presenting with an acute coronary syndrome.  Addad F., Oueslati C., Ibn El Hadj Z., Hammami N., Jebri F., Ben Halima A., Kammoun I., Yaalaoui S., Kachboura S.  Journal of Thrombosis and Haemostasis. Conference: 24th Congress of the International Society on Thrombosis and Haemostasis. Amsterdam Netherlands. Conference Publication: (var.pagings). 11(SUPPL. 2) (pp 628-629), 2013. Date of Publication: July 2013.  [Conference Abstract]  AN: 71208379 | Not relevant to the main subject/ wrong population or geographical location/ duplicate or repetition |
|  | Novel drug delivery system of plant extract for the management of diabetes: An antidiabetic study.  Momoh M.A., Chime S.A., Kenechukwu F.C.  Journal of Dietary Supplements. 10(3) (pp 252-263), 2013. Date of Publication: September 2013.  [Article]  AN: 369640169 | Not relevant to the main subject/ wrong population or geographical location/ duplicate or repetition |
|  | Adherence to diabetes self care for white, African-American and Hispanic American telemedicine participants: 5 year results from the IDEATel project.  Trief P.M., Izquierdo R., Eimicke J.P., Teresi J.A., Goland R., Palmas W., Shea S., Weinstock R.S.  Ethnicity and Health. 18(1) (pp 83-96), 2013. Date of Publication: 01 Feb 2013.  [Article]  AN: 368712083 | Not relevant to the main subject/ wrong population or geographical location/ duplicate or repetition |
|  | An e-health intervention for increasing diabetes knowledge in African Americans.  Moussa M., Sherrod D., Choi J.  International Journal of Nursing Practice. 19(SUPPL.3) (pp 36-43), 2013. Date of Publication: September 2013.  [Article]  AN: 1369949436 | Not relevant to the main subject/ wrong population or geographical location/ duplicate or repetition |
|  | Influence of patient characteristics on assessment of diabetes self-management support.  Wallace A., Perkhounkova Y., Tseng H., Schillinger D.  Nursing Research. 62(2) (pp 106-114), 2013. Date of Publication: March-April 2013.  [Article]  AN: 368531227 | Not relevant to the main subject/ wrong population or geographical location/ duplicate or repetition |
|  | The effectiveness of text messages support for diabetes self-management: protocol of the TEXT4DSM study in the democratic Republic of Congo, Cambodia and the Philippines.  van Olmen J., Ku G.M., van Pelt M., Kalobu J.C., Hen H., Darras C., Van Acker K., Villaraza B., Schellevis F., Kegels G.  BMC public health. 13 (pp 423), 2013. Date of Publication: 2013.  [Article]  AN: 603060700 | Not relevant to the main subject/ wrong population or geographical location/ duplicate or repetition |
|  | Mobile phone messaging for preventive health care.  Vodopivec-Jamsek V., de Jongh T., Gurol-Urganci I., Atun R., Car J.  Cochrane Database of Systematic Reviews. 2017(12) (no pagination), 2012. Article Number: CD007457. Date of Publication: 12 Dec 2012.  [Review]  AN: 619465506 | Not relevant to the main subject/ wrong population or geographical location/ duplicate or repetition |
|  | Mobile phone messaging for facilitating self-management of long-term illnesses.  de Jongh T., Gurol-Urganci I., Vodopivec-Jamsek V., Car J., Atun R.  Cochrane Database of Systematic Reviews. 2017(12) (no pagination), 2012. Article Number: CD007459. Date of Publication: 12 Dec 2012.  [Review]  AN: 619465510 | Not relevant to the main subject/ wrong population or geographical location/ duplicate or repetition |
|  | Diabetes Buddies: Peer Support Through a Mobile Phone Buddy System.  Rotheram-Borus M.J., Tomlinson M., Gwegwe M., Comulada W.S., Kaufman N., Keim M.  Diabetes Educator. 38(3) (pp 357-365), 2012. Date of Publication: May-June 2012.  [Article]  AN: 364832054 | Not relevant to the main subject/ wrong population or geographical location/ duplicate or repetition |
|  | Ocular telehealth screenings in an urban community.  Shahid K., Kolomeyer A.M., Nayak N.V., Salameh N., Pelaez G., Khouri A.S., Eck T.T., Szirth B.  Telemedicine journal and e-health : the official journal of the American Telemedicine Association. 18(2) (pp 95-100), 2012. Date of Publication: Mar 2012.  [Article]  AN: 365184538 | Not relevant to the main subject/ wrong population or geographical location/ duplicate or repetition |
|  | The selection and use of essential medicines.  Abdel-Aleem H., Bero L.A., Cheraghali A.M., Cranswick N., Fernandopulle R., Gray A., Hoppu K., Kearns G.L., Ofori-Adjei D., Wannmacher L., Zaidi A., Raymond A.S., Welbeck J., Nielsen H., Ahmed K.U., Porras A., Kertesz D., Annan E.A., Ondari C., Hill S., Ridge A., Renevier M.  World Health Organization - Technical Report Series. (965) (pp 1-268), 2012. Date of Publication: 2012.  [Conference Paper]  AN: 369181147 | Not relevant to the main subject/ wrong population or geographical location/ duplicate or repetition |
|  | Nurse-led disease management for hypertension control in a diverse urban community: A randomized trial.  Hebert P.L., Sisk J.E., Tuzzio L., Casabianca J.M., Pogue V.A., Wang J.J., Chen Y., Cowles C., McLaughlin M.A.  Journal of General Internal Medicine. 27(6) (pp 630-639), 2012. Date of Publication: June 2012.  [Article]  AN: 51752021 | Not relevant to the main subject/ wrong population or geographical location/ duplicate or repetition |
|  | Streptococcus uberis, an unusual cause of community acquired pneumonia.  Upadhyay S., Pesola G.R.  American Journal of Respiratory and Critical Care Medicine. Conference: American Thoracic Society International Conference, ATS 2012. San Francisco, CA United States. Conference Publication: (var.pagings). 185(MeetingAbstracts) (no pagination), 2012. Date of Publication: 2012.  [Conference Abstract]  AN: 71993286 | Not relevant to the main subject/ wrong population or geographical location/ duplicate or repetition |
|  | Knowledge of risk factors for heart disease among yoruba community of Nigeria.  Oladapo O., Salako L., Sadiq L., Shoyinka K., Falase A.  Circulation. Conference: World Congress of Cardiology Scientific Sessions 2012, WCC 2012. Dubai United Arab Emirates. Conference Publication: (var.pagings). 125(19) (pp e809), 2012. Date of Publication: 15 May 2012.  [Conference Abstract]  AN: 71051656 | Not relevant to the main subject/ wrong population or geographical location/ duplicate or repetition |
|  | Platelet reactivity and CD39 ectonucleotidase activities in cryptogenic stroke.  Marcus A.J., Drosopoulos J.H.F., Olson K.E., Anand R., Leifer D., Kizer J.R.  Blood. Conference: 54th Annual Meeting of the American Society of Hematology, ASH 2012. Atlanta, GA United States. Conference Publication: (var.pagings). 120(21) (no pagination), 2012. Date of Publication: 16 Nov 2012.  [Conference Abstract]  AN: 70964512 | Not relevant to the main subject/ wrong population or geographical location/ duplicate or repetition |
|  | Mestinon 60 mg in the treatment of diabetic cystopathy: Our experience about 24 cases. Mestinon 60 mg dans le traitement de la cystopathie diabetique: notre experience a propos de 24 cas <Mestinon 60 mg dans le traitement de la cystopathie diabetique: notre experience a propos de 24 cas.>  Ouanes W., Benzarti H., Mallat F., Hmida W., Salah N., Tlili G., Hidoussi A., Ben Sorba N., Jaidane M., Mosbah F., Maaref K., Bouaziz A., Zaoui A., Nejib R.  Annals of Physical and Rehabilitation Medicine. Conference: 27e Congres de Medecine Physique et de Readaptation. Toulouse France. Conference Publication: (var.pagings). 55(SUPPL.1) (pp e384+e386), 2012. Date of Publication: October 2012.  [Conference Abstract]  AN: 70941589 | Not relevant to the main subject/ wrong population or geographical location/ duplicate or repetition |
|  | Photo-electromagnetic field treatment of diabetes.  Ramdawon P.  Lasers in Medical Science. Conference: 26th International Congress Laser Medicine and IALMS Courses, Laser Florence 2012. Florence Italy. Conference Publication: (var.pagings). 27(6) (pp 1121-1122), 2012. Date of Publication: November 2012.  [Conference Abstract]  AN: 70915330 | Not relevant to the main subject/ wrong population or geographical location/ duplicate or repetition |
|  | Mestinon 60 mg in the treatment of diabetic cystopathy: Our experience about 27 cases.  Wiseem H., Ghassen T., Adnen H., Salah Nejib B., Adel S., Mehdi J., Sorba Nebil B., Faouzi M.  Journal of Endourology. Conference: 30th World Congress of Endourology and SWL, WCE 2012. Istanbul Turkey. Conference Publication: (var.pagings). 26(SUPPL. 1) (pp A396), 2012. Date of Publication: September 2012.  [Conference Abstract]  AN: 70876657 | Not relevant to the main subject/ wrong population or geographical location/ duplicate or repetition |
|  | Recruiting lower-income african american women into a diabetes medical nutritional therapy intervention.  Brooks M.A., Miller-Hughes S.  Diabetes. Conference: 72nd Scientific Sessions of the American Diabetes Association. Philadelphia, PA United States. Conference Publication: (var.pagings). 61(SUPPL. 1) (pp A585), 2012. Date of Publication: June 2012.  [Conference Abstract]  AN: 70798857 | Not relevant to the main subject/ wrong population or geographical location/ duplicate or repetition |
|  | Patient characteristics and participation in genetics studies: A type 2 diabetes cohort.  Amiri L., Thomas A., Cassidy-Bushrow A.E., Dakki H., Li J., Wells K., Lanfear D., Oliveria S.A., Phillips S., Ulcickas M.  Journal of Investigative Medicine. Conference: 2012 Combined Annual Meeting of the Central Society for Clinical Research and the Midwestern Section American Federation for Medical Research. Chicago, IL United States. Conference Publication: (var.pagings). 60(4) (pp 718), 2012. Date of Publication: April 2012.  [Conference Abstract]  AN: 70780618 | Not relevant to the main subject/ wrong population or geographical location/ duplicate or repetition |
|  | Val(8)GLP-1 rescues synaptic plasticity and reduces dense core plaques in APP/PS1 mice.  Gengler S., McClean P.L., McCurtin R., Gault V.A., Holscher C.  Neurobiology of Aging. 33(2) (pp 265-276), 2012. Date of Publication: February 2012.  [Article]  AN: 50854679 | Not relevant to the main subject/ wrong population or geographical location/ duplicate or repetition |
|  | Current strategic approaches in ethnomedicinal plants of Tinospora cordifolia and Gloriosa superba - A review.  Veeraiah S., Jaganmohan Reddy K.  International Journal of Pharma and Bio Sciences. 3(2) (pp 320-326), 2012. Date of Publication: April/June 2012.  [Review]  AN: 366069385 | Not relevant to the main subject/ wrong population or geographical location/ duplicate or repetition |
|  | Socio-demographic psychosocial and clinical characteristics of participants in e-healthystrides©: An interactive eHealth program to improve diabetes self-management skills.  Pemu P.E., Quarshie A.Q., Josiah-Willock R., Ojutalayo F.O., Alema-Mensah E., Ofili E.O.  Journal of Health Care for the Poor and Underserved. 22(4 SUPPL.) (pp 146-164), 2011. Date of Publication: 2011.  [Article]  AN: 364019058 | Not relevant to the main subject/ wrong population or geographical location/ duplicate or repetition |
|  | Natural antidiabetic compound for the therapeutic management of diabetes mellitus and its drug delivery system.  Emeje M., Boyi S., Obidike I., Isimi C., Kunle O., Ofoefule S.  Journal of Dietary Supplements. 8(3) (pp 266-279), 2011. Date of Publication: September 2011.  [Article]  AN: 362876729 | Not relevant to the main subject/ wrong population or geographical location/ duplicate or repetition |
|  | Knowledge, attitude and practice of ministry of health primary health care physicians in the management of type 2 diabetes mellitus: A crosssectional study in the Al Hasa District of Saudi Arabia, 2010.  Khan A.T., Lateef N.A.-A., Khamseen Ma.B., Al Aithan Ma., Khan S.A., Al Ibrahim I.  Nigerian Journal of Clinical Practice. 14(1) (pp 52-59), 2011. Date of Publication: January-March 2011.  [Article]  AN: 361658645 | Not relevant to the main subject/ wrong population or geographical location/ duplicate or repetition |
|  | Negative intercepts in the Heckel analysis of the crude extract of Vernonia galamensis: A major setback of the equation.  Allagh T., Autamashih M., Isah A.  Journal of Pharmaceutical Negative Results. 2(1) (pp 14-19), 2011. Date of Publication: January-June 2011.  [Article]  AN: 365693464 | Not relevant to the main subject/ wrong population or geographical location/ duplicate or repetition |
|  | Feasibility and usability of a text message-based program for diabetes self-management in an urban African-American population.  Dick J.J., Nundy S., Solomon M.C., Bishop K.N., Chin M.H., Peek M.E.  Journal of diabetes science and technology. 5(5) (pp 1246-1254), 2011. Date of Publication: Sep 2011.  [Article]  AN: 560048040 | Not relevant to the main subject/ wrong population or geographical location/ duplicate or repetition |
|  | Development of a mobile phone based ophthalmoscope for telemedicine.  Blanckenberg M., Worst C., Scheffer C.  Conference proceedings : ... Annual International Conference of the IEEE Engineering in Medicine and Biology Society. IEEE Engineering in Medicine and Biology Society. Conference. 2011 (pp 5236-5239), 2011. Date of Publication: 2011.  [Article]  AN: 365004581 | Not relevant to the main subject/ wrong population or geographical location/ duplicate or repetition |
|  | A patient-centric, provider-assisted diabetes telehealth self-management intervention for urban minorities.  Carter E.L., Nunlee-Bland G., Callender C.  Perspectives in health information management / AHIMA, American Health Information Management Association. 8 (pp 1b), 2011. Date of Publication: 2011.  [Article]  AN: 361917440 | Not relevant to the main subject/ wrong population or geographical location/ duplicate or repetition |
|  | Prevalence and severity of diabetic retinopathy in Northwest Cameroon as identified by teleophthalmology.  Jivraj I., Ng M., Rudnisky C.J., Dimla B., Tambe E., Nathoo N., Tennant M.T.  Telemedicine journal and e-health : the official journal of the American Telemedicine Association. 17(4) (pp 294-298), 2011. Date of Publication: May 2011.  [Article]  AN: 362652063 | Not relevant to the main subject/ wrong population or geographical location/ duplicate or repetition |
|  | Fourier transform infrared spectroscopy for in-process inspection, counterfeit detection and quality control of anti-diabetic drugs.  Farouk F., Moussa B.A., Azzazy H.M.E.-S.  Spectroscopy. 26(4-5) (pp 297-309), 2011. Date of Publication: 2011.  [Article]  AN: 364424536 | Not relevant to the main subject/ wrong population or geographical location/ duplicate or repetition |
|  | Adiponectin and all-cause mortality in a cohort of elderly people with type 2 diabetes.  Singer J.R., Palmas W., Shea S., Luchsinger J.A.  Journal of General Internal Medicine. Conference: 34th Annual Meeting of the Society of General Internal Medicine. Phoenix, AZ United States. Conference Publication: (var.pagings). 26(10) (pp 1219-1220), 2011. Date of Publication: October 2011.  [Conference Abstract]  AN: 71127943 | Not relevant to the main subject/ wrong population or geographical location/ duplicate or repetition |
|  | Patient centered care: Baseline demographics data from a stage I hypertension clinical trial.  Memon N., Kashem A., Gupta A., Gonzalez J., Alkhouli M., Hewitt V., Scheiring K., Bove A.A.  Circulation: Cardiovascular Quality and Outcomes. Conference: Quality of Care and Outcomes Research in Cardiovascular Disease and Stroke 2010 Scientific Sessions, QCOR 2010. Washington, DC United States. Conference Publication: (var.pagings). 4(6 MeetingAbstracts2010) (no pagination), 2011. Date of Publication: November 2011.  [Conference Abstract]  AN: 71256853 | Not relevant to the main subject/ wrong population or geographical location/ duplicate or repetition |
|  | Factors affecting the decision to treat cardiovascular risk in an urban underserved population with intermediate framingham risk.  Afari-Armah N., Kashem A., Memon N., Alkhouli M., Reed A., Rakita V., Hewitt V., Homko C., Santamore W.P., Bove A.A.  Circulation: Cardiovascular Quality and Outcomes. Conference: Quality of Care and Outcomes Research in Cardiovascular Disease and Stroke 2011 Scientific Sessions, QCOR 2011. Washington, DC United States. Conference Publication: (var.pagings). 4(6 MeetingAbstracts2011) (no pagination), 2011. Date of Publication: November 2011.  [Conference Abstract]  AN: 71256551 | Not relevant to the main subject/ wrong population or geographical location/ duplicate or repetition |
|  | Potential barriers to mammography screening in Hispanic/Latino women living in the Northeast United States.  Jones B.A., Doyle M., Torres A., Genao I., Kasl S.V., Soler-Vila H., Nunez Smith M., Claus E.B., Nappi S., Miranda A.  Cancer Epidemiology Biomarkers and Prevention. Conference: American Association for Cancer Research, AACR International Conference on the Science of Cancer Health Disparities 2011. Washington, DC United States. Conference Publication: (var.pagings). 20(10 Meeting Abstracts) (no pagination), 2011. Date of Publication: September 2011.  [Conference Abstract]  AN: 70707285 | Not relevant to the main subject/ wrong population or geographical location/ duplicate or repetition |
|  | Liraglutide, a novel GLP-1 analogue, prevents the impairment of learning and LTP and plaque formation in an APP/ PS-1 mouse model of Alzheimer's disease.  McClean P.L., Parthasarathy V., Gault V.A., Holscher C.  Irish Journal of Medical Science. Conference: 4th Annual Neuroscience Ireland Conference 2010. Dublin Ireland. Conference Publication: (var.pagings). 180(SUPPL. 2) (pp S24), 2011. Date of Publication: February 2011.  [Conference Abstract]  AN: 70701302 | Not relevant to the main subject/ wrong population or geographical location/ duplicate or repetition |
|  | Adiponectin and all-cause mortality in a cohort of elderly people with type 2 diabetes.  Rohman Singer J., Palmas W., Shea S., Alejandro Luchsinger J.  Journal of General Internal Medicine. Conference: 34th Annual Meeting of the Society of General Internal Medicine. Phoenix, AZ United States. Conference Publication: (var.pagings). 26(SUPPL. 1) (pp S5), 2011. Date of Publication: May 2011.  [Conference Abstract]  AN: 70653242 | Not relevant to the main subject/ wrong population or geographical location/ duplicate or repetition |
|  | The role of medication non-adherence in blood glucose control in the hispanic population in riverside service area.  Nimalasuriya A., Diestra M.D., Castellanos M., Truong L.K., Rajasingham A.R., Lara O.M., Merica K.M., Rajaratnam R.G., Mirza B.  Diabetes. Conference: 71st Scientific Sessions of the American Diabetes Association. San Diego, CA United States. Conference Publication: (var.pagings). 60(SUPPL. 1) (pp A321-A322), 2011. Date of Publication: July 2011.  [Conference Abstract]  AN: 70628934 | Not relevant to the main subject/ wrong population or geographical location/ duplicate or repetition |
|  | Hypertension management in urban underserved patients using an Internet communication system.  Kashem A., Keper M., Homko C.J., Santamore W.P., Hewitt V., Eubanks A., Raza F., Reed A., Alkhouli M., Bove A.A.  Journal of the American College of Cardiology. Conference: 60th Annual Scientific Session of the American College of Cardiology and i2 Summit: Innovation in Intervention, ACC.11. New Orleans, LA United States. Conference Publication: (var.pagings). 57(14 SUPPL. 1) (pp E1280), 2011. Date of Publication: 05 Apr 2011.  [Conference Abstract]  AN: 70400957 | Not relevant to the main subject/ wrong population or geographical location/ duplicate or repetition |
|  | Cellphone use to improve glucose control in a busy hospital practice.  Johnson-Loots L.  Diabetes Technology and Therapeutics. Conference: 4th International Conference on Advanced Technologies and Treatments for Diabetes, ATTD 2011. London United Kingdom. Conference Publication: (var.pagings). 13(2) (pp 234), 2011. Date of Publication: February 2011.  [Conference Abstract]  AN: 70350650 | Not relevant to the main subject/ wrong population or geographical location/ duplicate or repetition |
|  | Long-awaited dream of oral insulin: Where did we reach?.  Madhav M.  Asian Journal of Pharmaceutical and Clinical Research. 4(SUPPL. 2) (pp 16-21), 2011. Date of Publication: December 2011.  [Review]  AN: 363089947 | Not relevant to the main subject/ wrong population or geographical location/ duplicate or repetition |
|  | Glipizide Pharmacokinetics in Healthy and Diabetic Volunteers.  Atif M., Ahmad M., Qamar-uz-zaman M., Asif M., Sulaiman S.A.S., Shafie A.A., Masood I., Minhas U., Us-saqib N.  Tropical Journal of Pharmaceutical Research. 10(2) (pp 147-152), 2011. Date of Publication: April 2011.  [Article]  AN: 361758411 | Not relevant to the main subject/ wrong population or geographical location/ duplicate or repetition |
|  | Patient understanding of diabetes self-management: Participatory decision-making in diabetes care.  Quinn C.C., Royak-Schaler R., Lender D., Steinle N., Gadalla S., Zhan M.  Journal of Diabetes Science and Technology. 5(3) (pp 723-730), 2011. Date of Publication: May 2011.  [Article]  AN: 370248270 | Not relevant to the main subject/ wrong population or geographical location/ duplicate or repetition |
|  | Chronotherapeutic drug delivery systems - an approach to circadian rhythms diseases.  Sunil S.A., Srikanth M.V., Rao N.S., Uhumwangho M.U., Latha K., Murthy K.V.R.  Current Drug Delivery. 8(6) (pp 622-633), 2011. Date of Publication: November 2011.  [Review]  AN: 362750773 | Not relevant to the main subject/ wrong population or geographical location/ duplicate or repetition |
|  | Infection control in theatre.  Hold A.  Southern African Journal of Anaesthesia and Analgesia. 17(1) (pp 56-64), 2011. Date of Publication: January-February 2011.  [Conference Paper]  AN: 361472922 | Not relevant to the main subject/ wrong population or geographical location/ duplicate or repetition |
|  | Effects of garlic on blood glucose levels and HbA1c in patients with type 2 diabetes mellitus.  Ashraf R., Khan R.A., Ashraf I.  Journal of Medicinal Plants Research. 5(13) (pp 2922-2928), 2011. Date of Publication: 04 Jul 2011.  [Article]  AN: 362270400 | Not relevant to the main subject/ wrong population or geographical location/ duplicate or repetition |
|  | Effects of selected diluents and maize starch mucilage binder on the tablets formulation of the crude aqueous extract of Vernonia galamensis.  Autamashih M., Isah A.B., Allagh T.S., Ibrahim M.A.  International Journal of Pharmacy and Technology. 3(2) (pp 2746-2756), 2011. Date of Publication: April-June 2011.  [Article]  AN: 362110307 | Not relevant to the main subject/ wrong population or geographical location/ duplicate or repetition |
|  | Preliminary application of a new bolus insulin model for type 1 diabetes.  Pelzer R., Mathews E.H., Liebenberg L.  Diabetes Technology and Therapeutics. 13(5) (pp 527-535), 2011. Date of Publication: 01 May 2011.  [Article]  AN: 361690321 | Not relevant to the main subject/ wrong population or geographical location/ duplicate or repetition |
|  | Quality of care for patients with type 2 diabetes in general practice according to patients' ethnic background: a cross-sectional study from Oslo, Norway.  Tran A.T., Diep L.M., Cooper J.G., Claudi T., Straand J., Birkeland K., Ingskog W., Jenum A.K.  BMC health services research. 10 (pp 145), 2010. Date of Publication: 2010.  [Article]  AN: 359876874 | Not relevant to the main subject/ wrong population or geographical location/ duplicate or repetition |
|  | Mobile telemonitoring for achieving tighter targets of blood pressure control in patients with complicated diabetes: A pilot study.  Earle K.A., Istepanian R.S.H., Zitouni K., Sungoor A., Tang B.  Diabetes Technology and Therapeutics. 12(7) (pp 575-579), 2010. Date of Publication: 01 Jul 2010.  [Article]  AN: 359144902 | Not relevant to the main subject/ wrong population or geographical location/ duplicate or repetition |
|  | Rationale and design: Telephone-delivered behavioral skills interventions for Blacks with type 2 diabetes.  Egede L.E., Strom J.L., Durkalski V.L., Mauldin P.D., Moran W.P.  Trials. 11 (no pagination), 2010. Article Number: 35. Date of Publication: 29 Mar 2010.  [Article]  AN: 358915311 | Not relevant to the main subject/ wrong population or geographical location/ duplicate or repetition |
|  | Interaction of sleep quality and psychosocial stress on obesity in African Americans: the Cardiovascular Health Epidemiology Study (CHES).  Bidulescu A., Din-Dzietham R., Coverson D.L., Chen Z., Meng Y.X., Buxbaum S.G., Gibbons G.H., Welch V.L.  BMC public health. 10 (pp 581), 2010. Date of Publication: 2010.  [Article]  AN: 360251118 | Not relevant to the main subject/ wrong population or geographical location/ duplicate or repetition |
|  | Telemedicine for recently discharged older patients.  Cardozo L., Steinberg J.  Telemedicine journal and e-health : the official journal of the American Telemedicine Association. 16(1) (pp 49-55), 2010. Date of Publication: 2010 Jan-Feb.  [Article]  AN: 358677149 | Not relevant to the main subject/ wrong population or geographical location/ duplicate or repetition |
|  | Vitamin D deficiency in ethnic minorities in Denmark.  Nielsen D.S., Svabo A., Sodemann M.  Bone. Conference: 37th European Symposium on Calcified Tissues, ECTS 2010. Glasgow United Kingdom. Conference Publication: (var.pagings). 47(SUPPL. 1) (pp S232-S233), 2010. Date of Publication: June 2010.  [Conference Abstract]  AN: 71732864 | Not relevant to the main subject/ wrong population or geographical location/ duplicate or repetition |
|  | A survey of in-patient diabetes care in a NHS foundation trust.  Dyer P.H., Holmes C.A.  Diabetes. Conference: 70th Scientific Sessions of the American Diabetes Association. Orlando, FL United States. Conference Publication: (var.pagings). (no pagination), 2010. Date of Publication: 2010.  [Conference Abstract]  AN: 71602441 | Not relevant to the main subject/ wrong population or geographical location/ duplicate or repetition |
|  | A comprehensive collaborative enhanced diabetes care program in the rural resource constrained setting of eldoret, kenya.  Ouma M.N., Pastakia S.D.  Diabetes. Conference: 70th Scientific Sessions of the American Diabetes Association. Orlando, FL United States. Conference Publication: (var.pagings). (no pagination), 2010. Date of Publication: 2010.  [Conference Abstract]  AN: 71600657 | Not relevant to the main subject/ wrong population or geographical location/ duplicate or repetition |
|  | Adherence to diabetes self care for white, African American and hispanic American telemedicine participants: Ideatel project.  Trief P.M., Izquierdo R., Eimicke J.P., Teresi J.A., Goland R., Palmas W., Shea S., Weinstock R.S.  Diabetes. Conference: 70th Scientific Sessions of the American Diabetes Association. Orlando, FL United States. Conference Publication: (var.pagings). (no pagination), 2010. Date of Publication: 2010.  [Conference Abstract]  AN: 71600483 | Not relevant to the main subject/ wrong population or geographical location/ duplicate or repetition |
|  | Breast cancer in men: Beyond the stigma.  Win Y., Ma A., Matti B., Quinlan E.  Journal of the American Geriatrics Society. Conference: 2010 Annual Scientific Meeting of the American Geriatrics Society. Orlando, FL United States. Conference Publication: (var.pagings). 58(SUPPL. 1) (pp S142), 2010. Date of Publication: April 2010.  [Conference Abstract]  AN: 70990638 | Not relevant to the main subject/ wrong population or geographical location/ duplicate or repetition |
|  | Omega-3 fatty acids reduce the number of crisis and steady state hemolysis in sickle cell disease.  Okpala I., Ibegbulam O., Duru A., Ocheni S., Emodi I., Ikefuna A., Kangiwa U., Asinobi I., Madu A., Okoye A., Nwagha T., Oguonu U., Uamai I., Agwu O., Nonyelum C., Anike U., Agu K., Anigbo C., Chukwura A., Ugwu O., Herrada S.  Blood. Conference: 52nd Annual Meeting of the American Society of Hematology, ASH 2010. Orlando, FL United States. Conference Publication: (var.pagings). 116(21) (no pagination), 2010. Date of Publication: 19 Nov 2010.  [Conference Abstract]  AN: 70776049 | Not relevant to the main subject/ wrong population or geographical location/ duplicate or repetition |
|  | CSHP Summer Educational Sessions (SES) 2010: Poster Abstracts.  Anonymous  Canadian Journal of Hospital Pharmacy. Conference: CSHP Summer Educational Sessions, SES 2010. Halifax, NS Canada. Conference Publication: (var.pagings). 63(4) (no pagination), 2010. Date of Publication: August 2010.  [Conference Review]  AN: 70638440 | Not relevant to the main subject/ wrong population or geographical location/ duplicate or repetition |
|  | The challenges of children with diabetes west Nile region Arua-Uganda.  Joyo A.N.  Pediatric Diabetes. Conference: 36th Annual Meeting of the International Society for Pediatric and Adolescent Diabetes, ISPAD. Buenos Aires Argentina. Conference Publication: (var.pagings). 11(SUPPL. 14) (pp 95), 2010. Date of Publication: October 2010.  [Conference Abstract]  AN: 70325638 | Not relevant to the main subject/ wrong population or geographical location/ duplicate or repetition |
|  | Society of General Internal Medicine - 33rd Annual Meeting.  Anonymous  Journal of General Internal Medicine. Conference: 33rd Annual Meeting of the Society of General Internal Medicine. Minneapolis, MN United States. Conference Publication: (var.pagings). 25(SUPPL. 3) (no pagination), 2010. Date of Publication: June 2010.  [Conference Review]  AN: 70315607 | Not relevant to the main subject/ wrong population or geographical location/ duplicate or repetition |
|  | Surgical treatment of aneurysms and false aneurysms.  Rawa M.  Journal of Vascular Access. Conference: Angio Access for Hemodialysis. Tours France. Conference Publication: (var.pagings). 11(SUPPL. 3) (pp S30-S31), 2010. Date of Publication: April-June 2010.  [Conference Abstract]  AN: 70296276 | Not relevant to the main subject/ wrong population or geographical location/ duplicate or repetition |
|  | Gender difference in the burden of cardiovascular risk factors among rural and urban adult cameroonian populations. Results from the vitaraa study.  Lemogoum D., Bayauli P., Toto Moukouo J., Van De Borne P., M'Buyamba-Kayamba R., Leeman M., Degaute J.P., M'Buyamba-Kabangu J.R.  Journal of Hypertension. Conference: 20th European Meeting on Hypertension of the European Society of Hypertension, ESH. Oslo Norway. Sponsor: Boehringer Ingelheim, Daiichi-Sankyo, NOVARTIS, SERVIER, RECORDATI . Conference Publication: (var.pagings). 28(SUPPL. A) (pp e134-e135), 2010. Date of Publication: June 2010.  [Conference Abstract]  AN: 70214643 | Not relevant to the main subject/ wrong population or geographical location/ duplicate or repetition |
|  | The Use of Quality Improvement and Health Information Technology Approaches to Improve Diabetes Outcomes in African American and Hispanic Patients.  Baig A.A., Wilkes A.E., Davis A.M., Peek M.E., Huang E.S., Bell D.S., Chin M.H.  Medical Care Research and Review. 67(5_suppl) (pp 163S-197S), 2010. Date of Publication: 01 Oct 2010.  [Review]  AN: 613125790 | Not relevant to the main subject/ wrong population or geographical location/ duplicate or repetition |
|  | Biopharmaceutical evaluation of formulated metformin/rosiglitazone tablets.  Ibrahim H.K., Attia A.M., Ghorab M.M.  Drug discoveries & therapeutics. 4(2) (pp 100-108), 2010. Date of Publication: 01 Apr 2010.  [Article]  AN: 611747788 | Not relevant to the main subject/ wrong population or geographical location/ duplicate or repetition |
|  | Review paper: The use of quality improvement and health information technology approaches to improve diabetes outcomes in African American and hispanic patients.  Baig A.A., Wilkes A.E., Davis A.M., Peek M.E., Huang E.S., Bell D.S., Chin M.H.  Medical Care Research and Review. 67(5) (pp 163S-197S), 2010. Date of Publication: October 2010.  [Review]  AN: 359571671 | Not relevant to the main subject/ wrong population or geographical location/ duplicate or repetition |
|  | Effect of interventions to improve health care services for ethnic minority populations.  Forsetlund L., Eike M.C., Vist G.E.  Norsk Epidemiologi. 20(1) (pp 41-52), 2010. Date of Publication: 2010.  [Article]  AN: 361468954 | Not relevant to the main subject/ wrong population or geographical location/ duplicate or repetition |
|  | Mobile phone ownership among Nigerians with diabetes.  Okoro E.O., Sholagberu H.O., Kolo P.M.  African Health Sciences. 10(2) (pp 183-186), 2010. Date of Publication: 2010.  [Article]  AN: 359887517 | Not relevant to the main subject/ wrong population or geographical location/ duplicate or repetition |
|  | Surveillance of Chronic Kidney Disease Around the World: Tracking and Reigning in a Global Problem.  Saran R., Hedgeman E., Huseini M., Stack A., Shahinian V.  Advances in Chronic Kidney Disease. 17(3) (pp 271-281), 2010. Date of Publication: May 2010.  [Review]  AN: 358720611 | Not relevant to the main subject/ wrong population or geographical location/ duplicate or repetition |
|  | Observance to antidiabetic treatment in a developing country: The case in Burkina Faso (sub-Saharan Africa). Observance du traitement antidiabetique dans un pays en developpement: Le cas du Burkina Faso (Afrique subsaharienne) <Observance du traitement antidiabetique dans un pays en developpement: Le cas du Burkina Faso (Afrique subsaharienne).>  Tieno H., Bouda M., Ouedraogo D.-D., Traore R., Ouedraogo C., Drabo Y.J.  Medecine des Maladies Metaboliques. 4(2) (pp 207-211), 2010. Date of Publication: March 2010.  [Article]  AN: 358586885 | Not relevant to the main subject/ wrong population or geographical location/ duplicate or repetition |
|  | Physical activity patterns and eating habits of adolescents living in major Arab cities. The Arab Teens Lifestyle Study.  Al-Hazzaa H.M., Musaiger A.O., Abahussain N.A., Al-Sobayel H.I., Alsulaiman N.A., Tayyem R.F., Al-Haifi A.R., Al-Mofty P.A., Ahmad H.S., Desouki T.E.  Saudi Medical Journal. 31(2) (pp 210-211), 2010. Date of Publication: February 2010.  [Article]  AN: 358465470  Embase Accession Number  2010174187  PMID  20174744 [http://www.ncbi.nlm.nih.gov/pubmed/?term=20174744]  Status | Not relevant to the main subject/ wrong population or geographical location/ duplicate or repetition |
|  | Managing the space between visits: A randomized trial of disease management for diabetes in a community health center.  Anderson D.R., Christison-Lagay J., Villagra V., Liu H., Dziura J.  Journal of General Internal Medicine. 25(10) (pp 1116-1122), 2010. Date of Publication: October 2010.  [Article]  AN: 50958078 | Not relevant to the main subject/ wrong population or geographical location/ duplicate or repetition |
|  | The diabetes TeleCare (DTC) Study: 24-month follow-up data on patients living in rural medically underserved areas.  Davis R., Hitch A., Salaam M., Nichols M., Moran R., Mayer-Davis E.J.  Diabetes. Conference: 69th Annual Meeting of the American Diabetes Association. New Orleans, LA United States. Conference Publication: (var.pagings). 58(SUPPL. 1A) (no pagination), 2009. Date of Publication: 2009.  [Conference Abstract]  AN: 70136810 | Not relevant to the main subject/ wrong population or geographical location/ duplicate or repetition |
|  | Mobile diabetes RCT: Testing personalized patient communication and provider recommendations for blood glucose control.  Quinn C.C., Gruber-Baldini A., Peeples M., Clough S.S., Shardell M.  Diabetes. Conference: 69th Annual Meeting of the American Diabetes Association. New Orleans, LA United States. Conference Publication: (var.pagings). 58(SUPPL. 1A) (no pagination), 2009. Date of Publication: 2009.  [Conference Abstract]  AN: 70135730 | Not relevant to the main subject/ wrong population or geographical location/ duplicate or repetition |
|  | Given little attention, the views of HIV positive patients on fixed drug combinations - Taso Uganda experience.  Birungi J., Luzze C., Etukoit B.M., Mwesigwa R.  American Journal of Hypertension. Conference: 2nd International Conference on Fixed Combination in the Treatment of Hypertension, Dyslipidemia and Diabetes Mellitus: An Ideal Approach for Improving Compliance and Combating Cardiovascular Disease. Valencia Spain. Conference Publication: (var.pagings). 22(SUPPL. 1) (pp 5), 2009. Date of Publication: November 2009.  [Conference Abstract]  AN: 70081470 | Not relevant to the main subject/ wrong population or geographical location/ duplicate or repetition |
|  | Neuroimaging in Behcet's disease.  Kessaci F., Berrah A., Hakem D., Sellah M., Mansouri B.  European Journal of Neurology. Conference: 13th Congress of the EFNS. Florence Italy. Conference Publication: (var.pagings). 16(S3) (pp 503), 2009. Date of Publication: 2009.  [Conference Abstract]  AN: 70063511 | Not relevant to the main subject/ wrong population or geographical location/ duplicate or repetition |
|  | Rural residence and higher level of physical activity lessen the probability of hypertension in the Africans. Results fromthe Vitaraa study.  Lemogoum D., Van De Borne P.H., M'buyamba J.R., Pik J.J., Leeman M., Toto Moukouo J., Degaute J.P.  European Heart Journal. Conference: European Society of Cardiology, ESC Congress 2009. Barcelona Spain. Conference Publication: (var.pagings). 30(SUPPL. 1) (pp 160), 2009. Date of Publication: September 2009.  [Conference Abstract]  AN: 70353734 | Not relevant to the main subject/ wrong population or geographical location/ duplicate or repetition |
|  | The Egyptian National Breast Screening Program: Priorities, challenges, and results of the pilot phase.  Salem D.S., Kamal R., Said N.H., Adel I., Talaat S., Adel L., Abdel Razek N., Helal M., Selim A.  Journal of Clinical Oncology. Conference: 2009 Annual Meeting of the American Society of Clinical Oncology, ASCO. Orlando, FL United States. Conference Publication: (var.pagings). 27(15 SUPPL. 1) (pp 1523), 2009. Date of Publication: 20 May 2009.  [Conference Abstract]  AN: 70240611 | Not relevant to the main subject/ wrong population or geographical location/ duplicate or repetition |
|  | Genetic screening of C2399A SNP associated with the XPNPEP2 gene in South African hypertensive, ACE inhibitor induced angioedema in patients and controls.  Stark J., Moholisa R., Sturrock E., Rayner B., Owen E.  British Journal of Clinical Pharmacology. Conference: Proceedings of the British Pharmacological Society Clinical Pharmacology Section. Brighton . Conference Publication: (var.paging). 68(2) (pp 291), 2009. Date of Publication: August 2009.  [Conference Abstract]  AN: 70004856 | Not relevant to the main subject/ wrong population or geographical location/ duplicate or repetition |
|  | Metformin-induced acute painful myopathy.  Kastalli S., El Aidli S., Lakhoua G., Zaiem A., Cherif F., Daghfous R., Lakhal M.  Drug Safety. Conference: 9th ISoP Annual Meeting a~From Pharmacovigilance to Risk ManagementaTM. Reims France. Conference Publication: (var.pagings). 32(10) (pp 957), 2009. Date of Publication: 2009.  [Conference Abstract]  AN: 70004565 | Not relevant to the main subject/ wrong population or geographical location/ duplicate or repetition |
|  | Photosensitivity induced by metformin: A report of 3 cases. Photosensibilite induite par la metformine: A propos de 3 cas <Photosensibilite induite par la metformine: A propos de 3 cas.>  Kastalli S., El Aidli S., Chaabane A., Amrani R., Daghfous R., Belkahia C.  Tunisie Medicale. 87(10) (pp 703-705), 2009. Date of Publication: October 2009.  [Article]  AN: 358385357 | Not relevant to the main subject/ wrong population or geographical location/ duplicate or repetition |
|  | A collaborative approach to the recruitment and retention of minority patients with diabetes in rural community health centers.  Davis R.M., Hitch A.D., Nichols M., Rizvi A., Salaam M., Mayer-Davis E.J.  Contemporary Clinical Trials. 30(1) (pp 63-70), 2009. Date of Publication: January 2009.  [Article]  AN: 50306183 | Not relevant to the main subject/ wrong population or geographical location/ duplicate or repetition |
|  | Outcomes of an intervention to reduce uncertainty among African American women with diabetes.  Amoako E., Skelly A.H., Rossen E.K.  Western Journal of Nursing Research. 30(8) (pp 928-942), 2008. Date of Publication: December 2008.  [Article]  AN: 352620589 | Not relevant to the main subject/ wrong population or geographical location/ duplicate or repetition |
|  | Care coordination and telemedicine improves glycaemic control in ethnically diverse veterans with diabetes.  Dang S., Ma F., Nedd N., Florez H., Aguilar E., Roos B.A.  Journal of telemedicine and telecare. 13(5) (pp 263-267), 2007. Date of Publication: 2007.  [Article]  AN: 350342739 | Not relevant to the main subject/ wrong population or geographical location/ duplicate or repetition |
|  | A community-based telehealth programme for elderly low-income African Americans.  Buckley K., Tran B., Agazio J., Wuertz E.  Journal on Information Technology in Healthcare. 6(6) (pp 400-412), 2008. Date of Publication: 2008.  [Article]  AN: 355479141 | Not relevant to the main subject/ wrong population or geographical location/ duplicate or repetition |
|  | Efficacy and safety of oral paliperidone extended-release tablets in the treatment of acute schizophrenia: Pooled data from three 52-week open-label studies.  Emsley R., Berwaerts J., Eerdekens M., Kramer M., Lane R., Lim P., Hough D., Palumbo J.  International Clinical Psychopharmacology. 23(6) (pp 343-356), 2008. Date of Publication: November 2008.  [Article]  AN: 352653645 | Not relevant to the main subject/ wrong population or geographical location/ duplicate or repetition |
|  | Relationship between level of circulating modified LDL and the extent of coronary artery disease in type 2 diabetic patients.  El-Bassiouni E.A., Helmy M.H., El-Zoghby S.M., El-Nabi Kamel M.A., Hosny R.M.  British Journal of Biomedical Science. 64(3) (pp 109-116), 2007. Date of Publication: 2007.  [Article]  AN: 47477571 | Not relevant to the main subject/ wrong population or geographical location/ duplicate or repetition |
|  | Screening for diabetic retinopathy in primary care with a mobile fundal camera - Evaluation of a South African pilot project.  Mash B., Powell D., du Plessis F., van Vuuren U., Michalowska M., Levitt N.  South African Medical Journal. 97(12 I) (pp 1284-1288), 2007. Date of Publication: December 2007.  [Article]  AN: 351148769 | Not relevant to the main subject/ wrong population or geographical location/ duplicate or repetition |
|  | A 24-week, multicenter, randomized, double-blind, placebo-controlled, parallel-group study of the efficacy and tolerability of combination therapy with rosiglitazone and sulfonylurea in african american and hispanic american patients with type 2 diabetes inadequately controlled with sulfonylurea monotherapy.  Davidson J.A., McMorn S.O., Waterhouse B.R., Cobitz A.R.  Clinical Therapeutics. 29(9) (pp 1900-1914), 2007. Date of Publication: September 2007.  [Article]  AN: 350214459 | Not relevant to the main subject/ wrong population or geographical location/ duplicate or repetition |
|  | Health beliefs of African-Caribbean people with type 2 diabetes: A qualitative study.  Brown K., Avis M., Hubbard M.  British Journal of General Practice. 57(539) (pp 461-469), 2007. Date of Publication: June 2007.  [Article]  AN: 46894985 | Not relevant to the main subject/ wrong population or geographical location/ duplicate or repetition |
|  | The real-life safety and efficacy of vardenafil: An international post-marketing surveillance study of 2824 patients from the Middle East.  Kamel A., Khaouli R., Sabha M., Al Mitwally K., Fouad W., Landen H.  Clinical Drug Investigation. 27(5) (pp 339-346), 2007. Date of Publication: 2007.  [Article]  AN: 46789887 | Not relevant to the main subject/ wrong population or geographical location/ duplicate or repetition |
|  | A controlled comparison of the effect of a high fiber diet on the glycaemic and lipid profile of Nigerian clinic patients with type 2 diabetes.  Ikem R.T., Kolawole B.A., Ojofeitimi E.O., Salawu A., Ajose O.A., Abiose S., Odewale F.  Pakistan Journal of Nutrition. 6(2) (pp 111-116), 2007. Date of Publication: 2007.  [Article]  AN: 46359281 | Not relevant to the main subject/ wrong population or geographical location/ duplicate or repetition |
|  | Postanalytical external quality assessment of blood glucose and hemoglobin A1c: an international survey.  Skeie S., Perich C., Ricos C., Araczki A., Horvath A.R., Oosterhuis W.P., Bubner T., Nordin G., Delport R., Thue G., Sandberg S.  Clinical chemistry. 51(7) (pp 1145-1153), 2005. Date of Publication: Jul 2005.  [Article]  AN: 41106925 | Not relevant to the main subject/ wrong population or geographical location/ duplicate or repetition |
|  | The South African stroke risk in general practice study.  Connor M., Rheeder P., Bryer A., Meredith M., Beukes M., Dubb A., Fritz V.  South African Medical Journal. 95(5) (pp 334-339), 2005. Date of Publication: May 2005.  [Article]  AN: 40767035 | Not relevant to the main subject/ wrong population or geographical location/ duplicate or repetition |
|  | Postanalytical external quality assessment of blood glucose and hemoglobin A1c: An international survey.  Skeie S., Perich C., Ricos C., Araczki A., Horvath A.R., Oosterhuis W.P., Bubner T., Nordin G., Delport R., Thue G., Sandberg S.  Clinical Chemistry. 51(7) (pp 1145-1153), 2005. Date of Publication: 2005.  [Review]  AN: 43079391 | Not relevant to the main subject/ wrong population or geographical location/ duplicate or repetition |
|  | Voltammetric study of glibenclamide at carbon paste and Sephadex-modified carbon paste electrodes.  Radi A.  Analytical and bioanalytical chemistry. 378(3) (pp 822-826), 2004. Date of Publication: Feb 2004.  [Article]  AN: 38873191 | Not relevant to the main subject/ wrong population or geographical location/ duplicate or repetition |
|  | Use of mapping technology in health intervention research.  Gesler W.M., Hayes M., Arcury T.A., Skelly A.H., Nash S., Soward A.C.M.  Nursing Outlook. 52(3) (pp 142-146), 2004. Date of Publication: June 2004.  [Article]  AN: 38759625 | Not relevant to the main subject/ wrong population or geographical location/ duplicate or repetition |
|  | Cost-Effectiveness Analysis of Telemedicine to Evaluate Diabetic Retinopathy in a Prison Population.  Aoki N., Dunn K., Fukui T., Beck J.R., Schull W.J., Li H.K.  Diabetes Care. 27(5) (pp 1095-1101), 2004. Date of Publication: May 2004.  [Article]  AN: 38579772 | Not relevant to the main subject/ wrong population or geographical location/ duplicate or repetition |
|  | Treatment of diabetic foot lesions in hospital: Results of 2 successive five-year periods, 1918-1993 and 1994-1998.  Benotmane A., Faraoun K., Mohammedi F., Amani M.E., Benkhelifa T.  Diabetes and Metabolism. 30(3 I) (pp 245-250), 2004. Date of Publication: June 2004.  [Article]  AN: 38917454 | Not relevant to the main subject/ wrong population or geographical location/ duplicate or repetition |
|  | Formal clinical primary health care training. Does it make a difference?.  Louwagie G.M., Bachmann M.O., Reid M.  Curationis. 25(4) (pp 32-37), 2002. Date of Publication: Nov 2002.  [Article]  AN: 137571658 | Not relevant to the main subject/ wrong population or geographical location/ duplicate or repetition |
|  | Excess type 2 diabetes in African-American women and men aged 40-74 and socioeconomic status: Evidence from the third national health and nutrition examination survey.  Robbins J.M., Vaccarino V., Zhang H., Kasl S.V.  Journal of Epidemiology and Community Health. 54(11) (pp 839-845), 2000. Date of Publication: 2000.  [Article]  AN: 30799148 | Not relevant to the main subject/ wrong population or geographical location/ duplicate or repetition |
|  | Desloratadine Sepracor.  Norman P.  Current Opinion in Anti-inflammatory and Immunomodulatory Investigational Drugs. 2(2) (pp 117-126), 2000. Date of Publication: 2000.  [Review]  AN: 30251238 | Not relevant to the main subject/ wrong population or geographical location/ duplicate or repetition |
|  | Effects of a traditional lifestyle on the cardiovascular risk profile: The Amondava population of the Brazilian Amazon. Comparison with matched African, Italian and Polish populations.  Pavan L., Casiglia E., Carvalho Braga L.M., Winnicki M., Puato M., Pauletto P., Pessina A.C.  Journal of Hypertension. 17(6) (pp 749-756), 1999. Date of Publication: 1999.  [Article]  AN: 29322160 | Not relevant to the main subject/ wrong population or geographical location/ duplicate or repetition |
|  | Noncommunicable disease management in resource-poor settings: A primary care model from rural South Africa.  Coleman R., Gill G., Wilkinson D.  Bulletin of the World Health Organization. 76(6) (pp 633-640), 1998. Date of Publication: 1998.  [Article]  AN: 29143877 | Not relevant to the main subject/ wrong population or geographical location/ duplicate or repetition |
|  | Induction of labour with single insertion of vaginal tablet of prostagladin E2 (PGE2), amniotomy, and oxytocin infusion.  Odum C.U., Isika A.N., Lambo A.O.  West African journal of medicine. 12(3) (pp 153-157), 1993. Date of Publication: 1993 Jul-Sep.  [Article]  AN: 24863891 | Not relevant to the main subject/ wrong population or geographical location/ duplicate or repetition |
|  | Circulating acute phase reactive proteins as indicators of infection in poorly controlled diabetes mellitus.  Van Eeden S.F., Strachan A.F., Hough S.F.  Diabetes Research and Clinical Practice. 5(2) (pp 99-105), 1988. Date of Publication: 1988.  [Article]  AN: 18164795 | Not relevant to the main subject/ wrong population or geographical location/ duplicate or repetition |
|  | Pregnancy in established non-insulin-dependent diabetics. A five-and-a-half year study at Groote Schuur Hospital.  Coetzee E.J., Jackson W.P.U.  South African Medical Journal. 58(20) (pp 795-802), 1980. Date of Publication: 1980.  [Article]  AN: 11252891 | Not relevant to the main subject/ wrong population or geographical location/ duplicate or repetition |
|  | Diabetes in Ghana; a 10 year study.  Owusu S.K.  Ghana Medical Journal. 15(2) (pp 93-96), 1976. Date of Publication: 1976.  [Article]  AN: 8082439 | Not relevant to the main subject/ wrong population or geographical location/ duplicate or repetition |
|  | A patient-centric, provider-assisted diabetes telehealth self-management intervention for urban minorities  Carter EL, Nunlee-Bland G, Callender C  Perspectives in health information management. Vol.8, pp.1b, 2011.  [Journal Article. Randomized Controlled Trial. Research Support, N.I.H., Extramural]    Link to the Ovid Full Text or citation:  [Click here for full text options](https://ovidsp.ovid.com/ovidweb.cgi?T=JS&CSC=Y&NEWS=N&PAGE=fulltext&D=cctr&AN=CN-00787714)  Link to the External Link Resolver:  [LibKey Link](https://libkey.io/libraries/2401/pdfexpress/openurl?genre=article&aulast=&issn=1559-4122&title=Perspectives+in+health+information+management&atitle=A+patient-centric%2C+provider-assisted+diabetes+telehealth+self-management+intervention+for+urban+minorities&volume=8&issue=&spage=1b&epage=b&date=2011&doi=&pmid=21307985&sid=OVID:cctrdb) | Not relevant to the main subject/ wrong population or geographical location/ duplicate or repetition |
|  | Managing the space between visits: a randomized trial of disease management for diabetes in a community health center  Anderson DR, Christison-Lagay J, Villagra V, Liu H, Dziura J  Journal of general internal medicine. 25(10):1116-1122, 2010.  [Comparative Study. Journal Article. Randomized Controlled Trial. Research Support, Non-U.S. Gov't] | Not relevant to the main subject/ wrong population or geographical location/ duplicate or repetition |
|  | The effectiveness of text messages support for diabetes self-management: protocol of the TEXT4DSM study in the democratic Republic of Congo, Cambodia and the Philippines  van Olmen J, Ku GM, van Pelt M, Kalobu JC, Hen H, Darras C, Van Acker K, Villaraza B, Schellevis F, Kegels G  BMC public health. Vol.13, pp.423, 2013.  [Comparative Study. Journal Article. Randomized Controlled Trial. Research Support, Non-U.S. Gov't] | Not relevant to the main subject/ wrong population or geographical location/ duplicate or repetition |
|  | Rationale, design, and methodology for the optimizing outcomes in women with gestational diabetes mellitus and their infants study  Berry DC, Neal M, Hall EG, Schwartz TA, Verbiest S, Bonuck K, Goodnight W, Brody S, Dorman KF, Menard MK, Stuebe AM  BMC pregnancy and childbirth. Vol.13, pp.184, 2013.  [Journal Article. Randomized Controlled Trial. Research Support, N.I.H., Extramural] | Not relevant to the main subject/ wrong population or geographical location/ duplicate or repetition |
|  | Mobile-Enhanced Peer Support for African Americans with Type 2 Diabetes: a Randomized Controlled Trial  Presley C, Agne A, Shelton T, Oster R, Cherrington A  Journal of general internal medicine. 35(10):2889-2896, 2020.  [Journal Article. Randomized Controlled Trial. Research Support, N.I.H., Extramural. Research Support, Non-U.S. Gov't. Research Support, U.S. Gov't, P.H.S] | Not relevant to the main subject/ wrong population or geographical location/ duplicate or repetition |
|  | Racial Differences in the Effectiveness of a Multifactorial Telehealth Intervention to Slow Diabetic Kidney Disease  Kobe EA, Diamantidis CJ, Bosworth HB, Davenport CA, Oakes M, Alexopoulos AS, Pendergast J, Patel UD, Crowley MJ  Medical care. 58(11):968-973, 2020.  [Journal Article. Randomized Controlled Trial. Research Support, N.I.H., Extramural. Research Support, U.S. Gov't, Non-P.H.S] | Not relevant to the main subject/ wrong population or geographical location/ duplicate or repetition |
|  | Digital messaging to support control for type 2 diabetes (StAR2D): a multicentre randomised controlled trial  Farmer A, Bobrow K, Leon N, Williams N, Phiri E, Namadingo H, Cooper S, Prince J, Crampin A, Besada D, Daviaud E, Yu LM, N'goma J, Springer D, Pauly B, Tarassenko L, Norris S, Nyirenda M, Levitt N  BMC public health. 21(1):1907, 2021.  [Journal Article. Multicenter Study. Randomized Controlled Trial. Research Support, Non-U.S. Gov't] | Not relevant to the main subject/ wrong population or geographical location/ duplicate or repetition |
|  | Manchester Intermittent and Daily diet Diabetes App Study  ISRCTN15394285  <https://trialsearch.who.int/Trial2.aspx?TrialID=ISRCTN15394285>. 2018. | Not relevant to the main subject/ wrong population or geographical location/ duplicate or repetition |
|  | Efficacy of mobile phone SMS in promoting adherence to anti-diabetic therapy and glycaemic control among diabetic patients in South Africa  PACTR201810599931422  <https://trialsearch.who.int/Trial2.aspx?TrialID=PACTR201810599931422>. 2017. | Not relevant to the main subject/ wrong population or geographical location/ duplicate or repetition |
|  | Diabetes nurse self-management phone call trial in Ghana  PACTR201907488398987  <https://trialsearch.who.int/Trial2.aspx?TrialID=PACTR201907488398987>. 2019. | Not relevant to the main subject/ wrong population or geographical location/ duplicate or repetition |
|  | A research study comparing a new medicine oral semaglutide to sitagliptin in people with type 2 diabetes (PIONEER 12)  EUCTR2018-002589-38-CZ  <https://trialsearch.who.int/Trial2.aspx?TrialID=EUCTR2018-002589-38-CZ>. 2019. | Not relevant to the main subject/ wrong population or geographical location/ duplicate or repetition |
|  | A research study comparing a new medicine oral semaglutide to placebo in people with type 2 diabetes  EUCTR2018-002590-22-HU  <https://trialsearch.who.int/Trial2.aspx?TrialID=EUCTR2018-002590-22-HU>. 2019. | Not relevant to the main subject/ wrong population or geographical location/ duplicate or repetition |
|  | The Management of Diabetes in Everyday Life (MODEL) program: development of a tailored text message intervention to improve diabetes self-care activities among underserved African-American adults  Gatwood J, Shuvo S, Ross A, Riordan C, Smith P, Gutierrez ML, Coday M, Bailey J  Translational behavioral medicine. 10(1):204-212, 2020.  [Journal Article. Randomized Controlled Trial. Research Support, Non-U.S. Gov't] | Not relevant to the main subject/ wrong population or geographical location/ duplicate or repetition |
|  | The management of diabetes in everyday life study: design and methods for a pragmatic randomized controlled trial comparing the effectiveness of text messaging versus health coaching  Bailey JE, Surbhi S, Gatwood J, Butterworth S, Coday M, Shuvo SA, Dashputre AA, Brooks IM, Binkley BL, Riordan CJ, Steinberg HO, Gutierrez ML, Haley LE, Leak CL, Tolley EA  Contemporary clinical trials. Vol.96, pp.106080, 2020.  [Journal Article. Pragmatic Clinical Trial. Randomized Controlled Trial. Research Support, Non-U.S. Gov't] | Not relevant to the main subject/ wrong population or geographical location/ duplicate or repetition |
|  | Comparative pharmaceutico-analytical study of two types of kanta loha bhasmas and their clinical efficacy in the management of type 2 diabetes  CTRI/2020/05/025358  <https://trialsearch.who.int/Trial2.aspx?TrialID=CTRI/2020/05/025358>. 2020. | Not relevant to the main subject/ wrong population or geographical location/ duplicate or repetition |
|  | Pragmatic Randomized Clinical Trial to Limit Weight Gain in Pregnancy and Prevent Obesity  NCT04724330  <https://clinicaltrials.gov/show/NCT04724330>. 2021. | Not relevant to the main subject/ wrong population or geographical location/ duplicate or repetition |
|  | Determining trustworthiness and safety of remote consulting during the COVID-19 pandemic in primary healthcare for chronic disease populations in Nigeria and Tanzania  ISRCTN17941313  <https://trialsearch.who.int/Trial2.aspx?TrialID=ISRCTN17941313>. 2021. | Not relevant to the main subject/ wrong population or geographical location/ duplicate or repetition |
|  | Predictors of engagement in clinical care: coyot1 to California  Garcia JJF, Reid MW, Pyatak E, Fox DS, Fogel JL, Salcedo-Rodriguez E, Bisno DI, Miller D, Mittal A, Raymond J  Diabetes. 70(SUPPL 1):2021.  [Journal: Conference Abstract] | Not relevant to the main subject/ wrong population or geographical location/ duplicate or repetition |
|  | Comparing two telehealth interventions for patients with clinic-refractory type 2 diabetes: a randomized controlled trial  Crowley MJ, Tarkington PE, Bosworth HB, Maciejewski ML, Steinhauser K, Jeffreys AS, Coffman C, Smith V, Danus S, Jeter DH, Strawbridge E, Wilmot TC, Tisdale GA, Marcano T, Overby DL, Durkee MA, Bullard S, Dar M, Mundy A, Szabo ST, Desai S, Kobe EA, Elliott NM, Edelman D  Diabetes. 70(SUPPL 1):2021.  [Journal: Conference Abstract] | Not relevant to the main subject/ wrong population or geographical location/ duplicate or repetition |
|  | SMS supporting treatment for people with type 2 diabetes  ISRCTN70768808  <https://trialsearch.who.int/Trial2.aspx?TrialID=ISRCTN70768808>. 2015. | Not relevant to the main subject/ wrong population or geographical location/ duplicate or repetition |
|  | Pharmaceutical standardization of saptarangyadi vati prepared by two methods and their comperative effect on MADHUMEHA(TYPE-2 DIABETIS MELLITUS)  CTRI/2016/03/006769  <https://trialsearch.who.int/Trial2.aspx?TrialID=CTRI/2016/03/006769>. 2016. | Not relevant to the main subject/ wrong population or geographical location/ duplicate or repetition |
|  | Evaluation of the effectiveness of telemedicine in the management of risk and cardiovascular problems in primary health care in Cameroon  PACTR201701001964199  <https://trialsearch.who.int/Trial2.aspx?TrialID=PACTR201701001964199>. 2017. | Not relevant to the main subject/ wrong population or geographical location/ duplicate or repetition |
|  | Efficacy and safety of oral semaglutide versus placebo in subjects with type 2 diabetes mellitus treated with diet and exercise only  EUCTR2015-005622-19-CZ  <https://trialsearch.who.int/Trial2.aspx?TrialID=EUCTR2015-005622-19-CZ>. 2016. | Not relevant to the main subject/ wrong population or geographical location/ duplicate or repetition |
|  | Pilot Mobile Phone Intervention in Promoting Type 2 Diabetes Management in an Urban Area in Ghana: a Randomized Controlled Trial  Asante E, Bam V, Diji AK, Lomotey AY, Owusu Boateng A, Sarfo-Kantanka O, Oparebea Ansah E, Adjei D  Diabetes educator. 46(5):455-464, 2020.  [Journal Article. Randomized Controlled Trial. Research Support, Non-U.S. Gov't] | Not relevant to the main subject/ wrong population or geographical location/ duplicate or repetition |
|  | Educational films for improving screening and self-management of gestational diabetes in India and Uganda (GUIDES): study protocol for a cluster-randomised controlled trial  Oakley LL, R D, Namara A, Sahu B, Nadal IP, Ana Y, Coombe H, Oteng-Ntim E, Seeley J, Nyirenda M, Babu G, Kinra S | Not relevant to the main subject/ wrong population or geographical location/ duplicate or repetition |
|  | Engaging African American patients with diabetes to form a patient advisory council for the management of diabetes in everyday life  Gutierrez ML, Bailey JE, Lopez L, Tate R  Journal of general internal medicine. 36(SUPPL 1):S64-, 2021.  [Journal: Conference Abstract] | Not relevant to the main subject/ wrong population or geographical location/ duplicate or repetition |
|  | Self-measured blood pressure monitoring (SBPM) during the COVID-19 pandemic  Girma B, Curtis NC  Journal of general internal medicine. 36(SUPPL 1):S138-, 2021.  [Journal: Conference Abstract] | Not relevant to the main subject/ wrong population or geographical location/ duplicate or repetition |
|  | Process evaluation of a brief messaging intervention to improve diabetes treatment adherence in sub-Saharan Africa  Leon N, Namadingo H, Cooper S, Bobrow K, Mwantisi C, Nyasulu M, Sicwebu N, Crampin A, Levitt N, Farmer A  BMC public health. 21(1):1576, 2021.  [Journal Article. Randomized Controlled Trial. Research Support, Non-U.S. Gov't] | Not relevant to the main subject/ wrong population or geographical location/ duplicate or repetition |
|  | Using mHealth (Mobile Health) to Optimize Glycemic Control in Adults With Type 2 Diabetes: proof of Concept Study  NCT05013294  <https://clinicaltrials.gov/show/NCT05013294>. 2021. | Not relevant to the main subject/ wrong population or geographical location/ duplicate or repetition |
|  | Advancing DSME/S and COVID-19 Prevention and Protection Through "emPOWERed to Change" Program  NCT04993326  <https://clinicaltrials.gov/show/NCT04993326>. 2021. | Not relevant to the main subject/ wrong population or geographical location/ duplicate or repetition |
|  | A Faithful Response to COVID-19 Project  NCT04978207  <https://clinicaltrials.gov/show/NCT04978207>. 2021. | Not relevant to the main subject/ wrong population or geographical location/ duplicate or repetition |
|  | A mobile personal health record for behavioral health homes: preliminary results from a randomized trial  Druss B  Schizophrenia bulletin. Vol.45, pp.S134-S135, 2019.  [Journal: Conference Abstract] | Not relevant to the main subject/ wrong population or geographical location/ duplicate or repetition |
|  | Telephone-Delivered Behavioral Skills Intervention for African American Adults with Type 2 Diabetes: a Randomized Controlled Trial  Egede LE, Williams JS, Voronca DC, Gebregziabher M, Lynch CP  Journal of general internal medicine. 32(7):775-782, 2017.  [Journal Article. Randomized Controlled Trial] | Not relevant to the main subject/ wrong population or geographical location/ duplicate or repetition |
|  | The effect of morning versus evening administration of empagliflozin on its pharmacokinetics and pharmacodynamics characteristics in healthy adults: a two-way crossover, non-randomised trial  ElDash RM, Raslan MA, Shaheen SM, Sabri NA  F1000research. Vol.10, 2021.  [Journal: Article] | Not relevant to the main subject/ wrong population or geographical location/ duplicate or repetition |
|  | Philadelphia Telemedicine Glaucoma Detection and Follow-up Study: ocular Findings at Two Health Centers  Hark L, Acito M, Adeghate J, Henderer J, Okudolo J, Malik K, Molineaux J, Eburuoh R, Zhan T, Katz LJ  Journal of health care for the poor and underserved. 29(4):1400-1415, 2018.  [Journal Article. Randomized Controlled Trial. Research Support, U.S. Gov't, P.H.S] | Not relevant to the main subject/ wrong population or geographical location/ duplicate or repetition |
|  | Effect of a nurse-led lifestyle choice and coaching intervention on systolic blood pressure among type 2 diabetic patients with a high atherosclerotic cardiovascular risk: study protocol for a cluster-randomized trial  Lumu W, Kibirige D, Wesonga R, Bahendeka S  Trials. 22(1):2021.  [Journal: Article] | Not relevant to the main subject/ wrong population or geographical location/ duplicate or repetition |
|  | Using a Theory-based SMS/VM Intervention to Improve Sexual and Reproductive Health of Female Entertainment Workers in Cambodia  NCT03117842  <https://clinicaltrials.gov/show/NCT03117842>. 2017. | Not relevant to the main subject/ wrong population or geographical location/ duplicate or repetition |
|  | Efficacy, acceptability and feasibility of daily text-messaging in promoting glycaemic control and other clinical outcomes in a low-resource setting of South Africa: a randomised controlled trial  Owolabi EO, Goon DT, Ajayi AI  PloS one. 14(11):e0224791, 2019.  [Journal Article. Multicenter Study. Randomized Controlled Trial. Research Support, Non-U.S. Gov't] | Not relevant to the main subject/ wrong population or geographical location/ duplicate or repetition |
|  | Impact of mobile phone text messaging intervention on adherence among patients with diabetes in a rural setting: a randomized controlled trial  Owolabi EO, Goon DT, Ajayi AI  Medicine. 99(12):e18953, 2020.  [Journal Article. Multicenter Study. Randomized Controlled Trial] | Not relevant to the main subject/ wrong population or geographical location/ duplicate or repetition |
|  | Knowledge of gestational diabetes mellitus at first consultation in a multi-ethnic pregnant population in the Oslo region, Norway - a cross-sectional study  Borgen I, Garnweidner-Holme LM, Jacobsen AF, Fayyad S, Cvancarova Smastuen M, Lukasse M  Ethnicity & health. 1-14p. 2019.  [Journal: Article in Press] | Not relevant to the main subject/ wrong population or geographical location/ duplicate or repetition |
|  | SMS education for the promotion of diabetes self-management in low & middle income countries: a pilot randomized controlled trial in Egypt  Abaza H, Marschollek M  BMC public health. 17(1):962, 2017.  [Journal Article. Randomized Controlled Trial] | Not relevant to the main subject/ wrong population or geographical location/ duplicate or repetition |
|  | Managing uncertainty in diabetes: an intervention for older African American women  Amoako E, Skelly AH  Ethnicity & disease. 17(3):515-521, 2007.  [Journal Article. Randomized Controlled Trial] | Not relevant to the main subject/ wrong population or geographical location/ duplicate or repetition |
|  | A 24-week, multicenter, randomized, double-blind, placebo-controlled, parallel-group study of the efficacy and tolerability of combination therapy with rosiglitazone and sulfonylurea in African American and Hispanic American patients with type 2 diabetes inadequately controlled with sulfonylurea monotherapy  Davidson JA, McMorn SO, Waterhouse BR, Cobitz AR  Clinical therapeutics. 29(9):1900-1914, 2007.  [Journal Article. Multicenter Study. Randomized Controlled Trial] | Not relevant to the main subject/ wrong population or geographical location/ duplicate or repetition |
|  | Mobile telemonitoring for achieving tighter targets of blood pressure control in patients with complicated diabetes: a pilot study  Earle KA, Istepanian RS, Zitouni K, Sungoor A, Tang B  Diabetes technology & therapeutics. 12(7):575-579, 2010.  [Journal Article. Randomized Controlled Trial. Research Support, Non-U.S. Gov't] | Not relevant to the main subject/ wrong population or geographical location/ duplicate or repetition |
|  | Managing hypertension in urban underserved subjects using telemedicine--a clinical trial  Bove AA, Homko CJ, Santamore WP, Kashem M, Kerper M, Elliott DJ  American heart journal. 165(4):615-621, 2013.  [Journal Article. Randomized Controlled Trial. Research Support, U.S. Gov't, P.H.S] | Not relevant to the main subject/ wrong population or geographical location/ duplicate or repetition |
|  | Adherence to diabetes self care for white, African-American and Hispanic American telemedicine participants: 5 year results from the IDEATel project  Trief PM, Izquierdo R, Eimicke JP, Teresi JA, Goland R, Palmas W, Shea S, Weinstock RS  Ethnicity & health. 18(1):83-96, 2013.  [Journal Article. Randomized Controlled Trial. Research Support, N.I.H., Extramural. Research Support, Non-U.S. Gov't] | Not relevant to the main subject/ wrong population or geographical location/ duplicate or repetition |
|  | Technology-Intensified Diabetes Education Study (TIDES) in African Americans with type 2 diabetes: study protocol for a randomized controlled trial  Williams JS, Lynch CP, Knapp RG, Egede LE  Trials. Vol.15, pp.460, 2014.  [Journal Article. Randomized Controlled Trial. Research Support, N.I.H., Extramural] | Not relevant to the main subject/ wrong population or geographical location/ duplicate or repetition |
|  | Tablet-Aided BehavioraL intervention EffecT on Self-management skills (TABLETS) for Diabetes  Lynch CP, Williams JS, J Ruggiero K, G Knapp R, Egede LE  Trials. Vol.17, pp.157, 2016.  [Journal Article. Randomized Controlled Trial. Research Support, N.I.H., Extramural] | Not relevant to the main subject/ wrong population or geographical location/ duplicate or repetition |
|  | Randomised controlled trial of alternative messages to increase enrolment in a healthy food programme among individuals with diabetes  Gopalan A, Paramanund J, Shaw PA, Patel D, Friedman J, Brophy C, Buttenheim AM, Troxel AB, Asch DA, Volpp KG  BMJ open. 6(11):e012009, 2016.  [Comparative Study. Journal Article. Randomized Controlled Trial] | Not relevant to the main subject/ wrong population or geographical location/ duplicate or repetition |
|  | SMS Education for the Promotion of Diabetes Self-Management in Low & Middle Income Countries: a Randomized Controlled Trial in Egypt  Abaza H, Marschollek M, Schulze M  Studies in health technology and informatics. Vol.245, pp.1209, 2017.  [Journal Article. Randomized Controlled Trial] | Not relevant to the main subject/ wrong population or geographical location/ duplicate or repetition |
|  | Telemedicine cardiovascular risk reduction in veterans: the CITIES trial  Bosworth HB, Olsen MK, McCant F, Stechuchak KM, Danus S, Crowley MJ, Goldstein KM, Zullig LL, Oddone EZ  American heart journal. Vol.199, pp.122-129, 2018.  [Journal Article. Multicenter Study. Randomized Controlled Trial. Research Support, Non-U.S. Gov't] | Not relevant to the main subject/ wrong population or geographical location/ duplicate or repetition |
|  | A cluster-randomized trial to estimate the effect of mobile screening and treatment feedback on HbA1c and diabetes-related complications in Tshwane primary health care clinics, South Africa  Webb EM, Rheeder P  Primary care diabetes. 11(6):546-554, 2017.  [Journal Article. Randomized Controlled Trial. Research Support, Non-U.S. Gov't] | Not relevant to the main subject/ wrong population or geographical location/ duplicate or repetition |
|  | Clinical Inertia in a Randomized Trial of Telemedicine-Based Chronic Disease Management: lessons Learned  Barton AB, Okorodudu DE, Bosworth HB, Crowley MJ  Telemedicine journal and e-health. 24(10):742-748, 2018.  [Journal Article. Randomized Controlled Trial. Research Support, Non-U.S. Gov't. Research Support, U.S. Gov't, Non-P.H.S] | Not relevant to the main subject/ wrong population or geographical location/ duplicate or repetition |
|  | Rationale and design of Smart Walk: a randomized controlled pilot trial of a smartphone-delivered physical activity and cardiometabolic risk reduction intervention for African American women  Joseph RP, Ainsworth BE, Vega-Lopez S, Adams MA, Hollingshead K, Hooker SP, Todd M, Gaesser GA, Keller C  Contemporary clinical trials. Vol.77, pp.46-60, 2019.  [Clinical Trial Protocol. Journal Article. Randomized Controlled Trial. Research Support, N.I.H., Extramural] | Not relevant to the main subject/ wrong population or geographical location/ duplicate or repetition |
|  | A Church-Based Weight Loss Intervention in African American Adults using Text Messages (LEAN Study): cluster Randomized Controlled Trial  Newton RL, Carter LA, Johnson W, Zhang D, Larrivee S, Kennedy BM, Harris M, Hsia DS  Journal of medical Internet research. 20(8):e256, 2018.  [Journal Article. Randomized Controlled Trial. Research Support, N.I.H., Extramural. Research Support, Non-U.S. Gov't] | Not relevant to the main subject/ wrong population or geographical location/ duplicate or repetition |
|  | Effect of Nigella Sativa oil versus metformin on glycemic control and biochemical parameters of newly diagnosed type 2 diabetes mellitus patients  Moustafa HAM, El Wakeel LM, Halawa MR, Sabri NA, El-Bahy AZ, Singab AN  Endocrine. 65(2):286-294, 2019.  [Comparative Study. Journal Article. Randomized Controlled Trial] | Not relevant to the main subject/ wrong population or geographical location/ duplicate or repetition |
|  | The effect of text message support on diabetes self-management in developing countries - A randomised trial  Van Olmen J, Kegels G, Korachais C, de Man J, Van Acker K, Kalobu JC, van Pelt M, Ku GM, Hen H, Kanda D, Malombo B, Darras C, Schellevis F  Journal of clinical and translational endocrinology. Vol.7, pp.33-41, 2017.  [Journal: Article] | Not relevant to the main subject/ wrong population or geographical location/ duplicate or repetition |
|  | The use of text messaging for improving adherence to anti-diabetic regimen and glycaemic control in low-resource settings of South Africa: a study protocol for a randomised controlled trial  Owolabi EO, Goon DT  Contemporary clinical trials communications. Vol.15, 2019.  [Journal: Article] | Not relevant to the main subject/ wrong population or geographical location/ duplicate or repetition |
|  | Community-and mHealth-based integrated management of diabetes in primary healthcare in Rwanda (D2;Rwanda): the protocol of a mixed-methods study including a cluster randomised controlled trial  Lygidakis C, Uwizihiwe JP, Kallestrup P, Bia M, Condo J, Vogele C  BMJ open. 9(7):2019.  [Journal: Article] | Not relevant to the main subject/ wrong population or geographical location/ duplicate or repetition |
|  | Adapting the Diabetes Prevention Program for low and middle-income countries: protocol for a cluster randomised trial to evaluate 'Lifestyle Africa'  Catley D, Puoane T, Tsolekile L, Resnicow K, Fleming K, Hurley EA, Smyth JM, Vitolins MZ, Lambert EV, Levitt N, Goggin K  BMJ open. 9(11):2019.  [Journal: Article] | Not relevant to the main subject/ wrong population or geographical location/ duplicate or repetition |
|  | Pilot Mobile Phone Intervention in Promoting Type 2 Diabetes Management in an Urban Area in Ghana: a Randomized Controlled Trial  Asante E, Bam V, Diji AK, Lomotey AY, Owusu Boateng A, Sarfo-Kantanka O, Oparebea Ansah E, Adjei D  Diabetes educator. 46(5):455-464, 2020.  [Academic Journal] | Not relevant to the main subject/ wrong population or geographical location/ duplicate or repetition |
|  | Practical telehealth to improve control and engagement for patients with clinic-refractory diabetes mellitus (PRACTICE-DM): protocol and baseline data for a randomized trial  Kobe EA, Edelman D, Tarkington PE, Bosworth HB, Maciejewski ML, Steinhauser K, Jeffreys AS, Coffman CJ, Smith VA, Strawbridge EM, Szabo ST, Desai S, Garrett MP, Wilmot TC, Marcano TJ, Overby DL, Tisdale GA, Durkee M, Bullard S, Dar MS, Mundy AC, Hiner J, Fredrickson SK, Majette Elliott NT, Howard T, Jeter DH, Danus S, Crowley MJ  Contemporary clinical trials. Vol.98, 2020.  [Journal: Article] | Not relevant to the main subject/ wrong population or geographical location/ duplicate or repetition |
|  | Acceptability, Feasibility and Effectiveness of a Worksite Intervention to Lower Cardiometabolic Risk in South Africa  NCT04494139  <https://clinicaltrials.gov/show/NCT04494139>. 2020. | Not relevant to the main subject/ wrong population or geographical location/ duplicate or repetition |
|  | Comparison of SMS and IVR Surveys in Tanzania  NCT04506918  <https://clinicaltrials.gov/show/NCT04506918>. 2020. | Not relevant to the main subject/ wrong population or geographical location/ duplicate or repetition |
|  | Hesperidin and Diosmin for Treatment of COVID-19  NCT04452799  <https://clinicaltrials.gov/show/NCT04452799>. 2020. | Not relevant to the main subject/ wrong population or geographical location/ duplicate or repetition |
|  | HEALTHY DIET/LIFESTYLE TEXT- MESSAGE BASED INTERVENTION IN PATIENTS WITH CHRONIC KIDNEY DISEASE  Kagalwalla M, Iddrisu M-D, Missikpode C, Frydrych A, Wilk M, Gerber B, Sharp L, Lash J, Cedillo-Couvert E, Porter A  American journal of kidney diseases. 75(4):592-, 2020.  [Journal: Conference Abstract] | Not relevant to the main subject/ wrong population or geographical location/ duplicate or repetition |
|  | Adapting the Diabetes Prevention Program for low- and middle-income countries: preliminary implementation findings from lifestyle Africa  Catley D, Puoane T, Goggin K, Tsolekile LP, Resnicow K, Fleming K, Smyth JM, Hurley EA, Schlachter S, Vitolins MZ, Lambert EV, Hassen M, Muhali K, Schoor R  Translational behavioral medicine. 10(1):46-54, 2018.  [Journal: Article] | Not relevant to the main subject/ wrong population or geographical location/ duplicate or repetition |
|  | Adherence to Dietary Regimen and Recommended Physical Activity: a missing Link in the Management of Diabetes in Low-Resource Settings of Eastern Cape South Africa  Owolabi EO  Metabolism: clinical and experimental. Vol.104, 2020.  [Journal: Conference Abstract] | Not relevant to the main subject/ wrong population or geographical location/ duplicate or repetition |
|  | High Level of Acceptability and Feasibility with Low Level of Efficacy of Daily Text-Messaging on Glycaemic Status and Self-Management: result of a Randomised Trial Among Low-Income Earning Black South Africans  Owolabi EO, Ter Goon D, Ajayi AI  Metabolism: clinical and experimental. Vol.104, 2020.  [Journal: Conference Abstract] | Not relevant to the main subject/ wrong population or geographical location/ duplicate or repetition |
|  | Efficacy of Ivabradine Versus Propranolol Premedication During Hypotensive Anesthesia in Endoscopic Sinus Surgery  NCT04208594  <https://clinicaltrials.gov/show/NCT04208594>. 2019. | Not relevant to the main subject/ wrong population or geographical location/ duplicate or repetition |
|  | Health Beliefs, Glycemic Control, and Preventing Cognitive Decline in African Americans With Diabetes and Mild Cognitive Impairment: a Randomized Clinical Trial  NCT04259047  <https://clinicaltrials.gov/show/NCT04259047>. 2020. | Not relevant to the main subject/ wrong population or geographical location/ duplicate or repetition |
|  | Pharmacosurveillance and Pharmacogenetics of First-line Diuretics in Hypertension: the StayOnDiur Study  NCT00408512  <https://clinicaltrials.gov/show/NCT00408512>. 2006. | Not relevant to the main subject/ wrong population or geographical location/ duplicate or repetition |
|  | Supporting Post Myocardial Infarction (MI) Risk Modification Intervention Via Telemedicine Evaluation  NCT00901277  <https://clinicaltrials.gov/show/NCT00901277>. 2009. | Not relevant to the main subject/ wrong population or geographical location/ duplicate or repetition |
|  | Cardiovascular Intervention Improvement Telemedicine Study  NCT01142908  <https://clinicaltrials.gov/show/NCT01142908>. 2010. | Not relevant to the main subject/ wrong population or geographical location/ duplicate or repetition |
|  | Effect of homocysteine-lowering therapy on diabetic nephropathy in children and adolescents with type 1 diabetes  Elbarbary N, Ismail EAR, Zaki MA, Ibrahim MZ, El-Hamamsy M  Hormone research in paediatrics. Vol.90, pp.66-, 2018.  [Journal: Conference Abstract] | Not relevant to the main subject/ wrong population or geographical location/ duplicate or repetition |
|  | Effectiveness of Technologically Enhanced Peer Support in Improving Glycemic Management Among Predominantly African American, Low-Income Adults With Diabetes  Heisler M, Choi H, Mase R, Long JA, Reeves PJ  Diabetes educator. 45(3):260-271, 2019.  [Journal Article. Randomized Controlled Trial. Research Support, N.I.H., Extramural. Research Support, U.S. Gov't, Non-P.H.S] | Not relevant to the main subject/ wrong population or geographical location/ duplicate or repetition |
|  | Clopidogrel for High Atherothrombotic Risk and Ischemic Stabilization, Management and Avoidance (CHARISMA)  NCT00050817  <https://clinicaltrials.gov/show/NCT00050817>. 2002. | Not relevant to the main subject/ wrong population or geographical location/ duplicate or repetition |
|  | Progression of HIV-Disease Under Low Dose Corticosteroids  NCT01299948  <https://clinicaltrials.gov/show/NCT01299948>. 2011. | Not relevant to the main subject/ wrong population or geographical location/ duplicate or repetition |
|  | Effect of Vitamin D Supplementation on Muscular Strength, Musculoskeletal Pain and Headache  NCT01263288  <https://clinicaltrials.gov/show/NCT01263288>. 2010. | Not relevant to the main subject/ wrong population or geographical location/ duplicate or repetition |
|  | SMS Technology for the Promotion of Diabetes Education and Self-Management in Egypt  NCT02868320  <https://clinicaltrials.gov/show/NCT02868320>. 2016. | Not relevant to the main subject/ wrong population or geographical location/ duplicate or repetition |
|  | The Effects of Potassium on Glucose Metabolism in African Americans  NCT02236598  <https://clinicaltrials.gov/show/NCT02236598>. 2014. | Not relevant to the main subject/ wrong population or geographical location/ duplicate or repetition |
|  | Tablet-Aided BehavioraL Intervention EffecT on Self-management Skills  NCT02128854  <https://clinicaltrials.gov/show/NCT02128854>. 2014. | Not relevant to the main subject/ wrong population or geographical location/ duplicate or repetition |
|  | COllaborative Shared Care to IMprove Psychosis Outcome  NCT02895269  <https://clinicaltrials.gov/show/NCT02895269>. 2016. | Not relevant to the main subject/ wrong population or geographical location/ duplicate or repetition |
|  | Interactive Mobile Messaging for Weight Control Among the Underserved  NCT01814644  <https://clinicaltrials.gov/show/NCT01814644>. 2013. | Not relevant to the main subject/ wrong population or geographical location/ duplicate or repetition |
|  | To Study the Nutri-Genomic Response of Vit-D Supplementation in African-Americans  NCT02802449  <https://clinicaltrials.gov/show/NCT02802449>. 2016. | Not relevant to the main subject/ wrong population or geographical location/ duplicate or repetition |
|  | Evaluation of Novartis Access; a Non-communicable Disease (NCD) Access Initiative  NCT02773095  <https://clinicaltrials.gov/show/NCT02773095>. 2016. | Not relevant to the main subject/ wrong population or geographical location/ duplicate or repetition |
|  | Community- and mHealth-Based Integrated Management of Diabetes in Primary Healthcare in Rwanda  NCT03376607  <https://clinicaltrials.gov/show/NCT03376607>. 2017. | Not relevant to the main subject/ wrong population or geographical location/ duplicate or repetition |
|  | The Philani Mobile Video Intervention for Exclusive Breastfeeding (MOVIE) Study  NCT03688217  <https://clinicaltrials.gov/show/NCT03688217>. 2018. | Not relevant to the main subject/ wrong population or geographical location/ duplicate or repetition |
|  | The Diabetes TeleCare Study  NCT00288132  <https://clinicaltrials.gov/show/NCT00288132>. 2006. | Not relevant to the main subject/ wrong population or geographical location/ duplicate or repetition |
|  | Treating Obesity in Underserved Overweight Populations  NCT00373230  <https://clinicaltrials.gov/show/NCT00373230>. 2006. | Not relevant to the main subject/ wrong population or geographical location/ duplicate or repetition |
|  | Reducing Emergency Diabetes Care for Older African Americans  NCT03466866  <https://clinicaltrials.gov/show/NCT03466866>. 2018. | Not relevant to the main subject/ wrong population or geographical location/ duplicate or repetition |
|  | Gestational Diabetes in Uganda and India Improving Screening and Self-management  NCT03937050  <https://clinicaltrials.gov/show/NCT03937050>. 2019. | Not relevant to the main subject/ wrong population or geographical location/ duplicate or repetition |
|  | Efficacy, feasibility and acceptability of mHealth technology (SMS) for promoting glycaemic status and self-management among lowincome earning adults in Eastern Cape, South Africa  Owolabi EO, Goon DT  Trials. Vol.20, 2019.  [Journal: Conference Abstract] | Not relevant to the main subject/ wrong population or geographical location/ duplicate or repetition |
|  | Telehealth for Weight Maintenance of African-American Women ("Exercise Your Faith")  NCT01402557  <https://clinicaltrials.gov/show/NCT01402557>. 2011. | Not relevant to the main subject/ wrong population or geographical location/ duplicate or repetition |
|  | Steering Together in a New Direction: reducing the Risk of HIV/STD Among African American Men  NCT02572401  <https://clinicaltrials.gov/show/NCT02572401>. 2015. | Not relevant to the main subject/ wrong population or geographical location/ duplicate or repetition |
|  | Effectiveness Trial of an E-Health Intervention To Support Diabetes Care in Minority Youth (3Ms)  NCT03168867  <https://clinicaltrials.gov/show/NCT03168867>. 2017. | Not relevant to the main subject/ wrong population or geographical location/ duplicate or repetition |
|  | The Efficacy of Specialist Collaboration and Mobile Screening for Improving the Management of Diabetes  NCT01275040  <https://clinicaltrials.gov/show/NCT01275040>. 2010. | Not relevant to the main subject/ wrong population or geographical location/ duplicate or repetition |
|  | Financial Incentives and Text Messaging to Improve African American Womens' Glycemic Control  NCT02384265  <https://clinicaltrials.gov/show/NCT02384265>. 2015. | Not relevant to the main subject/ wrong population or geographical location/ duplicate or repetition |
|  | CHAMPS Study: chronic HepAtitis C Management to ImProve OutcomeS  NCT02402218  <https://clinicaltrials.gov/show/NCT02402218>. 2015. | Not relevant to the main subject/ wrong population or geographical location/ duplicate or repetition |
|  | Impact of Providing Medical Records in a Patient-Centered, Community Pharmacy Based, HIV Care Model (HIV-MOI)  NCT03437694  <https://clinicaltrials.gov/show/NCT03437694>. 2018. | Not relevant to the main subject/ wrong population or geographical location/ duplicate or repetition |
|  | A Mobile Based Diabetes Prevention Program  NCT01579292  <https://clinicaltrials.gov/show/NCT01579292>. 2012. | Not relevant to the main subject/ wrong population or geographical location/ duplicate or repetition |
|  | Iron Supplementation Using Total Dose Infusion and Oral Routes for Treatment of Iron Deficiency Anemia in Pregnancy  NCT02086838  <https://clinicaltrials.gov/show/NCT02086838>. 2014. | Not relevant to the main subject/ wrong population or geographical location/ duplicate or repetition |
|  | Quit and Fit: a Tobacco Cessation and Energy Balance Pilot for African Americans  NCT02103582  <https://clinicaltrials.gov/show/NCT02103582>. 2014. | Not relevant to the main subject/ wrong population or geographical location/ duplicate or repetition |
|  | Fit and Trim for Diabetes Prevention  NCT02278939  <https://clinicaltrials.gov/show/NCT02278939>. 2014. | Not relevant to the main subject/ wrong population or geographical location/ duplicate or repetition |
|  | Clinical Trial of a Serious Game for Individuals With SCI/D  NCT02341950  <https://clinicaltrials.gov/show/NCT02341950>. 2015. | Not relevant to the main subject/ wrong population or geographical location/ duplicate or repetition |
|  | The Management of Diabetes in Everyday Life Program  NCT02957513  <https://clinicaltrials.gov/show/NCT02957513>. 2016. | Not relevant to the main subject/ wrong population or geographical location/ duplicate or repetition |
|  | mHealth for Diabetes Adherence Support  NCT02990299  <https://clinicaltrials.gov/show/NCT02990299>. 2016. | Not relevant to the main subject/ wrong population or geographical location/ duplicate or repetition |
|  | Cardiovascular Health Promotion Among African-Americans by FAITH!  NCT03084822  <https://clinicaltrials.gov/show/NCT03084822>. 2017. | Not relevant to the main subject/ wrong population or geographical location/ duplicate or repetition |
|  | Community- and mHealth-Based Integrated Management of Diabetes in Primary Healthcare in Rwanda  NCT03376607  <https://clinicaltrials.gov/show/NCT03376607>. 2017. | Not relevant to the main subject/ wrong population or geographical location/ duplicate or repetition |
|  | The Effectiveness of SMS in Improving Antiretroviral Medication Adherence Among Adolescents Living With HIV in Nigeria  NCT03394391  <https://clinicaltrials.gov/show/NCT03394391>. 2017. | Not relevant to the main subject/ wrong population or geographical location/ duplicate or repetition |
|  | Omarigliptin & Trelagliptin in Twelve Healthy Egyptian Volunteers  NCT03362398  <https://clinicaltrials.gov/show/NCT03362398>. 2017. | Not relevant to the main subject/ wrong population or geographical location/ duplicate or repetition |
|  | Technology Delivered Diabetes-Modified Behavioral Activation Treatment  NCT03593694  <https://clinicaltrials.gov/show/NCT03593694>. 2018. | Not relevant to the main subject/ wrong population or geographical location/ duplicate or repetition |
|  | Corrigendum: how do smart device apps for diabetes self-management correspond with theoretical indicators of empowerment? an analysis of app features (International Journal of Technology Assessment in Health Care (2019) 35 (150-159) DOI: 10.1017/S0266462319000163)  International journal of technology assessment in health care. 35(3):252-, 2019.  [Journal: Erratum] | Not relevant to the main subject/ wrong population or geographical location/ duplicate or repetition |
|  | Philadelphia telemedicine glaucoma detection and follow-up study: interphysician agreement for screening images  Kolomeyer NN, Hark LA, Katz LJ, Myers JS, Lee D, Razeghinejad MR, Leite S, Rahmatnejad K, Zhan T, Leiby B, Hegarty S, Lee PP  Investigative ophthalmology & visual science. 59(9):2018.  [Journal: Conference Abstract] | Not relevant to the main subject/ wrong population or geographical location/ duplicate or repetition |
|  | Philadelphia telemedicine glaucoma detection and follow-up study: adherence to community eye exam appointments  Hark LA, Katz LJ, Myers JS, Haller JA  Investigative ophthalmology & visual science. 59(9):2018.  [Journal: Conference Abstract] | Not relevant to the main subject/ wrong population or geographical location/ duplicate or repetition |
|  | A randomized trial using mobile short-text messaging to improve cardiovascular risk profile in poorly controlled diabetes in kenya  Soin G, Kunyiha N, Shah J, Patel K, Arisi C, Njenga E, Jeilan M, Sorathia S, Ngunga LM, Barasa AL  Circulation. Vol.138, 2018.  [Journal: Conference Abstract] | Not relevant to the main subject/ wrong population or geographical location/ duplicate or repetition |
|  | Effects of ginger powder supplementation on glycemic status and lipid profile in newly diagnosed obese patients with type 2 diabetes mellitus  El Gayar MH, Aboromia MMM, Ibrahim NA, Abdel Hafiz MH  Obesity medicine. Vol.14, 2019.  [Journal: Article] | Not relevant to the main subject/ wrong population or geographical location/ duplicate or repetition |
|  | An ICT-Based Diabetes Management System Tested for Health Care Delivery in the African Context  Takenga C, Berndt RD, Musongya O, Kitero J, Katoke R, Molo K, Kazingufu B, Meni M, Vikandy M, Takenga H  International journal of telemedicine and applications. Vol.2014, pp.437307, 2014.  [Journal Article] | Not relevant to the main subject/ wrong population or geographical location/ duplicate or repetition |
|  | SMS-based intervention in type 2 diabetes: clinical trial in Senegal  BMJ innovations. 2018.  [Article In Press] | Not relevant to the main subject/ wrong population or geographical location/ duplicate or repetition |
|  | Adiponectin and all-cause mortality in a cohort of elderly people with type 2 diabetes  Rohman Singer J, Palmas W, Shea S, Alejandro Luchsinger J  Journal of general internal medicine. Vol.26, pp.S5-, 2011.  [Journal: Conference Abstract] | Not relevant to the main subject/ wrong population or geographical location/ duplicate or repetition |
|  | Adiponectin and all-cause mortality in a cohort of elderly people with type 2 diabetes  Singer JR, Palmas W, Shea S, Luchsinger JA  Journal of general internal medicine. 26(10):1219-1220, 2011.  [Journal: Conference Abstract] | Not relevant to the main subject/ wrong population or geographical location/ duplicate or repetition |
|  | Real-time tele-monitoring of glucose as adjunct to the management of type 2 diabetes in primary care  Heudebert A, Eichold B, Arrieta MI, Roach D, Brown S, Hansberry S, Brye W, Steigler S, Mitchell F, Oliver B, Foreman R, Moody K, Crook E  Journal of investigative medicine. 61(2):398-, 2013.  [Journal: Conference Abstract] | Not relevant to the main subject/ wrong population or geographical location/ duplicate or repetition |
|  | A tailored breastfeeding support intervention for women with gestational diabetes  Stuebe AM, Bonuck K, Adatorwovor R, Schwartz TA, Berry D  American journal of obstetrics and gynecology. 214(1):S68-, 2016.  [Journal: Conference Abstract] | Not relevant to the main subject/ wrong population or geographical location/ duplicate or repetition |
|  | Textmessagingto improve outcomes in patients with painful diabetic peripheral neuropathy (PDPN)  Bauer V, Wang C-H, Goodman N, Craig TL, Glosner S, Juhn M, Cappelleri JC, Sadosky A, Cooley C, Lapin B, Masi C  Journal of general internal medicine. 32(2):S328-, 2017.  [Journal: Conference Abstract] | Not relevant to the main subject/ wrong population or geographical location/ duplicate or repetition |
|  | Development and feasibility of the reach tailored text messaging intervention for low-income adults with type 2 diabetes  Nelson LA, Mayberry LS, Wallston KA, Kripalani S, Gentry CK, Brown A, Gregory BP, Acuff SW, Harper KJ, Bergner EM, Lestourgeon LM, Mcphillips SE, Gebretsadik T, Elasy TA, Johnson KB, Osborn CY  Diabetes. Vol.65, pp.A207-, 2016.  [Journal: Conference Abstract] | Not relevant to the main subject/ wrong population or geographical location/ duplicate or repetition |
|  | 2017 North American Forum on Family Planning Scientific Abstracts  Contraception. 96(4):2017.  [Journal: Conference Review] | Not relevant to the main subject/ wrong population or geographical location/ duplicate or repetition |
|  | SMFM 39th Annual Meeting-The Pregnancy Meeting  American journal of obstetrics and gynecology. 220(1):A1-A50, 2019.  [Journal: Conference Review] | Not relevant to the main subject/ wrong population or geographical location/ duplicate or repetition |
|  | Proposing a risk-factor based clinical app to predict outcomes following initial hospitalization in patients with multiple sclerosis  Sharma K, Fattal D, Kamholz J, Bittner F  Multiple sclerosis journal. 24(1):123-, 2018.  [Journal: Conference Abstract] | Not relevant to the main subject/ wrong population or geographical location/ duplicate or repetition |
|  | Philadelphia telemedicine glaucoma detection and follow-up study: methods and visit 1 results  Hark LA, Katz LJ, Waisbourd M, Myers JS, Johnson D, Fudemberg SS, Mantravadi A, Henderer JD, Bui TD, Lee J, Haller JA  Investigative ophthalmology & visual science. 58(8):2017.  [Journal: Conference Abstract] | Not relevant to the main subject/ wrong population or geographical location/ duplicate or repetition |
|  | Baseline health utility scores in subjects with suspected ocular disease: an interim analysis of the philadelphia telemedicine glaucoma detection and follow-up study  Mehta R, Prioli KM, Hark L, Katz LJ, Myers JS, Pizzi LT  Value in health. Vol.21, pp.S126-, 2018.  [Journal: Conference Abstract] | Not relevant to the main subject/ wrong population or geographical location/ duplicate or repetition |
|  | Characteristics of underserved adults enrolled in the mobile diabetes detective (MoDD) randomized controlled trial  Heitkemper E, Mamykina L, Cassells A, Tobin J, Smaldone A  Diabetes. Vol.66, pp.A188-A189, 2017.  [Journal: Conference Abstract] | Not relevant to the main subject/ wrong population or geographical location/ duplicate or repetition |
|  | Short-term efficacy of the combination of perindopril-amlodipine versus perindopril-indapamide on blood pressure control in a population of type 2 diabetics in sub-saharan africa  Florence KSM  Journal of hypertension. Vol.36, pp.e243-, 2018.  [Journal: Conference Abstract] | Not relevant to the main subject/ wrong population or geographical location/ duplicate or repetition |
|  | Community health workers, mobile health, or both for management of medicaid patients with diabetes  Katz RJ, Nunlee-Bland G, Magee MF, Young H, Witkin L, Nassar C, Cohen JL  Diabetes. Vol.66, pp.A96-A97, 2017.  [Journal: Conference Abstract] | Not relevant to the main subject/ wrong population or geographical location/ duplicate or repetition |
|  | Managing hypertension in urban underserved subjects using telemedicine: a clinical trial  Bove AA, Homko C, Santamore W, Kashem M, Kerper M, Elliott D  Journal of the american college of cardiology. Conference: 62nd annual scientific session of the american college of cardiology and i2 summit: innovation in intervention, ACC.13. San francisco, CA united states. 61(10 Suppl 1):E1401, 2013. | Not relevant to the main subject/ wrong population or geographical location/ duplicate or repetition |
|  | Process evaluation of a mobile health intervention for people with diabetes in low income countries - the implementation of the TEXT4DSM study  Journal of telemedicine and telecare. 23(1):96-105, 2017.  [Article] | Not relevant to the main subject/ wrong population or geographical location/ duplicate or repetition |
|  | Philadelphia telemedicine glaucoma detection and follow-up study: methods and visit 1 results  Hark LA, Katz LJ, Waisbourd M, Myers JS, Johnson D, Fudemberg SS, Mantravadi A, Henderer JD, Bui TD, Lee J, Haller JA  Investigative ophthalmology and visual science. Conference:. 2017 annual meeting of the association for research in vision and ophthalmology, ARVO 2017. United states 58(8) (no pagination):2017.  [Conference Abstract] | Not relevant to the main subject/ wrong population or geographical location/ duplicate or repetition |
|  | Racialvariations in medical care spending patterns among high-risk primary care patients: results from the STOP-DKD study  Machen L, Davenport C, Oakes M, Patel U, Diamantidis CJ  Journal of general internal medicine. Conference: 40th annual meeting of the society of general internal medicine, SGIM. 2017. United states 32(2 Supplement 1):S299, 2017.  [Conference Abstract] | Not relevant to the main subject/ wrong population or geographical location/ duplicate or repetition |
|  | Rationale, design, and methodology for the optimizing outcomes in women with gestational diabetes mellitus and their infants study  Berry DC, Neal M, Hall EG, Schwartz TA, Verbiest S, Bonuck K, Goodnight W, Brody S, Dorman KF, Menard MK, Stuebe AM  BMC pregnancy and childbirth. Vol.13, 2013.  [Journal: Article] | Not relevant to the main subject/ wrong population or geographical location/ duplicate or repetition |
|  | A complex behavioural change intervention to reduce the risk of diabetes and prediabetes in the pre-conception period in Malaysia: study protocol for a randomised controlled trial  Skau JKH, Nordin ABA, Cheah JCH, Ali R, Zainal R, Aris T, Ali ZM, Matzen P, Biesma R, Aagaard-Hansen J, Hanson MA, Norris SA  Trials. 17(1) (no pagination):2016.  [Journal: Article] | Not relevant to the main subject/ wrong population or geographical location/ duplicate or repetition |
|  | A controlled comparison of the effect of a high fiber diet on the glycaemic and lipid profile of Nigerian clinic patients with type 2 diabetes  Ikem RT, Kolawole BA, Ojofeitimi EO, Salawu A, Ajose OA, Abiose S, Odewale F  Pakistan journal of nutrition. 6(2):111-116, 2007.  [Journal: Article] | Not relevant to the main subject/ wrong population or geographical location/ duplicate or repetition |
|  | Randomized double blind placebo-controlled trial of nitazoxanide in the treatment of patients with chronic hepatitis C genotype 4  Rossignol JF, Kabil SM, El-Gohary Y, Keeffe EB  Journal of hepatology. 48(Suppl 2):S311, 2008. | Not relevant to the main subject/ wrong population or geographical location/ duplicate or repetition |
|  | Diabetes TeleCare: dissemination Research in Underserved Communities  Davis RM, Hitch A, Dorman P, Wigfall L, Zhen H, Mayer-Davis EJ  IOVS. Vol.47, pp.ARVO E-abstract 1013, 2006. | Not relevant to the main subject/ wrong population or geographical location/ duplicate or repetition |
|  | A cluster-randomized trial to estimate the effect of mobile screening and treatment feedback on HbA1c and diabetes-related complications in Tshwane primary health care clinics, South Africa  Webb EM, Rheeder P  Primary care diabetes. Vol.(no pagination), 2017.  [Article In Press] | Not relevant to the main subject/ wrong population or geographical location/ duplicate or repetition |
|  | Control of blood pressure and cardiovascular risk in Moroccan patients with newly diagnosed hypertension: a 3-month observational study in primary care  Alami M, El Hattaoui M, Seqat M, Sadik J, Aouad A, Benghanem Gharbi M  Therapeutic advances in cardiovascular disease. 11(2):49-56, 2017.  [Journal: Article] | Not relevant to the main subject/ wrong population or geographical location/ duplicate or repetition |
|  | Tablet-Aided BehavioraL intervention EffecT on Self-management skills (TABLETS) for Diabetes  Lynch CP, Williams JS, Ruggiero KJ, Knapp RG, Egede LE  Trials. 17(1) (no pagination):2016.  [Journal: Article] | Not relevant to the main subject/ wrong population or geographical location/ duplicate or repetition |
|  | Effectiveness of a scalable telemedicine intervention for veterans with persistent poor diabetes control.  Crowley MJ, Edelman D, Mcandrew AT, Kistler S, Danus S, Webb JA, Zanga J, Sanders LL, Coffman CJ, Jackson GL, Bosworth HB  Diabetes. Vol.64, pp.A80, CONFERENCE START: 2015 Jun 5 CONFERENCE END: 2015 Jun 9, 75th Scientific Sessions of the American Diabetes Association Boston, MA United States.,  [Journal: Conference Abstract] | Not relevant to the main subject/ wrong population or geographical location/ duplicate or repetition |
|  | Improving medication adherence in hypertension using home measured blood pressure and telemedicine reporting to modify patient and physician behavior.  Peters A, Rakita V, Homko C, Kothapalli P, Bove A  Journal of the American College of Cardiology. 65 (10 SUPPL. 1):A1395, CONFERENCE START: 2015 Mar 14 CONFERENCE END: 2015 Mar 16, 64th Annual Scientific Session of the American College of Cardiology and i2 Summit: Innovation in Intervention, ACC.15 San Diego, CA United States.,  [Journal: Conference Abstract | Not relevant to the main subject/ wrong population or geographical location/ duplicate or repetition |
|  | Effect of allopurinol versus angiotensin converting enzyme inhibitors in decreasing microalbuminuria in type I diabetic patients.  El-Samahy MH, Elbarbary NS, Afify MA-A, Sallam DE  Pediatric diabetes. Vol.15, pp.84, CONFERENCE START: 2014 Sep 3 CONFERENCE END: 2014 Sep 6, 40th Annual Conference of the International Society for Pediatric and Adolescent Diabetes, ISPAD 2014 Toronto, ON Canada.,  [Journal: Conference Abstract] | Not relevant to the main subject/ wrong population or geographical location/ duplicate or repetition |
|  | Race/ethnic disparities in weight and glycemia in older adults receiving lifestyle interventions via peer-leaders with or without mobile enhancement for diabetes prevention and management.  Dang S, Oropesa L, Byrne MM, Gutt M, Andrade F, Guanipa C, Sorial A, Pelaez M, Schwarzberg R, Roos B, Valencia W, Florez H  Journal of the American Geriatrics Society. Vol.62, pp.S216, CONFERENCE START: 2014 May 15 CONFERENCE END: 2014 May 17, 2014 Annual Scientific Meeting of the American Geriatrics Society Orlando, FL United States.,  [Journal: Conference Abstract] | Not relevant to the main subject/ wrong population or geographical location/ duplicate or repetition |
|  | Comparison of the antiplatelet effect of crushed clopidogrel vs. Whole tablet in diabetic patients presenting with an acute coronary syndrome.  Addad F, Oueslati C, Ibn El Hadj Z, Hammami N, Jebri F, Ben Halima A, Kammoun I, Yaalaoui S, Kachboura S  Journal of thrombosis and haemostasis : JTH. Vol.11, pp.628-9, CONFERENCE START: 2013 Jun 29 CONFERENCE END: 2013 Jul 4, 24th Congress of the International Society on Thrombosis and Haemostasis Amsterdam Netherlands.,  [Journal: Conference Abstract] | Not relevant to the main subject/ wrong population or geographical location/ duplicate or repetition |
|  | Access to mobile phone and willingness to receive mHealth services among patients with diabetes in Northwest Ethiopia: a cross-sectional study  Jemere, AT (Jemere, Adamu Takele); Yeneneh, YE (Yeneneh, Yohannes Ezezew); Tilahun, B (Tilahun, Biniam); Fritz, F (Fritz, Fleur); Alemu, S (Alemu, Shitaye); Kebede, M (Kebede, Mihiretu)  Source: BMJ OPEN  Volume: 9  Issue: 1  Article Number: e021766  DOI: 10.1136/bmjopen-2018-021766  Published: JUN 2019 | Not relevant to the main subject/ wrong population or geographical location/ duplicate or repetition |
|  | Factors influencing healthcare providers' attitude and willingness to use information technology in diabetes management  Seboka, BT (Seboka, Binyam Tariku); Yilma, TM (Yilma, Tesfahun Melese); Birhanu, AY (Birhanu, Abraham Yeneneh)  Source: BMC MEDICAL INFORMATICS AND DECISION MAKING  Volume: 21  Issue: 1  Article Number: 24  DOI: 10.1186/s12911-021-01398-w  Published: JAN 21 2021 | Not relevant to the main subject/ wrong population or geographical location/ duplicate or repetition |
|  | Digital messaging to support control for type 2 diabetes (StAR2D): a multicentre  randomised controlled trialAuthor(s): Farmer, A (Farmer, A.); Bobrow, K (Bobrow, K.); Leon, N (Leon, N.);  Williams, N (Williams, N.); Phiri, E (Phiri, E.); Namadingo, H (Namadingo, H.);  Cooper, S (Cooper, S.); Prince, J (Prince, J.); Crampin, A (Crampin, A.); Besada, D  (Besada, D.); Daviaud, E (Daviaud, E.); Yu, LM (Yu, L-M); N'goma, J (N'goma, J.);  Springer, D (Springer, D.); Pauly, B (Pauly, B.); Tarassenko, L (Tarassenko, L.); Norris,  S (Norris, S.); Nyirenda, M (Nyirenda, M.); Levitt, N (Levitt, N.)  Source: BMC PUBLIC HEALTH Volume: 21 Issue: 1 Article  Number: 1907 DOI: 10.1186/s12889-021-11874-7 Published: OCT 21 2021 | Not relevant to the main subject/ wrong population or geographical location/ duplicate or repetition |
|  | Intervention development of a brief messaging intervention for a randomised  controlled trial to improve diabetes treatment adherence in sub-Saharan Africa  Author(s): Leon, N (Leon, Natalie); Namadingo, H (Namadingo, Hazel); Bobrow, K  (Bobrow, Kirsty); Cooper, S (Cooper, Sara); Crampin, A (Crampin, Amelia); Pauly, B  (Pauly, Bruno); Levitt, N (Levitt, Naomi); Farmer, A (Farmer, Andrew)  Source: BMC PUBLIC HEALTH Volume: 21 Issue: 1 Article  Number: 147 DOI: 10.1186/s12889-020-10089-6 Published: JAN 15 2021 | Not relevant to the main subject/ wrong population or geographical location/ duplicate or repetition |
|  | EVALUATION OF A TEXT MESSAGING SYSTEM TO ENHANCE  DELIVERY AND EFFECTIVENESS OF A DIABETES PREVENTION PROGRAM  IN SOUTH AFRICA  Author(s): Materia, FT (Materia, Frank T.); Smyth, JM (Smyth, Joshua M.); Puoane, T  (Puoane, Thandi); Goggin, K (Goggin, Kathy); Tsolekile, LP (Tsolekile, Lungiswa P.);  Resnicow, K (Resnicow, Ken); Catley, D (Catley, Delwyn)  Source: ANNALS OF BEHAVIORAL MEDICINE Volume: 55 Pages: S193-  S193 Supplement: 1 Published: APR 2021  Accession Number: WOS:000648922700393  ISSN: 0883-6612  eISSN: 1532-4796 | Not relevant to the main subject/ wrong population or geographical location/ duplicate or repetition |
|  | Title: Barriers to the Use of Mobile Health in Improving Health Outcomes in  Developing Countries: Systematic Review  Author(s): Kruse, C (Kruse, Clemens); Betancourt, J (Betancourt, Jose); Ortiz, S (Ortiz,  Stephanie); Luna, SMV (Luna, Susana Melissa Valdes); Bamrah, IK (Bamrah,  Inderdeep Kaur); Segovia, N (Segovia, Narce)  Source: JOURNAL OF MEDICAL INTERNET  RESEARCH Volume: 21 Issue: 10 Article  Number: e13263 DOI: 10.2196/13263 Published: OCT 9 2019 | Not relevant to the main subject/ wrong population or geographical location/ duplicate or repetition |
|  | Impact of mobile phone text messaging intervention on adherence among patients  with diabetes in a rural setting A randomized controlled trial  Author(s): Owolabi, EO (Owolabi, Eyitayo Omolara); Ter Goon, D (Ter Goon, Daniel);  Ajayi, AI (Ajayi, Anthony Idowu)  Source: MEDICINE Volume: 99 Issue: 12 Article  Number: e18953 DOI: 10.1097/MD.0000000000018953 Published: MAR 2020 | Not relevant to the main subject/ wrong population or geographical location/ duplicate or repetition |
|  | Title: High Level of Acceptability and Feasibility with Low Level of Efficacy of Daily  Text-Messaging on Glycaemic Status and Self-Management: Result of a Randomised  Trial Among Low-Income Earning Black South Africans  Author(s): Owolabi, EO (Owolabi, Eyitayo Omolara); Ter Goon, D (Ter Goon, Daniel);  Ajayi, AI (Ajayi, Anthony Idowu)  Source: METABOLISM-CLINICAL AND EXPERIMENTAL Volume: 104 Article  Number: 154057 DOI: 10.1016/j.metabol.2019.12.003 Supplement: S Published: M  AR 2020  Accession Number: WOS:000518472500004 | Not relevant to the main subject/ wrong population or geographical location/ duplicate or repetition |
|  | A Church-Based Weight Loss Intervention in African American Adults using  Text Messages (LEAN Study): Cluster Randomized Controlled Trial  Author(s): Newton, RL (Newton, Robert L., Jr.); Carter, LA (Carter, Leah A.);  Johnson, W (Johnson, William); Zhang, DC (Zhang, Dachuan); Larrivee, S (Larrivee,  Sandra); Kennedy, BM (Kennedy, Betty M.); Harris, M (Harris, Melissa); Hsia, DS  (Hsia, Daniel S.)Source: JOURNAL OF MEDICAL INTERNET  RESEARCH Volume: 20 Issue: 8 Article  Number: e256 DOI: 10.2196/jmir.9816 Published: AUG 2018 | Not relevant to the main subject/ wrong population or geographical location/ duplicate or repetition |
|  | Efficacy, acceptability and feasibility of daily text-messaging in promoting  glycaemic control and other clinical outcomes in a low-resource setting of South Africa:  A randomised controlled trial  Author(s): Owolabi, EO (Owolabi, Eyitayo Omolara); Ter Goon, D (Ter Goon, Daniel);  Ajayi, AI (Ajayi, Anthony Idowu)  Source: PLOS ONE Volume: 14 Issue: 11 Article  Number: e0224791 DOI: 10.1371/journal.pone.0224791 Published: NOV 27 2019 | Not relevant to the main subject/ wrong population or geographical location/ duplicate or repetition |
|  | Evaluation of Text Messaging Effects on Health Goal Adherence in the  Management of Participants With Chronic Diseases  Author(s): Stagg, SJ (Stagg, Sharon J.); Speroni, KG (Speroni, Karen Gabel); Daniel,  MG (Daniel, Marlon G.); Eigenbrode, M (Eigenbrode, Melissa); Geisler, L (Geisler,  Lori)  Source: PROFESSIONAL CASE  MANAGEMENT Volume: 22 Issue: 3 Pages: 126-  135 DOI: 10.1097/NCM.0000000000000190 Published: MAY-JUN 2017 | Not relevant to the main subject/ wrong population or geographical location/ duplicate or repetition |
|  | Mobile phone messaging for facilitating self-management of long-term illnesses  Author(s): de Jongh, T (de Jongh, Thyra); Gurol-Urganci, I (Gurol-Urganci, Ipek);  Vodopivec-Jamsek, V (Vodopivec-Jamsek, Vlasta); Car, J (Car, Josip); Atun, R (Atun,  Rifat)  Source: COCHRANE DATABASE OF SYSTEMATIC REVIEWS Issue: 12 Article  Number: CD007459 DOI: 10.1002/14651858.CD007459.pub2 Published: 2012 | Not relevant to the main subject/ wrong population or geographical location/ duplicate or repetition |
|  | Adherence to Dietary Regimen and Recommended Physical Activity: A missing  Link in the Management of Diabetes in Low-Resource Settings of Eastern Cape South  Africa  Author(s): Owolabi, EO (Owolabi, Eyitayo Omolara)  Source: METABOLISM-CLINICAL AND EXPERIMENTAL Volume: 104 Article  Number: 154058 DOI: 10.1016/j.metabol.2019.12.004 Supplement: S Published: M  AR 2020  Accession Number: WOS:000518472500005  ISSN: 0026-0495  eISSN: 1532-8600 | Not relevant to the main subject/ wrong population or geographical location/ duplicate or repetition |
|  | Developing targeted client communication messages to pregnant women in  Bangladesh: a qualitative study  Author(s): Pervin, J (Pervin, Jesmin); Sarker, BK (Sarker, Bidhan Krishna); Nu, UT  (Nu, U. Tin); Khatun, F (Khatun, Fatema); Rahman, AMQ (Rahman, A. M. Quaiyum);  Venkateswaran, M (Venkateswaran, Mahima); Rahman, A (Rahman, Anisur); Froen, JF  (Froen, J. Frederik); Friberg, IK (Friberg, Ingrid K.)  Source: BMC PUBLIC HEALTH Volume: 21 Issue: 1 Article  Number: 759 DOI: 10.1186/s12889-021-10811-y Published: APR 20 2021 | Not relevant to the main subject/ wrong population or geographical location/ duplicate or repetition |
|  | Process evaluation of a mobile health intervention for people with diabetes in low  income countries - the implementation of the TEXT4DSM study  Author(s): Van Olmen, J (Van Olmen, Josefien); Van Pelt, M (Van Pelt, Maurits);  Malombo, B (Malombo, Billy); Ku, GM (Ku, Grace M.); Kanda, D (Kanda,  Dominique); Heang, H (Heang, Hen); Darras, C (Darras, Christian); Kegels, G (Kegels,  Guy); Schellevis, F (Schellevis, Francois)  Source: JOURNAL OF TELEMEDICINE AND  TELECARE Volume: 23 Issue: 1 Pages: 96-  105 DOI: 10.1177/1357633X15617885 Published: JAN 2017 | Not relevant to the main subject/ wrong population or geographical location/ duplicate or repetition |
|  | Process evaluation of a brief messaging intervention to improve diabetes  treatment adherence in sub-Saharan Africa  Author(s): Leon, N (Leon, N.); Namadingo, H (Namadingo, H.); Cooper, S (Cooper,  S.); Bobrow, K (Bobrow, K.); Mwantisi, C (Mwantisi, C.); Nyasulu, M (Nyasulu, M.);  Sicwebu, N (Sicwebu, N.); Crampin, A (Crampin, A.); Levitt, N (Levitt, N.); Farmer, A  (Farmer, A.)  Source: BMC PUBLIC HEALTH Volume: 21 Issue: 1 Article  Number: 1576 DOI: 10.1186/s12889-021-11552-8 Published: AUG 21 2021 | Not relevant to the main subject/ wrong population or geographical location/ duplicate or repetition |
|  | The Design, Usability, and Feasibility of a Family-Focused Diabetes Self-Care  Support mHealth Intervention for Diverse, Low-Income Adults with Type 2 Diabetes  Author(s): Mayberry, LS (Mayberry, Lindsay Satterwhite); Berg, CA (Berg, Cynthia  A.); Harper, KJ (Harper, Kryseana J.); Osborn, CY (Osborn, Chandra Y.)  Source: JOURNAL OF DIABETES RESEARCH Volume: 2016 Article  Number: 7586385 DOI: 10.1155/2016/7586385 Published: 2016 | Not relevant to the main subject/ wrong population or geographical location/ duplicate or repetition |
|  | Feasibility of text message sleep assessment in African American and Latino  patients with type 2 diabetes  Author(s): Biggers, A (Biggers, Alana); Henkins, J (Henkins, Julia); Barton, I (Barton,  Isaye); Hubbard, C (Hubbard, Colin); Perez, R (Perez, Rose); Sharp, LK (Sharp, Lisa  K.); Gerber, B (Gerber, Ben S.)  Source: JOURNAL OF CLINICAL SLEEP  MEDICINE Volume: 17 Issue: 1 Pages: 69-  78 DOI: 10.5664/jcsm.8828 Published: JAN 1 2021 | Not relevant to the main subject/ wrong population or geographical location/ duplicate or repetition |
|  | Characteristics of Health-related Text Messages Preferred by Medically  Underserved African-American Patients With Diabetes  Author(s): Udoko, AN (Udoko, Aniekan N.); Graff, J (Graff, Joyce); Ransone, S  (Ransone, Samantha); Coday, M (Coday, Mace); Gatwood, JD (Gatwood, Justin D.);  Bailey, JE (Bailey, James E.)  Source: CUREUS Volume: 11 Issue: 9 Article  Number: e5743 DOI: 10.7759/cureus.5743 Published: SEP 24 2019 | Not relevant to the main subject/ wrong population or geographical location/ duplicate or repetition |
|  | The management of diabetes in everyday life study: Design and methods for a  pragmatic randomized controlled trial comparing the effectiveness of text messaging  versus health coaching  Author(s): Bailey, JE (Bailey, James E.); Surbhi, S (Surbhi, Satya); Gatwood, J  (Gatwood, Justin); Butterworth, S (Butterworth, Susan); Coday, M (Coday, Mace);  Shuvo, SA (Shuvo, Sohul A.); Dashputre, AA (Dashputre, Ankur A.); Brooks, IM  (Brooks, Ian M.); Binkley, BL (Binkley, Bonnie L.); Riordan, CJ (Riordan, Carrie Jo);  Steinberg, HO (Steinberg, Helmut O.); Gutierrez, ML (Gutierrez, Mary Lou); Haley, LE  (Haley, Lauren E.); Leak, CL (Leak, Cardella L.); Tolley, EA (Tolley, Elizabeth A.)  Source: CONTEMPORARY CLINICAL TRIALS Volume: 96 Article  Number: 106080 DOI: 10.1016/j.cct.2020.106080 Published: SEP 2020 | Not relevant to the main subject/ wrong population or geographical location/ duplicate or repetition |
|  | The use of text messaging for improving adherence to anti-diabetic regimen and  glycaemic control in low-resource settings of South Africa: A study protocol for a  randomised controlled trial  Author(s): Owolabi, EO (Owolabi, Eyitayo Omolara); Ter Goon, D (Ter Goon, Daniel)  Source: CONTEMPORARY CLINICAL TRIALS  COMMUNICATIONS Volume: 15 Article  Number: 100418 DOI: 10.1016/j.conctc.2019.100418 Published: SEP 2019 | Not relevant to the main subject/ wrong population or geographical location/ duplicate or repetition |
|  | Developing a behavioral model for mobile phone-based diabetes interventions  Author(s): Nundy, S (Nundy, Shantanu); Dick, JJ (Dick, Jonathan J.); Solomon, MC  (Solomon, Marla C.); Peek, ME (Peek, Monica E.)  Source: PATIENT EDUCATION AND  COUNSELING Volume: 90 Issue: 1 Pages: 125-  132 DOI: 10.1016/j.pec.2012.09.008 Published: JAN 2013 | Not relevant to the main subject/ wrong population or geographical location/ duplicate or repetition |
|  | Is the lack of smartphone data skewing wealth indices in low-income settings?  Author(s): Poirier, MJP (Poirier, Mathieu J. P.); Barnighausen, T (Barnighausen, Till);  Harling, G (Harling, Guy); Sie, A (Sie, Ali); Grepin, KA (Grepin, Karen A.)  Source: POPULATION HEALTH METRICS Volume: 19 Issue: 1 Article  Number: 4 DOI: 10.1186/s12963-021-00246-3 Published: FEB 1 2021 | Not relevant to the main subject/ wrong population or geographical location/ duplicate or repetition |
|  | How Do Mobile Phone Diabetes Programs Drive Behavior Change? Evidence  From a Mixed Methods Observational Cohort Study  Author(s): Nundy, S (Nundy, Shantanu); Mishra, A (Mishra, Anjuli); Hogan, P (Hogan,  Patrick); Lee, SM (Lee, Sang Mee); Solomon, MC (Solomon, Marla C.); Peek, ME  (Peek, Monica E.)  Source: DIABETES EDUCATOR Volume: 40 Issue: 6 Pages: 806-  819 DOI: 10.1177/0145721714551992 Published: NOV-DEC 2014 | Not relevant to the main subject/ wrong population or geographical location/ duplicate or repetition |
|  | Claim More (TM) Empowering African American Women to Make Healthy  Choices  Author(s): Tkatch, R (Tkatch, Rifky); Musich, S (Musich, Shirley); Draklellis, J  (Draklellis, Jennifer); Hetzel, M (Hetzel, Marla); Banks, J (Banks, Jo); Dugan, J (Dugan,  Jessica); Thompson, K (Thompson, Kaylene); Hawkins, K (Hawkins, Kevin)  Source: JOURNAL OF HOLISTIC NURSING Volume: 36 Issue: 1 Pages: 91-  98 DOI: 10.1177/0898010117691167 Published: MAR 2018 | Not relevant to the main subject/ wrong population or geographical location/ duplicate or repetition |
|  | The effect of text message support on diabetes self-management in developing  countries - A randomised trial  Author(s): Van Olmen, J (Van Olmen, Josefien); Kegels, G (Kegels, Guy); Korachais,  C (Korachais, Catherine); de Man, J (de Man, Jeroen); Van Acker, K (Van Acker,  Kristien); Kalobu, JC (Kalobu, Jean Clovis); van Pelt, M (van Pelt, Maurits); Ku, GM  (Ku, Grace Marie); Hen, H (Hen, Heang); Kanda, D (Kanda, Dominique); Malombo, B  (Malombo, Billy); Darras, C (Darras, Christian); Schellevis, F (Schellevis, Francois)  Source: JOURNAL OF CLINICAL AND TRANSLATIONAL  ENDOCRINOLOGY Volume: 7 Pages: 33-  41 DOI: 10.1016/j.jcte.2016.12.005 Published: MAR 2017 | Not relevant to the main subject/ wrong population or geographical location/ duplicate or repetition |
|  | Design and patient characteristics of the randomized controlled trial TExT-MED  plus FANS A test of mHealth augmented social support added to a patient-focused text-  messaging intervention for emergency department patients with poorly controlled  diabetes  Author(s): Burner, E (Burner, Elizabeth); Mercado, J (Mercado, Janisse); Hernandez-  Saenz, A (Hernandez-Saenz, Antonio); Peters, A (Peters, Anne); Baezconde-Garbanati,  L (Baezconde-Garbanati, Lourdes); Arora, S (Arora, Sanjay); Wu, SY (Wu, Shinyi)  Source: CONTEMPORARY CLINICAL TRIALS Volume: 80 Pages: 1-  8 DOI: 10.1016/j.cct.2019.03.003 Published: MAY 2019 | Not relevant to the main subject/ wrong population or geographical location/ duplicate or repetition |
|  | Using Mobile Health to Improve Social Support for Low-Income Latino Patients  with Diabetes: A Mixed-Methods Analysis of the Feasibility Trial of TExT-MED plus  FANS  Author(s): Burner, E (Burner, Elizabeth); Lam, CN (Lam, Chun Nok); DeRoss, R  (DeRoss, Rebecca); Singer, MK (Singer, Marjorie Kagawa); Menchine, M (Menchine,  Michael); Arora, S (Arora, Sanjay)  Source: DIABETES TECHNOLOGY &  THERAPEUTICS Volume: 20 Issue: 1 Pages: 39-  48 DOI: 10.1089/dia.2017.0198 Early Access Date: DEC 2017 Published: JAN  2018 | Not relevant to the main subject/ wrong population or geographical location/ duplicate or repetition |
|  | A Pharmacist and Health Coach-Delivered Mobile Health Intervention for Type 2  Diabetes: Protocol for a Randomized Controlled Crossover Study  Author(s): Sharp, LK (Sharp, Lisa Kay); Biggers, A (Biggers, Alana); Perez, R (Perez,  Rosanne); Henkins, J (Henkins, Julia); Tilton, J (Tilton, Jessica); Gerber, B (Gerber, Ben  S.)  Source: JMIR RESEARCH PROTOCOLS Volume: 10 Issue: 3 Article  Number: e17170 DOI: 10.2196/17170 Published: MAR 2021 | Not relevant to the main subject/ wrong population or geographical location/ duplicate or repetition |
|  | Mobile phone messaging for preventive health care  Author(s): Vodopivec-Jamsek, V (Vodopivec-Jamsek, Vlasta); de Jongh, T (de Jongh,  Thyra); Gurol-Urganci, I (Gurol-Urganci, Ipek); Atun, R (Atun, Rifat); Car, J (Car,  Josip)  Source: COCHRANE DATABASE OF SYSTEMATIC REVIEWS Issue: 12 Article  Number: CD007457 DOI: 10.1002/14651858.CD007457.pub2 Published: 2012 | Not relevant to the main subject/ wrong population or geographical location/ duplicate or repetition |
|  | Diabetes Buddies Peer Support Through a Mobile Phone Buddy System  Author(s): Rotheram-Borus, MJ (Rotheram-Borus, Mary Jane); Tomlinson, M  (Tomlinson, Mark); Gwegwe, M (Gwegwe, Margaret); Comulada, WS (Comulada, W.  Scott); Kaufman, N (Kaufman, Neal); Keim, M (Keim, Marion)  Source: DIABETES EDUCATOR Volume: 38 Issue: 3 Pages: 357-  365 DOI: 10.1177/0145721712444617 Published: MAY-JUN 2012 | Not relevant to the main subject/ wrong population or geographical location/ duplicate or repetition |
|  | The Management of Diabetes in Everyday Life (MODEL) program: development  of a tailored text message intervention to improve diabetes self-care activities among  underserved African-American adults  Author(s): Gatwood, J (Gatwood, Justin); Shuvo, S (Shuvo, Sohul); Ross, A (Ross,  Alan); Riordan, C (Riordan, Carolyn); Smith, P (Smith, Patti); Gutierrez, ML (Gutierrez,  Mary Lou); Coday, M (Coday, Matilda); Bailey, J (Bailey, James)  Source: TRANSLATIONAL BEHAVIORAL  MEDICINE Volume: 10 Issue: 1 Special Issue: SI Pages: 204-  212 DOI: 10.1093/tbm/ibz024 Published: FEB 2020 | Not relevant to the main subject/ wrong population or geographical location/ duplicate or repetition |
|  | EatSmart, a Web-Based and Mobile Healthy Eating Intervention for  Disadvantaged People With Type 2 Diabetes: Protocol for a Pilot Mixed Methods  Intervention Study  Author(s): Karimi, N (Karimi, Nazgol); Crawford, D (Crawford, David); Opie, R  (Opie, Rachelle); Maddison, R (Maddison, Ralph); O'Connell, S (O'Connell, Stella);  Hamblin, PS (Hamblin, Peter Shane); Ng, AH (Ng, Ashley Huixian); Steele, C (Steele,  Cheryl); Rasmussen, B (Rasmussen, Bodil); Ball, K (Ball, Kylie)  Source: JMIR RESEARCH PROTOCOLS Volume: 9 Issue: 11 Article  Number: e19488 DOI: 10.2196/19488 Published: NOV 2020 | Not relevant to the main subject/ wrong population or geographical location/ duplicate or repetition |
|  | A systematic review of randomized controlled trials of mHealth interventions  against non-communicable diseases in developing countries  Author(s): Stephani, V (Stephani, Victor); Opoku, D (Opoku, Daniel); Quentin, W  (Quentin, Wilm)  Source: BMC PUBLIC HEALTH Volume: 16 Article  Number: 572 DOI: 10.1186/s12889-016-3226-3 Published: JUL 15 2016 | Not relevant to the main subject/ wrong population or geographical location/ duplicate or repetition |
|  | The effectiveness of e-& mHealth interventions to promote physical activity and  healthy diets in developing countries: A systematic review  Author(s): Muller, AM (Mueller, Andre Matthias); Alley, S (Alley, Stephanie);  Schoeppe, S (Schoeppe, Stephanie); Vandelanotte, C (Vandelanotte, Corneel)  Source: INTERNATIONAL JOURNAL OF BEHAVIORAL NUTRITION AND  PHYSICAL ACTIVITY Volume: 13 Article Number: 109 DOI: 10.1186/s12966-  016-0434-2 Published: OCT 10 2016 | Not relevant to the main subject/ wrong population or geographical location/ duplicate or repetition |
|  | Adapting the Diabetes Prevention Program for low and middle-income countries:  protocol for a cluster randomised trial to evaluate 'Lifestyle Africa'  Author(s): Catley, D (Catley, Delwyn); Puoane, T (Puoane, Thandi); Tsolekile, L  (Tsolekile, Lungiswa); Resnicow, K (Resnicow, Ken); Fleming, K (Fleming, Kandace);  Hurley, EA (Hurley, Emily A.); Smyth, JM (Smyth, Joshua M.); Vitolins, MZ (Vitolins,  Mara Z.); Lambert, EV (Lambert, Estelle V.); Levitt, N (Levitt, Naomi); Goggin, K  (Goggin, Kathy)  Source: BMJ OPEN Volume: 9 Issue: 11 Article  Number: e031400 DOI: 10.1136/bmjopen-2019-031400 Published: NOV 2019 | Not relevant to the main subject/ wrong population or geographical location/ duplicate or repetition |
|  | Efficacy of Mobile Health for Self-management of Cardiometabolic Risk Factors  A Theory-Guided Systematic Review  Author(s): Delva, S (Delva, Sabianca); Mendez, KJW (Mendez, Kyra J. Waligora);  Cajita, M (Cajita, Mia); Koirala, B (Koirala, Binu); Shan, RZ (Shan, Rongzi);  Wongvibulsin, S (Wongvibulsin, Shannon); Vilarino, V (Vilarino, Valerie); Gilmore,  DR (Gilmore, Danielle R.); Han, HR (Han, Hae-Ra)  Source: JOURNAL OF CARDIOVASCULAR  NURSING Volume: 36 Issue: 1 Pages: 34-  55 DOI: 10.1097/JCN.0000000000000659 Published: JAN-FEB 2021 | Not relevant to the main subject/ wrong population or geographical location/ duplicate or repetition |
|  | Adapting the Diabetes Prevention Program for low- and middle-income countries:  preliminary implementation findings from lifestyle Africa  Author(s): Catley, D (Catley, Delwyn); Puoane, T (Puoane, Thandi); Goggin, K  (Goggin, Kathy); Tsolekile, LP (Tsolekile, Lungiswa P.); Resnicow, K (Resnicow,  Ken); Fleming, K (Fleming, Kandace); Smyth, JM (Smyth, Joshua M.); Hurley, EA  (Hurley, Emily A.); Schlachter, S (Schlachter, Sarah); Vitolins, MZ (Vitolins, Mara Z.);  Lambert, EV (Lambert, Estelle, V); Hassen, M (Hassen, Mariam); Muhali, K (Muhali,  Kenneth); Schoor, R (Schoor, Rachel)  Source: TRANSLATIONAL BEHAVIORAL  MEDICINE Volume: 10 Issue: 1 Special Issue: SI Pages: 46-  54 DOI: 10.1093/tbm/ibz187 Published: FEB 2020 | Not relevant to the main subject/ wrong population or geographical location/ duplicate or repetition |
|  | One-way SMS and healthcare outcomes in Africa: Systematic review of  randomised trials with meta-analysis  Author(s): Linde, DS (Linde, Ditte S.); Korsholm, M (Korsholm, Malene); Katanga, J  (Katanga, Johnson); Rasch, V (Rasch, Vibeke); Lundh, A (Lundh, Andreas); Andersen,  MS (Andersen, Marianne S.)  Source: PLOS ONE Volume: 14 Issue: 6 Article  Number: e0217485 DOI: 10.1371/journal.pone.0217485 Published: JUN 6 2019 | Not relevant to the main subject/ wrong population or geographical location/ duplicate or repetition |
|  | Unmet Primary Care Needs in Diabetic Patients with Multimorbidity in a  Medically Underserved Area  Author(s): Jackson, BM (Jackson, Bianca M.); Gutierrez, ML (Gutierrez, Mary Lou);  Relyea, GE (Relyea, George E.); Carlton, EL (Carlton, Erik L.); Ahn, S (Ahn,  SangNam); Binkley, BL (Binkley, Bonnie L.); Bailey, JE (Bailey, James E.)  Source: HEALTH SERVICES RESEARCH AND MANAGERIAL  EPIDEMIOLOGY Volume: 4 Pages: 1-  9 DOI: 10.1177/2333392817702760 Published: MAY 11 2017 | Not relevant to the main subject/ wrong population or geographical location/ duplicate or repetition |
|  | Phone-based Intervention under Nurse Guidance after Stroke: Concept for  Lowering Blood Pressure after Stroke in Sub-Saharan Africa  Author(s): Ovbiagele, B (Ovbiagele, Bruce)  Source: JOURNAL OF STROKE & CEREBROVASCULAR  DISEASES Volume: 24 Issue: 1 Pages: 1-  9 DOI: 10.1016/j.jstrokecerebrovasdis.2014.08.011 Published: JAN 2015 | Not relevant to the main subject/ wrong population or geographical location/ duplicate or repetition |
|  | Rationale, design, and methodology for the optimizing outcomes in women with  gestational diabetes mellitus and their infants study  Author(s): Berry, DC (Berry, Diane C.); Neal, M (Neal, Madeline); Hall, EG (Hall,  Emily G.); Schwartz, TA (Schwartz, Todd A.); Verbiest, S (Verbiest, Sarah); Bonuck, K  (Bonuck, Karen); Goodnight, W (Goodnight, William); Brody, S (Brody, Seth);  Dorman, KF (Dorman, Karen F.); Menard, MK (Menard, Mary K.); Stuebe, AM  (Stuebe, Alison M.)  Source: BMC PREGNANCY AND CHILDBIRTH Volume: 13 Article  Number: 184 DOI: 10.1186/1471-2393-13-184 Published: OCT 10 2013 | Not relevant to the main subject/ wrong population or geographical location/ duplicate or repetition |
|  | Health and Wellness Technology Use by Historically Underserved Health  Consumers: Systematic Review  Author(s): Montague, E (Montague, Enid); Perchonok, J (Perchonok, Jennifer)  Source: JOURNAL OF MEDICAL INTERNET  RESEARCH Volume: 14 Issue: 3 Pages: 299-320 Article  Number: e78 DOI: 10.2196/jmir.2095 Published: MAY-JUN 2012 | Not relevant to the main subject/ wrong population or geographical location/ duplicate or repetition |
|  | Experience of cardiac implantable electronic device lead removal from a South  African tertiary referral centre  Author(s): Mkoko, P (Mkoko, Philasande); Mdakane, NX (Mdakane, Nicholus Xolani);  Govender, G (Govender, Glenda); Scherman, J (Scherman, Jacques); Chin, A (Chin,  Ashley)  Source: CARDIOVASCULAR JOURNAL OF  AFRICA Volume: 32 Issue: 4 Pages: 193-197 DOI: 10.5830/CVJA-2021-  010 Published: JUL-AUG 2021 | Not relevant to the main subject/ wrong population or geographical location/ duplicate or repetition |
|  | Study protocol for a randomized controlled trial to assess the feasibility of an  open label intervention to improve hydroxyurea adherence in youth with sickle cell  disease  Author(s): Smaldone, A (Smaldone, Arlene); Findley, S (Findley, Sally); Bakken, S  (Bakken, Suzanne); Matiz, LA (Matiz, L. Adriana); Rosenthal, SL (Rosenthal, Susan  L.); Jia, HM (Jia, Haomiao); Matos, S (Matos, Sergio); Manwani, D (Manwani, Deepa);  Green, NS (Green, Nancy S.)  Source: CONTEMPORARY CLINICAL TRIALS Volume: 49 Pages: 134-  142 DOI: 10.1016/j.cct.2016.06.004 Published: JUL 2016 | Not relevant to the main subject/ wrong population or geographical location/ duplicate or repetition |
|  | Targeting pregnancy-related weight gain to reduce disparities in obesity: Baseline  results from the Healthy Babies trial  Author(s): Herring, SJ (Herring, Sharon J.); Albert, JJ (Albert, Jessica J.); Darden, N  (Darden, Niesha); Bailer, B (Bailer, Brooke); Cruice, J (Cruice, Jane); Hassan, S  (Hassan, Sarmina); Bennett, GG (Bennett, Gary G.); Goetzl, L (Goetzl, Laura); Yu, DH  (Yu, Daohai); Kilby, LM (Kilby, Linda M.); Foster, GD (Foster, Gary D.)  Source: CONTEMPORARY CLINICAL TRIALS Volume: 87 Article  Number: 105822 DOI: 10.1016/j.cct.2019.105822 Published: DEC 2019 | Not relevant to the main subject/ wrong population or geographical location/ duplicate or repetition |
|  | Effect of a nurse-led lifestyle choice and coaching intervention on systolic blood  pressure among type 2 diabetic patients with a high atherosclerotic cardiovascular risk:  study protocol for a cluster-randomized trial  Author(s): Lumu, W (Lumu, William); Kibirige, D (Kibirige, Davis); Wesonga, R  (Wesonga, Ronald); Bahendeka, S (Bahendeka, Silver)  Source: TRIALS Volume: 22 Issue: 1 Article Number: 133 DOI: 10.1186/s13063-  021-05085-z Published: FEB 11 2021 | Not relevant to the main subject/ wrong population or geographical location/ duplicate or repetition |
|  | Interventions to Improve Medication Adherence in Ethnically Diverse Patients: A  Narrative Systematic Review  Author(s): Singh, P (Singh, Pavneet); LeBlanc, P (LeBlanc, Pamela); King-Shier, K  (King-Shier, Kathryn)  Source: JOURNAL OF TRANSCULTURAL  NURSING Volume: 32 Issue: 5 Pages: 600-  613 DOI: 10.1177/10436596211017971 Early Access Date: MAY  2021 Published: SEP 2021 | Not relevant to the main subject/ wrong population or geographical location/ duplicate or repetition |
|  | The Impact of Changing Antiseptic Skin Preparation Agent used for Cardiac  Implantable Electronic Device (CIED) Procedures on the Risk of Infection  Author(s): Qintar, M (Qintar, Mohammed); Zardkoohi, O (Zardkoohi, Omeed);  Hammadah, M (Hammadah, Muhammad); Hsu, A (Hsu, Amy); Wazni, O (Wazni,  Oussama); Wilkoff, BL (Wilkoff, Bruce L.); Tarakji, KG (Tarakji, Khaldoun G.)  Source: PACE-PACING AND CLINICAL  ELECTROPHYSIOLOGY Volume: 38 Issue: 2 Pages: 240-  246 DOI: 10.1111/pace.12514 Published: FEB 2015 | Not relevant to the main subject/ wrong population or geographical location/ duplicate or repetition |
|  | Tackling the growing diabetes burden in Sub-Saharan Africa: A framework for  enhancing outcomes in stroke patients  Author(s): Ovbiagele, B (Ovbiagele, Bruce)  Source: JOURNAL OF THE NEUROLOGICAL SCIENCES Volume: 348 Issue: 1-  2 Pages: 136-141 DOI: 10.1016/j.jns.2014.11.023 Published: JAN 15 2015 | Not relevant to the main subject/ wrong population or geographical location/ duplicate or repetition |
|  | The effectiveness of text messages support for diabetes self-management:  protocol of the TEXT4DSM study in the democratic Republic of Congo, Cambodia and  the Philippines  Author(s): van Olmen, J (van Olmen, Josefien); Ku, GM (Ku, Grace Marie); van Pelt,  M (van Pelt, Maurits); Kalobu, JC (Kalobu, Jean Clovis); Hen, H (Hen, Heang); Darras,  C (Darras, Christian); Van Acker, K (Van Acker, Kristien); Villaraza, B (Villaraza,  Balthazar); Schellevis, F (Schellevis, Francois); Kegels, G (Kegels, Guy)  Source: BMC PUBLIC HEALTH Volume: 13 Article  Number: 423 DOI: 10.1186/1471-2458-13-423 Published: MAY 1 2013 | Not relevant to the main subject/ wrong population or geographical location/ duplicate or repetition |
|  | mHealth Application Areas and Technology Combinations  Author(s): Abaza, H (Abaza, Haitham); Marschollek, M (Marschollek, Michael)  Source: METHODS OF INFORMATION IN MEDICINE Volume: 56 Pages: E105-  E122 DOI: 10.3414/ME17-05-0003 Published: 2017 | Not relevant to the main subject/ wrong population or geographical location/ duplicate or repetition |
|  | Improving Diabetes Management in Emerging Adulthood: An Intervention  Development Study Using the Multiphase Optimization Strategy  Author(s): Carcone, AI (Carcone, April Idalski); Ellis, DA (Ellis, Deborah A.); Eggly,  S (Eggly, Susan); MacDonell, KE (MacDonell, Karen E.); Ghosh, S (Ghosh, Samiran);  Buggs-Saxton, C (Buggs-Saxton, Colleen); Ondersma, SJ (Ondersma, Steven J.)  Source: JMIR RESEARCH PROTOCOLS Volume: 9 Issue: 10 Article  Number: e20191 DOI: 10.2196/20191 Published: OCT 2020 | Not relevant to the main subject/ wrong population or geographical location/ duplicate or repetition |
|  | Racial and ethnic healthcare disparities in patients undergoing laser lead  extraction  Author(s): Rodriguez, Y (Rodriguez, Yasser); Irizarry, F (Irizarry, Francisco); Carrillo,  RG (Carrillo, Roger G.)  Source: INTERNATIONAL JOURNAL OF  CARDIOLOGY Volume: 286 Pages: 181-  185 DOI: 10.1016/j.ijcard.2018.07.003 Published: JUL 1 2019 | Not relevant to the main subject/ wrong population or geographical location/ duplicate or repetition |
|  | Results of a Culturally Tailored Smartphone-Delivered Physical Activity  Intervention Among Midlife African American Women: Feasibility Trial  Author(s): Joseph, RP (Joseph, Rodney P.); Ainsworth, BE (Ainsworth, Barbara E.);  Hollingshead, K (Hollingshead, Kevin); Todd, M (Todd, Michael); Keller, C (Keller,  Colleen)  Source: JMIR MHEALTH AND UHEALTH Volume: 9 Issue: 4 Article  Number: e27383 DOI: 10.2196/27383 Published: APR 22 2021 | Not relevant to the main subject/ wrong population or geographical location/ duplicate or repetition |
|  | m-Diabete in Senegal. ''Be He@lthy Be Mobile'', a program initiated by WHO  and the ITU  Author(s): Eskandar, H (Eskandar, H.); Pujari, S (Pujari, S.); Dia, IK (Dia, I-K);  Kleinebreil, L (Kleinebreil, L.); Meagher, S (Meagher, S.)  Source: MEDECINE ET SANTE TROPICALES Volume: 27 Issue: 4 Pages: 364-  369 DOI: 10.1684/mst.2017.0729 Published: OCT-DEC 2017 | Not relevant to the main subject/ wrong population or geographical location/ duplicate or repetition |
|  | Mobile phone access and comfort: implications for HIV and tuberculosis care in  India and South Africa  Author(s): Cox, SN (Cox, S. N.); Elf, JL (Elf, J. L.); Lokhande, R (Lokhande, R.);  Ogale, YP (Ogale, Y. P.); DiAndreth, L (DiAndreth, L.); Dupuis, E (Dupuis, E.);  Milovanovic, M (Milovanovic, M.); Mpungose, N (Mpungose, N.); Mave, V (Mave,  V.); Suryavanshi, N (Suryavanshi, N.); Gupta, A (Gupta, A.); Martinson, N (Martinson,  N.); Golub, JE (Golub, J. E.); Mathad, JS (Mathad, J. S.)  Source: INTERNATIONAL JOURNAL OF TUBERCULOSIS AND LUNG  DISEASE Volume: 23 Issue: 7 Pages: 865-  872 DOI: 10.5588/ijtld.18.0542 Published: JUL 1 2019 | Not relevant to the main subject/ wrong population or geographical location/ duplicate or repetition |
|  | Diabetes Applications for Arabic Speakers: A Critical Review of Available Apps  for Android and iOS operated Smartphones  Author(s): Alhuwail, D (Alhuwail, Dari)  Edited by: Sermeus W; Procter PM; Weber P  Source: NURSING INFORMATICS 2016: EHEALTH FOR ALL: EVERY LEVEL  COLLABORATION - FROM PROJECT TO REALIZATION Book Series: Studies in  Health Technology and Informatics Volume: 225 Pages: 587-591 DOI: 10.3233/978-  1-61499-658-3-587 Published: 2016 | Not relevant to the main subject/ wrong population or geographical location/ duplicate or repetition |
|  | Design Principles for mHealth Application Development in Rural Parts of  Developing Countries: The Case of Noncommunicable Diseases in Kenya  Author(s): Viljoen, A (Viljoen, Altus); Klinker, K (Klinker, Kai); Wiesche, M  (Wiesche, Manuel); Uebernickel, F (Uebernickel, Falk); Krcmar, H (Krcmar, Helmut)  Source: IEEE TRANSACTIONS ON ENGINEERING  MANAGEMENT DOI: 10.1109/TEM.2021.3072601 Early Access Date: JUN 2021 | Not relevant to the main subject/ wrong population or geographical location/ duplicate or repetition |
|  | SMS Education for the Promotion of Diabetes Self-Management in Low &  Middle Income Countries: A Randomized Controlled Trial in Egypt  Author(s): Abaza, H (Abaza, Haitham); Marschollek, M (Marschollek, Michael);  Schulze, M (Schulze, Mareike)  Edited by: Gundlapalli AV; Jaulent MC; Zhao D  Source: MEDINFO 2017: PRECISION HEALTHCARE THROUGH  INFORMATICS Book Series: Studies in Health Technology and  Informatics Volume: 245 Pages: 1209-1209 DOI: 10.3233/978-1-61499-830-3-  1209 Published: 2017 | Not relevant to the main subject/ wrong population or geographical location/ duplicate or repetition |
|  | Implementing Innovative Approaches to Healthcare in a Lower-Middle Income  Country: Perspectives from Malawi  Author(s): Larsson, E (Larsson, Emma); Mawkin, M (Mawkin, Mala); Taylor-  Robinson, SD (Taylor-Robinson, Simon D.); Harrington, P (Harrington, Peter);  Gondwe, H (Gondwe, Hastings); Watson, C (Watson, Chris); Gallagher, J (Gallagher,  Joseph); Ledwidge, M (Ledwidge, Mark); Chirambo, GB (Chirambo, Griphin Baxter);  O'Donoghue, J (O'Donoghue, John)  Source: INTERNATIONAL JOURNAL OF GENERAL  MEDICINE Volume: 13 Pages: 1723-  1730 DOI: 10.2147/IJGM.S285130 Published: 2020 | Not relevant to the main subject/ wrong population or geographical location/ duplicate or repetition |
|  | Assessment of Rwandan diabetic patients' needs and expectations to develop their  first diabetes self-management smartphone application (Kir'App)  Author(s): Kabeza, CB (Kabeza, Claudine B.); Harst, L (Harst, Lorenz); Schwarz, PEH  (Schwarz, Peter E. H.); Timpel, P (Timpel, Patrick)  Source: THERAPEUTIC ADVANCES IN ENDOCRINOLOGY AND  METABOLISM Volume: 10 Article  Number: 2042018819845318 DOI: 10.1177/2042018819845318 Published: APR  2019 | Not relevant to the main subject/ wrong population or geographical location/ duplicate or repetition |
|  | Telehealth Program for Type 2 Diabetes: Usability, Satisfaction, and Clinical  Usefulness in an Urban Community Health Center  Author(s): Welch, G (Welch, Garry); Balder, A (Balder, Andrew); Zagarins, S  (Zagarins, Sofija)  Source: TELEMEDICINE AND E-HEALTH Volume: 21 Issue: 5 Pages: 395-  403 DOI: 10.1089/tmj.2014.0069 Published: MAY 1 2015 | Not relevant to the main subject/ wrong population or geographical location/ duplicate or repetition |
|  | Clinical relevance of smartphone apps for diabetes management: A global  overview  Author(s): Huang, ZL (Huang, Zhilian); Soljak, M (Soljak, Michael); Boehm, BO  (Boehm, Bernhard Otto); Car, J (Car, Josip)  Source: DIABETES-METABOLISM RESEARCH AND  REVIEWS Volume: 34 Issue: 4 Article  Number: e2990 DOI: 10.1002/dmrr.2990 Published: MAY 2018 | Not relevant to the main subject/ wrong population or geographical location/ duplicate or repetition |
|  | Assessment of Rwandan diabetic patients' needs and expectations to develop their  first diabetes self-management smartphone application (Kir'App) (vol 10, pg 1, 2019)  Author(s): Kabeza, CB (Kabeza, C. B.); Harst, L (Harst, L.); Schwarz, PEH (Schwarz,  P. E. H.)  Source: THERAPEUTIC ADVANCES IN ENDOCRINOLOGY AND  METABOLISM Volume: 11 Article  Number: 2042018820973813 DOI: 10.1177/2042018820973813 Published: DEC  2020 | Not relevant to the main subject/ wrong population or geographical location/ duplicate or repetition |
|  | Mobile Health and Technology Usage by Patients in the Diabetes, Nutrition, and  Weight Management Clinic at an Urban Academic Medical Center  Author(s): Stockman, MC (Stockman, Mary-Catherine); Modzelewski, K  (Modzelewski, Katherine); Steenkamp, D (Steenkamp, Devin)Source: DIABETES TECHNOLOGY &  THERAPEUTICS Volume: 21 Issue: 7 Pages: 400-  405 DOI: 10.1089/dia.2018.0369 Early Access Date: MAY 2019 Published: JUL 1  2019 | Not relevant to the main subject/ wrong population or geographical location/ duplicate or repetition |
|  | Rams Have Heart, a Mobile App Tracking Activity and Fruit and Vegetable  Consumption to Support the Cardiovascular Health of College Students: Development  and Usability Study  Author(s): Krzyzanowski, MC (Krzyzanowski, Michelle C.); Kizakevich, PN  (Kizakevich, Paul N.); Duren-Winfield, V (Duren-Winfield, Vanessa); Eckhoff, R  (Eckhoff, Randall); Hampton, J (Hampton, Joel); Carr, LTB (Carr, Loneke T.  Blackman); McCauley, G (McCauley, Georgia); Roberson, KB (Roberson, Kristina B.);  Onsomu, EO (Onsomu, Elijah O.); Williams, J (Williams, John); Price, AA (Price,  Amanda Alise)  Source: JMIR MHEALTH AND UHEALTH Volume: 8 Issue: 8 Article  Number: e15156 DOI: 10.2196/15156 Published: AUG 5 2020 | Not relevant to the main subject/ wrong population or geographical location/ duplicate or repetition |
|  | Examining the Success Factors for Mobile Applications for Self-Management of  Diabetic Treatment in a South African Context  Author(s): Mainoti, GF (Mainoti, Ganizani Fidelis); Isabirye, N (Isabirye, Naomi)  Edited by: Ouma C  Source: 2018 OPEN INNOVATIONS CONFERENCE (OI) Pages: 198-  202 Published: 2018 | Not relevant to the main subject/ wrong population or geographical location/ duplicate or repetition |
|  | Community- and mHealth-based integrated management of diabetes in primary  healthcare in Rwanda (D(2)Rwanda): the protocol of a mixed-methods study including a  cluster randomised controlled trial  Author(s): Lygidakis, C (Lygidakis, Charilaos); Uwizihiwe, JP (Uwizihiwe, Jean Paul);  Kallestrup, P (Kallestrup, Per); Bia, M (Bia, Michela); Condo, J (Condo, Jeanine);  Vogele, C (Vogele, Claus)  Source: BMJ OPEN Volume: 9 Issue: 7 Article  Number: e028427 DOI: 10.1136/bmjopen-2018-028427 Published: AUG 2019 | Not relevant to the main subject/ wrong population or geographical location/ duplicate or repetition |
|  | Usability of Commercially Available Mobile Applications for Diverse Patients  Author(s): Sarkar, U (Sarkar, Urmimala); Gourley, GI (Gourley, Gato I.); Lyles, CR  (Lyles, Courtney R.); Tieu, L (Tieu, Lina); Clarity, C (Clarity, Cassidy); Newmark, L  (Newmark, Lisa); Singh, K (Singh, Karandeep); Bates, DW (Bates, David W.)  Source: JOURNAL OF GENERAL INTERNAL  MEDICINE Volume: 31 Issue: 12 Pages: 1417-1426 DOI: 10.1007/s11606-016-  3771-6 Published: DEC 2016 | Not relevant to the main subject/ wrong population or geographical location/ duplicate or repetition |
|  | Community-Driven Priorities in Smartphone Application Development:  Leveraging Social Networks to Self-Manage Type 2 Diabetes in a Low-Income African  American Neighborhood  Author(s): Surkan, PJ (Surkan, Pamela J.); Mezzanotte, KS (Mezzanotte, Kathryne S.);  Sena, LM (Sena, Laura M.); Chang, LW (Chang, Larry W.); Gittelsohn, J (Gittelsohn,  Joel); Lagerros, YT (Lagerros, Ylva Trolle); Quinn, CC (Quinn, Charlene C.); Zachary,  WW (Zachary, Wayne W.)  Source: INTERNATIONAL JOURNAL OF ENVIRONMENTAL RESEARCH AND  PUBLIC HEALTH Volume: 16 Issue: 15 Article  Number: 2715 DOI: 10.3390/ijerph16152715 Published: AUG 1 2019 | Not relevant to the main subject/ wrong population or geographical location/ duplicate or repetition |
|  | Critical appraisal of a mHealth-assisted community-based cardiovascular disease  risk screening program in rural Kenya: an operational research studyAuthor(s): Aw, M (Aw, Michael); Ochieng, BO (Ochieng, Benard Omondi); Attambo,  D (Attambo, Daniel); Opot, D (Opot, Danet); Aw, J (Aw, James); Francis, S (Francis,  Stacy); Hawkes, MT (Hawkes, Michael T.)  Source: PATHOGENS AND GLOBAL HEALTH Volume: 114 Issue: 7 Pages: 379-  387 DOI: 10.1080/20477724.2020.1816286 Early Access Date: SEP  2020 Published: OCT 2 2020 | Not relevant to the main subject/ wrong population or geographical location/ duplicate or repetition |
|  | Centering TechQuity through Biomedical Informatics Centers at Minority-  Serving Academic Institutions Providing Informatics Solutions for Urban Safety-Net  Settings  Author(s): Ogunyemi, OI (Ogunyemi, Omolola I.); George, S (George, Sheba);  Mukherjee, S (Mukherjee, Sukrit); Gandhi, M (Gandhi, Meghal); Jenders, RA (Jenders,  Robert A.)  Source: JOURNAL OF HEALTH CARE FOR THE POOR AND  UNDERSERVED Volume: 32 Issue: 2 Pages: 278-  289 DOI: 10.1353/hpu.2021.0063 Supplement: S Published: MAY 2021 | Not relevant to the main subject/ wrong population or geographical location/ duplicate or repetition |
|  | Reutilization and adaptation of a mobile architecture for Diabetes self-  management  Author(s): Bessin, ITI (Bessin, Ivan Teddy I.); Guinko, F (Guinko, Ferdinand); Ben  Sta, H (Ben Sta, Hatem)  Book Group Author(s): IEEE  Source: 2018 INTERNATIONAL CONFERENCE ON SMART APPLICATIONS,  COMMUNICATIONS AND NETWORKING (SMARTNETS) Published: 2018 | Not relevant to the main subject/ wrong population or geographical location/ duplicate or repetition |
|  | Cost and Efficiency of a Hybrid Mobile Multidisease Testing Approach With  High HIV Testing Coverage in East Africa  Author(s): Chang, W (Chang, Wei); Chamie, G (Chamie, Gabriel); Mwai, D (Mwai,  Daniel); Clark, TD (Clark, Tamara D.); Thirumurthy, H (Thirumurthy, Harsha);  Charlebois, ED (Charlebois, Edwin D.); Petersen, M (Petersen, Maya); Kabami, J  (Kabami, Jane); Ssemmondo, E (Ssemmondo, Emmanuel); Kadede, K (Kadede, Kevin);  Kwarisiima, D (Kwarisiima, Dalsone); Sang, N (Sang, Norton); Bukusi, EA (Bukusi,  Elizabeth A.); Cohen, CR (Cohen, Craig R.); Kamya, M (Kamya, Moses); Havlir, DV  (Havlir, Diane V.); Kahn, JG (Kahn, James G.)  Source: JAIDS-JOURNAL OF ACQUIRED IMMUNE DEFICIENCY  SYNDROMES Volume: 73 Issue: 3 Pages: E39-E45 Published: NOV 1 2016 | Not relevant to the main subject/ wrong population or geographical location/ duplicate or repetition |
|  | Pregnant women's experiences with an integrated diagnostic and decision support  device for antenatal care in Ghana  Author(s): Abejirinde, IOO (Abejirinde, Ibukun-Oluwa Omolade); Douwes, R  (Douwes, Renate); Bardaji, A (Bardaji, Azucena); Abugnaba-Abanga, R (Abugnaba-  Abanga, Rudolf); Zweekhorst, M (Zweekhorst, Marjolein); van Roosmalen, J (van  Roosmalen, Jos); De Brouwere, V (De Brouwere, Vincent)  Source: BMC PREGNANCY AND CHILDBIRTH Volume: 18 Article  Number: 209 DOI: 10.1186/s12884-018-1853-7 Published: JUN 5 2018 | Not relevant to the main subject/ wrong population or geographical location/ duplicate or repetition |
|  | Designing an integrated, nurse-driven and home-based digital intervention to  improve insulin management in under-resourced settings  Author(s): Piotie, PN (Piotie, Patrick Ngassa); Wood, P (Wood, Paola); Webb, EM  (Webb, Elizabeth M.); Hugo, JFM (Hugo, Johannes F. M.); Rheeder, P (Rheeder, Paul)  Source: THERAPEUTIC ADVANCES IN ENDOCRINOLOGY AND  METABOLISM Volume: 12 Article  Number: 20420188211054688 DOI: 10.1177/20420188211054688 Published: OCT  2021 | Not relevant to the main subject/ wrong population or geographical location/ duplicate or repetition |
|  | A qualitative study of users' experiences after 3 months: the first Rwandan  diabetes self-management Smartphone application "Kir'App"  Author(s): Kabeza, CB (Kabeza, Claudine B.); Harst, L (Harst, Lorenz); Schwarz, PEH  (Schwarz, Peter E. H.); Timpel, P (Timpel, Patrick)  Source: THERAPEUTIC ADVANCES IN ENDOCRINOLOGY AND  METABOLISM Volume: 11 Article  Number: 2042018820914510 DOI: 10.1177/2042018820914510 Published: APR  2020 | Not relevant to the main subject/ wrong population or geographical location/ duplicate or repetition |
|  | Bibliometric analysis of worldwide scientific literature in mobile - health: 2006-  2016  Author(s): Sweileh, WM (Sweileh, Waleed M.); Al-Jabi, SW (Al-Jabi, Samah W.);  AbuTaha, AS (AbuTaha, Adham S.); Zyoud, SH (Zyoud, Sa'ed H.); Anayah, FMA  (Anayah, Fathi M. A.); Sawalha, AF (Sawalha, Ansam F.)  Source: BMC MEDICAL INFORMATICS AND DECISION  MAKING Volume: 17 Article Number: 72 DOI: 10.1186/s12911-017-0476-  7 Published: MAY 30 2017 | Not relevant to the main subject/ wrong population or geographical location/ duplicate or repetition |
|  | Diabetes Connect Developing a Mobile Health Intervention to Link Diabetes  Community Health Workers With Primary Care  Author(s): Cherrington, AL (Cherrington, Andrea L.); Agne, AA (Agne, April A.);  Lampkin, Y (Lampkin, Yolanda); Birl, A (Birl, Annie); Shelton, TC (Shelton, Tanya  C.); Guzman, A (Guzman, Alfredo); Willig, JH (Willig, James H.)  Source: JOURNAL OF AMBULATORY CARE  MANAGEMENT Volume: 38 Issue: 4 Pages: 333-  345 DOI: 10.1097/JAC.0000000000000110 Published: OCT-DEC 2015  Abstract: Community health worker (CHW) interventions can help improv | Not relevant to the main subject/ wrong population or geographical location/ duplicate or repetition |
|  | Social Support for Diabetes Self-Management via eHealth Interventions  Author(s): Vorderstrasse, A (Vorderstrasse, Allison); Lewinski, A (Lewinski, Allison);  Melkus, GD (Melkus, Gail D'Eramo); Johnson, C (Johnson, Constance)  Source: CURRENT DIABETES REPORTS Volume: 16 Issue: 7 Article  Number: 56 DOI: 10.1007/s11892-016-0756-0 Published: JUL 2016 | Not relevant to the main subject/ wrong population or geographical location/ duplicate or repetition |
|  | Epic Allies, a Gamified Mobile Phone App to Improve Engagement in Care,  Antiretroviral Uptake, and Adherence Among Young Men Who Have Sex With Men  and Young Transgender Women Who Have Sex With Men: Protocol for a Randomized  Controlled Trial  Author(s): LeGrand, S (LeGrand, Sara); Muessig, KE (Muessig, Kathryn E.); Platt, A  (Platt, Alyssa); Soni, K (Soni, Karina); Egger, JR (Egger, Joseph R.); Nwoko, N  (Nwoko, Nkechinyere); McNulty, T (McNulty, Tobias); Hightow-Weidman, LB  (Hightow-Weidman, Lisa B.)  Source: JMIR RESEARCH PROTOCOLS Volume: 7 Issue: 4 Article  Number: e94 DOI: 10.2196/resprot.8811 Published: APR 2018 | Not relevant to the main subject/ wrong population or geographical location/ duplicate or repetition |
|  | Implementation and impact of mobile health (mHealth) in the management of  diabetes mellitus in Africa: a systematic review protocol  Author(s): Dike, FO (Dike, Franklin Okechukwu); Mutabazi, JC (Mutabazi, Jean  Claude); Ubani, BC (Ubani, Blessing Chinenye); Isa, AS (Isa, Ahmed Sherif); Ezeude,  C (Ezeude, Chidiebele); Musa, E (Musa, Ezekiel); Iheonye, H (Iheonye, Henry); Ainavi,  II (Ainavi, Isah Idris)  Source: BMJ OPEN Volume: 11 Issue: 12 Article  Number: e047556 DOI: 10.1136/bmjopen-2020-047556 Published: DEC 2021 | Not relevant to the main subject/ wrong population or geographical location/ duplicate or repetition |
|  | Prevalence and Severity of Diabetic Retinopathy in Northwest Cameroon as  Identified by Teleophthalmology  Author(s): Jivraj, I (Jivraj, Imran); Ng, M (Ng, Mancho); Rudnisky, CJ (Rudnisky,  Chris J.); Dimla, B (Dimla, Beri); Tambe, E (Tambe, Emmanuel); Nathoo, N (Nathoo,  Nawaaz); Tennant, MTS (Tennant, Matthew T. S.)  Source: TELEMEDICINE AND E-HEALTH Volume: 17 Issue: 4 Pages: 294-  298 DOI: 10.1089/tmj.2010.0155 Published: MAY 2011 | Not relevant to the main subject/ wrong population or geographical location/ duplicate or repetition |
|  | Implementing Modular Interactive Tiles for Rehabilitation in Tanzania - a Pilot  Study  Author(s): Lund, HH (Lund, Henrik Hautop); Jensen, LSD (Jensen, Line S. D.);  Ssessanga, Y (Ssessanga, Yusuf); Abdalahman, R (Abdalahman, Rashid)  Book Group Author(s): IEEE  Source: 2014 IST-AFRICA CONFERENCE PROCEEDINGS Published: 2014 | Not relevant to the main subject/ wrong population or geographical location/ duplicate or repetition |
|  | Post-diagnosis Management of Diabetes through a Mobile Health Consultation  Application  Author(s): Gittens, M (Gittens, Mechelle); King, R (King, Reco); Gittens, C (Gittens,  Curtis); Als, A (Als, Adrian)  Book Group Author(s): IEEE  Source: 2014 IEEE 16TH INTERNATIONAL CONFERENCE ON E-HEALTH  NETWORKING, APPLICATIONS AND SERVICES (HEALTHCOM) Pages: 152-  157 Published: 2014 | Not relevant to the main subject/ wrong population or geographical location/ duplicate or repetition |
|  | Towards a Telehomecare in Algeria: Case of Diabetes Measurement and Remote  Monitoring  Author(s): Zarour, K (Zarour, Karim)  Source: INTERNATIONAL JOURNAL OF E-HEALTH AND MEDICAL  COMMUNICATIONS Volume: 8 Issue: 4 Pages: 61-  80 DOI: 10.4018/IJEHMC.2017100104 Published: 2017 | Not relevant to the main subject/ wrong population or geographical location/ duplicate or repetition |
|  | Practical telehealth to improve control and engagement for patients with clinic-  refractory diabetes mellitus (PRACTICE-DM): Protocol and baseline data for a  randomized trial  Author(s): Kobe, EA (Kobe, Elizabeth A.); Edelman, D (Edelman, David); Tarkington,  PE (Tarkington, Phillip E.); Bosworth, HB (Bosworth, Hayden B.); Maciejewski, ML  (Maciejewski, Matthew L.); Steinhauser, K (Steinhauser, Karen); Jeffreys, AS (Jeffreys,  Amy S.); Coffman, CJ (Coffman, Cynthia J.); Smith, VA (Smith, Valerie A.);  Strawbridge, EM (Strawbridge, Elizabeth M.); Szabo, ST (Szabo, Steven T.); Desai, S  (Desai, Shivan); Garrett, MP (Garrett, Mary P.); Wilmot, TC (Wilmot, Theresa C.);  Marcano, TJ (Marcano, Teresa J.); Overby, DL (Overby, Donna L.); Tisdale, GA  (Tisdale, Glenda A.); Durkee, M (Durkee, Melissa); Bullard, S (Bullard, Susan); Dar,  MS (Dar, Moahad S.); Mundy, AC (Mundy, Amy C.); Hiner, J (Hiner, Janette);  Fredrickson, SK (Fredrickson, Sonja K.); Elliott, NTM (Elliott, Nadya T. Majette);  Howard, T (Howard, Teresa); Jeter, DH (Jeter, Deborah H.); Danus, S (Danus,  Susanne); Crowley, MJ (Crowley, Matthew J.)  Source: CONTEMPORARY CLINICAL TRIALS Volume: 98 Article  Number: 106157 DOI: 10.1016/j.cct.2020.106157 Published: NOV 2020 | Not relevant to the main subject/ wrong population or geographical location/ duplicate or repetition |
|  | Interventions to Improve Management of Chronic Conditions Among Racial and  Ethnic Minorities  Author(s): Doshi, R (Doshi, Riddhi); Aseltine, RH (Aseltine, Robert H.); Sabina, AB  (Sabina, Alyse B.); Graham, GN (Graham, Garth N.)  Source: JOURNAL OF RACIAL AND ETHNIC HEALTH  DISPARITIES Volume: 4 Issue: 6 Pages: 1033-1041 DOI: 10.1007/s40615-017-  0431-4 Published: DEC 2017 | Not relevant to the main subject/ wrong population or geographical location/ duplicate or repetition |
|  | Development and Evaluation of a Tailored Mobile Health Intervention to Improve  Medication Adherence in Black Patients With Uncontrolled Hypertension and Type 2  Diabetes: Pilot Randomized Feasibility Trial  Author(s): Schoenthaler, A (Schoenthaler, Antoinette); Leon, M (Leon, Michelle);  Butler, M (Butler, Mark); Steinhaeuser, K (Steinhaeuser, Karsten); Wardzinski, W  (Wardzinski, William)  Source: JMIR MHEALTH AND UHEALTH Volume: 8 Issue: 9 Article  Number: e17135 DOI: 10.2196/17135 Published: SEP 23 2020 | Not relevant to the main subject/ wrong population or geographical location/ duplicate or repetition |
|  | A Mobile Application for Health Information Dissemination: a Namibian Context  Author(s): Angula, N (Angula, Nikodemus); Dlodlo, N (Dlodlo, Nomusa)  Edited by: Kumar V; Singh UG; Sudarsan SD  Source: 2016 THIRD INTERNATIONAL CONFERENCE ON ADVANCES IN  COMPUTING, COMMUNICATION AND ENGINEERING (ICACCE  2016) Pages: 461-466 Published: 2016 | Not relevant to the main subject/ wrong population or geographical location/ duplicate or repetition |
|  | Evaluation of the care for diabetes patients by general practitioners in the province  of Khouribga, Morocco  Author(s): Hassoune, S (Hassoune, S.); Badri, S (Badri, S.); Nani, S (Nani, S.);  Belhadi, L (Belhadi, L.); Maaroufi, A (Maaroufi, A.)  Source: EASTERN MEDITERRANEAN HEALTH  JOURNAL Volume: 19 Issue: 1 Pages: 52-  58 DOI: 10.26719/2013.19.1.52 Published: JAN 2013 | Not relevant to the main subject/ wrong population or geographical location/ duplicate or repetition |
|  | Development of a Mobile Phone Based Ophthalmoscope for Telemedicine  Author(s): Blanckenberg, M (Blanckenberg, Mike); Worst, C (Worst, Christo);  Scheffer, C (Scheffer, Cornie)Book Group Author(s): IEEE  Source: 2011 ANNUAL INTERNATIONAL CONFERENCE OF THE IEEE  ENGINEERING IN MEDICINE AND BIOLOGY SOCIETY (EMBC) Book  Series: IEEE Engineering in Medicine and Biology Society Conference  Proceedings Pages: 5236-5239 Published: 2011 | Not relevant to the main subject/ wrong population or geographical location/ duplicate or repetition |
|  | Factors influencing the effective management of diabetes during humanitarian  crises in low- and middle-income countries: a systematic review  Author(s): Song, K (Song, K.); Lee, A (Lee, A.)  Source: PUBLIC HEALTH Volume: 199 Pages: 110-  117 DOI: 10.1016/j.puhe.2021.08.020 Published: OCT 2021 | Not relevant to the main subject/ wrong population or geographical location/ duplicate or repetition |
|  | Telehealth Diabetes Prevention Intervention for the Next Generation of African  American Youth: Protocol for a Pilot Trial  Author(s): Gamble, A (Gamble, Abigail); Beech, B (Beech, Bettina); Wade, B (Wade,  Breanna); Sutton, V (Sutton, Victor); Lim, C (Lim, Crystal); Sandridge, S (Sandridge,  Shanda); Welsch, M (Welsch, Michael)  Source: JMIR RESEARCH PROTOCOLS Volume: 10 Issue: 3 Article  Number: e25699 DOI: 10.2196/25699 Published: MAR 2021 | Not relevant to the main subject/ wrong population or geographical location/ duplicate or repetition |
|  | Design of a novel digital intervention to promote healthy weight management  among postpartum African American women  Author(s): Evans, WD (Evans, W. D.); Harrington, C (Harrington, C.); Patchen, L  (Patchen, L.); Andrews, V (Andrews, V); Gaminian, A (Gaminian, A.); Ellis, LP (Ellis,  L. P.); Napolitano, MA (Napolitano, M. A.)  Source: CONTEMPORARY CLINICAL TRIALS  COMMUNICATIONS Volume: 16 Article  Number: 100460 DOI: 10.1016/j.conctc.2019.100460 Published: DEC 2019 | Not relevant to the main subject/ wrong population or geographical location/ duplicate or repetition |
|  | Pilot Mobile Phone Intervention in Promoting Type 2 Diabetes Management in an  Urban Area in Ghana: A Randomized Controlled Trial  Author(s): Asante, E (Asante, Ernest); Bam, V (Bam, Victoria); Diji, AKA (Diji,  Abigail Kusi-Amponsah); Lomotey, AY (Lomotey, Alberta Yemotsoo); Boateng, AO  (Owusu Boateng, Agnes); Sarfo-Kantanka, O (Sarfo-Kantanka, Osei); Ansah, EO  (Oparebea Ansah, Eunice); Adjei, D (Adjei, Dennis)  Source: DIABETES EDUCATOR Volume: 46 Issue: 5 Pages: 455-  464 DOI: 10.1177/0145721720954070 Published: OCT 2020 | Not relevant to the main subject/ wrong population or geographical location/ duplicate or repetition |
|  | Evaluating the primary care clinical pharmacist visit transition to telehealth during  the COVID-19 pandemic by comparing medication related problems from telehealth  visits and in-person visits  Author(s): McNamara, A (McNamara, Anusha); Zhao, M (Zhao, Milly); Lee, SY (Lee,  Shin-Yu)  Source: JOURNAL OF THE AMERICAN COLLEGE OF CLINICAL  PHARMACY Volume: 4 Issue: 8 Special Issue: SI Pages: 914-  923 DOI: 10.1002/jac5.1487 Early Access Date: JUN 2021 Published: AUG 2021 | Not relevant to the main subject/ wrong population or geographical location/ duplicate or repetition |
[truncated: 318,753 more chars]
