# Supplementary material for: Implementation and impact of mhealth in the management of diabetes mellitus in Africa: A systematic review and meta-analysis
Source: PLOS Digit Health. 2025 Apr 8;4(4):e0000776. doi: 10.1371/journal.pdig.0000776 (PMC11978007; doi:10.1371/journal.pdig.0000776)
Supplement: S2 Table — Table of selected full text articles after abstract and title screening. (DOCX) [file pdig.0000776.s002.docx]

| S/N | Full Articles | Reason for exclusion/Included |
| --- | --- | --- |
|  | The use of text messaging for improving adherence to anti-diabetic regimen  and glycaemic control in low-resource settings of South Africa: A study  protocol for a randomised controlled trial  Eyitayo Omolara Owolabi*, Daniel Ter Goon  Department of Nursing Science, Faculty of Health Sciences, University of Fort Hare, East London, South Africa | Not RCT / Different outcomes targeted/ Outcome evaluation method different/ Repetition |
|  | SMS-based intervention in type 2  diabetes: clinical trial in Senegal  Matthieu Wargny,1,2 Line Kleinebreil,3 Said Norou Diop,4 Maïmouna  Ndour-Mbaye,4 Mady Ba,5 Beverley Balkau,6,7 Dominique Simon8 | INCLUDED |
|  | SMS education for the promotion of  diabetes self-management in low & middle  income countries: a pilot randomized  controlled trial in Egypt  Haitham Abaza* and Michael Marschollek | INCLUDED |
|  | SMS education for the promotion of  diabetes self-management in low & middle  income countries: a pilot randomized  controlled trial in Egypt  Haitham Abaza* and Michael Marschollek | Repetition |
|  | Process evaluation of a brief messaging  intervention to improve diabetes treatment  adherence in sub-Saharan Africa  N. Leon1,2* N. Levitt4 and A. Farmer7  , H. Namadingo3, S. Cooper1, K. Bobrow4, C. Mwantisi3, M. Nyasulu3, N. Sicwebu5, A. Crampin3,6  , | Not RCT / Different outcomes targeted/ Outcome evaluation method different/ Repetition |
|  | Pilot Mobile Phone Intervention  in Promoting Type 2  Diabetes Management in an  Urban Area in Ghana: A  Randomized Controlled Trial  Ernest Asante, BSc, MSc, RGN  Dennis Adjei, BSc, RGN  Victoria Bam, PhD, SRN  Abigail Kusi-Amponsah Diji, BSc, MSc, RGN, FGCNM  Alberta Yemotsoo Lomotey, BSc, MPH, SRN  Agnes Owusu Boateng, BSc, MSc, RGN  Osei Sarfo-Kantanka, BSc, MBCHB, MWACP, MGCP, FWACP  Eunice Oparebea Ansah, BSc, MBCHB, MWACP, MGCP, PG Dip | INCLUDED |
|  | Mobile phone ownership and willingness to receive  mHealth services among patients with diabetes  mellitus in South-West, Nigeria  Michael Adeyemi Olamoyegun, Taiwo Hassan Raimi, Oluwabukola Ayodele Ala, Joseph Olusesan Fadare | Not RCT / Different outcomes targeted/ Outcome evaluation method different/ Repetition |
|  | Mobile phone ownership among Nigerians with diabetes  *Okoro EO1, Sholagberu HO1, Kolo PM2 | Not RCT / Different outcomes targeted/ Outcome evaluation method different/ Repetition |
|  | Mobile Monitoring of Diabetic Patients in  the Active File of the Medical Center of Do  (Burkina Faso)  Bry SYLLAa, Seydou Golo BARROb,1, Jeoffray DIENDEREc, Rachid G. KONFEa  ,  Ghislain BOUDAd and Pascal STACCINIe  a Health Information System Department, Ministry of Health, Burkina Faso  b Nazi BONI University, Bobo-Dioulasso, Burkina Faso  c Health Sciences Research Institute (IRSS)  d Health District of Do, Burkina Faso  e RETINES Lab - Cote-d’Azur University, Nice, France | Not RCT / Different outcomes targeted/ Outcome evaluation method different/ Repetition |
|  | m H e a l t h I n t e r v e n t i o n s t o  C o u n t e r  N o n c o m m u n i c a b l e D i s e a s e s D e v e l o p i n g C o u n t r i e s  Still an Uncertain Promise  i n  Andrea Beratarrechea, MD, MSc*, Daniela Moyano, BSc,  Vilma Irazola, MD, MSc, Adolfo Rubinstein, MD, MSc, PhD | Not RCT / Different outcomes targeted/ Outcome evaluation method different/ Repetition |
|  | Impact of mobile phone text messaging  intervention on adherence among patients with  diabetes in a rural setting  A randomized controlled trial  Eyitayo Omolara Owolabi, MCura,∗, Daniel Ter Goon, DTecha, Anthony Idowu Ajayi, PhDb | Repetition |
|  | High Level of Acceptability and Feasibility with Low Level of Efficacy  of Daily Text-Messaging on Glycaemic Status and Self-Management:  Result of a Randomised Trial Among Low-Income Earning Black  South Africans  Eyitayo Omolara Owolabi, Daniel Ter Goon, Anthony Idowu Ajayi  Conference paper | Repetition |
|  | Factors influencing healthcare providers’  attitude and willingness to use information  technology in diabetes management  Binyam Tariku Seboka1*, Tesfahun Melese Yilma2 and Abraham Yeneneh Birhanu2 | Not RCT / Different outcomes targeted/ Outcome evaluation method different/ Repetition |
|  | Examining the Success Factors for Mobile  Applications for Self-Management of Diabetic  Treatment in a South African Context  Ganizani Fidelis Mainoti  Naomi Isabirye | Not RCT / Different outcomes targeted/ Outcome evaluation method different/ Repetition |
|  | Efficacy, acceptability and feasibility of daily  text-messaging in promoting glycaemic  control and other clinical outcomes in a low-  resource setting of South Africa: A  randomised controlled trial  Eyitayo Omolara OwolabiID1*  , Daniel Ter Goon1  , Anthony Idowu AjayiID2 | INCLUDED |
|  | Digital messaging to support control for  type 2 diabetes (StAR2D): a multicentre  randomised controlled trial  A. Farmer1*† N. Levitt2  , K. Bobrow2†, N. Leon3, N. Williams1, E. Phiri4, H. Namadingo4, S. Cooper5, J. Prince6, A. Crampin4  ,  D. Besada3, E. Daviaud3, L-M Yu1, J. N’goma7, D. Springer8, B. Pauly9, L. Tarassenko6, S. Norris10, M. Nyirenda4 and  Access  A | INCLUDED |
|  | Efficacy, acceptability and feasibility of daily  text-messaging in promoting glycaemic  control and other clinical outcomes in a low-  resource setting of South Africa: A  randomised controlled trial  Eyitayo Omolara OwolabiID1*  , Daniel Ter Goon1  , Anthony Idowu AjayiID2 | Repetition |
|  | Diabetes Buddies: Peer support through a mobile phone buddy  system  Mary Jane Rotheram-Borus, Ph.D.1  , Mark Tomlinson, Ph.D.2  , Margaret Gwegwe3  , W. Scott  Comulada1  , Dr. P.H. Neal Kaufmann, M.D.4, and Marion Keim, Ph.D.5 | Not RCT / Different outcomes targeted/ Outcome evaluation method different/ Repetition |
|  | Development and Evaluation of a Tailored Mobile Health Intervention to Improve Medication Adherence in Black Patients With Uncontrolled Hypertension and Type 2 Diabetes: Pilot Randomized Feasibility Trial Monitoring Editor: Gunther Eysenbach Reviewed by Lyndsay Nelson and Jared Magnani [Antoinette Schoenthaler](https://www.ncbi.nlm.nih.gov/pubmed/?term=Schoenthaler%20A%5BAuthor%5D&cauthor=true&cauthor_uid=32965230), EdD,^#1^ [Michelle Leon](https://www.ncbi.nlm.nih.gov/pubmed/?term=Leon%20M%5BAuthor%5D&cauthor=true&cauthor_uid=32965230), BA,^#2^ [Mark Butler](https://www.ncbi.nlm.nih.gov/pubmed/?term=Butler%20M%5BAuthor%5D&cauthor=true&cauthor_uid=32965230), PhD,^#3^ [Karsten Steinhaeuser](https://www.ncbi.nlm.nih.gov/pubmed/?term=Steinhaeuser%20K%5BAuthor%5D&cauthor=true&cauthor_uid=32965230), MS, PhD,^#4^ and [William Wardzinski](https://www.ncbi.nlm.nih.gov/pubmed/?term=Wardzinski%20W%5BAuthor%5D&cauthor=true&cauthor_uid=32965230), BS^#4^ | Not RCT / Different outcomes targeted/ Outcome evaluation method different/ Repetition |
|  | Determining the potential of mobilephone-based health interventions in Kumasi,  Ghana  Victor Stephani1, Daniel Opoku1 and Easmon Otupiri2  *Ghana Med J* 2020; 54(2): 88-92 DOI: http://dx.doi.org/10.4314/gmj.v54i2.6 | Not RCT / Different outcomes targeted/ Outcome evaluation method different/ Repetition |
|  | A cluster-randomized trial to estimate the effect of  mobile screening and treatment feedback on  HbA1c and diabetes-related complications in  Tshwane primary health care clinics, South Africa  Elizabeth Melanie Webb a,∗  , Paul Rheeder b | Not RCT / Different outcomes targeted/ Outcome evaluation method different/ Repetition |
|  | Awareness and readiness to use  telemonitoring to support diabetes care  among care providers at teaching  hospitals in Ethiopia: an institution-based  cross-­ sectional study  Binyam Tariku Seboka ‍‍ ,  1 Tesfahun Melese Yilma,2 Abraham Yeneneh Birhanu2 | Not RCT / Different outcomes targeted/ Outcome evaluation method different/ Repetition |
|  | Awareness and readiness to use  telemonitoring to support diabetes care  among care providers at teaching  hospitals in Ethiopia: an institution-based  cross-­ sectional study  Binyam Tariku Seboka ‍‍ ,  1 Tesfahun Melese Yilma,2 Abraham Yeneneh Birhanu2 | Not RCT / Different outcomes targeted/ Outcome evaluation method different/ Repetition |
|  | Assessment of Rwandan diabetic patients’  needs and expectations to develop their  first diabetes self-management smartphone  application (Kir’App)  Ther Adv Endocrinol  Metab  2019, Vol. 10: 1–21  DOI: 10.1177/  https://doi.org/10.1177/2042018819845318  https://doi.org/10.1177/2042018819845318  2042018819845318  © The Author(s), 2019.  Article reuse guidelines:  sagepub.com/journals-  permissions  Claudine B. Kabeza , Lorenz Harst, Peter E. H. Schwarz and Patrick Timpel | Not RCT / Different outcomes targeted/ Outcome evaluation method different/ Repetition |
|  | An ICT-Based Diabetes Management System Tested for  Health Care Delivery in the African Context  Claude Takenga,1 Rolf-Dietrich Berndt,1 Olivier Musongya,2  Joël Kitero,2 Remi Katoke,2 Kakule Molo,2 Basile Kazingufu,3  Malikwisha Meni,3 Mambo Vikandy,3 and Henri Takenga4 | INCLUDED |
|  | Access to mobile phone and willingness  to receive mHealth services among  patients with diabetes in Northwest  Ethiopia: a cross-sectional study  Adamu Takele Jemere,1 Yohannes Ezezew Yeneneh,1 Biniam Tilahun,1 Fleur Fritz,2  Shitaye Alemu,3 Mihiretu Kebede1,4,5 | Not RCT / Different outcomes targeted/ Outcome evaluation method different/ Repetition |
|  | Acceptability of Mobile Health Interventions to Increase Diabetic  Risk Factor Awareness Among the Commuter Population in  Johannesburg: Descriptive Cross-Sectional Study  Alex Fischer1, BSc, MPH; Martha Chadyiwa1, BSc, MBA; Ndumiso Tshuma2, BSc, MBA, PhD; Vusumuzi Nkosi1,3,4  ,  PhD | Not RCT / Different outcomes targeted/ Outcome evaluation method different/ Repetition |
|  | A qualitative study of users’ experiences  after 3 months: the first Rwandan diabetes  self-management Smartphone application  “Kir’App”  Ther Adv Endocrinol  Metab  2020, Vol. 11: 1–12  DOI: 10.1177/  https://doi.org/10.1177/2042018820914510  https://doi.org/10.1177/2042018820914510  2042018820914510  © The Author(s), 2020.  Claudine B. Kabeza , Lorenz Harst, Peter E.H. Schwarz and Patrick Timpel | Not RCT / Different outcomes targeted/ Outcome evaluation method different/ Repetition |
|  | Abstract 14912: A Randomized Trial Using Mobile Short-Text Messaging to Improve Cardiovascular Risk Profile in Poorly Controlled Diabetes in Kenya  [Gurveen Soin](https://www.ahajournals.org/doi/abs/10.1161/circ.138.suppl_1.14912#con1), [Nancy Kunyiha](https://www.ahajournals.org/doi/abs/10.1161/circ.138.suppl_1.14912#con2), [Jasmit Shah](https://www.ahajournals.org/doi/abs/10.1161/circ.138.suppl_1.14912#con3), [Kinita Patel](https://www.ahajournals.org/doi/abs/10.1161/circ.138.suppl_1.14912#con4), [Christopher Arisi](https://www.ahajournals.org/doi/abs/10.1161/circ.138.suppl_1.14912#con5), [Erick Njenga](https://www.ahajournals.org/doi/abs/10.1161/circ.138.suppl_1.14912#con6), [Mohamed Jeilan](https://www.ahajournals.org/doi/abs/10.1161/circ.138.suppl_1.14912#con7), [Shaheed Sorathia](https://www.ahajournals.org/doi/abs/10.1161/circ.138.suppl_1.14912#con8), [Leonard M Ngunga](https://www.ahajournals.org/doi/abs/10.1161/circ.138.suppl_1.14912#con9), and [Anders L Barasa](https://www.ahajournals.org/doi/abs/10.1161/circ.138.suppl_1.14912#con10) | FULL TEXT NOT AVAILABLE |
