# Supplementary material for: Implementation and impact of mhealth in the management of diabetes mellitus in Africa: A systematic review and meta-analysis
Source: PLOS Digit Health. 2025 Apr 8;4(4):e0000776. doi: 10.1371/journal.pdig.0000776 (PMC11978007; doi:10.1371/journal.pdig.0000776)
Supplement: Attachment — Submitted filename: PDIG-D-23-00388_reviewerSuggested.pdf [file pdig.0000776.s006.pdf]

PLOS Digital Health

IMPLEMENTATION AND IMPACT OF mHEALTH IN THE MANAGEMENT OF DIABETES MELLITUS IN AFRICA: A SYSTEMATIC REVIEW

--Manuscript Draft--

Manuscript Number:	PDIG-D-23-00388
Article Type:	Research Article
Full Title:	IMPLEMENTATION AND IMPACT OF mHEALTH IN THE MANAGEMENT OF DIABETES MELLITUS IN AFRICA: A SYSTEMATIC REVIEW
Short Title:	Implementation and impact of mHealth in the management of diabetes mellitus in Africa
Corresponding Author:	Franklin Okechukwu Dike, MBBCH<br>University of Uyo Teaching Hospital<br>Uyo, Akwa Ibom NIGERIA
Order of Authors:	Franklin Okechukwu Dike, MBBCH, FWACP
	Jean Claude Mutabazi, MD, PhD
	Ezekiel Musa, MBBS, FWACP, PhD
	Blessing Chinenye Ubani, MBBCh, FWACP
	Ahmed Sherif Isa, B.Sc, M.Sc, PhD
	Chidiebele Malachy Ezeude, MBBS, FWACP
	Henry Iheonye, MBBS, FWACP
	Isah Idris Ainavi, MBBS, FWACP
Keywords:	mHealth; diabetes mellitus; Africa; eHealth; healthcare services delivery
Abstract:	Background<br><br>The World Health Organization (WHO) has proposed the concept of mobile health to support healthcare systems delivery worldwide. Mobile health (mHealth) involves the use of Information and Communication Technology (ICT) for health care provision or delivery services. Africa has seen a remarkable increase in mobile phone availability and usage in the last decade. The incidence and prevalence of diabetes mellitus (DM) in Africa have also been on the increase in the last decade, in sharp contrast to an ailing healthcare system. We aimed to review the extent of implementation of mHealth in the management of diabetes mellitus in Africa and estimate its impact in helping patients achieve desired glycemic targets, sustain control and prevent complications in the past decade.<br><br>Methods and analysis<br><br>The studies assessing the utilization of mHealth in the management of patients with DM in Africa were considered based on the PICO method: Population; Intervention; Comparator; and Outcomes. MEDLINE, PubMed, SCOPUS, and the Pan African Clinical Trials Registry, among others, were searched. Two authors independent of each other screened titles and abstracts retrieved using the search strategy, retrieved the full-text articles, and assessed them for eligibility, extracting data thereafter. A third independent reviewing author was brought in to resolve any disagreement between the two authors by discussion. The revised Cochrane Collaboration Risk of Bias Tool was used to assess the quality of included studies. A narrative synthesis of extracted data was done due to the paucity of eligible studies with the results summarized. PROSPERO CRD42021218674<br><br>Results<br><br>Among the six included studies, none measured the mean FPG or percentage changes as primary outcomes. Five measured the percentage change in HbA1c from

Powered by Editorial Manager® and ProduXion Manager® from Aries Systems Corporation

	baseline to the end of the study. The percentage change in HbA1c from the baseline ranged from 3.6% in the least to 20.53% in the highest study, achieving significance in 2 studies, and showing a positive trend in 2 others compared with standard of care. None of the studies addressed the secondary outcomes of measuring the direct relationships between these mHealth interventions and the prevention/early detection of diabetes complications. All six studies showed indications for the acceptability of mHealth interventions through administered questionnaires. No details of harm were available for assessment, though one study stated the limitation imposed by the high cost of implementation of its preferred mHealth strategy.<br><br>Conclusion<br><br>This systematic review demonstrated a paucity of useful data or evidence on mHealth usage in Africa. Findings show a positive trend in mHealth efficacy in the care of patients with diabetes mellitus, particularly improvement in glycated hemoglobin. The study findings may provide useful information for policymaking in the continent in the aspect of mHealth utilization.<br>We recommend more well-designed randomized controlled trials investigating mHealth intervention/strategies in the management of DM in Africa.<br><br>Systematic review registration: PROSPERO<br><br>CRD42021218674
**Additional Information:**	
**Question**	**Response**
**Government Employee**<br><br>Are you or any of the contributing authors an employee of the United States government?<br><br>Manuscripts authored by one or more US Government employees are not copyrighted, but are licensed under a CC0 Public Domain Dedication, which allows unlimited distribution and reuse of the article for any lawful purpose. This is a legal requirement for US Government employees.<br><br>This will be typeset if the manuscript is accepted for publication.	No - No authors are employees of the U.S. government.
**Financial Disclosure**<br><br>Enter a financial disclosure statement that describes the sources of funding for the work included in this submission and the role the funder(s) played. This includes grants and any commercial funding of the work or authors.	No funding was received for this research.

This statement will be typeset if the manuscript is accepted for publication.<br><br>Review the submission guidelines and the instructions link below for detailed requirements and guidance.	
**Competing Interests**<br><br>On behalf of all authors, disclose any competing interests that could be perceived to bias this work.<br><br>This statement will be typeset if the manuscript is accepted for publication.<br><br>Review the instructions link below and PLOS Digital Health's competing interests policy to determine what information must be disclosed at submission.	All authors disclose no competing interests.
**Data Availability**<br><br>Provide a **Data Availability Statement** in the box below. This statement should detail where the data used in this submission can be accessed. This statement will be typeset if the manuscript is accepted for publication.<br><br>Before publication, authors are required to make all data underlying their findings fully available, without restriction. Review our PLOS Data Policy page for detailed information on this policy. Instructions for writing your Data Availability statement can be accessed via the Instructions link below.	All data will be available upon publication without restrictions.

Manuscript Click here to access/download;Manuscript;Manuscript.docx

IMPLEMENTATION AND IMPACT OF mHEALTH IN THE MANAGEMENT OF DIABETES MELLITUS IN AFRICA: A SYSTEMATIC REVIEW

Franklin O. Dike[1], Jean Claude Mutabazi[2], Ezekiel Musa[3,4], Blessing Ubani[1, 5], Ahmed Sherif Isa[6,7], Chidiebele M. Ezeude[8, 9], Henry Iheonye[10], Isah Idris Ainavi[11, 12]

Corresponding Author:

Franklin O. Dike MBBCh, FWACP

Neurology Unit, Department of Internal Medicine,

University of Uyo Teaching Hospital,

P.M.B 1136 Uyo, Akwa Ibom State, Nigeria.

Email: frankincense4m@uuthuyo.net, frankincense4m@gmail.com

Affiliations:

1 University of Uyo Teaching Hospital, Internal Medicine

2 Department of Social and Preventive Medicine, École de Santé Publique, Université de Montréal.

3 Kaduna State University, Internal Medicine

4 University of Cape Town, Medicine

5 University of Uyo, Internal Medicine

6 Stellenbosch University, Faculty of Medicine and Health Sciences, Department of Anesthesiology and Critical Care

7 Ahmadu Bello University, Human physiology

8 Nnamdi Azikiwe University, Internal Medicine

9 Nnamdi Azikiwe University Teaching Hospital, Internal Medicine

10 Federal Medical Center Lokoja, Internal Medicine

11 Kaduna State University, Chemical Pathology and Immunology

12 Barau Dikko Teaching Hospital Kaduna, Chemical Pathology

Email addresses of co-authors:

Jean Claude Mutabazi: mutajeanc@yahoo.fr,

Ezekiel Musa:MSXEZE001@myuct.ac.za,

Blessing Ubani: blessiykebani@gmail.com,

Ahmed Sherif Isa:isa.a.sherif@gmail.com,

Chidiebele Ezeude: docchidi@yahoo.co.uk, cm.ezeude@unizik.edu.ng

Henry Iheonye: henryio2010@gmail.com

Isah Idris Ainavi: ozioohoku@yahoo.com

ABSTRACT

Background: The World Health Organization (WHO) has proposed the concept of mobile health to support healthcare systems delivery worldwide. Mobile health (mHealth) involves the use of Information and Communication Technology (ICT) for health care provision or delivery services. Africa has seen a remarkable increase in mobile phone availability and usage in the last decade. The incidence and prevalence of diabetes mellitus (DM) in Africa have also been on the increase in the last decade, in sharp contrast to an ailing healthcare system. We aimed to review the extent of implementation of mHealth in the management of diabetes mellitus in Africa and estimate its impact in helping patients achieve desired glycemic targets, sustain control and prevent complications in the past decade.

Methods and analysis: The studies assessing the utilization of mHealth in the management of patients with DM in Africa were considered based on the PICO method: Population; Intervention; Comparator; and Outcomes. MEDLINE, PubMed, SCOPUS, and the Pan African Clinical Trials Registry, among others, were searched. Two authors independent of each other screened titles and abstracts retrieved using the search strategy, retrieved the full-text articles, and assessed them for eligibility, extracting data thereafter. A third independent reviewing author was brought in to resolve any disagreement between the two authors by discussion. The revised Cochrane Collaboration Risk of Bias Tool was used to assess the quality of included studies. A narrative synthesis of extracted data was done due to the paucity of eligible studies with the results summarized. **PROSPERO** CRD42021218674

Results: Among the six included studies, none measured the mean FPG or percentage changes as primary outcomes. Five measured the percentage change in HbA1c from baseline to the end of the study. The percentage change in HbA1c from the baseline ranged from 3.6% in the least to 20.53% in the highest study, achieving significance in 2 studies, and showing a positive trend in 2 others compared with standard of care.

None of the studies addressed the secondary outcomes of measuring the direct relationships between these mHealth interventions and the prevention/early detection of diabetes complications. All six studies showed indications for the acceptability of mHealth interventions through administered questionnaires. No details of harm were available for assessment, though one study stated the limitation imposed by the high cost of implementation of its preferred mHealth strategy.

Conclusion: This systematic review demonstrated a paucity of useful data or evidence on mHealth usage in Africa. Findings show a positive trend in mHealth efficacy in the care of patients with diabetes mellitus, particularly improvement in glycated hemoglobin. The study findings may provide useful information for policymaking in the continent in the aspect of mHealth utilization.

We recommend more well-designed randomized controlled trials investigating mHealth intervention/strategies in the management of DM in Africa.

Systematic review registration: PROSPERO CRD42021218674

Keywords: mHealth, diabetes mellitus, Africa, eHealth, healthcare services delivery.

INTRODUCTION

In 2009, the Global Observatory for eHealth (GOe) a subsidiary of the World Health Organization devoted an entire section of its global survey to mobile health (mHealth).[1] The survey sought to determine the extent of adoption of mHealth interventions/initiatives, the types of initiatives, the status of the evaluation and the barriers to implementation across 112 member states.[1] mHealth was defined by GOe as medical and public health practice supported by mobile devices such as mobile phones, patient monitoring devices, personal digital assistants and other wireless devices.[1] mHealth involves the use of Information and Communication Technology for healthcare provision or delivery services. This concept mainly involves the employment of the basic functionality of mobile phones in the delivery of healthcare services. mHealth employs various functions, encompassing basic features like voice calls and Short Messaging Services (SMS), alongside more sophisticated capabilities and applications such as the Global Positioning System (GPS) General Packet Radio Service (GPRS) and third and fourth generation telecommunications3G, 4G, 5G and Bluetooth technology either as part of the mobile phones or as independent wireless devices.[1] This survey by the GOe was a follow-up to an earlier survey in 2005. It showed that mHealth was only just emerging in many member states, through experimentation in their health sectors. Greater strategic implementation was observed in high-income countries, with up to 87% implementation of various mHealth categories, compared with low-income and middle-income countries (LMIC) with about 77%.[2] Most LMICs could only boast of one mHealth intervention being implemented at the time of the GOe survey in 2005[1]. Africa had the lowest number of responders in that survey, and the lowest mHealth usage, with a mobile phone penetration of 47%.[2] In the past decade, there has been a progressive increase in the penetration and usage of mobile phones in Africa. A recent country-by-country survey in Africa [involving six countries namely South Africa, Nigeria, Tanzania, Ghana, Senegal and Kenya]showed a 91% penetration in South Africa, 80% in Nigeria, and 75% in Tanzania which had the lowest.[3] Intriguingly, there has been a phenomenal increase in the prevalence of non-communicable diseases (NCDs) in Africa.[4] This in addition to the already high prevalence of infectious diseases notably malaria, HIV and tuberculosis resulting in a double burden of diseases in Africa. Additionally, this epidemiological transition has witnessed an increasing incidence of diabetes mellitus (DM) and hypertension across populations in Africa.[5] The transition is thought to be a resultant effect of increased urbanization, adoption of Western diets, sedentary lifestyles (leading to obesity), and longevity.[6,7] Diabetes mellitus remains one of the NCD in Africa and indeed globally with the highest economic expenditure.[8] Therefore, prevention of DM and improving self-management among people with DM constitute major public health priorities. mHealth has theoretically been thought to be a suitable supportive intervention in the management of DM to help patients achieve desired glycemic targets, sustained control and prevent complications.[9,10] A 2011 meta-analysis involving 1657 individuals with either type 1 or type 2 DM using SMS messages to send self-monitored blood glucose values and receive self-management information showed a 0.5% reduction in glycated hemoglobin (HbA1c) over 6 months in mHealth intervention groups compared to the control groups.[10] More recently, another meta-analysis in 2017 which included 13 studies reported a mean reduction of 0.44% in HbA1c in mHealth intervention groups compared to controls.[10] Furthermore, it reported an increased perception of self-care.[10] The WHO recommends an HbA1c glycemic target of ≤7% for most patients with type 2 DM.[11] Thus, the growing increase in mobile phone usage across Africa is an encouraging trend in terms of its potential to support existing overburdened healthcare systems in combating both communicable and non-communicable diseases. In the absence of a more recent global survey, it is still pertinent to focus on Africa, and review its adoption and implementation of mHealth strategies to support most of its ailing healthcare delivery

systems, and probably identify any impeding factors for its implementation. Currently, no data shows the degree of implementation of mHealth in Africa as recommended by WHO-GOe, especially regarding the management of DM. While a systematic review of economic evaluation of mHealth solutions from studies conducted mostly in upper and upper-middle-income countries concluded that mHealth is cost-effective,[12] same cannot be said for the African continent due to unavailability of data. The usage of mHealth is not without problems as reported by various researchers. These issues encompass concerns such as breaches of privacy, poor quality of data due to app inadequacies, and unsafe action or inaction of users arising from poor advice from apps or faulty algorithms.[13] This study aimed to review the extent to which mHealth is implemented in the management of DM in Africa and estimate its impact in the past decade. The objective of this systematic review is to provide data that will offer guidance to clinicians, diabetes nurse educators and dieticians on the benefits and compelling need for implementation of mHealth. Furthermore, the data obtained will be useful for developing policy frameworks that aim to incorporate mHealth into the standard of diabetes care in Africa.

OBJECTIVES

1. To determine the extent of implementation of mHealth strategies, and the common forms of mHealth used in the management of DM in patients in Africa.

2. To determine the impact/efficacy of mHealth interventions in helping patients achieve treatment goals in diabetes in Africa.

3. To determine the impact of mHealth on early detection, management and outcome of some diabetic complications.

4. To identify any factors impeding the utilization and implementation of mHealth in Africa in the management of patients with DM.

5. To determine the possible harms and cost of implementation of mHealth strategies in Africa.

METHODS

Eligibility criteria

We considered all published randomized clinical trials and non-randomized

trials, quasi-randomized clinical trials and observational studies deploying one or more mHealth strategies in the management of DM in Africa done in the continent between January 2010 and October 2021, including unpublished studies from the Pan African Clinical Trials Registry (PACTR).

All the articles considered had similar standards for diagnosis of DM and treatment targets (fasting plasma glucose and HbA1c) based on the guidelines of the WHO and the International Diabetes Federation (IDF).

There were no language restrictions, however, all articles turned up by the search were in the English language.

We excluded studies testing the acceptability of mHealth among health professionals and duplicate publications.

PARTICIPANTS

The review considered studies on African type 2 DM patients residing in Africa with no restrictions to age or sex. Studies reporting type I DM, or a combination of type 1 and type 2 DM were excluded.

INTERVENTIONS

Studies that investigated the implementation and or efficacy of any mHealth modality in the management of type 2 DM were included. The mHealth interventions reviewed include individual behaviour change, chronic disease self-management, clinic appointment reminders or clinical diagnostic aid. mHealth strategies under scrutiny included but were not limited to the use of SMS text messages, phone calls, use of customized mobile phone applications and other components of mHealth. Most of the studies paid little attention to the secondary outcomes concerning complication prevention by early detection using these mHealth interventions/delivery strategies.

OUTCOME MEASURES

The primary outcome measure assessed the impact and efficacy of adding mHealth modalities intervention to the treatment plan of the treatment groups viz a viz the changes in mean HbA1c or FPG from baseline to the end of the trial duration and comparing this change to a comparators group on the conventional therapy.

Secondary outcomes included the rate or percentage reduction in specific complications of DM over the trial periods from a baseline. However, most of the studies included failed to investigate this aspect. Studies directed at mHealth strategies targeted directly at these secondary outcomes were also not available for separate consideration.

SEARCH STRATEGY AND SELECTION CRITERIA

The following electronic databases were searched for relevant studies implementing mHealth for DM in Africa published between January 2010 and October 2020: Medline database (PubMed), Cochrane Register of Controlled Trials (CENTRAL) and Embase (Scopus). The PACTR was also searched for ongoing studies that met the criteria, as well as recently completed but unpublished studies.

Medical Subject Headings (MeSH) were used to search through the Medline database for all available studies on the subject. Terms describing all the available m-Health categories or relating to them were used in the MeSH search, using DM and Africa (regions and countries) as further filters as seen below.

(implement* OR execute* OR administrate* OR organize* OR fulfill* OR perform* OR utilize* OR manage* OR operat* OR act* OR realize* OR effect* OR impact*)

AND

(mHealth* OR "mobile health*" OR eHealth* OR "electronic health*")

AND

(diabetes* OR diabetes mellitus, type 1/ OR diabetes mellitus, type 2 OR diabetes, gestational/)

AND

Africa/ OR “Africa South of the Sahara”/ OR “Sub-Saharan Africa”/ OR north Africa/ OR Africa, Northern/ Egypt or Libya OR Tunisia OR Algeria OR Morocco OR "Western Sahara" OR Angola/ OR Benin/ OR Botswana/ OR Burkina Faso/ OR Burundi/ OR Cameroon/ OR Cape Verde/ OR Central African Republic/ OR Chad/ OR Comoros/ OR Congo/ OR Brazzaville/ OR Cote d'Ivoire/ OR Djibouti/ OR Equatorial Guinea/ OR Eritrea/ OR Ethiopia/ OR Gabon/ OR Gambia/ OR Ghana/ OR Guinea/ OR Bissau/ OR Kenya/ OR Lesotho/ OR Liberia/ OR Madagascar/ OR Malawi/ OR Mali/ OR Mauritania/ OR Mauritius/ OR Mozambique/ OR Namibia/ OR Niger/ OR Nigeria/ OR Rwanda/ OR Sao Tome e Principe/ OR Senegal/ OR Seychelles/ OR Sierra Leone/ OR Somalia/ OR South Africa/ OR South Sudan/ OR Sudan/ OR Swaziland/ OR Eswatini OR Tanzania/ OR Togo/ OR Uganda/ OR Western Sahara/ OR Zaire/ OR Zambia/ OR Zimbabwe/

All available controlled trials using this search were retrieved. Similar methods were used in searching through CENTRAL, and Embase (Scopus). Hand searching and snowballing were used, when necessary, through all the databases to retrieve all relevant articles. Data from dissertations that are indexed were retrieved where possible. The PACTR was also searched for any ongoing, recently published or unpublished trials meeting the inclusion criteria. All searches were carried out using the Preferred Reporting Items for Systematic Reviews and Meta-Analyses guidance of Population, Intervention, Comparator and Outcome method as listed in the table below (table 2).

The protocol was registered with the international prospective register of systematic reviews (**PROSPERO** CRD42021218674), and has been detailed previously.[14]

The Rayyan application was adopted to retrieve, screen for duplicates and reference all articles.

STUDY RECORDS

Data management/selection process

Screening of titles and abstracts retrieved using the search strategy and from additional sources was done by reviewers in multiples of two (2) to select those studies that meet the inclusion criteria outlined above [FD and BU, EM and CE, JC and II, IS and HI].

Thereafter, the selected full-text articles were retrieved and independently assessed for eligibility again by the two authors. A third reviewing author was brought in from another group to resolve any disagreement between the two authors in any group by discussion. The selected studies were then included for data extraction and analysis.

Data collection process

A Microsoft Word form designed and standardized for the extraction of data, assessment of study quality and synthesis of evidence was used to extract data. Data extracted from the articles selected included the names of authors, year of publication, country of research, sample sizes of both study and control groups, drug treatments used in each study, particular mHealth intervention used in the study with the strategy of delivery and functionalities involved, type and duration of studies, study methodology, outcomes and times of measurement including mean baseline glycemic control using HbA1cor fasting plasma glucose, the mean glycemic control at the end of the study using HbA1c and fasting plasma glucose, the percentage change in HbA1c, number of hypoglycemic events and hyperglycemic crises, quality of life and body mass index (BMI) changes; harms including loss of privacy and inappropriate clinical events; cost of implementation; and indicators of acceptability of intervention information for assessment of risk of bias.

RISK OF BIAS IN SELECTED STUDIES

The methodological quality of the included articles was assessed independently initially by one author [FD] using the Revised Cochrane Risk of Bias tool for RCTs. This was then vetted by two other authors [EM] and [JCM]. Disagreements were then resolved by discussion.[15]

The assessments were made using standardized judgement processes in the following areas:

1. Concealment of treatment allocation: was the allocated treatment adequately concealed from study participants, clinicians and other healthcare or research staff at the enrolment stage?

2. Blinding: were the personnel assessing outcomes and analyzing data sufficiently blinded to the intervention allocation throughout the trial?

3. Completeness of outcome data: were participant exclusions, attrition and incomplete outcome data adequately addressed in the published report?

4. Selective outcome reporting: is there evidence of selective outcome reporting and might this have affected the study results?

5. Other sources of bias: was the trial free of any other problems that could produce a high risk of bias?

The automatically generated conclusions of the Revised Cochrane risk of bias tool were used, hence increasing inter-assessor agreement,

DATA SYNTHESIS

We used a narrative synthesis to describe the interventions used in the studies included such as individual behaviour change, chronic disease self-management, clinic appointment reminders or clinical diagnostic aid. Similarly included were the mHealth category used and the mobile phone functionalities employed, the characteristics of the sample population and the expected outcomes especially changes in HbA1c, number of hypoglycemic events and hyperglycemic crises, quality of life and BMI changes. We expected to use risk ratios for dichotomous outcomes and standardized mean differences for continuous

outcomes to provide summaries of the intervention effects with extracted data. However, the paucity of eligible studies impeded these calculations.

We extracted the main outcomes of interest where available, analyzed and presented them as narrative, alongside other constructed variables of interest. Thereafter the results were expressed statistically in a table comparing outcomes across the included studies with key conclusions summarized in the evidence table. However, it was impossible to run a meta-analysis as the eligible studies had significant limitations in number, sample size and heterogeneity of study outcomes. We therefore attempted to summarize our conclusions in a narrative summary.

RESULTS

The flowchart of the search results is shown in Figure 1. A total of 403 duplicates were removed. After title and abstract screening, studies 1,012 titles were excluded leaving a total of 28 articles screened for full-text eligibility. Assessment of the full-text articles resulted in the exclusion of an additional 22 articles leaving a final total of 6 studies meeting the eligibility criteria for the systematic review. The search strategy yielded 1,443 articles in all. [MEDLINE = 305, EMBASE = 591, Web of Science = 244, Cochrane review = 150, Global Health citations = 102].

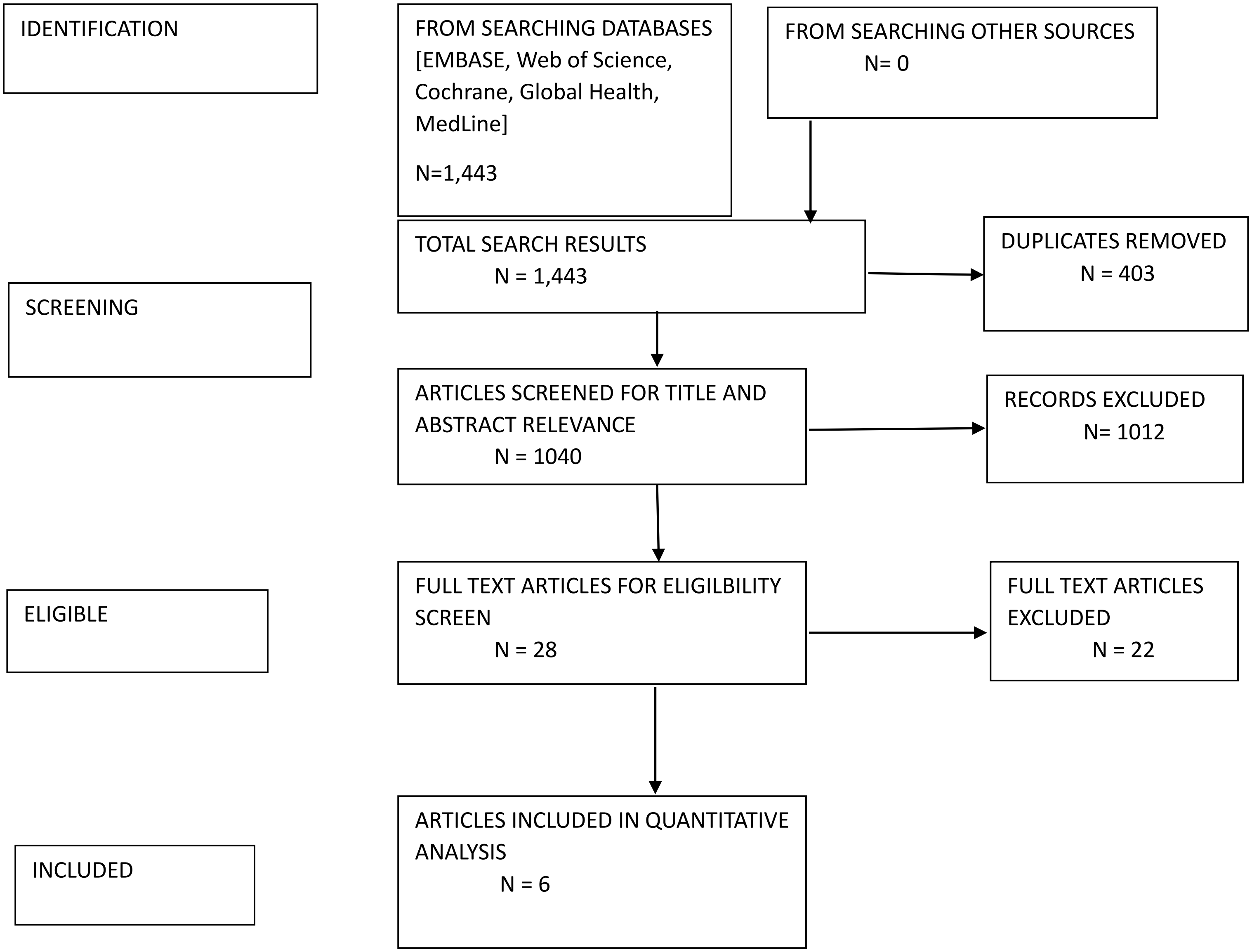

FIGURE 1. FLOW CHART ON SELECTION OF ELIGIBLE STUDIES

CHARACTERISTICS OF INCLUDED STUDIES

All six eligible studies were experimental in design and published between 2010 and 2021. None of the observational studies met the eligibility criteria. All the eligible studies investigated a mHealth intervention using a similar mHealth delivery strategy [SMS text messaging] apart from one which used a phone call and another which used a mobile application. All targeted the same intervention, which is modifying the patient's behaviour and attitude to treatment. The eligible studies were conducted in Ghana, Egypt, South Africa, Senegal and the DRC. The characteristics of the eligible studies are presented in Table 1.

Table 1. Characteristics of the eligible studies

**Author**	**Year**	**Country**	**Study aim**	**Study design**	**Sampling strategy**
Haitham et al	2017	Egypt	To examine the feasibility of SMS education among diabetic patients in Egypt, and assess the impact of educational text messages, compared to traditional paper-based methods, on glycemic control and self-management behaviors.	Randomized controlled trial	None given
Ernest Asante et al	2020	Ghana	To evaluate the feasibility and effectiveness of a nurse-led mobile phone call intervention on glycemic management and adherence to self-management practices among patients with type 2 diabetes mellitus (T2DM) in Ghana.	Randomized controlled trial	None given
Matthieu Wargny et al	2018	Senegal	To evaluate the impact of mHealth on diabetes control.	Randomized controlled trial	Consecutive
Owolabi et al	2020	South Africa	To determine the efficacy, acceptability and feasibility of text messaging in improving glycemic control and other clinical outcomes among individuals living with diabetes in low-resource settings in Eastern Cape, South Africa.	Randomized controlled trial	None given
Farmer et al	2021	South Africa	To test the effectiveness of SMS-text messaging in improving outcomes in adults with type 2 diabetes.	Randomized controlled trial	Sequential

Takenga et al	2014	Democratic Republic of Congo	To involve patients in the treatment process and motivate them for active participation.	Randomized controlled trial	Sequential

METHODOLOGICAL QUALITY OF INCLUDED STUDIES

This was assessed using the revised Cochrane Risk of Bias tool[16], as all the included studies were randomized control trials with varying degrees of methodological rigor. The summary of the Risk of Bias assessment for each study is depicted in Table 2. The outcome under examination is the change in HbA1c over the trial duration.

Table 2. Risk of Bias assessment

	**Randomization process**	**Deviation from intended intervention**	**Missing outcome data**	**Measure-ment of the outcome**	**Selection of the reported result**	**Overall**
Haitham et al 2017	low	low	low	low	low	low
Ernest Asante et al 2020	high	low	low	low	high	high
Matthieu Wargny et al 2018	low	low	low	low	high	low
Owolabi et al 2020	low	low	low	low	low	low
Farmer et al 2021	Some concerns	low	low	low	low	low
Takenga et al 2014	low	low	low	low	low	low

EFFICACY OF THE INTERVENTIONS AND EFFICACY OF THE MHEALTH DELIVERY STRATEGIES

Five of the six studies targeted behavioural change by reaching remotely using SMS text messages and phone calls, while one study combined this with a direct feedback system. One of the studies did not provide the desired primary outcome of either HbA1c or FPG but used random blood glucose as its primary outcome. All the studies were hospital-based and interventional, with follow-up periods ranging from 8 weeks to 52 weeks. They studied only T2DM population and were conducted between 2014-2020. A clearly defined sampling strategy was available in only 3 studies, with clear randomization techniques. The study with the largest sample size of 1186 had a 91% response rate. Drug treatments for enrolled patients were heterogeneous across the board, including oral antihyperglycemic agents and

insulin therapy. None of the included studies measured primary outcomes in mean FPG or percentage changes. Five measured the percentage change in HbA1c from baseline to the end of the study. The percentage change in HbA1c from the baseline ranged from 3.6% in the least to 20.53% in the highest study, achieving significance in 2 studies, and showing a positive trend in 2 others compared with standard of care. None of the studies addressed the secondary outcomes of measuring the direct relationships between these mHealth interventions and the prevention/ early detection of diabetes complications. All six studies showed indications for the acceptability of mHealth interventions through administered questionnaires. No details of harms were available for assessment, though one study stated the limitation imposed by the high cost of implementation of its preferred mHealth strategy (Table 3).

Table 3. Summary of studies on interventions and efficacy of the mHealth delivery

**Author/Year/ Country**	**Study aim**	**Study design/Sampling strategy**	**mHealth tool/strategy**	**mHhealth acceptance/use /response rate**	**mHhealth intervention outcomes**
Haitham et al	To examine the feasibility of SMS education among diabetic patients in Egypt, and assess the impact of educational text messages, compared to traditional paper-based methods, on glycemic control and self-management behaviours.	Randomized controlled trial	Reaching remotely via SMS/call	74.4%	10.7 % HbA1c reduction
Ernest Asante et al	To evaluate the feasibility and effectiveness of a nurse-led mobile phone call intervention on glycemic management and adherence to self-management practices among patients with type 2 diabetes mellitus (T2DM) in Ghana.	Randomized controlled trial	Reaching remotely via SMS/call	100%	15.8 % HbA1c reduction
Matthieu Wargny et al	To evaluate the impact of mHealth on diabetes control.	Randomized controlled trial	Reaching remotely via SMS/call	98%	3.6 % HbA1c reduction
Owolabi et al	To determine the efficacy, acceptability and feasibility of text-messaging in improving glycemic control	Randomized controlled trial	Reaching remotely via SMS/call	100%	

	and other clinical outcomes among individuals living with diabetes in low-resource settings in Eastern Cape, South Africa.				
Farmer et al	To test the effectiveness of SMS-text messaging in improving outcomes in adults with type 2 diabetes.	Randomized controlled trial	Reaching remotely via SMS/call	91%	
Takenga et al	To involve patients in the treatment process and motivate them for active participation	Randomized controlled trial	Direct feedback	100%	20.53 % HbA1c reduction

DISCUSSION

The study aimed to demonstrate the extent to which mHealth has been implemented and the dimension of impact attributable to mHealth implementation in Africa by identifying the best evidence available. Most of the included studies either showed a significant effect on improving the primary outcomes or a positive trend, but the paucity of studies and their heterogeneity did not allow for a meta-analysis hence precluding more concrete conclusions for the African continent. This should therefore be a stimulus for future research implementing mHealth in DM management in Africa as compared to other continents in the world where implementation has exceeded 87% in some high-income countries.

There is a paucity of evidence for mHealth utilization, impact and efficacy in Africa making a comparison of this result with previous evidence almost impossible. However, the available global surveys of mHealth implementation carried out by the WHO noted a higher strategic implementation of mHealth in high-income countries (HICs). It reaches as high as 87% across all mHealth categories, in stark contrast to the notably low implementation levels observed in Africa during the same survey. Only one mHealth strategy was implemented at the time, with a low phone penetration of 47%. This paucity is confirmed in a global systematic review by Mao et al studying the global impact of mHealth in the management of DM and hypertension.[17] Out of 51 studies finally included from a total of 1747 articles only 15 studies were conducted in developing countries, two (2) from Africa.[17] The review included a study from Egypt and another from South Africa. They were able to demonstrate that integrating mHealth into care had a positive impact on countries with different levels of economic development and that better outcomes follow the combination of mHealth with human intelligence.[17] Another systematic review by Stevens et al studying the effectiveness of digital health technologies for patients with diabetes mellitus included 25 RCTs in its final analysis with 3,360 patients included.[18] None of the included studies was conducted in Africa.[18] It showed an overall greater improvement in HbA1c directly attributed to the use of a mHealth intervention compared with usual care for patients with T1DM, T2DM and Prediabetes.[18] In our review, four of the six studies demonstrated either a positive trend to improved efficacy of glycemic control measured by HbA1c or outright significant benefit. This finding is consistent with global findings reported by Mao et al. and Stevens et al.[17,18] Although marked by limitations, it is adequate evidence to guide policies directed at increasing adoption of mHealth interventions for the management of diabetes mellitus in a region grappling with poor health care systems and the burden of communicable diseases.

IMPLICATIONS FOR FUTURE RESEARCH

With the increasing uptake of mobile phones in the African continent and the dearth of strong healthcare systems, mHealth interventions remain unharnessed in the management of DM as practiced in the HICs where various mHealth strategies are already massively deployed for self-management of the disease. While the great contrast in uptake and implementation of mHealth interventions may be due to limited resources, limited awareness, and limited research, it is hoped that the outcome of this study will trigger intentional strategic policy-making by policymakers geared at increasing the implementation of mHealth interventions in the delivery of DM care.

STUDY LIMITATIONS AND STRENGTHS

Only six studies were eligible for inclusion, thus providing a limited body of evidence. This reflects the paucity of research in this direction in Africa.

FUNDING SOURCES

No external funding was available for this research.

COMPETING INTERESTS

All authors declare no competing interests.

DATA AVAILABILITY

All data will be made available on publication.

CONCLUSION

The study shows mHealth is beneficial in improving glycemic control (HbA1c) in patients with diabetes mellitus and its utilization could be essential in preventing and or delaying diabetes complications. Furthermore, the study shows limited data on mHealth interventions in diabetes care in Africa. We therefore recommend more well-designed randomized controlled trials investigating mHealth interventions/strategies in the management of diabetes mellitus in Africa.

REFERENCES

1 WHO Global Observatory for eHealth, World Health Organization. MHealth: new horizons for health through mobile technologies. [Internet]. Geneva: World Health Organization, 2011. Available: http://www. who. int/ goe/ publications/ goe_ mhealth_ web. pdf [Accessed 06 Oct 2020].

2 GSM Assoc. *African Mobile Observatory 2011 driving economic and social development through mobile services*, 2011.

3 NW 1615 L. St, Suite 800Washington, Inquiries D 20036USA202-419-4300| M857-8562| F-419-4372| M. Basic mobile phones more common than smartphones in sub-Saharan Africa [Internet]. Pew Research Center's Global Attitudes Project, 2018. Available: https://www. pewresearch. org/ global/ 2018/ 10/ 09/ majorities- in- sub- Saharan Africa-own- mobile- phones- but- smartphone- adoption- is- modest/[Accessed 05 Oct 2020].

4 Smartphone and Mobile Internet Penetration In Africa And Globally [Internet]. GeoPoll, 2019. Available: https://www. geopoll. com/ blog/smartphone- mobile- internet- penetration- Africa/ [Accessed 05 Oct2020].

5 Frenk J, Bobadilla JL, Sepuúlveda J, *et al*. Health transition in middle-income

countries: new challenges for health care. *HealthPolicy Plan* 1989; 4:29–39.

6 Osei K. Global epidemic of type 2 diabetes: implications for developing countries. *Ethn Dis* 2003;13: S102–6.

7 Young LR, Nestle M. The contribution of expanding portion sizes to the US obesity epidemic. *Am J Public Health* 2002; 92:246–9.

8 Kengne AP, June-RoseMcHiza Z, Amoah AGB, *et al*. Cardiovascular diseases and diabetes as economic and developmental challenges in Africa. *Prog Cardiovasc Dis* 2013; 56:302–13.

9 Shan R, Sarkar S, Martin SS. Digital health technology and mobile devices for the management of diabetes mellitus: state of the art. *Diabetologia* 2019;62:877–87.

10 Bonoto BC, de Araújo VE, Godói IP, *et al*. Efficacy of mobile Apps to support the care of patients with diabetes mellitus: a systematic review and meta-analysis of randomized controlled trials. *JMIRMhealth Uhealth* 2017;5: e4.

11 World Health Organization. *Diagnosis and management of type 2 diabetes (HEARTS-D). (WHO/UCN/NCD/20.1)*. Geneva, 2020.

12 Iribarren SJ, Cato K, Falzon L, *et al*. What is the economic evidence for mHealth? A systematic review of economic evaluations of mHealth solutions. *PLoS One* 2017;12: e0170581.

13 Lewis TL, Wyatt JC. mHealth and mobile medical Apps: a framework to assess risk and promote safer use. *J Med Internet Res*2014;16: e210.

14 Dike FO, Mutabazi JC, Ubani BC*, et a* Implementation and impact of mobile health (mHealth) in the management of diabetes mellitus in Africa: a systematic review protocol *BMJ Open* 2021;**11:**e047556. doi: 10.1136/bmjopen-2020-047556

15 PROSPERO - Register Guidance Notes [Internet]. Available: https://www. crd. york. ac. uk/ PROSPERO/ guidance/ risk_ of_ bias. Asp [Accessed 09 Oct 2020].

16 Sterne JAC, Savović J, Page MJ, Elbers RG, Blencowe NS, Boutron I, Cates CJ, Cheng H-Y, Corbett MS, Eldridge SM, Hernán MA, Hopewell S, Hróbjartsson A, Junqueira DR, Jüni P, Kirkham JJ, Lasserson T, Li T, McAleenan A, Reeves BC, Shepperd S, Shrier I, Stewart LA, Tilling K, White IR, Whiting PF, Higgins JPT. RoB 2: a revised tool for assessing risk of bias in randomized trials. BMJ 2019; 366: l4898.

17 Mao Y, Lin W, Wen J, et al. Impact and efficacy of mobile health intervention in the management of diabetes and hypertension: a systematic review and meta-analysis. BMJ Open Diab Res Care 2020;8: e001225. doi:10.1136/ bmjdrc-2020-001225

18 Stevens S, Gallagher S, Andrews T, Ashall-Payne L, Humphreys L and Leigh S (2022) The effectiveness of digital health technologies for patients with diabetes mellitus: A systematic review. *Front. Clin. Diabetes Healthc*. 3: 936752. doi: 10.3389/fcdhc.2022.936752

Figure

Click here to access/download;Figure;FIGURE 1.tif

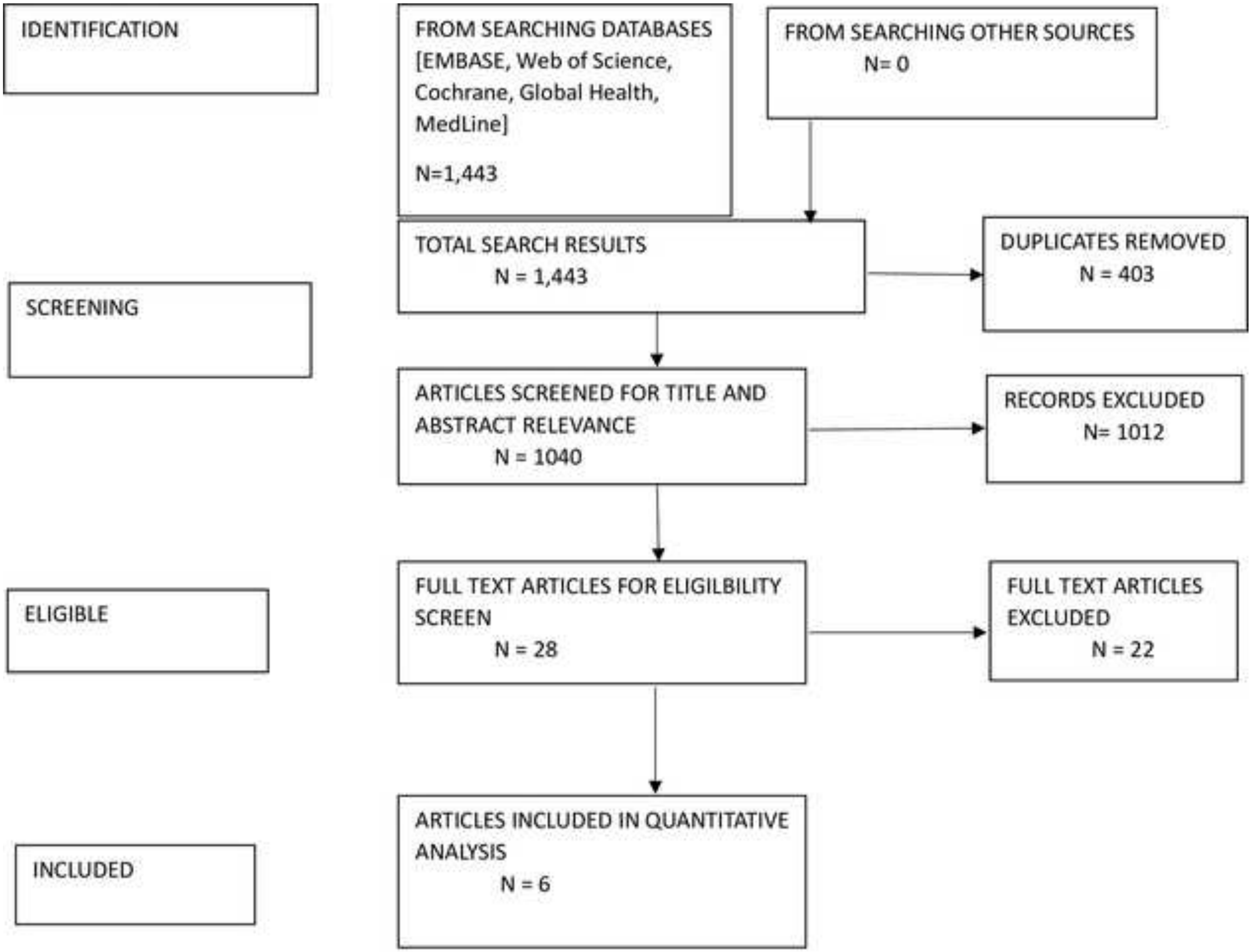

FIGURE 1. FLOW CHART ON SELECTION OF ELIGIBLE STUDIES

 Click here to access/download;Figure;Table 1.tif

Table 1. Characteristics of the eligible studies

Author	Year	Country	Study aim	Study design	Sampling strategy
Haitham et al	2017	Egypt	To examine the feasibility of SMS education among diabetic patients in Egypt, and assess the impact of educational text messages, compared to traditional paper-based methods, on glycemic control and self-management behaviors.	Randomized controlled trial	None given
Ernest Asante et al	2020	Ghana	To evaluate the feasibility and effectiveness of a nurse-led mobile phone call intervention on glycemic management and adherence to self-management practices among patients with type 2 diabetes mellitus (T2DM) in Ghana.	Randomized controlled trial	None given
Matthieu Wargny et al	2018	Senegal	To evaluate the impact of mHealth on diabetes control.	Randomized controlled trial	Consecutive
Owolabi et al	2020	South Africa	To determine the efficacy, acceptability and feasibility of text messaging in improving glycemic control and other clinical outcomes among individuals living with diabetes in low-resource settings in Eastern Cape, South Africa.	Randomized controlled trial	None given
Farmer et al	2021	South Africa	To test the effectiveness of SMS-text messaging in improving outcomes in adults with type 2 diabetes.	Randomized controlled trial	Sequential
Takenga et al	2014	Democratic Republic of Congo	To involve patients in the treatment process and motivate them for active participation.	Randomized controlled trial	Sequential

Figure Click here to access/download;Figure;Table 2.tif

Table 2. Risk of Bias assessment

	Randomization process	Deviation from intended intervention	Missing outcome data	Measure-ment of the outcome	Selection of the reported result	Overall
Haitham et al 2017	low	low	low	low	low	low
Ernest Asante et al 2020	high	low	low	low	high	high
Matthieu Wargny et al 2018	low	low	low	low	high	low
Owolabi et al 2020	low	low	low	low	low	low
Farmer et al 2021	Some concerns	low	low	low	low	low
Takenga et al 2014	low	low	low	low	low	low

 Click here to access/download;Figure;Table 3 (1).tif

	and other clinical outcomes among individuals living with diabetes in low-resource settings in Eastern Cape, South Africa.				
Farmer et al	To test the effectiveness of SMS-text messaging in improving outcomes in adults with type 2 diabetes.	Randomized controlled trial	Reaching remotely via SMS/call	91%	
Takenga et al	To involve patients in the treatment process and motivate them for active participation	Randomized controlled trial	Direct feedback	100%	20.53 % HbA1c reduction

Click here to access/download;Figure;Table 3.tif

Table 3. Summary of studies on interventions and efficacy of the mHealth delivery

Author/Year/ Country	Study aim	Study design/Sampling strategy	mHealth tool/strategy	mHhealth acceptance/use /response rate	mHhealth intervention outcomes
Haitham et al	To examine the feasibility of SMS education among diabetic patients in Egypt, and assess the impact of educational text messages, compared to traditional paper-based methods, on glycemic control and self-management behaviours.	Randomized controlled trial	Reaching remotely via SMS/call	74.4%	10.7 % HbA1c reduction
Ernest Asante et al	To evaluate the feasibility and effectiveness of a nurse-led mobile phone call intervention on glycemic management and adherence to self-management practices among patients with type 2 diabetes mellitus (T2DM) in Ghana.	Randomized controlled trial	Reaching remotely via SMS/call	100%	15.8 % HbA1c reduction
Matthieu Wargny et al	To evaluate the impact of mHealth on diabetes control.	Randomized controlled trial	Reaching remotely via SMS/call	98%	3.6 % HbA1c reduction
Owolabi et al	To determine the efficacy, acceptability and feasibility of text-messaging in improving glycemic control	Randomized controlled trial	Reaching remotely via SMS/call	100%	

Click here to access/download
Supporting Information
PRISMA_2020_checklist.docx

Click here to access/download
Supporting Information
Updated data from included articles.xlsx

Click here to access/download
Supporting Information
ROB2_IRPG_beta_v9.xlsm
