# Supplementary material for: Implementation and impact of mhealth in the management of diabetes mellitus in Africa: A systematic review and meta-analysis
Source: PLOS Digit Health. 2025 Apr 8;4(4):e0000776. doi: 10.1371/journal.pdig.0000776 (PMC11978007; doi:10.1371/journal.pdig.0000776)
Supplement: Attachment — Submitted filename: Authors Responses to Reviewers comments..docx [file pdig.0000776.s007.docx]

**Responses to Reviewers’ Comments**

**Journal Requirements:

1. Please provide separate figure files in .tif or .eps format only and remove any figures embedded in your manuscript file. Please also ensure that all files are under our size limit of 10MB.**

**Authors’ response: This has been implemented as requested. Figures now provided separately in tif format.

For more information about figure files please see our guidelines:**

[**https://journals.plos.org/digitalhealth/s/figures**](https://journals.plos.org/digitalhealth/s/figures)
[**https://journals.plos.org/digitalhealth/s/figures#loc-file-requirements**](https://journals.plos.org/digitalhealth/s/figures#loc-file-requirements) **2. Tables should not be uploaded as individual files. Please remove these files and include the Tables in your manuscript file as editable, cell-based objects. For more information about how to format tables, see our guidelines:**

**Authors’ response: All tables have been included in the manuscript according to guideline. Thank you.**

[**https://journals.plos.org/digitalhealth/s/tables**](https://journals.plos.org/digitalhealth/s/tables) **Additional Editor Comments (if provided):**

**Reviewer #1: Yes: Shlomo Yeshurun
Reviewer #1: The paper by Dike et al. addresses an important issue, the feasibility and effectiveness of mobile health services for diabetics in Africa. This systematic review was originally planned to be conducted as a meta-analysis. However, no analysis was conducted due to the low number of studies found.
The authors are correct in recommending the need for more trials, but they fail to address the major criteria for publication, as well as some other important considerations.
Firstly, the authors did not seem to follow the journal’s guidelines as described here:** [**https://journals.plos.org/digitalhealth/s/submission-guidelines#loc-systematic-reviews-and-meta-analyses**](https://journals.plos.org/digitalhealth/s/submission-guidelines#loc-systematic-reviews-and-meta-analyses)

**Authors’ response: Thank you very much. In reference to the link above, we have done the following:Included a PRISMA checklist (in supporting information) and flow diagram.**

**Stated the existence of our protocol in the method section, with citation and reference, in addition to including the registry number in the abstract. We have also added a copy as supporting information.**

**Our cover letter states unequivocally that this is a systematic review.**

**Notably, there is no numbering of the lines,**

**Authors’ response: Thank you. Numbering of the lines has been inputed as advised in the manuscript.**

**References have not been correctly cited,**

**Authors’ response: References citations have been re-organised**

**And there is no author's summary.**

**Authors’ response: Authors’ summary has been included. Thank you very much.**

**There is also no mention of the countries of affiliations.**

**Authors’ response: Countries of affiliations have been included in the manuscript. Thank you.**

**A major problem is that the main articles mentioned in the manuscript, the six that were retrieved, are not included in the references section, making it difficult to review the paper.**

**Authors’ response: The included studies have been appropriately cited and included in the references. Thank you.**

**I also believe that the authors should mention that they only refer to type 2 diabetes in the title and throughout the manuscript.**

**Authors’ response: Thank you. The review is on diabetes mellitus (type 1 and 2 diabetes) as reflected in the search strategy. However, the available articles are mainly on type 2 diabetes. We have made appropriate corrections across the manuscript.**

**Other notes per sections in the manuscript:
Introduction
While the introduction is comprehensive and nicely summarizes the literature and the objectives of the paper, it is a bit unclear the way it is structured (there is no separation of paragraphs), and perhaps longer than needed. I feel that from “mHealth employs…” until “...GOe survey in 2005” is a bit too much of information and should be reduced to make it clearer.**

**Authors’ response: Thank you very much. This has been addressed.**

**Methods
The methods section is very clear and gives a good idea of the search strategy the authors used. Minor suggestions include moving the “Intervention” and “Outcomes” sections after the “Search strategy and selection criteria” section.**

**Authors’ response: This has been revised in the manuscript.**

**In addition, the authors should add a reference after mentioning the WHO and IDF guidelines in the first section.**

**Authors’ response: References have been added for above guidelines.**

**Results
In my opinion, this section needs to be substantially revised. As mentioned earlier, the primary articles of the review are not referenced, which makes understanding its scope difficult.**

**Authors’ response: Primary articles have been referenced.**

**It is also important to note that, since only two studies reported significant outcomes, table 3 may be misleading as it indicates the average outcome of the intervention. The average should at the very least be noted along with the confidence intervals so that readers can determine the nature of the results.**

**Authors’ response: Weighted mean differences have been added with confidence intervals.**

**Additionally, these non-significant studies should be mentioned in the text with caution.**

**Authors’ response: Thank you for this.**

**The duration of each intervention in the table should also be noted since there are only six studies. A simple description of their length of time between 8 and 52 weeks is insufficient to provide a comprehensive view of them.**

**Authors’ response: The duration of each study was included in the data file added as supplementary information. This has now been added to Table 3.**

**Other minor comments:
1.    There are no references to the supplementary files in the text.**

**Authors’ response: Noted. Supplementary files referenced.**

**2.    In the first paragraph, first mention how many articles were found (1,443) and then mention how many were excluded (1,012) and how many left.**

**Authors’ response: Thank you. Tis have been revised accordingly in the manuscript.**

**3.    In the text before table 3 “One of the studies did not…” – need to mention which one it is.**

**Authors, response: The study is now mentioned.**

**4.    Also mention which studies found significance and which did not.**

**Authors’ response: The studies are now mentioned.**

**Discussion
While the implications in this section are compelling, it would be useful to elaborate on why more of these studies should be conducted in Africa. Additionally, this section lacks references. In addition, I believe that since most people intend to use smartphones in 2023 and thus applications for diabetes, it would be worthwhile to mention the limitations of the SMS/call approach in the discussion, since smartphone applications may prove to be more useful in treating T2D.**

**Authors’ response: The discussion has been updated and references updated.**

**Conclusion
In my opinion, this review does not provide strong evidence that mHealth is beneficial in improving HbA1c for diabetics in Africa (only six studies were reviewed, and only two of those showed significance). According to this study, the only main conclusion is that more RCTs are necessary, primarily because there is a lack of evidence at this time.**

**Authors’ response: This has been addressed and edited. Thank you very much.**

**Reviewer #2: Yes: Eric Mugambi
Reviewer #2: Article is well written, statistically sound and supporting information is accessible though macros in Excel are blocked by microsoft on my machine. It is unfortunate but illuminating that there is a dearth of published studies in this field. Refer to attached PDF for minor proposals**

**Authors’ response: Thank you very much. The proposals are acknowledged and addressed.


Reviewer #3: The authors present the results of a rigorous systematic literature review. For the huge amount of work that went into this systematic literature review, I was surprised about the shortness of the results. Would it be possible to add some graphs or report some other characteristics of the studies, for example some baseline characteristics of the study populations? Although the authors write that there were not enough data, I was still wondering if it would be possible to perform a meta-regression on the outcome? The outcome of two references is missing in Table 3, so I assume it was not HbA1c but another endpoint. By using the ratio of means instead of the mean the difference in outcomes could be overcome. Then there would be 6 studies included in the meta-analysis, which seems to be a reasonable number despite high heterogeneity. I also miss a visualisation in this manuscript. Please consider adding one. For example, a plot including the ratio of means (y-axis)
in dependence with the duration of follow-up (x-axis), maybe with different sizes of the markers to visualise the different sample sizes, would be interesting. Or a boxplot visualising differences in interventional and control groups.**

**Authors’ response: We have performed a meta-analysis of the available results and updated the manuscript. Included is forest plot for easy visualization.**

**In addition, or alternatively, would it be feasible to compare the African context with another one, e.g. Latinamerican, Asian, and then perform a stratified meta-analysis?**

**Authors’ response: Thank you for the suggestion. However, in addition to knowledge dissemination through this systematic review, we intend to highlight the huge gaps in the utilisation of mHealth in the management of diabetes mellitus (DM)in Africa, which may further initiate discussions around policy formulation and funding investments in mHealth intervention in DM research. Therefore, we intend to conduct a systematic review in the future comparing the utilisation of mHealth in DM management in Africa and other populations (Latin America, Asia and the Caribbean).

These are only a few suggestions that might increase the impact and quality of the manuscript.

Minor comments:
Affiliations: please add country to the affiliations**

**Authors’ response: country affiliations have been added, see main text. Thank you


Introduction:
I suggest writing clearly that this article is about type 2 diabetes and does not refer to type 1 diabetes.**

**Authors’ response: The article is about diabetes mellitus (type 1 DM and type 2 DM. All the inconsistencies have been revised in the main text. Thank you


It is not clear if the sentences following “It showed that mHealth was only just emerging in many member states...” are referring to the present situation or** **the survey in 2005; please clarify in the text.**

**Authors’ response: we have revised the sentence in the main text. Thank you


I suggest writing the objectives in a dedicated paragraph instead of listing them up.**

**Authors’ response: we have revised the objectives in a paragraph format as follows:** **The objectives of this systematic review was to determine the extent of implementation of mHealth strategies, and the common forms of mHealth used in the management of DM in patients in Africa, to determine the positive impact/efficacy of mHealth interventions in helping patients achieve treatment goals in diabetes in Africa, as well as to determine the impact of mHealth on early detection, management and outcome of some diabetic complications. Furthermore, the study sought to identify any factors impeding the utilization and implementation of mHealth in Africa in the management of patients with DM and to determine the possible harms and cost of implementation of mHealth strategies in Africa.**

**Thank you


Methods:
Outcome measures: please introduce the abbreviation FPG before the first use**

**Authors’ response: FPG- fasting plasma glucose- revised in the main text. Thank you**

**Outcome measures: what is the conventional therapy?**

**Authors’ response: conventional therapy means standard treatment of diabetes mellitus.**

**Outcome measures: “However, most of the studies included...” This is a result and should not be in the methods section.
Data Synthesis: what ist meant by “expressed statistically”? Please rephrase**

**Authors’ response: We have rephrased the “expressed statistically”?


Results:
Characteristics of included studies: “None of the observational studies met the eligibility criteria.” Why is this sentence here, as the paragraph refers to the characteristics of the included studies?**

**Authors’ response: paragraph revised in the main text. Thank you

Characteristics of included studies: “All the eligible studies investigated a mHealth intervention using a similar mHealth delivery strategy [SMS text messaging]…” “all” meaning four out of six?**

**Authors’ response: paragraph revised in the main text. Thank you

Table 1: it would be nice to see the sample size. In addition, it would be nice to add the information regarding the mHealth delivery strategy in a separate column for better overview.

Efficacy of the interventions and efficacy of the mHealth delivery strategies: “Five of the six studies…” which study are you referring to? Please add the reference. The same applies to the next sentence. Idem for “The study with the largest sample size of 1186 had a 91% response rate.”

Table 1 and Table 3 contain almost the same information, please get rid of the redundant information.

Table 3: what was the outcome in Owolabi and what in Farmer?

Authors’ response: The data requested were included in the data file uploaded as supporting information, ie sample sizes, delivery strategy and outcomes. We have updated the table in the manuscript with this information.**

**Conclusion:
The study does not support the conclusion “The study shows mHealth is beneficial in improving glycemic control (HbA1c) in patients with diabetes mellitus and its utilization could be essential in preventing and or delaying diabetes complications.” We would need to see the results of a meta-analysis to assess this.**

**Authors’ response: We have performed a meta-analysis with the available data as requested. The results are included in the manuscript.**

**Reviewer #4: No**

**Reviewer #4: Thank you for this important Review concerning mHealth in Diabetes in Africa. Only six randomized trials was selected in this carefully performed process. This is really well done and the paper is ready to get published. Hopefully we will get more data within this field in the future, mHealth is probably a most important tool.**

**Authors’ response: Thank you very much.**
