# Supplementary material for: Implementation and impact of mhealth in the management of diabetes mellitus in Africa: A systematic review and meta-analysis
Source: PLOS Digit Health. 2025 Apr 8;4(4):e0000776. doi: 10.1371/journal.pdig.0000776 (PMC11978007; doi:10.1371/journal.pdig.0000776)
Supplement: Attachment — Submitted filename: Responses to Reviewers Comments 2.docx [file pdig.0000776.s010.docx]

Responses to Reviewers

Line 49 - utilisation vs utilization - in other parts of manuscript American translation of similar sounding words is used e.g. standardized in line 309. It would be useful to stick to one version.

Response: Spellings are now standardised in the manuscript to British format. Thank you.

Line 54 - Proposed "A third independent reviewer..."

Response: Revised as advised in the main text. Thank you.

Lines 61/62 - as 'range' automatically denotes min to max, authors can omit the words least and highest

Response: Revised as advised in the main text. Thank you.

Line 64 - ... HBA1C weighted mean difference (WMD)...

Response: Revised as advised in the main text. Thank you.

Line 68 - instead of using "/" as in prevention/early detection, suggest using "or" or "and" as appropriate.

Response: Revised as advised in the main text. Thank you.

Line 73 - You might want to rephrase your conclusion for conciseness: e.g. The research indicates that mHealth shows promise in optimizing glycated haemoglobin levels among diabetes patients in Africa... These findings offer valuable insights for policymakers regarding the integration of mHealth strategies in African healthcare systems. Additionally, the systematic review underscores the limited availability of data on the utilization and implementation of mHealth in diabetes care across Africa.

Response: Revised as advised in the main text.

Line 81 - instead of "/" use "and"

Response: Revised as advised in the main text.

Line 91 - not sure if the word "impact" adds value here.

Response: Revised as advised in the main text. Thank you.

Line 92 - Propose avoid the word "etc" e.g. Various tools such as text messages, phone calls, mobile applications, and wearable devices have been employed to implement this strategy.

Response: Revised as advised in the main text. Thank you.

Line 96 - We systematically searched major databases....

Response: Revised as advised in the main text. Thank you.

Line 101 to 105 - rephrase for impact e.g. The primary takeaway from these findings underscores the necessity for further efforts to gather sufficient data, enabling more comprehensive research and conclusive outcomes.

Response: Revised as advised in the main text. Thank you.

Line 124 - propose avoid "/"

Response: Revised as advised in the main text. Thank you.

Lines 144-146 - rephrase e.g. A recent survey conducted across six countries in Africa, including South Africa, Nigeria, Tanzania, Ghana, Senegal, and Kenya, revealed penetration rates of 91% in South Africa, 80% in Nigeria, and 75% in Tanzania, the latter being the lowest among the surveyed nations.

Response: Revised as advised in the main text. Thank you.

Line 147 - review use of word "phenomenal"

Response: The word "phenomenal" has been replaced by “dramatic” in the main text. Thank you.

Lines 154-155: Bring out the true impact of DM in Africa - e.g. as per reference 5, Diabetes Mellitus remains a key economic and developmental challenge in Africa, with estimated costs of ............ in the year.............

Response: We agree. We have revised as advised in the main text. Thank you.

Line 155 - in the context of this study focussing on diabetic patients, focus is on secondary and tertiary prevention and not primary

Response: Thank you for the comment.

Line 174 - the dash is unnecessary.

Response: Revised as advised in the main text. Thank you.

Line 174 - "nine the same"? delete word "nine”.

Response: Revised as advised in the main text. Thank you.

Line 199 to 205 - suggested: The study aimed to assess the impact of mHealth interventions on managing Diabetes Mellitus in Africa, including their effectiveness in achieving treatment goals and their influence on early detection and management of diabetic complications. It also sought to identify barriers to mHealth utilization in Africa and evaluate the potential harms and costs of implementing mHealth strategies.

Response: Thank you for the useful suggestion.

Line 200 - Replace dash with "or"?

Response: Revised as advised in the main text. Thank you.

Line 204 - utilisation vs utilization - standardize across board.

Response: Spellings have been standardised in the main text. Thank you.

Line 221 - not sure whether the studies had Africans in the inclusion criteria or were just conducted in Africa - Clarify and convey appropriate message

Response: Revised as advised in the main text. Thank you.

246-254 - Rephrase for clarity e.g. We conducted searches across several electronic databases to identify relevant studies on the implementation of mHealth for Diabetes Mellitus (DM) in Africa, covering the period from January 2010 to October 2020. Specifically, we searched the

Medline database (PubMed), the Cochrane Register of Controlled Trials (CENTRAL), Embase (Scopus), and the CINAHL database. Additionally, we explored the Pan African Clinical Trials Registry (PACTR) for ongoing and recently completed but unpublished studies meeting our criteria.

In our search strategy for the Medline database, we utilized Medical Subject Headings (MeSH) to identify all available studies related to mHealth and DM. We included terms representing various mHealth categories and applied filters for DM and African regions and countries.

Response: Thank you for the useful input. We have made relevant changes to the main text.

Line 290 - 297: Rephrase, e.g. The primary focus was on assessing the effectiveness of integrating mHealth interventions into treatment plans, comparing changes in HbA1c or FPG levels between intervention and control groups. Secondary outcomes aimed to measure reductions in DM complications, but most studies did not address this, nor did they specifically investigate mHealth strategies targeting these outcomes.

Response: Thank you for the useful suggestion. We have made relevant changes to the main text.

Line 302 - that met.... (not meet)

Response: Revised as advised in the main text. Thank you.

Line 304 - "reassessed for eligibility" instead of assessed for eligibility again.

Response: Revised as advised in the main text. Thank you

Line 309, 310 - We utilized a standardized Microsoft Word form for data extraction, quality assessment of studies, and evidence synthesis.

Response: Revised as advised in the main text. Thank you

326-338: - The assessments were conducted using standardized processes to ensure objectivity across various domains. These included evaluating the concealment of treatment allocation, blinding of personnel involved in outcome assessment and data analysis, completeness of outcome data, presence of selective outcome reporting, and identification of other potential sources of bias.

Response: Revised as advised in the main text. Thank you

Line 401 - you mention 2021 whilst your inclusion criteria was January 2010 and October 2020

Response: Thank you. We have revised it in the main text.

Line 305 - can just say "a third reviewer”.

Response: Revised as advised in the main text. Thank you

Lines 464-466 - indicate the study focussed on individuals with Diabetes.

Response: Revised as advised in the main text. Thank you

Lines 470 - 474 - integrate within conclusion

Response: Thank you. Section integrated within the conclusion.

Reviewer 1:

Major

While I understand that the aim of the study was to search for both types of diabetes, since the 6 studies only tested type 2 diabetes, all results, discussion, and conclusions section should highlight this fact.

Response: This has been adequately addressed in the results, discussion, and conclusions as advised in the main text. Thank you.

Furthermore, there are still many unsuitable references throughout the manuscript. Notably, in line 134 (introduction) the references do not support the statement. I believe the authors should look carefully at each reference and its relevance, in particular in the introduction and discussion sections.

Response: Thank you. We have revised it as advised in the main text.

Please mention if achieved significant for Hba1c for each paper mentioned in the results section under “EFFICACY OF THE INTERVENTIONS AND EFFICACY OF THE MHEALTH DELIVERY STRATEGIES”. I believe this is important to understand these studies with cautious.

Response: Thank you for the comment. We have made an attempt to show that HbA1c achieved significance in five studies as follows:

“The percentage change in HbA1c from the baseline ranged from 3.6% to 20.53%, achieving significance in the studies by Takenga et al., Wargny et al., and Asante et al. A meta-analysis of the pooled sample of 5 studies with a total participant size of 3112 excluding the article by Owolabi et al. which measured random glucose as its primary outcome was done with. There was a significant effect size in HbA1c WMD after mHealth intervention, with an overall WMD (95% CI) of 0.992 (0.48, 1.50).”

I believe that after a comprehensive analysis about this important topic, the discussion should be expanded, mostly with suggestions about future studies and how they should be conducted with relevance to this day and age. In addition, the strength and limitation’s part should be much further explained. In particular they should mention the large differences between the six studies in their population, length, methodology, and results. They could also mention the limitations of SMS and why smartphone apps can do a better job.

While the authors responded that the conclusion part has been edited, I don’t see any changes. I think that since only 6 studies have been reviewed with large differences between them, words like “demonstrates” and “essential” should be avoided. In my understanding, the main conclusion from this review is that future research is urgently needed since it’s impossible to conclude anything about the impact of mHealth for diabetes in Africa at the current stage.

Response: Thank you for your comment. The conclusion section has been revised as follows:

The study indicates that mHealth shows promise in optimising glycated haemoglobin levels among diabetes patients in Africa. These findings offer valuable insights for policymakers regarding the integration of mHealth strategies in African healthcare systems. Interestingly, all the eligible studies only tested type 2 diabetes. Additionally, the systematic review emphasises the limited availability of data on the utilisation and implementation of mHealth in diabetes care across Africa. We therefore recommend more well-designed randomised controlled trials to investigate mHealth interventions and strategies in the management of diabetes mellitus in Africa.

Minor

The Author’s Summary is well-written and a nice addition to the manuscript. Still, I would suggest looking at similar articles in PLOS Digital Health as it should be more concise and in one paragraph.

While the authors made some changes to the introduction, they have not addressed my comment regarding lines 116 to 120. Please consider modifying this.

Response: Revised as advised in the main text. Thank you

In addition, I believe that most cited papers are outdates regarding the study aims. Since the manuscript it about Digital Health, it’s important to look for more recent surveys and meta-analysis as technology advanced rapidly since (most cited studies are before the age of smartphones).

Response: Thank you. We have expanded the main text as advised.

In the results section, please add a reference each time a study is mentioned (line 315 for example).

Response: Thank you. We have added the references as suggested.

Thank you for adding the extra information in the tables. Please consider merging tables 1 and 3 into one table as some of the information is repeatable. Also, the heading of the first column in table 3 does not reflect the contents. I would also suggest another column of “Significance” with Yes/No for it.

Response: Thank you for the suggestion. We have revised Tables 1 and 3 to improve clarity in the main text. We suggest both tables stand alone, as each represents specific information.

I still don’t see a reference in the manuscript for the supplementary files. Please make sure that they are mentioned correctly.

Response: Thank you for the comment. However, the supplementary files we uploaded are PRISMA and RoB, which we plotted and may not require citation.

Reviewer 2:

The manuscript is coherent but does not make for easy reading - I have provided examples of how certain critical area can be rephrased. It may be useful to have a technical writer improve the flow of text without altering the core structure or message.

Response: Thank you so much for your useful suggestions. We have adapted them and revised the relevant sections in the main text as suggested.

Reviewer #3: Comments regarding meta-analysis:

Thank you for doing a meta-analysis. It would be nice if the authors would describe the meta-analysis in more detail in the “data synthesis” section. On what was the meta-analysis done (mean difference of what); using which statistical package was the meta-analysis done. The authors should explain how metrics of heterogeneity will be interpreted (e.g. I^2). I suggest adding the corresponding reference (e.g. Higgins JPT, Thompson SG, Deeks JJ, Altman DG. Measuring inconsistency in meta-analyses. BMJ 2003; 327: 557–60.)

Response: Thank you for the compliment and suggestion. The meta-analysis was done on the mean difference of HbA1c (in %). The STATA 18 statistical package was used to perform the meta-analysis. The interpretation of heterogeneity metrics is as follows: I^2^ of at least 75% - high heterogeneity, I^2^ between 50-75% - moderate heterogeneity, and I^2^ of less than 50% - little to no heterogeneity. These changes have been embedded in the main text.

We have also added the reference in the main text.

In the forest plot of the meta-analysis there is a missing space between study author and year of publication for Asante and Takenga. The abbreviation DRC and WMD should be explained in the figure legend. I suggest to also add the sample size in the graph.

Response: Thank you for the suggestion. The meta-analysis forest plot has been amended as suggested.

In the flowchart, it says “articles included in quantitative analysis = 6”. What is meant by “quantitative analysis” and how does this differ from meta-analysis? The flowchart should also include the meta-analysis (by adding one more row for example).

Response: Thank you. The flowchart has been revised as suggested.

Other comments:

The abbreviation “DM” is used without introducing it (in the main text; it seems that the introduction has been deleted in the revised version of the manuscript)

Response: Thank you. We have revised it in the main text.

It is still not clear that the systematic review and meta-analysis are referring to type 2 diabetes. I suggest adding “type 2” in the title

Response: Our systematic review was aimed at investigating the extent of mHealth implementation in managing diabetes mellitus in Africa, but we found that all six eligible studies focused exclusively on type 2 diabetes. This highlights the lack of data on the impact and implementation of mHealth in other types of diabetes and, therefore, calls for further research. We believe the current topic and our findings effectively underscore this gap.

Lines 356-358 is difficult to understand; please review.

Response: Thank you. We have revised it in the main text.
