# Supplementary material for: Implementation and impact of mhealth in the management of diabetes mellitus in Africa: A systematic review and meta-analysis
Source: PLOS Digit Health. 2025 Apr 8;4(4):e0000776. doi: 10.1371/journal.pdig.0000776 (PMC11978007; doi:10.1371/journal.pdig.0000776)
Supplement: Attachment — Submitted filename: Reviewers Comments 20122024.docx [file pdig.0000776.s011.docx]

**RESPONSES TO REVIEWER’S COMMENTS**

**Reviewer #1**: Abstract

1) The use of the term “trend” should not be used.

***Please mention if reached significance or not.

***Please clearly indicate whether the meta-analysis results are statistically significant.

This is crucial for understanding the validity of your findings.

Response: we have revised the abstract as advised, see main text. Thank you

2) The conclusions section:
***Consider making it more concise to fit the abstract format.
***Ensure that the conclusions in the abstract match exactly with those in the discussion section for consistency.
***It is essential to explicitly mention type 2 diabetes in the conclusions to avoid any ambiguity regarding the scope of your study.

Response: We have revised the conclusion section, with type 2 diabetes explicitly mentioned. Thank you

Authors’ Summary

This section still does not conform to the style and format typical of similar articles in PLOS Digital Health.

***It should be more concise and structured into a single paragraph.

I urge you to revise this section to meet these standards.

Response: Author summary revised to conform to the prescribed format. It was made more concised and structured into a single paragraph. Thank you

Introduction

1) For the second time,

****the introduction has not been updated as requested.

***The first and second paragraphs contain outdated information and do not reflect the latest advancements and definitions in mHealth as of 2024.

***It is imperative to include recent surveys, definitions, and relevant manuscripts to provide a current context.

Response: Introduction updated, with major changes in the first and second paragraphs to reflect latest definitions and advancement. An attempt has been made to provide a hopefully more satisfactory current context by including recent surveys and articles. Thank you

2) While the changes in the rest of the introduction are appreciated,

***the paragraph before the last (line 163) feels disconnected from the rest of the introduction.

***Please either expand on this subject to integrate it seamlessly with the other paragraphs or consider relocating it to the discussion section where it might be more relevant.

Response: We have made relevant changes. Thank you

Results

3) ***There are still instances where studies are mentioned without proper references, particularly in lines 325 and 326.

**It is crucial to provide references for all mentioned studies to ensure the credibility and traceability of your work.

Response : We have updated all cited references. Thank you

4) Thank you for adding the significance of the findings.

***However, it is still unclear whether the significance indicated in Table 3 pertains to the WMD or if it was determined within the individual studies. ***Please clarify this in ---the table for better understanding.

Response: The significance in the table relates to the individual studies, while the WMD is from the meta-analysis. We have now indicated these in the table for better clarity. Thank you

Discussion

1) While the discussion section has been expanded, it still requires further enhancement. Specifically,

****I recommend including detailed suggestions for future studies, highlighting how they should be conducted in the context of current technological advancements and healthcare needs.
2) The strengths and limitations section needs a more comprehensive explanation.

****It is important to discuss the significant differences among the six studies regarding their population, length, methodology, and results. ****Furthermore, addressing the limitations of SMS-based interventions and discussing why smartphone applications might offer better solutions would add significant value to this section.
I hope these detailed comments provide clear guidance for your revisions. Ensuring these points are adequately addressed will greatly enhance the quality and impact of your manuscript.

Response: Thank you for the very helpful suggestions. Some recommendations have been added in the above context. In addition we have added more detailed explanation to the strengths and limitation section which we hope will be adequate. Thank you

**Reviewer #2**: The Authors have satisfactorily adjusted the article based on my recommendations.

Response: Thank you very for your kind comments.

**Reviewer #3**:

Unfortunately the authors didn't address my previous comments.

****Stata is not a statistical package, but a statistical software. The authors should

Response: Thank you very much for this observation. This has been revised in the manuscript. Thank you

****add the reference (can be found via google) and

Response: Reference to STATA added/cited. Thank you

****explain which statistical package/function/command they used for their meta-analysis (can also be found via google).

Response: the “metan” command in STATA was used. This is now reflected in manuscript. Thank you

****The interpretation of the I^2 should also include a reference (pubmed!)

Response : Interpreted and reference added. Thank you

****Figure 2 is still not adequate (missing spaces between author and year).

Response: Space added between authors and year in table 2. Thank you

****lines 324/380/450: I don't think "tested" is the correct wording in this context, as it is likely the app that was assessed. ****What about "included"?

Response: the word “tested” replaced by included/researched as indicated. Thank you.

**OTHER COMMENTS IN ATTACHMENT**

Line 49 - utilisation vs utilization - in other parts of manuscript American translation of similar sounding words is used e.g. standardized in line 309. It would be useful to stick to one version.

Response: Spellings are now standardized in the manuscript to American format. Thank you.

Line 54 - Proposed "A third independent reviewer..."

Response: Revised as advised in the main text. Thank you.

Lines 61/62 - as 'range' automatically denotes min to max, authors can omit the words least and highest

Response: Revised as advised in the main text. Thank you.

Line 64 - ... HBA1C weighted mean difference (WMD)...

Response: Revised as advised in the main text. Thank you.

Line 68 - instead of using "/" as in prevention/early detection, suggest using "or" or "and" as appropriate.

Response: Revised as advised in the main text. Thank you.

Line 73 - You might want to rephrase your conclusion for conciseness: e.g. The research indicates that mHealth shows promise in optimizing glycated haemoglobin levels among diabetes patients in Africa... These findings offer valuable insights for policymakers regarding the integration of mHealth strategies in African healthcare systems. Additionally, the systematic review underscores the limited availability of data on the utilization and implementation of mHealth in diabetes care across Africa.

Response: Revised as advised in the main text.

Line 81 - instead of "/" use "and"

Response: Revised as advised in the main text.

Line 91 - not sure if the word "impact" adds value here.

Response: Revised as advised in the main text. Thank you.

Line 92 - Propose avoid the word "etc" e.g. Various tools such as text messages, phone calls, mobile applications, and wearable devices have been employed to implement this strategy.

Response: Revised as advised in the main text. Thank you.

Line 96 - We systematically searched major databases....

Response: Revised as advised in the main text. Thank you.

Line 101 to 105 - rephrase for impact e.g. The primary takeaway from these findings underscores the necessity for further efforts to gather sufficient data, enabling more comprehensive research and conclusive outcomes.

Response: Revised as advised in the main text. Thank you.

Line 124 - propose avoid "/"

Response: Revised as advised in the main text. Thank you.

Lines 144-146 - rephrase e.g. A recent survey conducted across six countries in Africa, including South Africa, Nigeria, Tanzania, Ghana, Senegal, and Kenya, revealed penetration rates of 91% in South Africa, 80% in Nigeria, and 75% in Tanzania, the latter being the lowest among the surveyed nations.

Response: Revised as advised in the main text. Thank you.

Line 147 - review use of word "phenomenal"

Response: The word "phenomenal" has been replaced by “dramatic” in the main text. Thank you.

Lines 154-155: Bring out the true impact of DM in Africa - e.g. as per reference 5, Diabetes Mellitus remains a key economic and developmental challenge in Africa, with estimated costs of ............ in the year.............

Response: We agree. We have revised as advised in the main text. Thank you.

Line 155 - in the context of this study focussing on diabetic patients, focus is on secondary and tertiary prevention and not primary

Response: Thank you for the comment.

Line 174 - the dash is unnecessary.

Response: Revised as advised in the main text. Thank you.

Line 174 - "nine the same"? delete word "nine”.

Response: Revised as advised in the main text. Thank you.

Line 199 to 205 - suggested: The study aimed to assess the impact of mHealth interventions on managing Diabetes Mellitus in Africa, including their effectiveness in achieving treatment goals and their influence on early detection and management of diabetic complications. It also sought to identify barriers to mHealth utilization in Africa and evaluate the potential harms and costs of implementing mHealth strategies.

Response: Thank you for the useful suggestion.

Line 200 - Replace dash with "or"?

Response: Revised as advised in the main text. Thank you.

Line 204 - utilisation vs utilization - standardize across board.

Response: Spellings have been standardised in the main text. Thank you.

Line 221 - not sure whether the studies had Africans in the inclusion criteria or were just conducted in Africa - Clarify and convey appropriate message

Response: Revised as advised in the main text. Thank you.

246-254 - Rephrase for clarity e.g. We conducted searches across several electronic databases to identify relevant studies on the implementation of mHealth for Diabetes Mellitus (DM) in Africa, covering the period from January 2010 to October 2020. Specifically, we searched the

Medline database (PubMed), the Cochrane Register of Controlled Trials (CENTRAL), Embase (Scopus), and the CINAHL database. Additionally, we explored the Pan African Clinical Trials Registry (PACTR) for ongoing and recently completed but unpublished studies meeting our criteria.

In our search strategy for the Medline database, we utilized Medical Subject Headings (MeSH) to identify all available studies related to mHealth and DM. We included terms representing various mHealth categories and applied filters for DM and African regions and countries.

Response: Thank you for the useful input. We have made changes to the main text.

Line 290 - 297: Rephrase, e.g. The primary focus was on assessing the effectiveness of integrating mHealth interventions into treatment plans, comparing changes in HbA1c or FPG levels between intervention and control groups. Secondary outcomes aimed to measure reductions in DM complications, but most studies did not address this, nor did they specifically investigate mHealth strategies targeting these outcomes.

Response: Thank you for the useful suggestion. We have made changes to the main text.

Line 302 - that met.... (not meet)

Response: Revised as advised in the main text. Thank you.

Line 304 - "reassessed for eligibility" instead of assessed for eligibility again.

Response: Revised as advised in the main text. Thank you

Line 309, 310 - We utilized a standardized Microsoft Word form for data extraction, quality assessment of studies, and evidence synthesis.

Response: Revised as advised in the main text. Thank you

326-338: - The assessments were conducted using standardized processes to ensure objectivity across various domains. These included evaluating the concealment of treatment allocation, blinding of personnel involved in outcome assessment and data analysis, completeness of outcome data, presence of selective outcome reporting, and identification of other potential sources of bias.

Response: Revised as advised in the main text. Thank you

Line 401 - you mention 2021 whilst your inclusion criteria was January 2010 and October 2020

Response: Thank you. We have revised it in the main text.

Line 305 - can just say "a third reviewer”.

Response: Revised as advised in the main text. Thank you

Lines 464-466 - indicate the study focussed on individuals with Diabetes.

Response: Revised as advised in the main text. Thank you

Lines 470 - 474 - integrate within conclusion

Response: Thank you. Section integrated within the conclusion.
