# Supplementary material for: Implementation and impact of mhealth in the management of diabetes mellitus in Africa: A systematic review and meta-analysis
Source: PLOS Digit Health. 2025 Apr 8;4(4):e0000776. doi: 10.1371/journal.pdig.0000776 (PMC11978007; doi:10.1371/journal.pdig.0000776)
Supplement: Attachment — Submitted filename: Reviewer comments.pdf [file pdig.0000776.s008.pdf]

Line 49 - utilisation vs utilization - in other parts of manuscript American translation of similar sounding words is used e.g. standardized in line 309. It would be useful to stick to one version.

Line 54 - Proposed "A third independent reviewer..."

Lines 61/62 - as 'range' automatically denotes min to max, authors can omit the words least and highest

Line 64 - ... HBA1C weighted mean difference (WMD)...

Line 68 - instead of using "/" as in prevention/early detection, suggest using "or" or "and" as appropriate.

Line 73 - You might want to rephrase your conclusion for conciseness: e.g. The research indicates that mHealth shows promise in optimizing glycated haemoglobin levels among diabetes patients in Africa... These findings offer valuable insights for policymakers regarding the integration of mHealth strategies in African healthcare systems. Additionally, the systematic review underscores the limited availability of data on the utilization and implementation of mHealth in diabetes care across Africa.

Line 81 - instead of "/" use "and"

Line 91 - not sure if the word "impact" adds value here.

Line 92 - Propose avoid the word "etc" e.g. Various tools such as text messages, phone calls, mobile applications, and wearable devices have been employed to implement this strategy.

Line 96 - We systematically searched major databases....

Line 101 to 105 - rephrase for impact e.g. The primary takeaway from these findings underscores the necessity for further efforts to gather sufficient data, enabling more comprehensive research and conclusive outcomes.

Line 124 - propose avoid "/"

Lines 144-146 - rephrase e.g. A recent survey conducted across six countries in Africa, including South Africa, Nigeria, Tanzania, Ghana, Senegal, and Kenya, revealed penetration rates of 91% in South Africa, 80% in Nigeria, and 75% in Tanzania, the latter being the lowest among the surveyed nations.

Line 147 - review use of word "phenomenal"

Lines 154-155: Bring out the true impact of DM in Africa - e.g. as per reference 5, Diabetes Mellitus remains a key economic and developmental challenge in Africa, with estimated costs of ……….. in the year…………

Line 155 - in the context of this study focussing on diabetic patients, focus is on secondary and tertiary prevention and not primary

Line 174 - the dash is unnecessary.

Line 174 - "nine the same"? delete word "nine".

Line 199 to 205 - suggested: The study aimed to assess the impact of mHealth interventions on managing Diabetes Mellitus in Africa, including their effectiveness in achieving treatment goals and their influence on early detection and management of diabetic complications. It also sought to identify barriers to mHealth utilization in Africa and evaluate the potential harms and costs of implementing mHealth strategies.

Line 200 - Replace dash with "or"?

Line 204 - utilisation vs utilization - standardize across board.

Line 221 - not sure whether the studies had Africans in the inclusion criteria or were just conducted in Africa - Clarify and convey appropriate message

246-254 - Rephrase for clarity e.g. We conducted searches across several electronic databases to identify relevant studies on the implementation of mHealth for Diabetes Mellitus (DM) in Africa, covering the period from January 2010 to October 2020. Specifically, we searched the

Medline database (PubMed), the Cochrane Register of Controlled Trials (CENTRAL), Embase (Scopus), and the CINAHL database. Additionally, we explored the Pan African Clinical Trials Registry (PACTR) for ongoing and recently completed but unpublished studies meeting our criteria.

In our search strategy for the Medline database, we utilized Medical Subject Headings (MeSH) to identify all available studies related to mHealth and DM. We included terms representing various mHealth categories and applied filters for DM and African regions and countries.

Line 290 - 297: Rephrase, e.g. The primary focus was on assessing the effectiveness of integrating mHealth interventions into treatment plans, comparing changes in HbA1c or FPG levels between intervention and control groups. Secondary outcomes aimed to measure reductions in DM complications, but most studies did not address this, nor did they specifically investigate mHealth strategies targeting these outcomes.

Line 302 - that met…. (not meet)

Line 304 - "reassessed for eligibility" instead of assessed for eligibility again.

Line 309, 310 - We utilized a standardized Microsoft Word form for data extraction, quality assessment of studies, and evidence synthesis.

326-338: - The assessments were conducted using standardized processes to ensure objectivity across various domains. These included evaluating the concealment of treatment allocation, blinding of personnel involved in outcome assessment and data analysis, completeness of outcome data, presence of selective outcome reporting, and identification of other potential sources of bias.

Line 401 - you mention 2021 whilst your inclusion criteria was January 2010 and October 2020

Line 305 - can just say "a third reviewer”.

Lines 464-466 - indicate the study focussed on individuals with Diabetes.

Lines 470 - 474 - integrate within conclusion.
